# A revision of the cleptoparasitic bee genus *Epeolus* Latreille for Nearctic species, north of Mexico (Hymenoptera, Apidae)

**DOI:** 10.3897/zookeys.755.23939

**Published:** 2018-05-08

**Authors:** Thomas M. Onuferko

**Affiliations:** 1 Department of Biology, York University 4700 Keele St., Toronto, ON M3J 1P3

**Keywords:** cleptoparasitic bee, DNA barcoding, *Epeolus*, morphology, taxonomic revision

## Abstract

Herein, the cleptoparasitic (cuckoo) bee genus *Epeolus* (Hymenoptera: Apidae) is revised for species occurring in North America, north of Mexico, and an updated checklist of all species known to occur in Canada and the United States of America is provided with comprehensive descriptions, diagnoses, and a single dichotomous key (using the same couplets for both sexes) to aid in their identification. To increase their recognition among North American naturalists, English common names are also proposed for all North American *Epeolus*. A total of 43 species is confirmed as present in the region, 15 of which are newly recognized. The following new species are proposed based on unique morphological (and in most cases also molecular) attributes: *E. andriyi* **sp. n.**, *E. attenboroughi* **sp. n.**, *E. axillaris* **sp. n.**, *E. basili* **sp. n.**, *E. brumleyi* **sp. n.**, *E. chamaesarachae* **sp. n.**, *E. deyrupi* **sp. n.**, *E. diadematus* **sp. n.**, *E. ferrarii* **sp. n.**, *E. gibbsi* **sp. n.**, *E. inornatus* **sp. n.**, *E. nebulosus* **sp. n.**, *E. packeri* **sp. n.**, *E. splendidus* **sp. n.**, and *E. tessieris* **sp. n.** Of the 15, six (*E. axillaris*, *E. brumleyi*, *E. chamaesarachae*, *E. diadematus*, *E. splendidus*, and *E. tessieris*) were identified as new species under different names (*nomina nuda*) in an M.Sc. thesis by Richard L. Brumley in 1965, but until now they have not been formally described. Detailed morphological comparisons with some evidence from DNA barcoding support the following synonymies, one of which **C** was first proposed by Brumley (1965): a) *E. melectimimus* Cockerell and Sandhouse, **syn**. **n**., under *E. asperatus* Cockerell; b) *E. crucis* Cockerell, **syn**. **n**., under *E. compactus* Cresson; c) *E. mesillae palmarum* Linsley, **syn**. **n**., under *E. mesillae* (Cockerell); and d) *E. weemsi* Mitchell, **syn**. **n**., and e) *E. vernalis* Mitchell, **syn**. **n**., under *E. ilicis* Mitchell. Only one member of the almost entirely Neotropical “Trophocleptria group” (*Epeolus bifasciatus* Cresson) is confirmed as occurring north of Mexico, and is widespread East of the Rocky Mountains. Known floral associations are indicated for each species, as are suspected or known host species of *Colletes* Latreille. Evidence is presented that suggests further investigation into the possible synonymy of *Colletes wickhami* Timberlake under *C. scopiventer* Swenk is warranted.

## Introduction

*Epeolus* Latreille (Hymenoptera: Apidae, subfamily Nomadinae) is one of the most widespread genera of cleptoparasitic bees (commonly referred to as cuckoo bees), occurring on all continents except Antarctica and Australia. The genus is also absent from Madagascar, Oceania, and parts of Southeast Asia, regions in which their host genus *Colletes* Latreille (Hymenoptera: Colletidae: Colletinae) is not present (Michener 2007). Other genera in the tribe Epeolini are largely restricted to the Americas, mostly to the Neotropical region. The similarly diverse bee genus *Triepeolus* Robertson has only two representatives in the Palearctic region, whereas *Epeolus* is represented across Africa, Asia, and Europe by about 48 species (Ascher and Pickering 2017). However, the genus is most diverse in North America, with 32 valid species confirmed as occurring north of Mexico before the date of this publication.

For North American species, the taxonomy of *Epeolus* has been in need of revision for some time. While Mitchell’s (1962) treatment of the Eastern United States fauna was fairly comprehensive, the Western species have been in much need of attention. In his M.Sc. thesis, Richard L. Brumley (1965) recognized several new species from the Western United States, but his names were never published and are therefore not considered valid. Recently, Onuferko (2017) identified 14 redundant names (most are of Western “species”), which were synonymized under the names of four valid species, but this treatment was limited to the Canadian fauna. The purpose of the present study is to resolve the taxonomy of *Epeolus* occurring in Canada and the USA by naming and describing new species and identifying which accepted names are valid and which are not, thereby standardizing name use, as well as to provide a user-friendly dichotomous identification key. To help amateur and professional entomologists become more familiar with these bees, English common names are proposed for all North American species of *Epeolus*. An additional objective is to present ecological information in terms of floral and *Colletes* hosts and phenology wherever possible, as well as comprehensive occurrence records to aid those interested in locating and identifying representatives of the species treated herein for further research.

## Materials and methods

To revise *Epeolus* an integrative biosystematics approach was followed, using morphological and molecular evidence to distinguish intraspecific from interspecific variation (as in Gibbs 2009, 2010, 2011, Pauly et al. 2014, Rocha-Filho and Packer 2015, Ferrari 2017, Onuferko 2017). Morphological evidence was prioritized over molecular evidence when the two were not in agreement, as in Gibbs (2009). Sequence data from a 658 bp segment of the mitochondrial cytochrome c oxidase subunit I (COI) gene (DNA barcode, Hebert et al. 2003a, b) were obtained from specimens of nearly all (42 out of 43) species, and 37 have sequences that are barcode compliant (i.e., have met the criteria to be assigned automated barcode index numbers (BINs) given to unique barcode clusters, Ratnasingham and Hebert 2007, 2013). One or two legs were removed from each specimen to be “barcoded”, and sent to the Canadian Centre for DNA Barcoding in Guelph, Ontario, Canada for DNA extraction and gene amplification and sequencing. A neighbor-joining (NJ) tree, based on Kimura’s two-parameter distance model (Kimura 1980), was used to compare short, non-compliant and barcode-compliant sequences for the purpose of validating species designations of sequenced specimens and checking for contamination errors. Partial and BIN-compliant sequences are published in the “*Epeolus* of North America project” on the Barcode of Life Data Systems website (http://www.barcodinglife.org/) and have been deposited in the GenBank database (see Suppl. material 1 for accession numbers).

**Figure 1.**
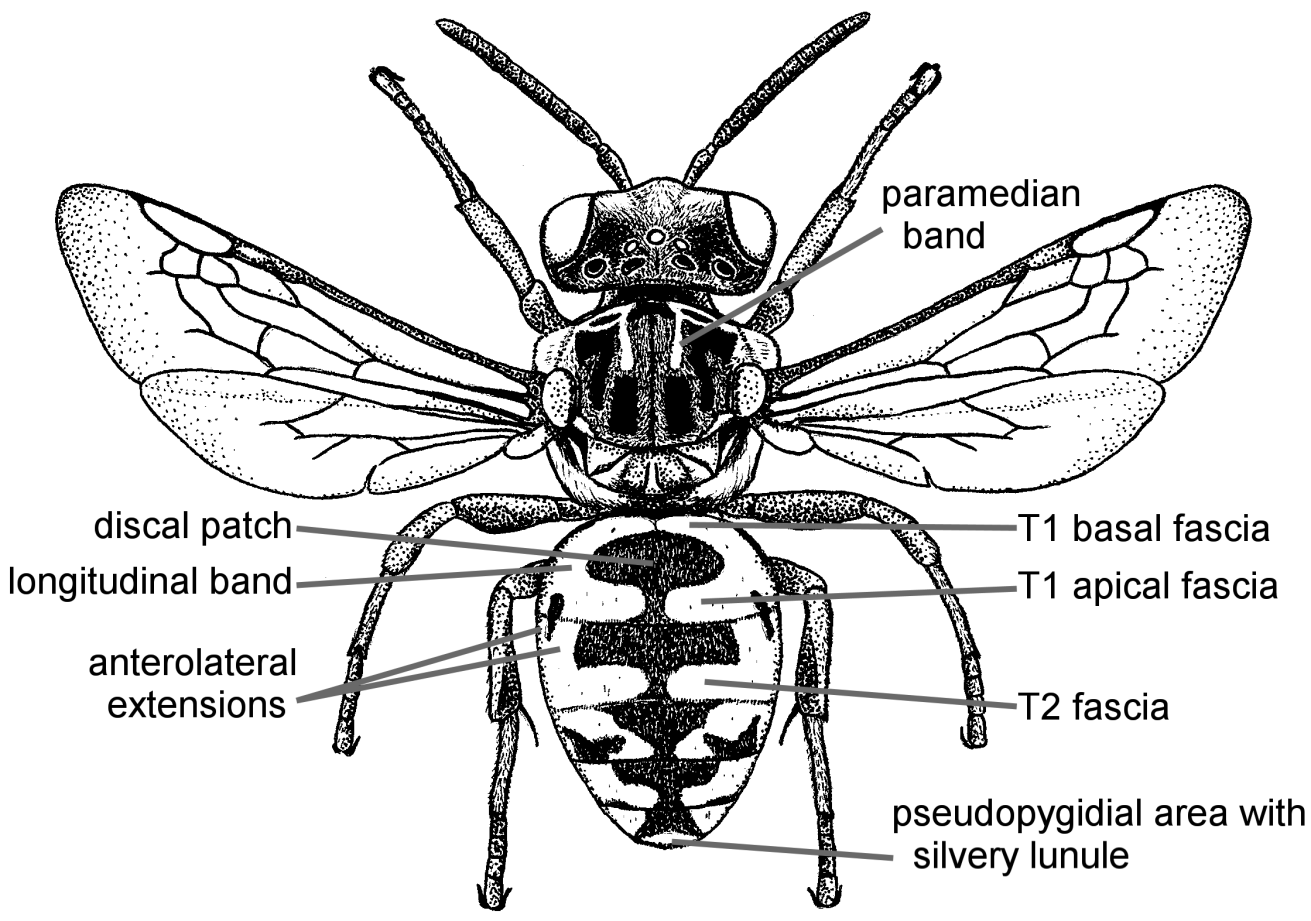
Female *E. chamaesarachae* sp. n. illustrating mesosomal and metasomal bands of tomentum commonly present in North American Epeolini.

Terminology used herein is consistent with that used in the recent treatment of Canadian *Epeolus* (Onuferko 2017), which generally followed Michener (2007), except the terms frontal area and vertexal area are used instead of frons and vertex, respectively. Acronyms used herein (in bold) are as follows. Puncture density is described in terms of interspaces (**i**) relative to the diameters (**d**) of punctures. Median ocellar diameter (**MOD**) is a comparative unit of measurement for smaller structures. **F** followed by a number represents one of 10 (for female) or 11 (for male) flagellomeres of the antenna. **T** followed by a number represents one of six (for female) or seven (for male) exposed metasomal terga. **S** followed by a number represents one of six (for female) or eight (for male) metasomal sterna. Several terms used in Onuferko (2017), some of which were taken from Rightmyer (2008), are defined here again for clarity, and are indicated in bold. **Length** refers to measurements made along the longitudinal axis of the bee, except in reference to the longitudinal extent of the transverse metasomal fasciae, for which the term **breadth** is used, and **width** refers to measurements made along the lateral axis. The length and width of an anatomical feature refer to its longest and widest margins, respectively, and were recorded at the highest magnification that allowed measurement in ocular micrometer units. The scape was measured without the radicle. In *Epeolus*, the frontal line extends into the supraclypeal area as a pronounced carina on a convex surface, referred to herein as the **frontal keel**. **Paramedian bands** are the paired lines of off-white or yellow tomentum located anteriorly on the mesoscutum of most *Epeolus* species (Fig. 1). The term **bigibbous** is an adjective used in reference to the biconvexities present on the mesoscutellum of *Epeolus* species. The basal and apical fasciae of T1 are often connected by a **longitudinal band** of pale tomentum of varying width. **Discal patch** refers to the discal region of T1 that is typically covered in dark tomentum and is bordered by bands of pale tomentum. This area is not always clearly delineated because the surrounding bands of pale tomentum may be reduced or missing entirely.

The species of *Epeolus* are, with the usual exceptions (differences in the number of antennal flagellomeres, number of exposed metasomal terga, length of the S4 and S5 subapical hairs [usually longer in males], and terminalia) and a few atypical ones, sexually monomorphic. For this reason, separate keys for females and males are not presented, and the few sex-specific features used to distinguish species are indicated as such in the couplets. The key to Nearctic *Epeolus* is heavily based on the structure of the axilla and the bands of pale tomentum forming the basal and apical fasciae on the metasomal terga. To limit the number of steps required to identify all species, efforts were made to make the key as close to fan shaped (evenly bifurcated) as possible, following the recommendations of Walter and Winterton (2007; see also Packer et al. 2016). When possible, couplets were based on more than a single feature (ideally one per tagma) should one be obscured or lost in the specimen being identified. However, avoiding monothetic couplets was not always possible. In such cases couplets were usually based on mesosomal features that should be visible even in damaged pinned specimens. In couplets that list multiple features, the most important (i.e., reliable) one for achieving a diagnosis is given first whereas features that do not always result in a positive identification (e.g., integument black vs integument black or ferruginous will resolve species with ferruginous but not black integument) are included but given at the end and always preceded by at least one feature that is fully contrasted between both halves of the couplet. The features referenced in the key were imaged. Quite often a single image or image plate was used to illustrate more than one feature, so a number of figures were cited two or more times within the key and elsewhere in the present monograph. As a result, it was not possible to put most illustrations near the couplets without duplicating them, and for practical reasons multiple versions of the same figures are not included herein. Many couplets rely on precise comparative measurements, and the key is meant to be used with the aid of an eyepiece graticule. None of the couplets require specimens to be dissected. Although the male S7, S8, and genital capsules of nearly all species were examined (except those represented by very few male specimens), the variation among them is minimal (illustrated in part in plates 2 and 3 in Onuferko 2017), and the terminalia have not proven useful in separating similar-looking species. Consequently, they have not been illustrated or imaged. The illustrations presented to aid in the identification of *Epeolus* species are my own. Images were taken with a digital camera (Canon EOS 40D SLR) using the Visionary Digital macro-imaging BK PLUS Lab System, focus stacked in Helicon Focus, and edited in Adobe Photoshop and PaintShop Pro.

Species descriptions follow the format of Onuferko (2017). A full description of the primary type specimen of each species is provided, except for the species occurring in Canada that were recently redescribed in Onuferko (2017). The physical name-bearing type specimens of all described North American *Epeolus* were seen and thoroughly examined, including those whose names are no longer considered valid, except in the case of *E. mercatus* Fabricius, for which the original type material cannot be traced and description is so insufficiently detailed that it is unclear if the species is an *Epeolus* or *Triepeolus* (Rightmyer 2008). Since most *Epeolus* species to date were described from female specimens, new species described herein are generally represented by a female holotype, male allotype, and paratypes. Given that *Epeolus* is a genus of largely sexually monomorphic species, descriptions of the sex opposite that of the name-bearing type list only key differences to avoid unnecessary duplication of text. In many, but not all, cases it is the female that is fully described. I have opted to propose new names for the species Brumley (1965) discovered rather than validate the ones he used. This will ensure that it is clear who made designations of type specimens (i.e., specimens used as types by Brumley (1965) and me have both our type labels, those unavailable to me but designated as types by Brumley (1965) have only his labels, and those seen exclusively by me and given type status have only my labels). This will also eliminate any possible confusion that could arise if Brumley’s (1965) names are published and registered in ZooBank long after their first appearance in his thesis.

The proposed common name for each species reflects its scientific name, which in most cases was easy to translate into English. Since there are many genera of cuckoo bees, epeolus is used herein as the common name for the genus instead of cuckoo bee or more specific but cumbersome names like Colletes cuckoo bee or polyester bee cuckoo bee.

Among the material examined were representatives of *Epeolus* from all Canadian provinces and territories except Newfoundland and Labrador and Nunavut, and all but six (Connecticut, Delaware, Kentucky, Rhode Island, Tennessee, and West Virginia) of the 49 states in the continental U.S. where the genus is expected to occur. Also examined were *Epeolus* records from 17 states in Mexico, and their data are included for species confirmed as occurring north of the Mexico–United States border. All examined records are presented in Suppl. material 1. Specimens were made available for study by curators and collections managers (in parentheses) from the following institutions:

**ABS** Archbold Biological Station, Venus, FL (M. Deyrup);

**AMNH** American Museum of Natural History, New York, NY (J.G. Rozen, Jr. and C. Smith);

**ANSP** Academy of Natural Sciences of Drexel University, Philadelphia, PA (J. Weintraub);

**AUMNH** Auburn University Museum of Natural History, Auburn, AL (C.H. Ray);

**BBSL** Utah State University USDA Bee Biology and Systematics Laboratory, Logan, UT (T.L. Griswold);

**BIML** Patuxent Wildlife Research Center USGS Native Bee Inventory and Monitoring Lab, Laurel, MD (S. Droege);

**CAS** California Academy of Sciences, San Francisco, CA (B. Fisher and R. Zuparko);

**CNC** Canadian National Collection of Insects, Arachnids and Nematodes, Ottawa, ON (S. Cardinal);

**CTMI** Central Texas Melittological Institute, Austin, TX (J.L. Neff);

**CUIC** Cornell University Insect Collection, Ithaca, NY (J. Dombroskie);

**CUM** University of Colorado Museum of Natural History, Boulder, CO (V. Scott);

**DEBU** University of Guelph Insect Collection, Guelph, ON (S.A. Marshall);

**EMEC** University of California Essig Museum of Entomology, Berkeley, CA (P. Oboyski);

**FMNH** Field Museum of Natural History, Chicago, IL (C. Maier);

**FSCA** Florida State Collection of Arthropods, Gainesville, FL (K. Schnepp and P.E. Skelley);

**INHS** Illinois Natural History Survey, Champaign, IL (C. Grinter);

**JBWM** University of Manitoba J.B. Wallis / R.E. Roughley Museum of Entomology, Winnipeg, MB (J. Gibbs);

**KUNHM** University of Kansas Biodiversity Institute and Natural History Museum, Lawrence, KS (M.S. Engel and J. Thomas);

**LACM** Natural History Museum of Los Angeles County, Los Angeles, CA (B.V. Brown and G.A. Kung);

**MCZ** Harvard University Museum of Comparative Zoology, Cambridge, MA (P.D. Perkins);

**NCSU** North Carolina State University Insect Museum, Raleigh, NC (R. Blinn);

**NHMUK** Natural History Museum, London, United Kingdom (D. Notton);

**PCYU** Packer Collection at York University, Toronto, ON (L. Packer);

**ROM** Royal Ontario Museum, Toronto, ON (A. Guidotti);

**RSKM** Royal Saskatchewan Museum, Regina, SK (C. Sheffield);

**UCBME** University of California Bohart Museum ​of Entomology, Davis, CA (S. Heydon and T.J. Zavortink);

**UCR** University of California Entomology Research Museum, Riverside, CA (D. Yanega); and

**USNM** U.S. National Entomological Collection, National Museum of Natural History, Washington, D.C. (S.G. Brady and B. Harris).

In lists of examined specimens, semi-colons separate records from different localities. Otherwise, commas are used between records from the same locality that are associated with a different collection date, collector(s), and/or repository. In such cases, the locality is not repeated and a comma appears after the specimen repository and before the collection date of the next record. If only the collection day and month were given, then “????” was used for the missing year. If the collection year was given to two digits but the century or millennium could not be inferred (e.g., from knowing who the collector was and the period in which he/she would have conducted field work), the two-digit year is still indicated but with “??” in front. All GPS coordinates indicated herein are taken directly from specimen labels. For approximate coordinates obtained post hoc for specimens with imprecise locality records used to construct range maps, see Suppl. material 1. For species reported from Canada, only total numbers of females and males from each province or state are shown for examined non-type specimens if the same records have already been published (Onuferko 2017).

Range maps were constructed as in Onuferko (2017) in RStudio (version 1.0.44) using the packages maptools (Bivand and Lewin-Koh 2014), raster (Hijmans 2014), rgdal (Bivand et al. 2014), and rgeos (Bivand and Rundel 2014) installed in R (version 3.3.2) (R Core Team 2016). The shapefiles used to plot projected maps of Canada, Mexico, and the USA were obtained from Statistics Canada (2015), DIVA-GIS (http://www.diva-gis.org/gdata), and the US Census Bureau (2015), respectively.

Floral associations are given for each species based on photo records, observations, and specimen labels. Records published in Onuferko (2017) are not repeated here, but they are included in Suppl. material 1. All floral records were checked against The Plant List (http://www.theplantlist.org/) to ensure that the scientific nomenclature is up to date.

## Taxonomy

### Epeolus

Taxon classificationAnimaliaHymenopteraApidae

Latreille, 1802

Epeolus Latreille, 1802: 427. Type species: Apis variegata Linnaeus, 1758, by monotypy.
Trophocleptria Holmberg, 1886: 233, 275. Type species: Trophocleptria variolosa Holmberg, 1886, by monotypy.
Epeolus (Diepeolus) Gribodo, 1894: 80. Type species: Epeolus giannellii Gribodo, 1894, by monotypy.
Epeolus (Monoepeolus) Gribodo, 1894: 80. Type species: Apis variegata Linnaeus, by monotypy.
Pyrrhomelecta Ashmead, 1899: 66. Type species: Epeolus glabratus Cresson, 1878, by original designation and monotypy.
Argyroselenis Robertson, 1903: 284. Type species: Triepeolus minimus Robertson, 1902, by original designation and monotypy.
Oxybiastes Mavromoustakis, 1954: 260. Type species: Oxybiastes bischoffi Mavromoustakis, 1954, by original designation and monotypy.

#### Remarks.

In his original description, Latreille (1802) did not explain the etymology of *Epeolus*, but it seems likely that the name is a diminutive of Epeus/Epeius, the soldier in Greek mythology to whom building the Trojan Horse is attributed, and that it was inspired by the sinister nature of these cleptoparasitic bees. This was the first genus of Epeolini described, and ‘epeolus’ has since become the root in the names of many other nomadine and non-nomadine genera and tribes (e.g., *Epeoloides* Giraud (Osirini), *Parepeolus* Ducke (Osirini), Protepeolini, *Pseudepeolus* Holmberg (Epeolini), etc.).

Several species of *Epeolus* were previously described as belonging to different genera, in particular *Triepeolus*. On account of Rightmyer’s (2008) revision of *Triepeolus*, the generic placement of species that were once erroneously switched has been corrected. A few North American species were (initially or at some point in the past) described as belonging to genera that are no longer considered valid, including *Argyroselenis* Robertson, *Phileremus* (the name is a synonym of Ammobates Latreille subgenus Ammobates Latreille s. str. in Michener 2007), and *Pyrrhomelecta* Ashmead. These represented unnatural groupings of species by shared homoplasious morphological features: if the fore wing has two submarginal cells (*Phileremus*) instead of the usual three, if the maxillary palpus is three-segmented (*Argyroselenis*) rather than two-segmented (both states occur within *Epeolus* and Thalestriina, Rightmyer 2004), and if there is extensive red versus black integument coloration and reduced pubescence (*Pyrrhomelecta*).

Species of *Epeolus* are small to moderate-sized (body length 5.5–10.0 mm) relatively robust cleptoparasitic (epeoliform) bees. In North America, *Epeolus* may be confused with *Triepeolus*, which it resembles in general appearance, although *Triepeolus* may attain a much larger size (body length up to 18 mm in some species, Rightmyer 2008). The only other North American epeoline genus, *Odyneropsis* Schrottky, is rare (known only from the American Southwest) (Griswold and Parker 1999) and more likely to be confused with vespid wasps (hence the root ‘odynerus’) rather than *Epeolus*. Comprehensive overviews of the distinguishing features of *Epeolus* in reference to all other Epeolini are provided in Rightmyer (2004) and Michener (2007).

#### Diagnosis for *Epeolus* in North America

**(Canada and the United States).** Diagnostic for female *Epeolus* is a very distinct S6, which is usually retracted except sometimes for a pair of convergent spatulate lateral apical processes bearing setae modified into minute, pointed denticles (Onuferko 2017, Fig. 2A, B). Basally, the processes are separated by a large lobe-like disc, which in *Triepeolus* is reduced to a narrow transverse bar. In both *Triepeolus* (Onuferko 2017, Fig. 2C, D) and *Odyneropsis*, the lateral apical processes are subparallel and bear coarse, spine-like setae. Additionally, females may be separated on the basis of the pseudopygidial area (the apicomedial region of T5 that changes slope from the rest of the tergum), which in *Epeolus* is covered in a silvery band of short apically rounded setae. In *Triepeolus*, the pseudopygidial area is usually longer than in *Epeolus* and in most species the setae reflect a golden color. The T5 in female *Odyneropsis* is unique in that it is broadly notched posteriorly and has a distinct middorsal depressed area in the shape of a pointed oval outlined by ridges (Rightmyer 2004, fig. 180A).

**Figure 2.**
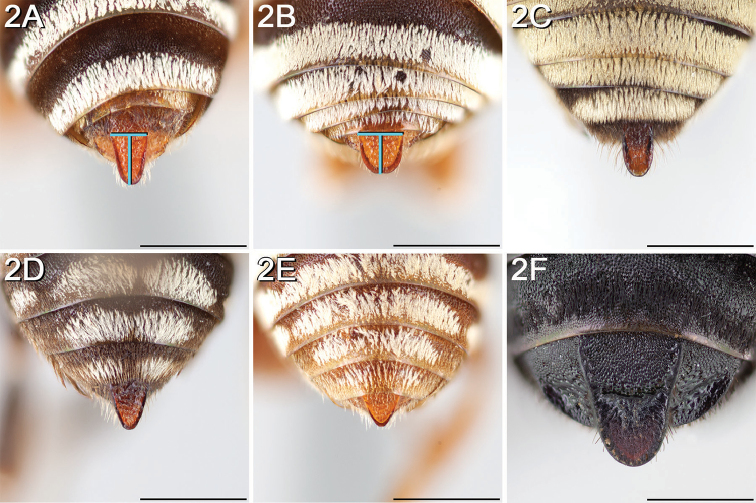
Pygidial plate (in dorsal view) of male **A** *E. australis* (longer than wide and apically narrowed) **B** *E. brumleyi* paratype (nearly as long as wide and apically rounded) **C** *E. flavofasciatus* (longer than wide, with the lateral margins parallel) **D** *E. asperatus* (longer than wide and apically narrowed) **E** *E. barberiellus* (somewhat longer than wide and apically narrowed), and **F** *T. concavus* (longer than wide, with the lateral margins somewhat concave). Scale bars 1 mm.

**Figure 3.**
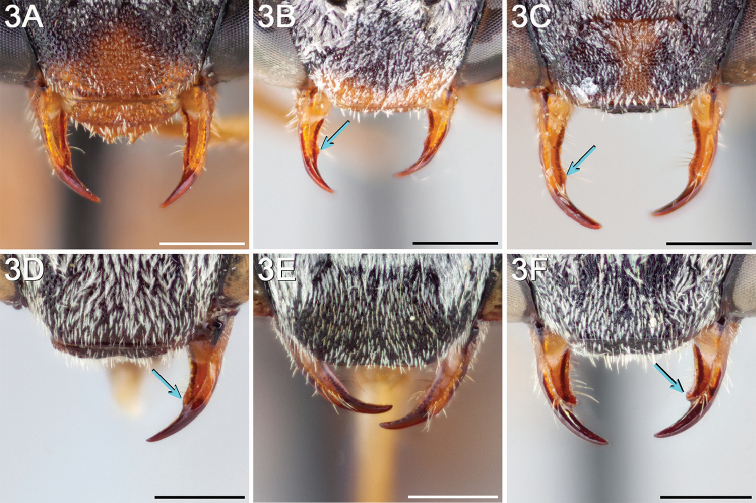
Mandible (in frontal view) of female **A** *E. ainsliei* without a preapical angulation or tooth **B** *E. attenboroughi* holotype with an inconspicuous, obtuse preapical tooth **C** *E. carolinus* with an inconspicuous, obtuse preapical tooth **D** *E. gibbsi* paratype with an obtuse angle appearing like a tooth **E** *E. vernalis* holotype (herein synonymized under *E. ilicis*) without a preapical angulation or tooth, and **F** *E. compactus* with a distinct preapical tooth with sides forming a right triangle. Scale bars 0.5 mm.

Male *Epeolus* are more difficult to diagnose. As in females, the body lacks integumental white or yellow areas but the mesosoma and usually other tagmata have short appressed plumose white and/or yellow setae; the maxillary palpus is two or three segmented; the inner margins of the compound eyes are distinctly convergent below; the axilla is produced to a rounded lobe or angle or spine (i.e., not continuing the contour of the mesoscutellum); the distitarsi of all legs have arolia; the fore wing usually has three submarginal cells (if with two, then the second is at least nearly as long as the first), and the marginal cell is apically removed from the wing margin and much longer than the stigma; and a pygidial plate is present. In male *Epeolus*, the pygidial plate in most species is broadly rounded posteriorly (Fig. 2B); in *Odyneropsis* and *Triepeolus* it is usually more elongate and with a median constriction (Fig. 2F). It should be noted that males of some species of *Epeolus* in North America (notably *E. australis* Mitchell, *E. flavofasciatus* Smith, and some males in the “*americanus* group”) have a very narrow and distinctly *Triepeolus*-like pygidial plate (Fig. 2A, C, D), as opposed to the more broadly rounded/subtruncate pygidial plate typically associated with male *Epeolus* (Fig. 2B). The presence of a preapical tooth of the mandible (Fig. 3B, C, D, F) (often hidden from view because the mandibles are usually closed) confirms these and other species as *Epeolus*; all *Triepeolus* and only some *Epeolus* (in North America *E. ainsliei*, *E. erigeronis*, *E. ilicis*, *E. inornatus*, and *E. zonatus*) lack one (Fig. 3A, E) (Rightmyer 2004).

#### List of species with their proposed common names

*Epeolus ainsliei* Crawford, 1932 – Ainslie’s epeolus

*Epeolus americanus* (Cresson, 1878) – American epeolus

*Epeolus andriyi* Onuferko, sp. n. – Andrew’s epeolus

*Epeolus asperatus* Cockerell, 1909 – rough epeolus

*Epeolus attenboroughi* Onuferko, sp. n. – Attenborough’s epeolus

*Epeolus australis* Mitchell, 1962 – southern epeolus

*Epeolus autumnalis* Robertson, 1902 – fall epeolus

*Epeolus axillaris* Onuferko, sp. n. – spiny epeolus

*Epeolus banksi* (Cockerell, 1907) – Banks’ epeolus

*Epeolus barberiellus* Cockerell, 1907 – Barber’s epeolus

*Epeolus basili* Onuferko, sp. n. – Basil’s epeolus

*Epeolus bifasciatus* Cresson, 1864 – two-banded epeolus

*Epeolus brumleyi* Onuferko, sp. n. – Brumley’s epeolus

*Epeolus canadensis* Mitchell, 1962 – Canada epeolus

*Epeolus carolinus* Mitchell, 1962 – Carolina epeolus

*Epeolus chamaesarachae* Onuferko, sp. n. – five eyes crowned epeolus

*Epeolus compactus* Cresson, 1878 – compact epeolus

*Epeolus deyrupi* Onuferko, sp. n. – Deyrup’s epeolus

*Epeolus diadematus* Onuferko, sp. n. – Texas crowned epeolus

*Epeolus erigeronis* Mitchell, 1962 – fleabane epeolus

*Epeolus ferrarii* Onuferko, sp. n. – Ferrari’s epeolus

*Epeolus flavofasciatus* Smith, 1879 – yellow-banded epeolus

*Epeolus floridensis* Mitchell, 1962 – Florida epeolus

*Epeolus gibbsi* Onuferko, sp. n. – Gibbs’ epeolus

*Epeolus glabratus* Cresson, 1878 – smooth epeolus

*Epeolus howardi* Mitchell, 1962 – Howard’s epeolus

*Epeolus ilicis* Mitchell, 1962 – holly epeolus

*Epeolus inornatus* Onuferko, sp. n. – inornate epeolus

*Epeolus interruptus* Robertson, 1900 – interrupted epeolus

*Epeolus lectoides* Robertson, 1901 – Eastern prized epeolus

*Epeolus lectus* Cresson, 1878 – Great Plains prized epeolus

*Epeolus mesillae* (Cockerell, 1895) – Mesilla epeolus

*Epeolus minimus* (Robertson, 1902) – least epeolus

*Epeolus nebulosus* Onuferko, sp. n. – clouded epeolus

*Epeolus novomexicanus* Cockerell, 1912 – New Mexico epeolus

*Epeolus olympiellus* Cockerell, 1904 – Olympia epeolus

*Epeolus packeri* Onuferko, sp. n. – Packer’s epeolus

*Epeolus pusillus* Cresson, 1864 – dwarf epeolus

*Epeolus rufulus* Cockerell, 1941 – reddish epeolus

*Epeolus scutellaris* Say, 1824 – shield-backed epeolus

*Epeolus splendidus* Onuferko, sp. n. – polished epeolus

*Epeolus tessieris* Onuferko, sp. n. – Tessier’s epeolus

*Epeolus zonatus* Smith, 1854 – white-banded red epeolus

### 1. Epeolus ainsliei

Taxon classificationAnimaliaHymenopteraApidae

Crawford, 1932

**Figure 4.**
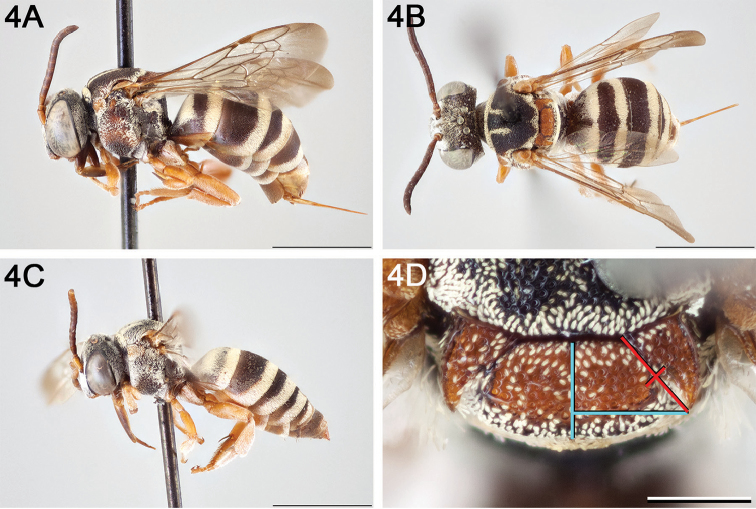
*Epeolus ainsliei* **A** female, lateral habitus (scale bar 3 mm) **B** female holotype, dorsal habitus (scale bar 3 mm) **C** male, lateral habitus (scale bar 3 mm), and **D** female axillae and mesoscutellum, dorsal view (scale bar 0.5 mm; blue lines indicate the posterior extent of the axilla relative to the length of the mesoscutellum; red lines indicate the extent of the free portion of the axilla relative to its entire medial length).

**Figure 5.**
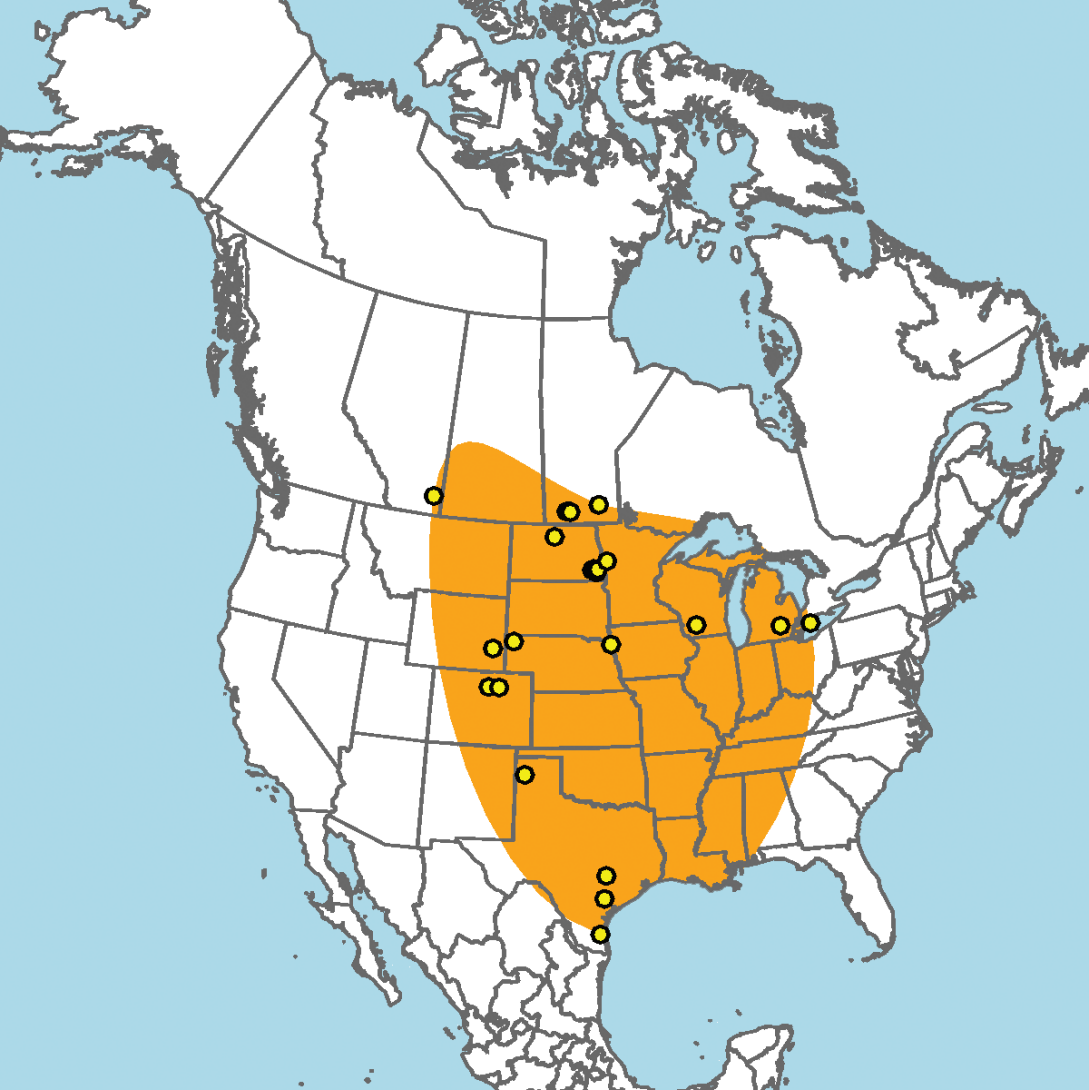
Approximate geographic range of *E. ainsliei* (orange) based on occurrence records known to the author (yellow circles).

Figs 3A , 4 , 5 , 95A

Epeolus ainsliei Crawford, 1932. Proc. Entomol. Soc. Wash. 34: 74 (♀).

#### Diagnosis.

The following morphological features in combination can be used to tell *E. ainsliei* apart from all other North American *Epeolus*: the mandible lacks a preapical angle or tooth and the preoccipital ridge joins the hypostomal carina. In some specimens of *E. scutellaris*, the preoccipital ridge joins or nearly joins the hypostomal carina, in which case it is separated from the hypostomal carina by less than 1 MOD at its terminal, but the species has a blunt, obtuse preapical tooth on the mandible and the axillae are relatively straight along the medial margin whereas in *E. ainsliei* the free portion is distinctly hooked. *Epeolus ainsliei* is also very similar to *E. attenboroughi* and *E. rufulus*, which it resembles in that in all three species the axilla is dilated laterally and the free portion is distinctly hooked, and the T1–T4 apical fasciae are complete; however, in both *E. attenboroughi* and *E. rufulus* the mandible has a blunt, obtuse preapical tooth, the mesoscutum lacks the distinct paramedian bands present in *E. ainsliei* and is instead largely obscured by pale tomentum, and the preoccipital ridge does not join the hypostomal carina.

#### Redescription.

This species was recently redescribed (Onuferko 2017).

#### Distribution.

Great Plains to southwestern Ontario (Fig. 5).

#### Ecology.

HOST RECORDS: *Epeolus ainsliei* has been collected with possible host species *Colletes susannae* Swenk in Birds Hill Provincial Park (Gibbs et al. 2017) and Spruce Woods Provincial Park (J. Gibbs, personal communication, 2017), Manitoba, Canada and Spring Green Preserve in Sauk County, Wisconsin, USA (Wolf and Ascher 2009). In all cases at least one other species of *Colletes* was observed at the same locality and time as *C. susannae* and *E. ainsliei*, but observations of other *Colletes* were limited to one or two localities.

FLORAL RECORDS: Labels of examined voucher specimens indicate floral associations with *Dalea purpurea* Vent. (Leguminosae) and *D. villosa* (Nutt.) Spreng.

#### Discussion.

Detailed morphological and taxonomic remarks about this species are given in Onuferko (2017).

#### Material studied.

**Type material.** Primary: USA: **Iowa**: Sioux City, 15.vii.1922, C.N. Ainslie (holotype ♀ [USNM, catalog number: 534035]).

#### DNA barcoded material with BIN-compliant sequences.

Available. BOLD:ACZ1957. Specimens examined and sequenced. Canada: **Manitoba**: 1♀ (PCYU); Birds Hill Provincial Park (50.0190°N; 96.8820°W) (Division 12), 05.viii.2017, J. Gibbs and Nozoe (1♀, JBWM); **Ontario**: Rondeau Provincial Park (42.2814°N; 81.8427°W) (Beach Access #10, near Visitor Centre), 08.viii.2017, R. Ferrari (1♂, PCYU).

#### Non-barcoded material examined.

Canada: **Alberta**: 10♀, 1♂ (BBSL, CNC); **Manitoba**: Yellow Quill Mixed Grass Prairie Preserve (49.6911°N; 99.5747°W) (near Treesbank), 17.vii.2006, A.M. Patenaude (1♀, JBWM); Bald Head Hills (Spruce Woods Provincial Park), 01.viii.1983, W.E. Ralley (1♀, JBWM); Birds Hill Provincial Park (50.0100°N; 96.9100°W) (Division 12), 15.vii.2017, J. Gibbs and Nozoe (1♂, JBWM); Birds Hill Provincial Park (50.0115°N; 96.9065°W) (Division 12), 05.viii.2017, J. Gibbs and Nozoe (2♀, JBWM).

USA: **Colorado**: Longmont (Boulder County), 21.vii.1936, R. Bauer (1♂, CUM); Roggen, 08.vii.1933, M. and H. James and L. Ireland (1♂, CUM); **Iowa**: 1♀ (AMNH); **Michigan**: Edwin S. George Reserve (Livingston County), 12.viii.1960, U.N. Lanham (1♀, CUM); **Minnesota**: 1♀ (EMEC); **Nebraska**: 1♀ (AMNH); **North Dakota**: 7♀, 3♂ (AMNH, EMEC); **Texas**: 3♀, 2♂ (AMNH, CAS, CTMI); **Wyoming**: 1♀ (USNM).

### 2. Epeolus americanus

Taxon classificationAnimaliaHymenopteraApidae

(Cresson, 1878)

Figs 6 , 7 , 92K

Phileremus americanus Cresson, 1878. Trans. Am. Entomol. Soc. 7: 83 (♀, ♂); Cresson, 1916. Mem. Am. Entomol. Soc. 1: 111 (♀) [lectotype designation].
Phileremus montanus Cresson, 1878. Trans. Am. Entomol. Soc. 7: 83 (♂).
Epeolus lanhami Mitchell, 1962. N. C. Agric. Exp. Stn. Tech. Bull. 152: 450 (♀).

#### Diagnosis.

The following morphological features in combination (excluding any that are specific to the opposite sex of the one being diagnosed) can be used to tell *E. americanus* apart from all other North American *Epeolus* except *E. asperatus* and *E. barberiellus*: in females, F2 is not more than 1.1 × as long as wide; the mesoscutum has distinct paramedian bands; the axilla is small to intermediate in size, not extending beyond the midlength of the mesoscutellum and the free portion is less than 1/4 as long as the entire medial length of the axilla, and like the mesoscutellum black; the mesopleuron is closely (i≤1d) and evenly punctate; T1 has a quadrangular discal patch, in dorsal view the longitudinal band is at least as wide as the breadth of the apical fascia; and the T1 and T2 apical fasciae are interrupted or at least greatly narrowed medially. Whereas in *E. barberiellus* the pronotal lobe and legs, at least from the tibiae to tarsi (sometimes the trochanters and femora as well), are reddish orange, in *E. americanus* the pronotal lobe and legs are brown or black. *Epeolus americanus* is also very similar to *E. asperatus*, but in *E. asperatus* the mesopleuron has much denser punctures ventrolaterally (most i<1d) than that of *E. americanus* and the T3 and T4 fasciae are never complete but broken or at least greatly narrowed laterally, as well as medially into separated or narrowly connected oval patches.

**Figure 6.**
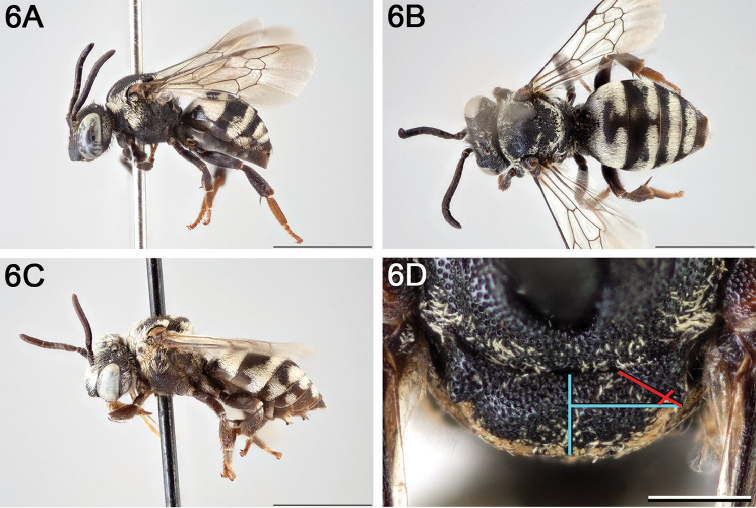
*Epeolus americanus* **A** female, lateral habitus (scale bar 3 mm) **B** female, dorsal habitus (scale bar 3 mm) **C** male, lateral habitus (scale bar 3 mm), and **D** female axillae and mesoscutellum, dorsal view (scale bar 0.5 mm; blue lines indicate the posterior extent of the axilla relative to the length of the mesoscutellum; red lines indicate the extent of the free portion of the axilla relative to its entire medial length).

#### Redescription.

This species was recently redescribed (Onuferko 2017).

#### Distribution.

**Figure 7.**
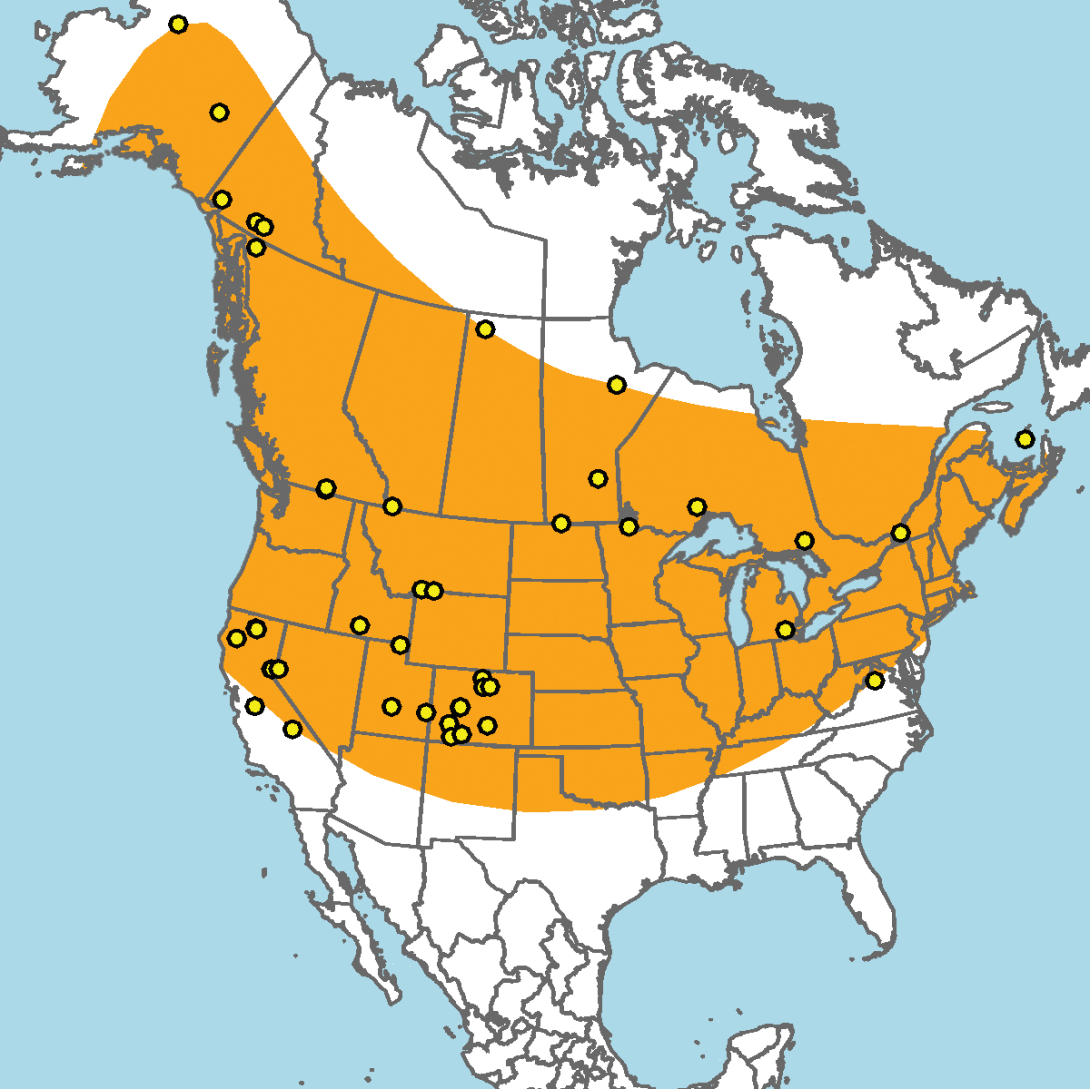
Approximate geographic range of *E. americanus* (orange) based on occurrence records known to the author (yellow circles).

Widely distributed across Canada and the United States, including Alaska; not known to occur in parts of northeastern North America, the southeastern United States, or the high arctic (Fig. 7).

#### Ecology.

See Onuferko (2017) for host and floral records. Floral associations are also indicated in Suppl. material 1, which includes newly discovered associations with *Leucanthemum vulgare* (Vaill.) Lam. (Compositae), *Plagiobothrys* Fisch. & C.A. Mey. (Boraginaceae), *Salix exigua* Nutt. (Salicaceae), and *S. interior* Rowlee based on labels of examined voucher specimens.

#### Discussion.

Detailed morphological and taxonomic remarks about this species are given in Onuferko (2017).

#### Material studied.

**Type material.** Primary: USA: **Colorado**: H.K. Morrison (*P. americanus* lectotype ♀ [ANSP, catalog number: 2235]); **Michigan**: Near Saline, 26.vi.1954, U.N. Lanham (*E. lanhami* holotype ♀ [CUM, catalog number: 0000041]); **Nevada**: H. Edwards (*P. montanus* holotype ♂ [ANSP, catalog number: 2231]).

Secondary: USA: **Michigan**: Near Saline, 26.vi.1954, U.N. Lanham (*E. lanhami* allotype ♂ [CUM, catalog number: 0000042]).

#### DNA barcoded material with BIN-compliant sequences.

Available. BOLD:AAB9110. Specimens examined and sequenced. Canada: **Quebec**: 1♂ (RSKM); **Yukon**: 12♀, 2♂ (PCYU).

USA: **Colorado**: 2♀ (PCYU); **Utah**: 1♀ (BBSL).

#### Non-barcoded material examined.

Canada: **Alberta**: 1♂ (CNC); **British Columbia**: 1♀, 2♂ (CNC); **Manitoba**: 1♀ (CNC); Adam Lake (Turtle Mountain Provincial Park), 27.vi.1987, T.D. Galloway (1♀, JBWM); Beaver Creek (Lake Winnipeg), 21.vi.1962, J.A. Garland (1♀, JBWM); **Ontario**: 6♀, 2♂ (CAS, CNC); **Quebec**: 1♀ (USNM); **Saskatchewan**: 2♀ (CNC); **Yukon**: 5♀, 1♂ (PCYU, RSKM).

USA: **Alaska**: 2♀, 3♂ (CNC); **California**: 1♂ (PCYU); 2 mi S Hilmar (Merced County), 14.iv.1961, R.R. Snelling (1♂, LACM); 3 mi SW Ash Creek (Siskiyou County), 16.vi.1974, D. Green (1♀, EMEC); Ash Creek Ranger Station (9 mi E McCloud, Siskiyou County), 07–09.vi.1974, J. Powell (1♂, EMEC), 10–12.vi.1974, R. Coville (4♀, 1♂, EMEC); Hayfork Ranger Station (Trinity County), 19.v.1973, J. Doyen (1♂, EMEC), 23.v.1973, J. Powell (1♀, EMEC); Independence Lake (Sierra County), 24.iv.1974, R.M. Bohart (1♂, UCBME); Lone Pine (Inyo County), 13.v.1969, J.A. Chemsak (1♀, EMEC); Sagehen Creek (Nevada County), 04.vii.??62, R.L. Westcott (1♀, LACM), 01.vii.??70, M.G. Axtman (1♂, LACM), 22.vi.1972, R.M. Bohart (1♀, 1♂, UCBME), 19.vi.1974, R.M. Bohart (4♀, UCBME), 23.vi.1976, N.J. Smith (1♀, UCBME), 23.vi.1976, R.M. Bohart (3♀, 2♂, UCBME), 23.vi.1976, R.M. Giblin (3♀, 1♂, UCBME), 23.vi.1976, R.E. Otondo (1♂, UCBME), 23.vi.1976, G.M. Streett (2♂, UCBME), 23.vi.1976, C.M. Bortfeid (1♂, UCBME), 30.vi.1976, N.J. Smith (1♀, UCBME), 14.vii.1976, R.M. Bohart (1♀, UCBME), 28.vi.1978, D.R. Smart (1♂, UCBME), 28.vi.1978, L.S. Kimsey (2♀, UCBME), 16.vii.1980, R.M. Bohart (1♀, UCBME); **Colorado**: 4♀ (PCYU); vi.1917 (1♀, AMNH); Cirque Meadows (Larimer County), 01.vii.1978, S. Hart (1♂, EMEC); Davenport Camp, 02.vii.1967, F., P., and M. Rindge (1♀, AMNH); Electra Lake, 28.vi.–01.vii.1919 (1♀, AMNH); Longmont (40.1507°N; 105.0385°W) (Weld County), 23.v.2012, V. Scott (1♂, CUM); Near Wolf Creek (37.4999°N; 106.7692°W) (Mineral County), 28.vii.2007, J. Gibbs and C. Sheffield (2♀, PCYU); Ouray (Summit road), 13.vii.1919 (1♂, AMNH); **Idaho**: 1♂ (USNM); **Nevada**: Reno, v.1940, U.N. Lar (1♀, CUM); **Utah**: 2♀ (PCYU); **Virginia**: 1♀ (USNM); **Wyoming**: 13 mi SE Cooke City, 27.vii.1962, F., P., and M. Rindge (1♀, AMNH); Yellowstone River (between Knowles Falls and Gardiner, Yellowstone National Park), 24.vi.1979, R.E. Dietz (1♂, EMEC).

### 3. Epeolus andriyi sp. n.

Taxon classificationAnimaliaHymenopteraApidae

http://zoobank.org/97D5B971-2314-4E0A-BCF1-0E938C0EDA25

Figs 8 , 9

#### Diagnosis.

The following morphological features in combination (excluding any that are specific to the opposite sex of the one being diagnosed) can be used to tell *E. andriyi* apart from all other North American *Epeolus*: the axilla is large, with the tip extending well beyond the midlength of the mesoscutellum but not as far back as its posterior margin, dilated laterally but relatively straight along the medial margin, and like the mesoscutellum ferruginous; the axilla’s free portion is clearly less than 2/5 as long as its entire medial length; the mesopleuron is closely (i≤1d) and evenly punctate; the metasomal terga are black; T1 has a distinct basal fascia, which may be narrowly interrupted medially; the mesoscutum and metasomal terga have bands of bright or pale yellow short appressed setae; at least the T1–T3 apical fasciae are distinctly interrupted medially; and the pseudopygidial area of the female is lunate with the apex <2 × the medial length. *Epeolus andriyi* is most similar to *E. howardi*, but in *E. howardi* the axillae extend further posteriorly, as far back as or beyond the posterior margin of the mesoscutellum, and both the axillae and mesoscutellum are entirely red whereas in *E. andriyi* the mesoscutellum is dark brown or black along the anterior margin. *Epeolus andriyi* is also similar to *E. scutellaris*, but in *E. scutellaris* the T1–T3 apical fasciae are complete or only very narrowly interrupted medially, and the pseudopygidial area of the female is lunate with the apex >2 × the medial length.

#### Description.

FEMALE: Length 8.2 mm; head length 1.9 mm; head width 2.6 mm; fore wing length 5.5 mm (margins of both worn in holotype).

*Integument coloration.* Mostly black; notable exceptions as follows: partially to entirely ferruginous on mandible, antenna, pronotal lobe, tegula, axilla, mesoscutum, mesoscutellum, mesopleuron, and legs. Mandible with apex darker than all but extreme base; preapical tooth lighter than mandibular apex. Antenna brown except scape, pedicel, and F1 extensively orange. F2 with orange spot basally. Pronotal lobe and tegula pale ferruginous to amber. Mesoscutum with reddish-brown spot anterolaterally between pronotal lobe and tegula. Wing membrane dusky subhyaline, slightly darker at apex. Legs more extensively reddish orange than brown or black.

*Pubescence.* Face with tomentum densest around antennal socket. Clypeus, upper paraocular and frontal areas, and vertexal area mostly exposed. Dorsum of mesosoma and metasoma with bands of off-white to pale yellow short appressed setae. Mesoscutum with paramedian band. Mesopleuron with upper half hairy, except beneath base of fore wing (hypoepimeral area); ventrolateral half nearly bare. Metanotum with tomentum sparser medially, uniformly off white. T1 with discal patch quadrangular and very wide, the basal and apical fasciae only narrowly joined laterally by few sparsely scattered pale hairs. T1–T3 with apical fasciae interrupted medially and narrowed before becoming somewhat broader laterally; T2 with fascia without anterolateral extensions of tomentum, although few sparsely scattered pale hairs present. T4 with fascia narrowed medially. T5 with two patches of pale tomentum (both quite faint in holotype because much of pubescence discolored or rubbed off) lateral to and contacting pseudopygidial area. T5 with pseudopygidial area lunate, its apex less than twice as wide as medial length, indicated by silvery setae on impressed disc of apicomedial region elevated from rest of tergum. S5 with apical fimbria of coppery to silvery hairs not extending beyond apex of sternum by more than 1/4 MOD.

*Surface sculpture.* Punctures dense. Labrum with larger and sparser punctures (i=1–2d) than clypeus (i<1d). Small impunctate matte spot lateral to lateral ocellus. Mesoscutum, mesoscutellum, and axilla coarsely and densely rugose-punctate. Tegula densely punctate mesally (i≤1d), less so laterally (i=1–2d). Mesopleuron with ventrolateral half densely punctate (i≤1d), the interspaces shining; mesopleuron with punctures more or less equally dense throughout. Metasomal terga with punctures very fine, dense (i≈1d), evenly distributed on disc; the interspaces shining somewhat.

*Structure.* Preapical tooth inconspicuous, blunt and obtuse. Labrum with pair of small subapical denticles, each preceded by small discrete longitudinal ridge. Frontal keel not strongly raised. Scape with greatest length 1.8 × greatest width. F2 noticeably longer than wide (L/W ratio = 1.5). Preoccipital ridge not joining hypostomal carina, from which it is separated by no less than 1 MOD at its terminal. Mesoscutellum weakly bigibbous. Axilla large, its lateral margin (L) half as long as mesoscutellar width (W) (L/W ratio = 0.5) and tip extending well beyond midlength of mesoscutellum but not as far back as its posterior margin; axilla with tip clearly visible, but unattached to mesoscutellum for less than 2/5 the medial length of axilla; axilla with lateral margin arcuate. Fore wing with three submarginal cells. Pygidial plate apically truncate.

MALE: Description as for female except for usual secondary sexual characters and as follows: F2 shorter, not noticeably longer than wide (L/W ratio = 1.1); S4 and S5 with much longer coppery to silvery subapical hairs; pygidial plate apically rounded, with large deep punctures more or less evenly spaced throughout, with the interspaces shining.

**Figure 8.**
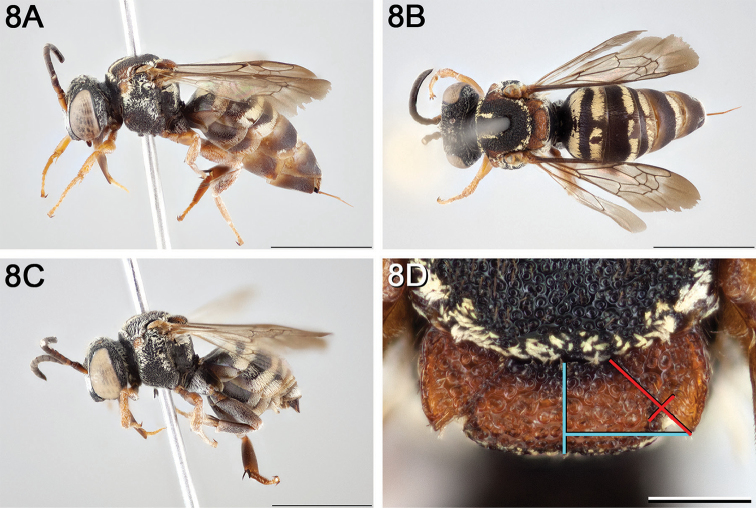
*Epeolus andriyi* **A** female holotype, lateral habitus (scale bar 3 mm) **B** female holotype, dorsal habitus (scale bar 3 mm) **C** male allotype, lateral habitus (scale bar 3 mm), and **D** female holotype axillae and mesoscutellum, dorsal view (scale bar 0.5 mm; blue lines indicate the posterior extent of the axilla relative to the length of the mesoscutellum; red lines indicate the extent of the free portion of the axilla relative to its entire medial length).

#### Etymology.

This species is named in honor of my father, Rev. Andriy Onuferko, in gratitude for encouraging my interests in the natural world and for his assistance in collecting *Epeolus* in the field.

#### Distribution.

**Figure 9.**
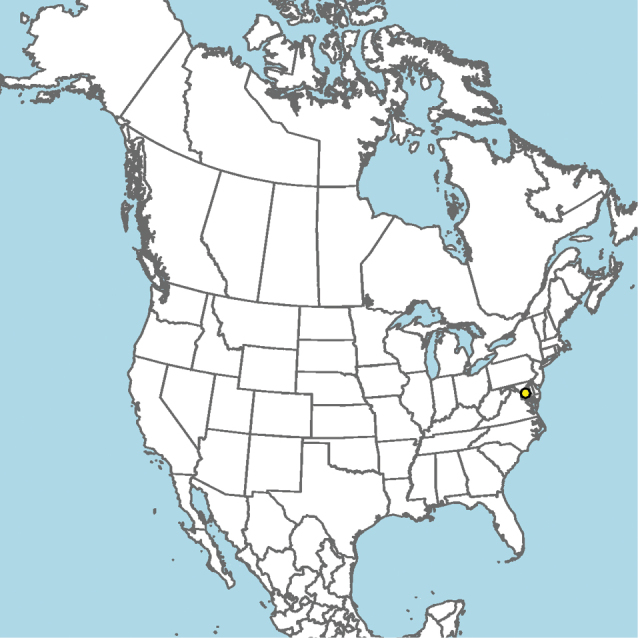
Occurrence record of *E. andriyi* known to the author (yellow circle).

Presently known from a single location along the Patuxent River in Maryland, USA (Fig. 9).

#### Ecology.

HOST RECORDS: The host species of *E. andriyi* is/are presently unknown.

FLORAL RECORDS: Unknown.

#### Discussion.

*Epeolus andriyi* and *E. howardi* are very similar to one another, and both species have been collected in Maryland, USA in late August. Although *E. andriyi* is known from only two specimens, in both the axillae are shorter than in any examined specimen of *E. howardi*. The status of *E. andriyi* as a separate species is further supported by a separate BIN, but unusually its nearest neighbor is *E. lectoides*, from which *E. andriyi* exhibits a large barcode sequence divergence (7.1%).

#### Material studied.

**Type material.** Primary: USA: **Maryland**: Jug Bay Wetlands Sanctuary (38.7839°N; 76.7014°W) (Anne Arundel County), 31.viii.2004, B. Hollister (♀ holotype [04-MD-1692], RSKM).

Secondary: USA: **Maryland**: Jug Bay Wetlands Sanctuary (38.7839°N; 76.7014°W) (Anne Arundel County), 31.viii.2004, B. Hollister (♂ allotype [04-MD-1691], RSKM).

#### DNA barcoded material with BIN-compliant sequences.

Available. BOLD:AAX7179. See Type material for specimens examined and sequenced (indicated by unique identifier number in square brackets).

### 4. Epeolus asperatus

Taxon classificationAnimaliaHymenopteraApidae

Cockerell, 1909

**Figure 10.**
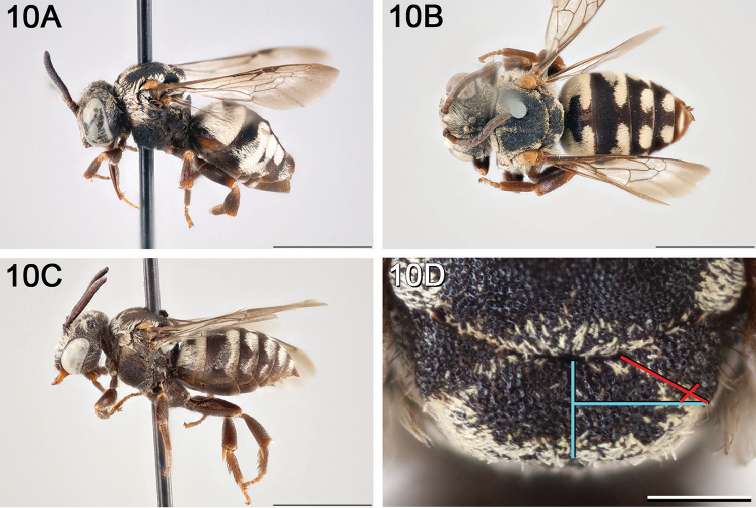
*Epeolus asperatus* **A** female, lateral habitus (scale bar 3 mm) **B** female holotype, dorsal habitus (scale bar 3 mm) **C** male, lateral habitus (scale bar 3 mm), and **D** female axillae and mesoscutellum, dorsal view (scale bar 0.5 mm; blue lines indicate the posterior extent of the axilla relative to the length of the mesoscutellum; red lines indicate the extent of the free portion of the axilla relative to its entire medial length).

**Figure 11.**
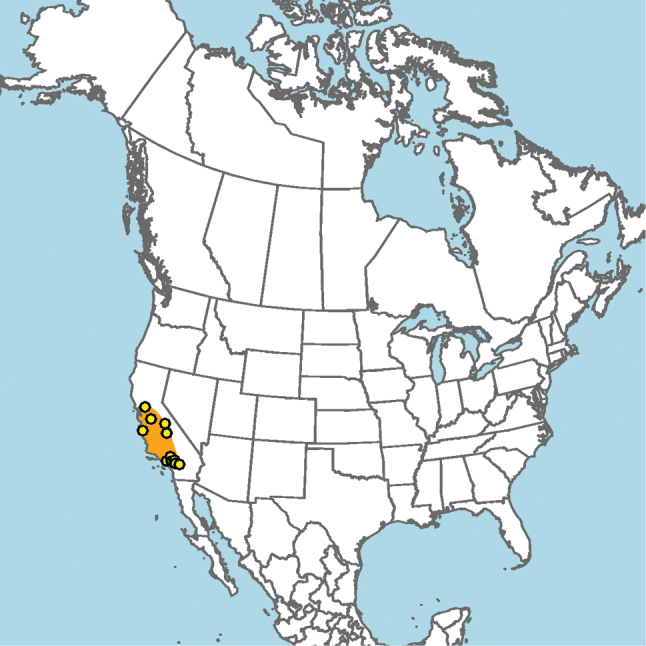
Approximate geographic range of *E. asperatus* (orange) based on occurrence records known to the author (yellow circles).

Figs 2D , 10 , 11 , 92L

Epeolus asperatus Cockerell, 1909. Ann. Mag. Nat. Hist. 5: 25 (♀).
Epeolus melectimimus Cockerell & Sandhouse, 1924. Proc. Calif. Acad. Sci. (4) 13: 317 (♂), **syn. n.**

#### Diagnosis.

The following morphological features in combination (excluding any that are specific to the opposite sex of the one being diagnosed) can be used to tell *E. asperatus* apart from all other North American *Epeolus* except *E. americanus* and *E. barberiellus*: in females, F2 is not more than 1.1 × as long as wide; the mesoscutum has distinct paramedian bands; the axilla is small to intermediate in size, not extending beyond the midlength of the mesoscutellum and the free portion is less than 1/4 as long as the entire medial length of the axilla, and like the mesoscutellum black; the mesopleuron is closely (most i<1d) and evenly punctate; T1 has a quadrangular discal patch, in dorsal view the longitudinal band is at least as wide as the breadth of the apical fascia; and the T1 and T2 apical fasciae are interrupted or at least greatly narrowed medially. Whereas in *E. barberiellus* the legs, at least from the tibiae to tarsi (sometimes the trochanters and femora as well), are reddish orange and the metasomal terga are fasciate, in *E. asperatus* the legs are brown or black and the T3 and T4 fasciae are broken or at least greatly narrowed laterally, as well as medially into separated or narrowly connected oval patches. *Epeolus asperatus* is most similar to *E. americanus*, but in *E. americanus* the mesopleuron has sparser punctures ventrolaterally (i≤1d) than that of *E. asperatus*, with the interspaces shining, and the T3 and T4 fasciae are complete or broken medially and/or laterally, but rarely into separated oval patches.

#### Redescription.

FEMALE: Length 7.8 mm; head length 2.0 mm; head width 2.8 mm; fore wing length 5.4 mm.

*Integument coloration.* Mostly black; notable exceptions as follows: at least partially ferruginous on mandible, labrum, antenna, pronotal lobe, tegula, and legs. Mandible with apex darker than rest of mandible; preapical tooth lighter than mandibular apex (difficult to see in the *E. asperatus* holotype; described from non-type specimens). Antenna brown except F1 and F2 orange in part. Flagellum slightly lighter than conspicuously dark brown scape and pedicel, primarily due to extensive pilosity on flagellum. Pronotal lobe and tegula pale ferruginous to amber. Wing membrane subhyaline, apically dusky. Legs with brown or black more extensive than reddish orange.

*Pubescence.* Face with tomentum densest around antennal socket. Dorsum of mesosoma and metasoma with bands of off-white to pale yellow short appressed setae. Mesoscutum with paramedian band. Mesopleuron with upper half hairy, ventrolateral half nearly bare. Metanotum with tomentum rubbed off medially in the *E. asperatus* holotype, but somewhat sparser medially and uniformly off white in non-type specimens. T1 with median quadrangular black discal patch enclosed by pale tomentum, except for medial separation at apex, and narrow, such that longitudinal band nearly half as wide as width of discal patch in dorsal view. T2–T4 with fasciae interrupted medially and with anterolateral extensions of sparser tomentum. T3 and T4 with fasciae also interrupted laterally, appearing as pair of oval patches between medial and lateral interruptions. T5 with two patches of pale tomentum lateral to and separate from pseudopygidial area (difficult to see in the *E. asperatus* holotype because T5 mostly retracted; described from non-type specimens). T5 with pseudopygidial area lunate, its apex more than twice as wide as medial length, indicated by silvery setae on impressed disc of apicomedial region elevated from rest of tergum. S5 with apical fimbria of coppery to silvery hairs not extending beyond apex of sternum by more than 1/4 MOD.

*Surface sculpture.* Punctures dense. Labrum with larger and sparser punctures (i=1–2d) than clypeus (i<1d). Small impunctate shiny spot lateral to lateral ocellus. Mesoscutum, mesoscutellum, and axilla coarsely and densely rugose-punctate. Tegula very densely punctate (i<1d). Mesopleuron with ventrolateral half densely punctate (i<1d); mesopleuron with punctures more or less equally dense throughout. Metasomal terga with punctures very fine, dense (i≈1d), evenly distributed on disc.

*Structure.* Preapical tooth with blunt tip. Labrum with pair of small subapical denticles, each preceded by small discrete longitudinal ridge. Frontal keel not strongly raised. Scape with greatest length 1.9 × greatest width. F2 as long as wide (L/W ratio = 1.0). Preoccipital ridge not joining hypostomal carina, from which it is separated by about 1.5–2 MOD at its terminal (difficult to see in the *E. asperatus* holotype; described from non-type specimens). Mesoscutellum moderately bigibbous. Axilla small to intermediate in size, its lateral margin (L) less than half as long as mesoscutellar width (W) (L/W ratio = 0.4) and tip not extending beyond midlength of mesoscutellum; axilla with tip visible, but unattached to mesoscutellum for less than 1/3 the medial length of axilla; axilla with lateral margin relatively straight and without carina. Fore wing with second submarginal crossvein incomplete in the *E. asperatus* holotype; with submarginal cells two or three and closed or second submarginal crossvein incomplete in non-type specimens. Pygidial plate apically truncate.

MALE: Description as for female except for usual secondary sexual characters and as follows: F2 shorter, nearly as long as wide (L/W ratio = 0.8); S4 and S5 with much longer coppery to silvery subapical hairs; pygidial plate V-shaped but apically rounded, with large deep, well-separated punctures, with the interspaces shining.

#### Distribution.

Central and southern California (Fig. 11).

#### Ecology.

HOST RECORDS: Nine representatives of this species were collected at the Robert J. Bernard Biological Field Station in Claremont, California, USA in the spring of 2016 (see Material studied), and the only *Colletes* collected or observed was a single female of a predominantly black species with pale pubescence limited to the mesosoma. The collected female of the possible host species was barcoded, and using Stephen’s (1954) key identified as *C. californicus* Provancher. However, its sequence clusters with sequences of specimens collected in New Mexico (also in the spring of 2016) and identified as *C. sphaeralceae* Timberlake (with entirely/predominantly pale pubescence) through the use of Stephen’s (1954) key, dissection of the male terminalia, and collection from red *Sphaeralcea* A. St.-Hil. (Malvaceae) flowers, and all were assigned the same BIN (BOLD:ABZ4529). Another predominantly black female specimen from the San Diego National Wildlife Refuge Otay-Sweetwater Unit in California was barcoded (its image and 601 bp sequence are available on the Barcode of Life Data Systems website [http://www.barcodinglife.org/]), and was assigned the same BIN as the female from Claremont and specimens from New Mexico.

FLORAL RECORDS: Labels of examined voucher specimens indicate floral associations with *Lasthenia* Cass. (Compositae) and *Plagiobothrys*.

#### Discussion.

Brumley (1965) synonymized *E. asperatus* and *E. melectimimus* under *E. americanus*, but current evidence suggests that the holotypes of *E. asperatus* and *E. melectimimus* belong to a cryptic species within the “*americanus* group”, distinct from *E. americanus* and *E. barberiellus*. In addition to the subtle diagnostic morphological features that separate *E. asperatus* from *E. americanus* and *E. barberiellus*, the status of *E. asperatus* as a separate species is supported by a separate BIN and large barcode sequence divergence (4.4%) from its nearest neighbor, *E. barberiellus*.

*Epeolus melectimimus*, with three submarginal cells, was described by Cockerell and Sandhouse (1924), who claimed that the species resembles a small *Pseudomelecta* Radoszkowski (a subgenus of *Melecta* Latreille in Michener 2007), from which it can be readily distinguished based on differences in the marginal cell. In the *E. asperatus* holotype, the second submarginal crossvein on each side is incomplete and inconspicuous. A series of *E. asperatus* was collected from the Robert J. Bernard Biological Field Station in Claremont, California, USA, which is in the same county as the type locality (Los Angeles). In some specimens, the fore wing has three submarginal cells whereas in others, the second submarginal crossvein is incomplete or lacking entirely. In some specimens, one fore wing has three submarginal cells and the other has an incomplete second submarginal crossvein. The male holotype of *E. melectimimus* was examined, and excluding sex-specific features the specimen with few exceptions agrees with the present redescription based on the female holotype of *E. asperatus*. Along with the abovementioned differences in wing venation, the pronotal lobe and tegula are darker in the holotype of *E. melectimimus* than in that of *E. asperatus*, but these differences fall within the range of observed intraspecific morphological variation among sequenced specimens. Although both *E. americanus* and *E. asperatus* are present in California, *E. americanus* appears to be absent from the southern part of the state.

#### Material studied.

**Type material.** Primary: USA: **California**: Huntington Lake (Fresno County), 07.vii.1919, E.P. Van Duzee (*E. melectimimus* holotype ♂ [CAS, catalog number: 01612]); Los Angeles (Los Angeles County), 24.iv.1909, F. Grinnell, Jr. (*E. asperatus* holotype ♀ [USNM, catalog number: 534036]).

#### DNA barcoded material with BIN-compliant sequences.

Available. BOLD:ACZ2142. Specimens examined and sequenced. USA: **California**: Robert J. Bernard Biological Field Station (Claremont, Los Angeles County), 18.iv.2002, M.G. Rightmyer (1♀, KUNHM); Robert J. Bernard Biological Field Station (34.1083°N; 117.7100°W) (Claremont, Los Angeles County), 13.iv.2016, T.M. Onuferko (2♂, PCYU).

#### Non-barcoded material examined.

USA: **California**: 2 mi S Hilmar (Merced County), 19.iv.1960, R.R. Snelling (1♀, AMNH); 2 mi S Pearblossom (Los Angeles County), 01–02.v.1977, R.R. Snelling (1♂, LACM); Arroyo Seco Campground (Monterey County), 01.v.1960, F.D. Parker (1♂, UCBME), 19.v.1964, R.M. Bohart (1♂, UCBME), 11.v.1971, R.M. Bohart (3♀, 2♂, UCBME); Claremont (Los Angeles County), Baker (1♂, USNM), Metz (1♀, AMNH); Devore (San Bernardino County), 21.vi.1974, J.C. and E.M. Hall (1♂, UCR); East Fork Kaweah River (Tulare County), 02.vii.1976, T.L. Griswold (1♀, BBSL); Millard Canyon (Riverside County), 07.iv.1974, J.C. and E.M. Hall (1♀, UCR); Moreno Valley (base of Box Springs Mountains, Riverside County), 26.iv.1992, R.K. Velten (1♀, UCR); Robert J. Bernard Biological Field Station (34.1083°N; 117.7100°W) (Claremont, Los Angeles County), 13.iv.2016, T.M. Onuferko (2♀, 1♂, PCYU), 14.iv.2016, T.M. Onuferko (1♀, PCYU), 26.iv.2016, T.M. Onuferko (3♂, PCYU); W L Jepson Prairie Preserve (TNC) (13 mi S Dixon, Solano County), 20.v.1983, J.D. Barbour (1♂, UCBME).

### 5. Epeolus attenboroughi sp. n.

Taxon classificationAnimaliaHymenopteraApidae

http://zoobank.org/FD2EAACB-3D7A-477C-9B7D-A7EABE7DE10B

**Figure 12.**
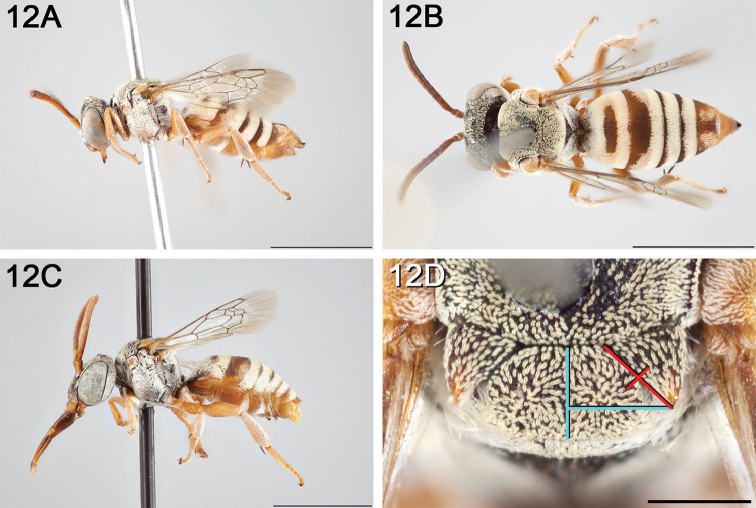
*Epeolus attenboroughi* **A** female holotype, lateral habitus (scale bar 3 mm) **B** female holotype, dorsal habitus (scale bar 3 mm) **C** male allotype, lateral habitus (scale bar 3 mm), and **D** female holotype axillae and mesoscutellum, dorsal view (scale bar 0.5 mm; blue lines indicate the posterior extent of the axilla relative to the length of the mesoscutellum; red lines indicate the extent of the free portion of the axilla relative to its entire medial length).

**Figure 13.**
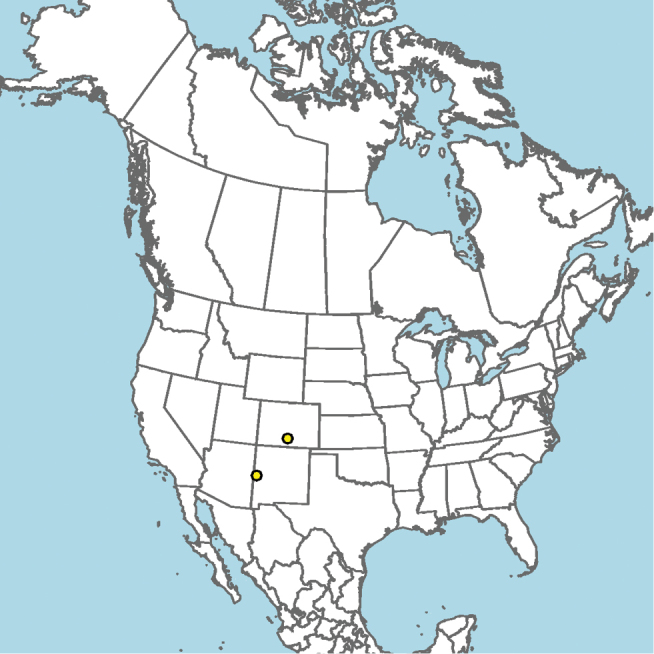
Occurrence records of *E. attenboroughi* known to the author (yellow circles).

Figs 3B , 12 , 13 , 94B , 95B , 96A

#### Diagnosis.

The following morphological features in combination can be used to tell *E. attenboroughi* apart from all other North American *Epeolus* except *E. rufulus*: the mandible has a blunt, obtuse preapical tooth; the preoccipital ridge does not join the hypostomal carina; the mesoscutum is largely obscured by pale tomentum; the axilla is elongate, extending well beyond the midlength of the mesoscutellum but not as far back as its posterior margin, and the free portion is distinctly hooked; the mesopleuron is closely (most i<1d) and evenly punctate; and T1–T4 have complete apical fasciae. Whereas in *E. rufulus* the discal patch is so wide that the longitudinal band is barely visible in dorsal view and in females F2 is noticeably longer than wide, in *E. attenboroughi* T1 has a comparatively narrow discal patch (the longitudinal band is more than half as wide as the breadth of the apical fascia in dorsal view) and in females F2 is less than 1.2 × as long as wide. *Epeolus attenboroughi* is also similar to *E. ainsliei* in that in both species the axilla is dilated laterally and the free portion is distinctly hooked, and the T1–T4 apical fasciae are complete; however, in *E. ainsliei* the mandible is simple, the preoccipital ridge joins the hypostomal carina, and the mesoscutum has distinct paramedian bands.

#### Description.

FEMALE: Length 6.8 mm; head length 1.7 mm; head width 2.2 mm; fore wing length 4.5 mm.

*Integument coloration*. Black in part, at least partially ferruginous on mandible, labrum, clypeus, antenna, pronotal lobe, tegula, axilla, mesopleuron, legs, metasomal terga (including pygidial plate), and metasomal sterna. Mandible with apex darker than rest of mandible; preapical tooth slightly lighter than mandibular apex. Antenna brown and orange in part. Pronotal lobe and tegula pale ferruginous to amber. Wing membrane subhyaline, apically dusky. Legs entirely reddish orange.

*Pubescence*. Face with tomentum densest around antennal socket, slightly sparser on clypeus, upper paraocular and frontal areas, and vertexal area. Dorsum of mesosoma and metasoma with bands of off-white to pale yellow short appressed setae. Mesoscutum, mesoscutellum, and axilla largely obscured by pale tomentum. Mesopleuron densely hairy, except for sparsely hairy circular patch occupying much of ventrolateral half of mesopleuron. Metanotum with tomentum uninterrupted, uniformly off white. T1 with median quadrangular reddish-brown discal patch entirely enclosed by pale tomentum and narrow, such that longitudinal band more than half as wide as breadth of apical fascia in dorsal view. T2–T4 with fasciae complete, T2 with fascia with anterolateral extensions of sparser tomentum. T5 with two patches of pale tomentum lateral to and separate from pseudopygidial area. T5 with pseudopygidial area lunate, its apex more than twice as wide as medial length, indicated by silvery setae on impressed disc of apicomedial region elevated from rest of tergum. S5 with apical fimbria of coppery to silvery hairs extending beyond apex of sternum by ~1/3 MOD.

*Surface sculpture*. Punctures dense. Labrum and clypeus with punctures equally dense (i<1d). Impunctate spot lateral to lateral ocellus absent. Mesoscutum, mesoscutellum, and axilla coarsely and densely rugose-punctate. Tegula very densely punctate (i<1d). Mesopleuron with ventrolateral half densely punctate (i<1d) to rugose; mesopleuron with punctures more or less equally dense throughout. Metasomal terga with punctures very fine, dense (i≈1d), evenly distributed on disc.

*Structure*. Preapical tooth blunt and obtuse. Labrum with pair of small subapical denticles not preceded by carinae. Frontal keel not strongly raised. Scape with greatest length 1.7 × greatest width. F2 not noticeably longer than wide (L/W ratio = 1.1). Preoccipital ridge not joining hypostomal carina, from which it is separated by no less than 1 MOD at its terminal. Mesoscutellum weakly bigibbous. Axilla large, its lateral margin (L) more than half as long as mesoscutellar width (W) (L/W ratio = 0.6) and tip extending well beyond midlength of mesoscutellum but not as far back as its posterior margin; axilla with tip conspicuously diverging from side of mesoscutellum, distinctly hooked, and axilla with free portion approximately half its medial length; axilla with lateral margin arcuate and carinate. Fore wing with three submarginal cells. Pygidial plate apically truncate.

MALE: Description as for female except for usual secondary sexual characters and as follows: F2 shorter, as long as wide (L/W ratio = 1.0); mesopleuron almost entirely obscured by white tomentum; S4 and S5 with much longer coppery to silvery subapical hairs, which individually are often darker apically; pygidial plate apically rounded, with large deep, well-separated punctures, with the interspaces shining.

#### Etymology.

This species is named in honor of English broadcaster and naturalist Sir David Attenborough in recognition of his inspiring books and television programs on natural history.

#### Distribution.

New Mexico and southern Colorado (Fig. 13).

#### Ecology.

HOST RECORDS: The host species of *E. attenboroughi* is/are presently unknown.

FLORAL RECORDS: Unknown.

#### Discussion.

*Epeolus attenboroughi* is similar in overall appearance to *E. ainsliei* and *E. rufulus*, and the ranges of the three species overlap to some extent. Although BIN-compliant sequences are presently not available for *E. attenboroughi*, partial sequences 421 bp and 289 bp in length are available for two specimens (male and female respectively) collected at the same locality and within one day of each other, and there is virtually no divergence (<1%) between the two. Moreover, the 421 bp sequence does not cluster closely with any sequences from other *Epeolus* species in a NJ tree of sequences >300 bp in length (Suppl. material 2). The longer of the two partial sequences is most similar (95.2%) to sequences from *E. glabratus* and *E. lectoides* (very different species).

In general, there is little morphological variation among examined specimens except in integument coloration; the axillae and mesoscutellum range from entirely black to partially ferruginous. Based on known records, adults of *E. attenboroughi* are active in summer.

#### Material studied.

**Type material.** Primary: USA: **Colorado**: Great Sand Dunes National Monument (Alamosa County), 03–13.vii.1989, W.J. Bell (holotype ♀, KUNHM).

Secondary: USA: **Colorado**: Great Sand Dunes National Monument (Alamosa County), 10.vii.1991, B. Cutler (paratype ♀, KUNHM), 03–13.vii.1989, W.J. Bell (paratypes 1♀, 1♂, KUNHM), 11.vii.1991, B. Alexander and B. Cutler (allotype ♂, KUNHM), 11.vii.1991, B. Alexander and B. Cutler (paratypes 3♂, KUNHM); **New Mexico**: 24 km W Quemado (Catron County), 02.ix.1990, T.L. Griswold (paratype ♀, BBSL).

#### DNA barcoded material with BIN-compliant sequences.

Unavailable.

### 6. Epeolus australis

Taxon classificationAnimaliaHymenopteraApidae

Mitchell, 1962

**Figure 14.**
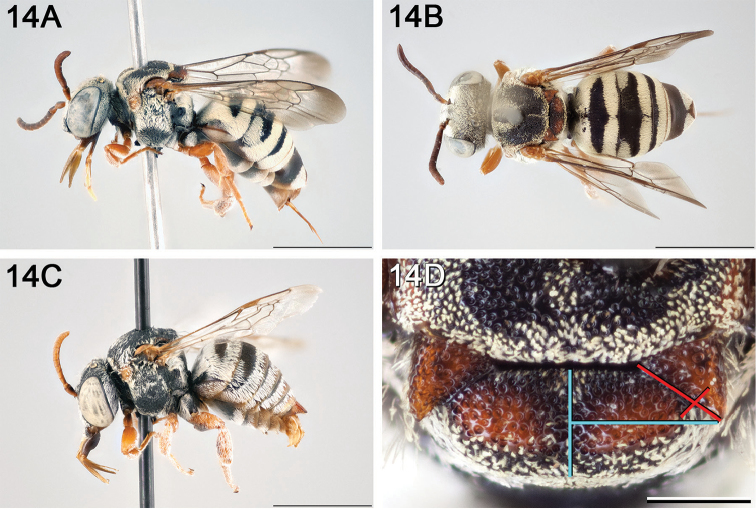
*Epeolus australis* **A** female, lateral habitus (scale bar 3 mm) **B** female, dorsal habitus (scale bar 3 mm) **C** male, lateral habitus (scale bar 3 mm), and **D** female axillae and mesoscutellum, dorsal view (scale bar 0.5 mm; blue lines indicate the posterior extent of the axilla relative to the length of the mesoscutellum; red lines indicate the extent of the free portion of the axilla relative to its entire medial length).

**Figure 15.**
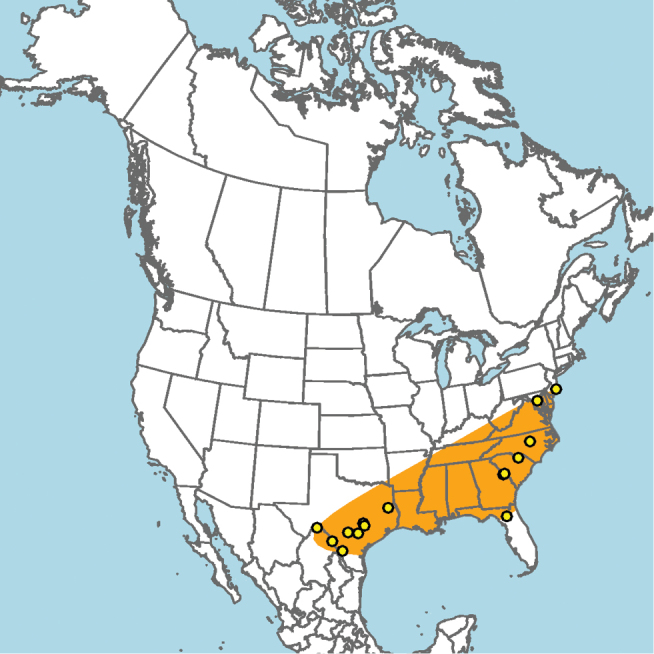
Approximate geographic range of *E. australis* (orange) based on occurrence records known to the author (yellow circles).

Figs 2A , 14 , 15 , 97I , 103A

Epeolus australis Mitchell, 1962. N. C. Agric. Exp. Stn. Tech. Bull. 152: 441 (♀).

#### Diagnosis.

The following morphological features in combination can be used to tell *E. australis* apart from all other North American *Epeolus*: the frontal carina is strongly convex, such that the supraclypeal area is distinctly protuberant in lateral view; T1–T4 have complete fasciae; and the T2 fascia has a pair of anterolateral extensions of tomentum that are strongly convergent basally. In *E. chamaesarachae* and *E. diadematus* and commonly in *E. bifasciatus* the frontal carina is also strongly convex, but in the first two species the vertexal area has two pairs of shiny (usually impunctate) protrusions and in *E. bifasciatus* the frontal area bears a pair of granulose protrusions whereas in *E. australis* the frontal and vertexal areas lack protrusions. *Epeolus australis* most closely resembles *E. brumleyi*, but in *E. brumleyi* the frontal carina is only weakly convex and the pygidial plate of the male is wider (the medial length ≈ the basal width) than in *E. australis* (the medial length is ~1.5 × the basal width).

#### Redescription.

FEMALE: Length 7.5 mm; head length 2.0 mm; head width 2.8 mm; fore wing length 5.7 mm.

*Integument coloration.* Mostly black; notable exceptions as follows: partially to entirely ferruginous on mandible, antenna, pronotal lobe, tegula, axilla, mesoscutellum, legs, pygidial plate, and metasomal sterna. Mandible with apex darker than rest of mandible; preapical tooth slightly lighter than mandibular apex. Both antennae missing in holotype, but brown and orange in part in paratype. Pronotal lobe and tegula pale ferruginous to amber. Wing membrane subhyaline, apically dusky. Legs more extensively reddish orange than brown or black.

*Pubescence.* Face with tomentum densest around antennal socket, slightly sparser on clypeus, upper paraocular and frontal areas, and vertexal area. Dorsum of mesosoma and metasoma with bands of off-white to pale yellow short appressed setae. Mesoscutum with paramedian band. Mesopleuron with upper half densely hairy, except beneath base of fore wing (hypoepimeral area); ventrolateral half sparsely hairy. Metanotum with tomentum uninterrupted, uniformly off white. T1 with discal patch elliptical and very wide, the basal and apical fasciae only narrowly joined laterally. T1 with basal and apical fasciae and T2–T4 with apical fasciae complete, T2 with fascia with basomedially convergent anterolateral extensions of tomentum. T5 with two large patches of pale tomentum lateral to and separate from pseudopygidial area, enclosing pseudopygidial area in triangle, except for medial separation at base. T5 with pseudopygidial area lunate, its apex more than twice as wide as medial length, indicated by silvery setae on disc of apicomedial region elevated from rest of tergum. S5 with apical fimbria of coppery to silvery hairs extending beyond apex of sternum by ~1/3 MOD.

*Surface sculpture.* Punctures dense. Labrum with larger punctures than clypeus, but punctures of both equally dense (i≤1d). Impunctate spot lateral to lateral ocellus absent in holotype, but shiny spot present in some non-type specimens. Mesoscutum, mesoscutellum, and axilla coarsely and densely rugose-punctate. Tegula densely punctate mesally (i≤1d), less so laterally (i=1–2d). Mesopleuron with ventrolateral half densely punctate (i<1d); mesopleuron with punctures more or less equally dense throughout. Metasomal terga with punctures very fine, dense (i≈1d), evenly distributed on disc.

*Structure.* Preapical tooth inconspicuous, blunt and obtuse. Labrum with pair of small subapical denticles (approximately at 1/4 length of labrum from apical margin) not preceded by carinae. Frontal keel strongly raised. Scape (missing in holotype) with greatest length 1.6 × greatest width in paratype. F2 (missing in holotype) not noticeably longer than wide (L/W ratio = 1.1) in paratype. Preoccipital ridge not joining hypostomal carina, from which it is separated by no less than 1 MOD at its terminal. Mesoscutellum moderately bigibbous. Axilla intermediate in size, its lateral margin (L) nearly half as long as mesoscutellar width (W) (L/W ratio = 0.4–0.5) and tip not extending beyond midlength of mesoscutellum; axilla with tip visible, but unattached to mesoscutellum for less than 2/5 the medial length of axilla; axilla with lateral margin relatively straight and without carina. Fore wing with three submarginal cells. Pygidial plate apically truncate.

MALE: Description as for female except for usual secondary sexual characters and as follows: F2 shorter, as long as wide (L/W ratio = 1.0); S4 and S5 with much longer coppery to silvery subapical hairs, which individually are often darker apically; pygidial plate unusually narrow (*Triepeolus*-like) and apically rounded, with large deep punctures closely clustered.

#### Distribution.

Mid-Atlantic states to Texas and presumably Mexico, given the close proximity of some collection localities (e.g., Eagle Pass, Texas) to the Mexico–United States border (Fig. 15).

#### Ecology.

HOST RECORDS: The host species of *E. australis* is/are presently unknown.

FLORAL RECORDS: Mitchell (1962) indicated floral associations with *Ceanothus* L. (Rhamnaceae), *Rubus* L. (Rosaceae), *Senecio* L. (Compositae), and *Specularia* (now *Triodanis*? Raf. ex Greene) (Campanulaceae). Labels of examined voucher specimens further indicate associations with *Chaetopappa asteroides* (Nutt.) Nutt. ex DC. (Compositae), *Hymenopappus artemisiifolius* DC. (Compositae), and *Sphaeralcea*.

#### Discussion.

This southeastern species displays minor sexual dimorphism in the coloration of the mesoscutellum, which is bright ferruginous in females and dark ferruginous to black in males. Otherwise, there is very little morphological variation among examined specimens. Although BIN-compliant sequences are presently not available for *E. australis*, 422 bp sequences were obtained from two male specimens (one from New Jersey, USA and one from South Carolina, USA), and there is virtually no divergence (<1%) between the two. Moreover, these sequences do not cluster with any sequences from other *Epeolus* species in a NJ tree (Suppl. material 2). Based on known records, adults of *E. australis* are active in spring.

#### Material studied.

**Type material.** Primary: USA: **North Carolina**: Raleigh, 19.v.1950, T.B. Mitchell (holotype ♀, NCSU).

Secondary: USA: **North Carolina**: Raleigh, 09.v.1948, T.B. Mitchell (paratype ♀, NHMUK), 19.v.1950, T.B. Mitchell (paratype ♀, USNM).

#### DNA barcoded material with BIN-compliant sequences.

Unavailable.

#### Non-barcoded material examined.

USA: **Florida**: Alachua (Alachua County), 29.iv.1974, E.E. Grissell (2♀, UCBME); **Georgia**: Augusta (Richmond County), 18.v.1959, R.R. Snelling (1♀, LACM), 17.v.1959, R.R. Snelling (1♀, LACM), 03.v.1959, R.R. Snelling (1♂, LACM), 26.iv.1959, R.R. Snelling (1♂, LACM); Fort Gordon (Richmond County), 08.v.1958, R.R. Snelling (1♀, LACM); **Maryland**: Bowie (Prince George’s County), 08.vi.1968, R.R. Snelling (1♂, LACM); **New Jersey**: Forsythe (39.5296°N; 74.3421°W) (Atlantic and Ocean counties), 01–30.vi.2008, M. Springer (1♀, BIML); **South Carolina**: Carolina Sandhills National Wildlife Refuge (34.6043°N; 80.2469°W) (Chesterfield County), 18–19.v.2006, S.W. Droege (1♂, BIML); **Texas**: 10.7 mi S Dryden (Terrell County), 21.iv.1973, R.R. Snelling (1♂, LACM); 12 mi S Seguin (29.4060°N; 97.8550°W) (TX-123, Guadalupe County), 03.v.2014, J.L. Neff (1♀, CTMI); 8–25 km N Castroville (Medina County), 12.v.1988, B.N. Danforth (1♀, KUNHM); Camp Swift (30.2910°N; 97.3060°W) (Bastrop County), 24.iv.2003, J.L. Neff (1♀, CTMI); Eagle Pass (Maverick County), 28.iii.1946, C.D. Michener (2♂, AMNH); Hwy 83 (14 mi S Jct. Texas State Hwy 44, Webb County), 21.iv.1973, R.R. Snelling (1♀, LACM); Nacogdoches (Nacogdoches County), 14.iv.1960 (1♀, KUNHM); Stengl Lost Pines Research Station (30.0800°N; 97.1830°W) (Bastrop County), 02.iv.2006, J.L. Neff (1♀, CTMI).

### 7. Epeolus autumnalis

Taxon classificationAnimaliaHymenopteraApidae

Robertson, 1902

Figs 16 , 17

Epeolus autumnalis Robertson, 1902. Entomol. News 13: 81 (♀, ♂). Webb, 1980. Ill. Nat. Hist. Surv. Bull. 32: 108 (♀) [lectotype designation (by W.E. LaBerge)].

#### Diagnosis.

The following morphological features in combination can be used to tell *E. autumnalis* apart from all other North American *Epeolus*: the axilla is large, with the tip extending well beyond the midlength of the mesoscutellum but not as far back as its posterior margin, dilated laterally, and like the mesoscutellum black; the mesopleuron is closely (i≤1d) and evenly punctate; the T1 discal patch is so wide that the longitudinal band is barely visible in dorsal view; and the T2 fascia lacks lobe-like anterolateral extensions of tomentum, although a few sparsely scattered pale hairs are sometimes present. *Epeolus autumnalis* is similar to *E. scutellaris* in terms of surface sculpture and the patterns of pubescence on the mesosoma and metasoma, but in *E. scutellaris* at least the axilla is partially to entirely ferruginous (as is often the mesoscutellum), and the axilla is more elongate, extending to or beyond the band of pale tomentum along the posterior margin of the mesoscutellum.

**Figure 16.**
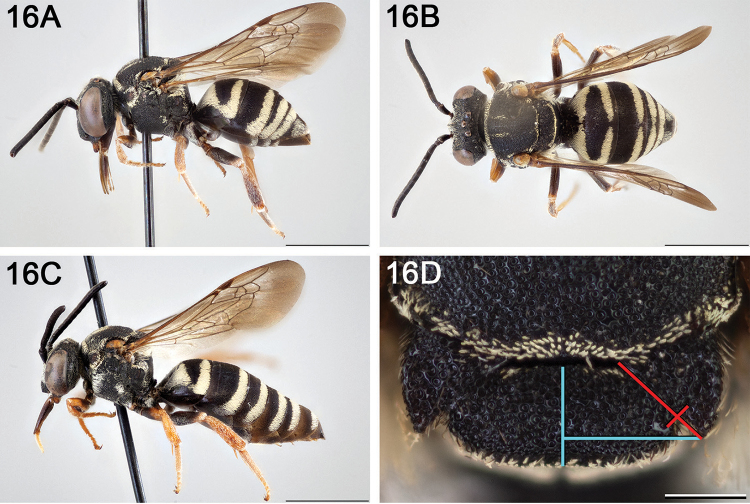
*Epeolus autumnalis* **A** female, lateral habitus (scale bar 3 mm) **B** female, dorsal habitus (scale bar 3 mm) **C** male, lateral habitus (scale bar 3 mm), and **D** female axillae and mesoscutellum, dorsal view (scale bar 0.5 mm; blue lines indicate the posterior extent of the axilla relative to the length of the mesoscutellum; red lines indicate the extent of the free portion of the axilla relative to its entire medial length).

#### Redescription.

This species was recently redescribed (Onuferko 2017).

#### Distribution.

**Figure 17.**
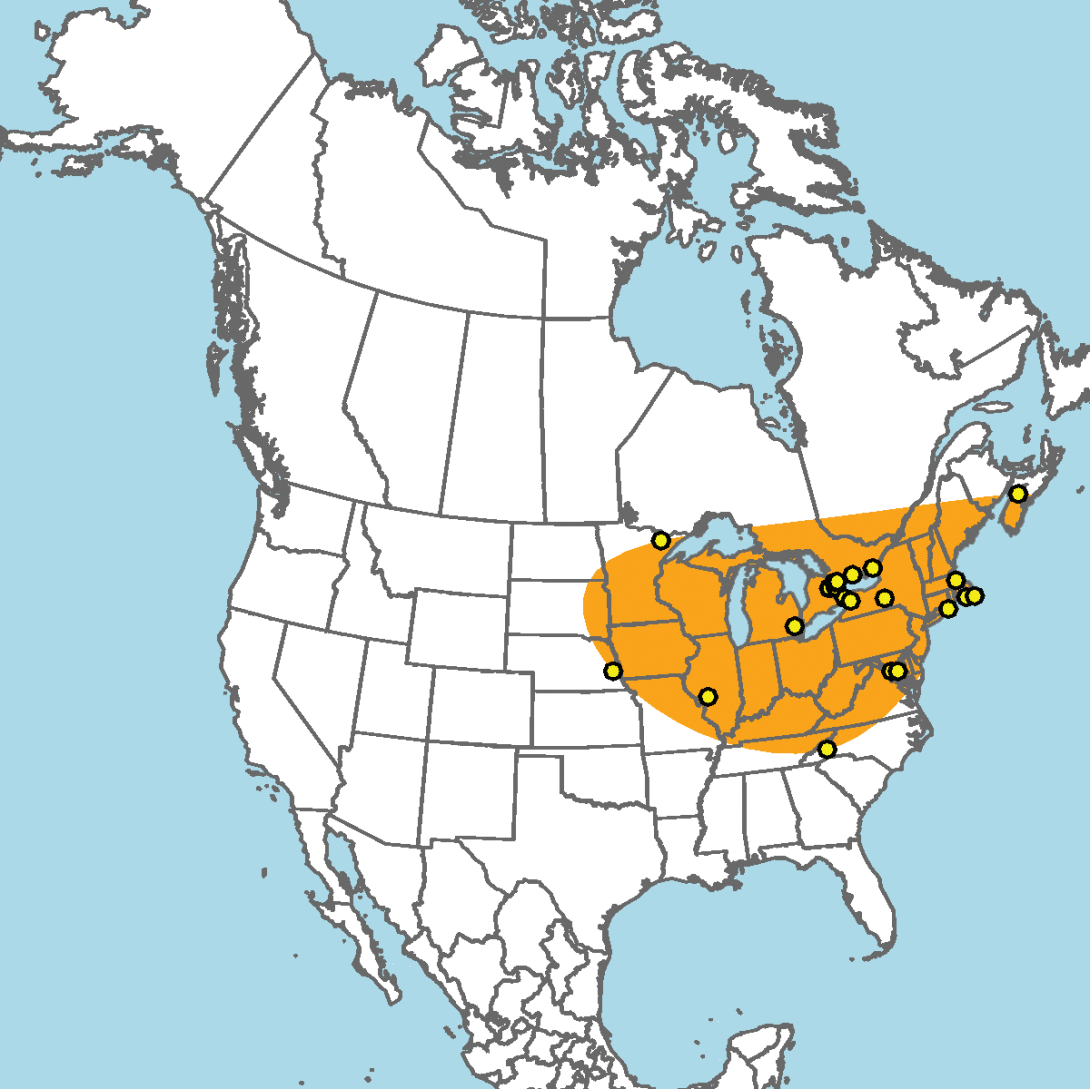
Approximate geographic range of *E. autumnalis* (orange) based on occurrence records known to the author (yellow circles).

Eastern North America (Fig. 17).

#### Ecology.

See Onuferko (2017) for host and floral records. Floral associations are also indicated in Suppl. material 1.

#### Discussion.

Detailed morphological and taxonomic remarks about this species are given in Onuferko (2017).

#### Material studied.

**Type material.** Primary: USA: **Illinois**: Carlinville (Macoupin County), C.A. Robertson (lectotype ♀ [INHS, catalog number: 44381]).

Secondary: USA: **Illinois**: Carlinville (Macoupin County), C.A. Robertson (lectoallotype ♂ [INHS, catalog number: 44382]).

#### DNA barcoded material with BIN-compliant sequences.

Available. BOLD:AAF2361. Specimens examined and sequenced. Canada: **Nova Scotia**: 2♀, 1♂ (PCYU, RSKM); **Ontario**: 1♀ (PCYU).

USA: **New York**: 1♀ (AMNH).

#### Non-barcoded material examined.

Canada: **Nova Scotia**: 2♀ (PCYU, RSKM); Avonport (45.1189°N; 64.2634°W) (Kings County), 27.viii.2000, C. Sheffield (1♂, PCYU); **Ontario**: 14♀, 24♂ (DEBU, PCYU, ROM); King (44.0410°N; 79.5060°W), 23.viii.2000, V. Kushnir (1♂, PCYU); King (44.0430°N; 79.3100°W), 28.viii.2002, V. Kushnir (1♂, PCYU); King (44.0430°N; 79.5410°W), 06.ix.2003, J. Grixti (1♂, PCYU).

USA: **Maryland**: 2♂ (BIML); **Massachusetts**: 1♀, 2♂ (AMNH, BIML); **New York**: 1♀, 1♂ (AMNH, CAS); Lime Hollow (42.5650°N; 76.2550°W) (Cortland County), 03.ix.2011, J. Gibbs (1♂, JBWM); **Virginia**: Glencarlyn, 06.ix.???? (1♂, CUM).

### 8. Epeolus axillaris sp. n.

Taxon classificationAnimaliaHymenopteraApidae

http://zoobank.org/DB3AE149-9E6B-4B37-B329-F264F23DA34B

Figs 18 , 19 , 94A

Epeolus scopulus Brumley, 1965. M.S. thesis, Utah State University, Logan 66 (♀) [*nomen nudum*].

#### Diagnosis.

*Epeolus axillaris* can be differentiated from all other *Epeolus* species in North America by the distinct posteromedial depression of the metanotum; in all other species the metanotum is flat, strongly convex, or weakly convex. *Epeolus axillaris* closely resembles *E. banksi*, *E. minimus*, and *E. olympiellus* in that the axilla (except sometimes the tip) and mesoscutellum are black; T1 has a quadrangular discal patch, in dorsal view the longitudinal band is at least half as wide as the breadth of the apical fascia; and the T2 fascia has lobe-like anterolateral extensions of tomentum. However, in all three species the metanotum is flat and the axilla does not extent much beyond the midlength of the mesoscutellum, whereas in *E. axillaris* the axilla is more elongate, extending well beyond the midlength of the mesoscutellum but not as far back as its posterior margin.

#### Description.

FEMALE: Length 10.0 mm; head length 2.1 mm; head width 2.9 mm; fore wing length 6.9 mm.

*Integument coloration.* Mostly black; notable exceptions as follows: partially to entirely ferruginous on mandible, antenna, pronotal lobe, tegula, axilla, legs, T5, and pygidial plate. Mandible with apex darker than all but extreme base; preapical tooth slightly lighter than mandibular apex (difficult to see in holotype because mandible closed; described from paratypes). Flagellum brown and (except F1) slightly lighter than partially dark brown (otherwise orange) scape, pedicel, and F1, primarily due to extensive pilosity on flagellum. Axilla only with tip orange. Pronotal lobe and tegula pale ferruginous to amber. Wing membrane subhyaline, apically dusky. Legs, except reddish-orange mesotibia, metatibia, and tarsi, with brown or black more extensive than reddish orange.

*Pubescence.* Face with tomentum densest around antennal socket. Dorsum of mesosoma and metasoma with bands of off-white to pale yellow short appressed setae. Mesoscutum with paramedian band wider and joined posteriorly. Mesopleuron densely hairy, except for two sparsely hairy circular patches (one behind pronotal lobe, a larger one occupying much of ventrolateral half of mesopleuron). Metanotum with tomentum uninterrupted except for median bare patch in posterior half, uniformly off white. T1 with median quadrangular black discal patch enclosed by pale tomentum, except for medial separation at apex. T2–T4 with fasciae interrupted medially and narrowed before becoming somewhat broader laterally, T2 with fascia with anterolateral extensions of equally dense tomentum. T5 with two patches of pale tomentum bordering and separate from pseudopygidial area. T5 with pseudopygidial area lunate, its apex more than twice as wide as medial length, indicated by silvery setae on impressed disc of apicomedial region elevated from rest of tergum. S5 with apical fimbria of coppery to silvery hairs extending beyond apex of sternum by ~2/5 MOD.

*Surface sculpture.* Punctures dense. Labrum with larger and sparser punctures (i=1–2d) than clypeus (i<1d). Small impunctate shiny spot lateral to lateral ocellus. Mesoscutum, mesoscutellum, and axilla coarsely and densely rugose-punctate. Tegula very densely punctate mesally (i<1d), less so laterally (i=1–2d). Mesopleuron largely obscured by tomentum, but ventrolateral half densely punctate (i<1d) to rugose where exposed; mesopleuron with punctures more or less equally dense throughout where exposed. Metasomal terga with punctures very fine, dense (i≈1d), evenly distributed on disc.

*Structure.* Labrum with pair of small subapical denticles, each preceded by small discrete longitudinal ridge. Frontal keel not strongly raised. Scape with greatest length 1.7 × greatest width. F2 noticeably longer than wide (L/W ratio = 1.4). Preoccipital ridge not joining hypostomal carina, from which it is separated by about 1.5–2 MOD at its terminal. Mesoscutellum moderately bigibbous. Axilla large, its lateral margin (L) half as long as mesoscutellar width (W) (L/W ratio = 0.5) and tip extending well beyond midlength of mesoscutellum but not as far back as its posterior margin; axilla with tip conspicuously diverging from side of mesoscutellum, distinctly hooked, and axilla with free portion 2/5 its medial length; axilla with lateral margin relatively straight and without carina. Metanotum with posteromedial depression beneath overhanging anterior portion. Fore wing with three submarginal cells. Pygidial plate apically truncate.

MALE: Description as for female except for usual secondary sexual characters and as follows: F2 shorter, not noticeably longer than wide (L/W ratio = 1.1); S4 and S5 with much longer coppery to silvery subapical hairs; pygidial plate apically rounded, with large deep punctures more or less evenly spaced throughout, with the interspaces shining.

**Figure 18.**
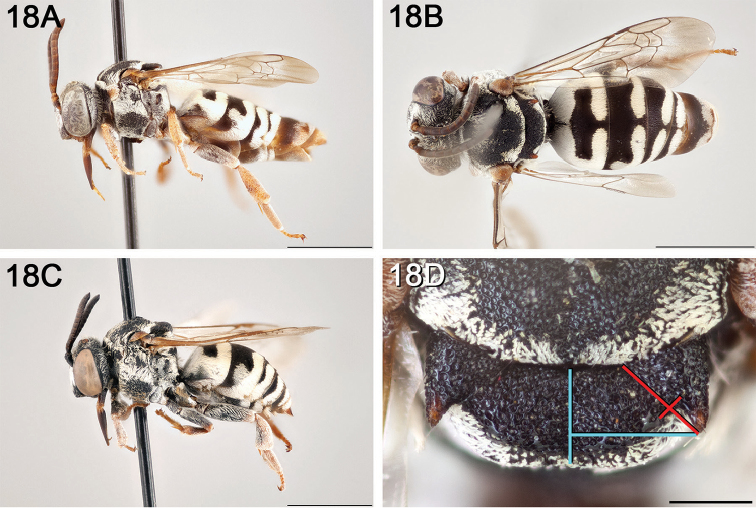
*Epeolus axillaris* **A** female paratype, lateral habitus (scale bar 3 mm) **B** female holotype, dorsal habitus (scale bar 3 mm) **C** male allotype, lateral habitus (scale bar 3 mm), and **D** female paratype axillae and mesoscutellum, dorsal view (scale bar 0.5 mm; blue lines indicate the posterior extent of the axilla relative to the length of the mesoscutellum; red lines indicate the extent of the free portion of the axilla relative to its entire medial length).

#### Etymology.

The name is in reference to the axillae of this species, which are distinctly longer than those of the similar *E. minimus* and *E. olympiellus*.

#### Distribution.

**Figure 19.**
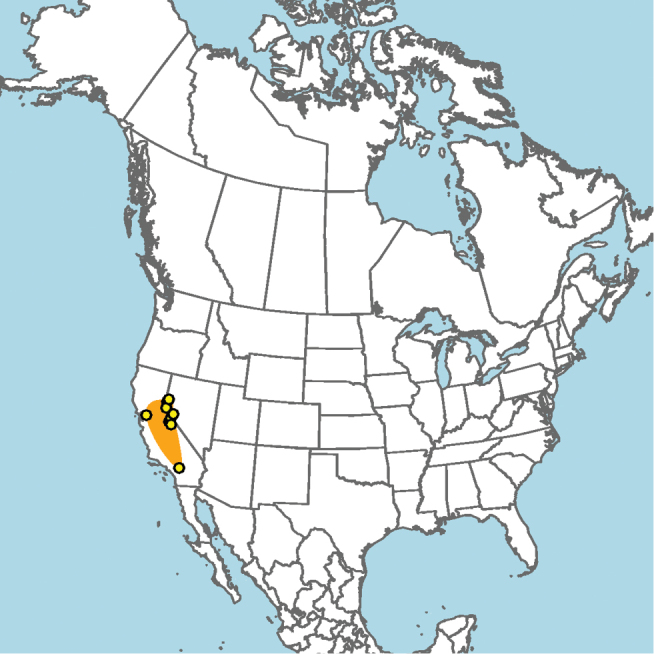
Approximate geographic range of *E. axillaris* (orange) based on occurrence records known to the author (yellow circles).

California and western Nevada. According to Brumley (1965), this species also ranges into Oregon, but its presence in that state could not be verified in the present study (Fig. 19).

#### Ecology.

HOST RECORDS: The host species of *E. axillaris* is/are presently unknown.

FLORAL RECORDS: Labels of examined voucher specimens indicate floral associations with *Chrysothamnus* Nutt. (Compositae) (possibly in reference to plants that now are in the genus *Ericameria* Nutt. (Compositae)), Ericameria nauseosa var. nauseosa (Pall. ex Pursh) G.L. Nesom & Baird, E. nauseosa var. oreophila (A. Nelson) G.L. Nesom & Baird, and *E. parryi* (A. Gray) G.L. Nesom & Baird.

#### Discussion.

This species is most similar to *E. minimus* and *E. olympiellus*, and there is overlap in the ranges of all three species. Brumley (1965) recognized *E. axillaris* as a separate species in which the axilla is more elongate and the metanotum is uniquely depressed posteromedially. The morphological distinction is supported by molecular data, as sequenced specimens exhibiting these attributes were assigned a separate BIN from either of the other two species.

#### Material studied.

**Type material.** Primary: USA: **Nevada**: Cottonwood Creek (38.6013°N; 118.8280°W) (Mineral County), 14.viii.1998, F.D. Parker (holotype ♀ [CCDB-28237 D01], BBSL).

Secondary: USA: **California**: Antioch (Contra Costa County), x.1938, J.A. Downes (paratype ♂, CNC), 10.ix.1947, P.D. Hurd (paratype ♂, BBSL), 10.ix.1947, U.N. Lanham (paratype ♀, CUM); Bodie (Mono County), 21.ix.1958, A.S. Menke and L.A. Stange (paratype ♀, LACM); Hot Creek (Mono County), 29.viii.1969, E.E. Grissell (paratypes 3♀, UCBME), 29.viii.1969, R.M. Bohart (paratype ♂, UCBME); Parker Creek at Walker Lake Road (37.8768°N; 119.1203°W) (Mono County), 02.ix.2009, G.R. Ballmer (allotype ♂ [CCDB-28313 H10], UCR), 02.ix.2009, G.R. Ballmer (paratypes 2♂ (1 barcoded [CCDB-28313 H08]), UCR); Upper Santa Ana River (San Bernardino County), 22.ix.1946, G.H. and J.L. Sperry (paratype ♂, KUNHM); **Nevada**: 17 mi N Sparks (Washoe County), 02.ix.1957, E.G. Linsley (paratype ♀, BBSL), 02.ix.1957, E.G. Linsley (paratype ♀, USNM); 3 mi N Minden (Douglas County), 10.ix.1957, R.C. Bechtel (paratype ♀, AMNH); Reno, 09.ix.1961, F.D. Parker (paratype ♂, UCBME).

#### DNA barcoded material with BIN-compliant sequences.

Available. BOLD:ACZ2412. See Type material for specimens examined and sequenced (indicated by unique CCDB-plate and well number).

### 9. Epeolus banksi

Taxon classificationAnimaliaHymenopteraApidae

(Cockerell, 1907)

Figs 20 , 21 , 96F

Triepeolus banksi Cockerell, 1907a. Entomologist 40: 135 (♂).
Epeolus banksi Mitchell, 1962. N. C. Agric. Exp. Stn. Tech. Bull. 152: 442.

#### Diagnosis.

The following morphological features in combination (excluding any that are specific to the opposite sex of the one being diagnosed) can be used to tell *E. banksi* apart from all other North American *Epeolus* except *E. minimus* and *E. olympiellus*: in females, F2 is at least 1.2 × as long as wide; the mesoscutum has distinct paramedian bands; the axilla is small to intermediate in size, not extending much beyond the midlength of the mesoscutellum (extending to <2/3 its length) but the free portion is more than 1/4 as long as the entire medial length of the axilla, and the axilla and mesoscutellum are black; the mesopleuron is closely (most i<1d) and evenly punctate; T1 has a quadrangular discal patch, in dorsal view the longitudinal band is at least half as wide as the breadth of the apical fascia; and the T2 fascia has anterolateral extensions of tomentum. Whereas in *E. minimus* and *E. olympiellus* the mesoscutum and metasomal terga have bands of off-white to pale yellow short appressed setae, in *E. banksi* the mesoscutum and metasomal terga have bands of gray short appressed setae. In *E. banksi*, the integument is entirely dark brown or black. In *E. olympiellus*, at least the pronotal lobe is ferruginous. In *E. minimus* from California, the integument is often entirely dark brown or black, but throughout most of its range *E. minimus* exhibits reddish-orange coloration on the labrum, antenna, pronotal lobe, and/or legs, except foreleg, from trochanters to tarsi. Both sexes of *E. banksi* are larger (~10 mm in length) on average than *E. minimus* or *E. olympiellus* (7–8 mm in length).

#### Redescription.

MALE: Length 9.4 mm; head length 2.3 mm; head width 3.3 mm; fore wing length 7.5 mm.

*Integument coloration*. Mostly black; notable exceptions as follows: at least partially ferruginous on mandible, antenna, tegula, and legs. Mandible black except apex reddish brown; preapical tooth same color as mandibular apex (difficult to see in holotype; described from non-type specimens). Flagellum, except right F1 and F2, missing in holotype, but brown and (except F1) slightly lighter than conspicuously dark brown scape and pedicel, primarily due to extensive pilosity on flagellum, in non-type specimens. Wing membrane subhyaline, apically dusky. Legs, except reddish-orange tarsi, with brown or black more extensive than reddish orange.

*Pubescence*. Face with tomentum densest on clypeus and around antennal socket, sparser on upper paraocular area and vertexal area. Dorsum of mesosoma and metasoma with bands of off-white to pale gray short appressed setae. Mesoscutum with paramedian band. Mesopleuron densely hairy, except for two sparsely hairy circular patches (one behind pronotal lobe, a larger one occupying much of ventrolateral half of mesopleuron). Metanotum with tomentum uninterrupted, uniformly off white. T1 with median quadrangular black discal patch enclosed by pale tomentum, except for medial separation at apex. T2–T6 with fasciae interrupted medially, those of T2–T4 narrowed before becoming somewhat broader laterally, T2 with fascia with anterolateral extensions of sparser tomentum. S4 and S5 with long coppery to silvery subapical hairs, which individually are often darker apically.

*Surface sculpture*. Punctures dense. Labrum with larger and sparser punctures (i=1–2d) than clypeus (i<1d). Small impunctate matte spot lateral to lateral ocellus. Mesoscutum, mesoscutellum, and axilla coarsely and densely rugose-punctate. Tegula very densely punctate mesally (i<1d), less so laterally (i=1–2d). Mesopleuron with ventrolateral half densely punctate (i<1d); mesopleuron with punctures more or less equally dense throughout. Metasomal terga with punctures very fine, dense (i≈1d), evenly distributed on disc.

*Structure*. Labral apex with pair of small denticles, each preceded by longitudinal carina. Frontal keel not strongly raised. Scape with greatest length 1.6 × greatest width. F2 noticeably longer than wide (L/W ratio = 1.2). Preoccipital ridge not joining hypostomal carina, from which it is separated by about 1.5–2 MOD at its terminal. Mesoscutellum moderately bigibbous. Axilla intermediate in size, its lateral margin (L) nearly half as long as mesoscutellar width (W) (L/W ratio = 0.4–0.5) and tip not extending much beyond midlength of mesoscutellum (extending to <2/3 its length); axilla with tip clearly visible, but unattached to mesoscutellum for less than 2/5 the medial length of axilla; axilla with lateral margin relatively straight and without carina. Fore wing with three submarginal cells. Pygidial plate apically rounded, with large deep punctures closely clustered.

FEMALE: Description as for male except for usual secondary sexual characters and as follows: F2 even longer than wide (L/W ratio = 1.4); T5 with two patches of pale tomentum bordering and separate from pseudopygidial area present only in female; T5 with pseudopygidial area lunate, its apex more than twice as wide as medial length, indicated by silvery setae on flat disc of apicomedial region elevated from rest of tergum; S4 and S5 with much shorter hairs (S5 with apical fimbria of coppery to silvery hairs extending beyond apex of sternum by ~2/5 MOD); pygidial plate apically truncate, with small, denser punctures.

**Figure 20.**
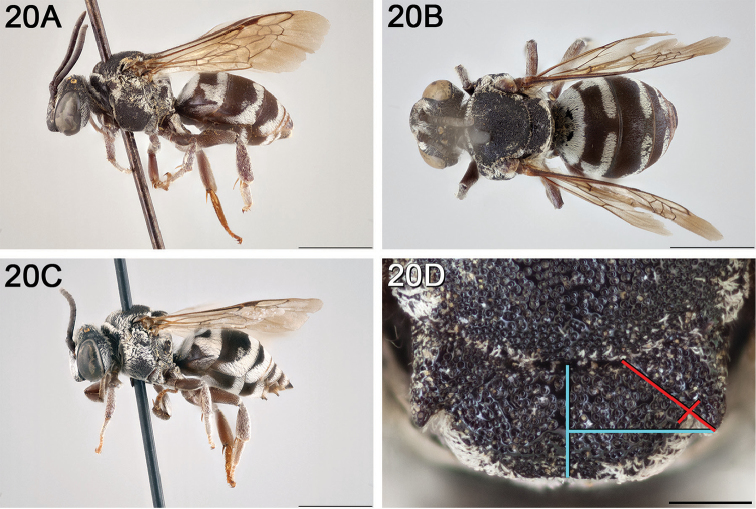
*Epeolus banksi* **A** female, lateral habitus (scale bar 3 mm) **B** female, dorsal habitus (scale bar 3 mm) **C** male, lateral habitus (scale bar 3 mm), and **D** female axillae and mesoscutellum, dorsal view (scale bar 0.5 mm; blue lines indicate the posterior extent of the axilla relative to the length of the mesoscutellum; red lines indicate the extent of the free portion of the axilla relative to its entire medial length).

#### Distribution.

**Figure 21.**
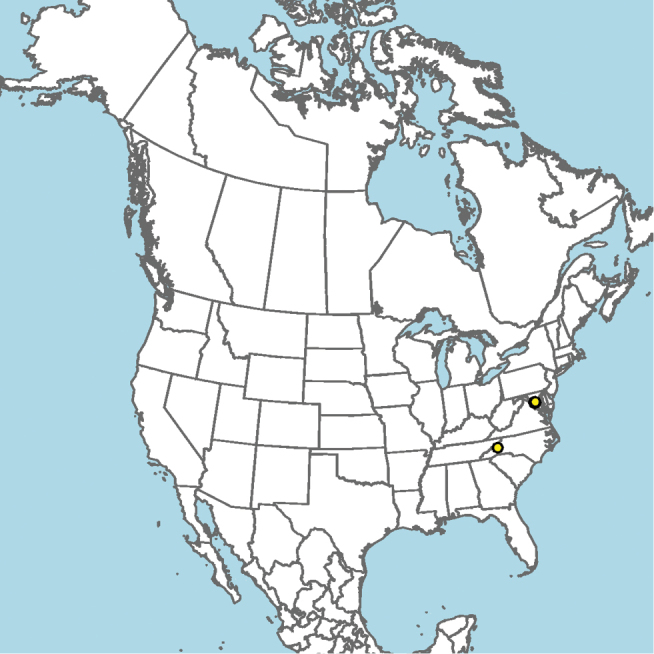
Occurrence records of *E. banksi* known to the author (yellow circles).

Maryland to North Carolina (Fig. 21).

#### Ecology.

HOST RECORDS: The host species of *E. banksi* is/are presently unknown.

FLORAL RECORDS: Mitchell (1962) indicated a floral association with *Fragaria* L. (Rosaceae). Labels of examined voucher specimens further indicate associations with *Solidago* L. (Compositae) and *Symphyotrichum ericoides* (L.) G.L. Nesom (Compositae).

#### Discussion.

Most of the specimens of this species that were examined were collected in the Washington metropolitan area. While Mitchell (1962) indicated *Epeolus banksi* as being quite prevalent across the Eastern United States, reportedly ranging from Minnesota to New Jersey and North Carolina, it seems that the name has been commonly misapplied to specimens of *E. minimus* (as in MacKay and Knerer (1979) for example, and probably by Mitchell (1962) as well). *Epeolus banksi* is much larger than *E. minimus*, and has completely black integument, but unlike similarly dark specimens of *E. minimus* from California, *E. banksi* has gray as opposed to pale yellow bands of tomentum on the mesosoma and metasoma. Unfortunately, no recently collected material was available for barcode sequencing, and the specimens seen are all from the early 1900s. The absence of this species from recent collections has not gone unnoticed (e.g in Colla et al. 2012 it is listed among the bee species not collected since 1990). Increased urbanization in and around Washington D.C. may have resulted in the extirpation of this species there, and perhaps it has even disappeared entirely throughout its earlier range. Hence, extensive efforts should be made to rediscover this species, by sampling its apparent historical range between North Carolina and Maryland, to assess its conservation status. The flight season of *E. banksi* appears to be late summer/early autumn.

#### Material studied.

**Type material.** Primary: USA: **Virginia**: Falls Church, 26.viii.????, N. Banks (holotype ♂ [USNM, catalog number: 534038]).

Secondary: USA: **Virginia**: Falls Church, 07.ix.????, N. Banks (paratype ♂, CAS).

#### DNA barcoded material with BIN-compliant sequences.

Unavailable.

#### Non-barcoded material examined.

USA: **Maryland**: Glen Echo (Montgomery County), 30.viii.1923, J.R. Malloch (1♂, USNM); **North Carolina**: Valley of Black Mountains, 30.ix.1906, W. Beutenmuller (1♂, AMNH); **Virginia**: Chain Bridge, 10.ix.1922, J.R. Malloch (1♂, USNM); Falls Church, G.G. Rohwer (1♂, USNM); Glencarlyn?, 20.ix.??30 (1♂, USNM); **Washington, D.C.** (2♀, BBSL); Rock Creek Park, 28.viii.1919, J.C. Crawford (1♂, AMNH).

### 10. Epeolus barberiellus

Taxon classificationAnimaliaHymenopteraApidae

Cockerell, 1907

**Figure 22.**
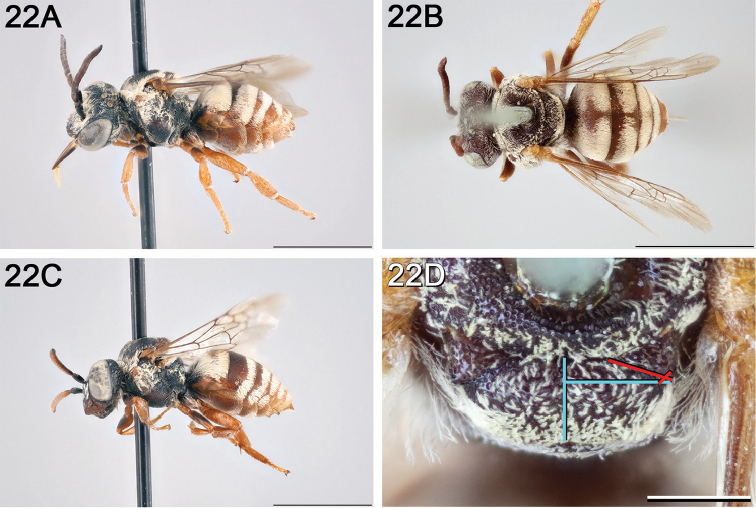
*Epeolus barberiellus* **A** female, lateral habitus (scale bar 3 mm) **B** female holotype, dorsal habitus (scale bar 3 mm) **C** male, lateral habitus (scale bar 3 mm), and **D** female holotype axillae and mesoscutellum, dorsal view (scale bar 0.5 mm; blue lines indicate the posterior extent of the axilla relative to the length of the mesoscutellum; red lines indicate the extent of the free portion of the axilla relative to its entire medial length).

**Figure 23.**
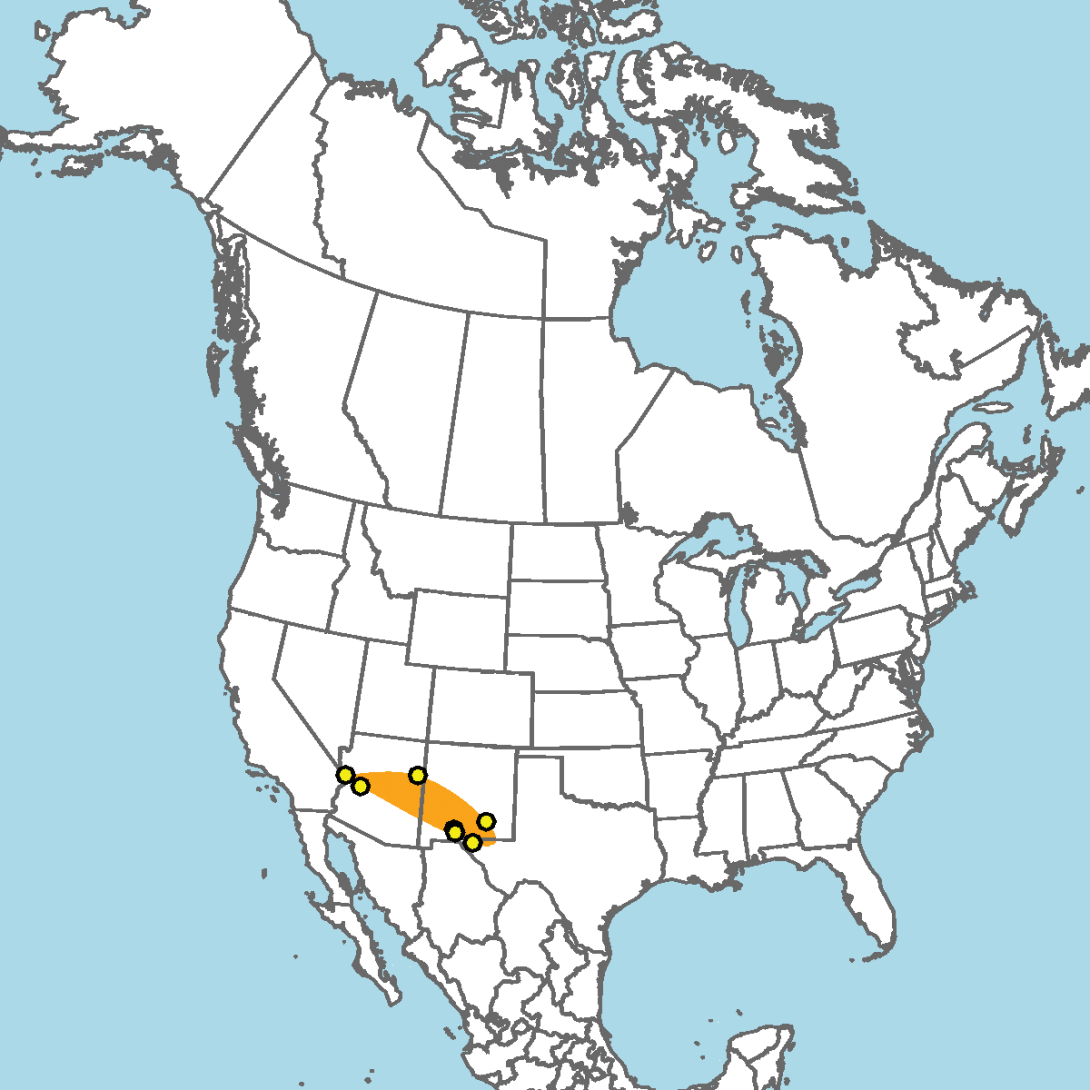
Approximate geographic range of *E. barberiellus* (orange) based on occurrence records known to the author (yellow circles).

Figs 2E , 22 , 23 , 96E

Epeolus barberiellus Cockerell, 1907b. Entomologist 40: 266 (♀).

#### Diagnosis.

The following morphological features in combination (excluding any that are specific to the opposite sex of the one being diagnosed) can be used to tell *E. barberiellus* apart from all other North American *Epeolus* except *E. americanus* and *E. asperatus*: in females, F2 is not more than 1.1 × as long as wide; the mesoscutum has distinct paramedian bands; the axilla is small to intermediate in size, not extending beyond the midlength of the mesoscutellum and the free portion is less than 1/4 as long as the entire medial length of the axilla, and like the mesoscutellum black; the mesopleuron is closely (i≤1d) and evenly punctate; T1 has a quadrangular discal patch, in dorsal view the longitudinal band is at least as wide as the breadth of the apical fascia; and the T1 and T2 apical fasciae are interrupted or at least greatly narrowed medially. In *E. asperatus* the mesopleuron has much denser punctures ventrolaterally (most i<1d) than that of *E. barberiellus* and the T3 and T4 fasciae are never complete but broken or at least greatly narrowed laterally, as well as medially into separated or narrowly connected oval patches. *Epeolus barberiellus* is most similar to *E. americanus*, but in *E. americanus* the pronotal lobe and legs are brown or black, not reddish orange.

#### Redescription.

FEMALE: Length 5.7 mm; head length 1.8 mm; head width 2.3 mm; fore wing length 5.0 mm.

*Integument coloration*. Mostly black; notable exceptions as follows: at least partially ferruginous on mandible, labrum, antenna, pronotal lobe, tegula, mesopleuron, metapleuron, propodeum, legs, metasomal terga (including pygidial plate), and metasomal sterna. Mandible with apex darker than rest of mandible; preapical tooth as dark as mandibular apex (difficult to see in holotype because mandible closed; described from non-type specimens). Pedicel and flagellum brown and orange in part, slightly lighter than dark brown scape. Pronotal lobe reddish brown. Tegula pale ferruginous to amber. Wing membrane subhyaline, apically dusky. Legs more extensively reddish orange than brown or black. T5 and pygidial plate reddish orange.

*Pubescence*. Face with tomentum densest around antennal socket. Dorsum of mesosoma and metasoma with bands of off-white to pale yellow short appressed setae. Mesoscutum with paramedian band and moderately dense pale tomentum along margins. Mesopleuron densely hairy, except for almost entirely bare circular patch occupying much of ventrolateral half of mesopleuron. Metanotum with tomentum uninterrupted, uniformly off white. T1 with median quadrangular reddish-brown discal patch enclosed by pale tomentum, except for medial separation at apex, and narrow, such that longitudinal band more than half as wide as width of discal patch in dorsal view. T2 with fascia interrupted medially and without anterolateral extensions of tomentum, although fascia broader laterally with hairs sparser basally. T3 and T4 with fasciae complete and narrowed laterally. T5 with two patches of pale tomentum lateral to and separate from pseudopygidial area. T5 with pseudopygidial area lunate, its apex more than twice as wide as medial length, indicated by silvery setae on impressed disc of apicomedial region elevated from rest of tergum. S5 with apical fimbria of coppery to silvery hairs not extending beyond apex of sternum by more than 1/4 MOD.

*Surface sculpture*. Punctures dense. Labrum with larger and sparser punctures (i=1–2d) than clypeus (i<1d). Impunctate spot lateral to lateral ocellus absent in holotype, but shiny spot present in non-type specimens. Mesoscutum, mesoscutellum, and axilla coarsely and densely rugose-punctate. Tegula densely punctate mesally (i≤1d), less so laterally (i=1–2d). Mesopleuron with ventrolateral half densely punctate (i≤1d), the interspaces shining; mesopleuron with punctures more or less equally dense throughout. Metasomal terga with punctures very fine, dense (i≈1d), evenly distributed on disc.

*Structure*. Labrum with pair of small subapical denticles not preceded by carinae. Frontal keel not strongly raised. Scape with greatest length 1.9 × greatest width. F2 as long as wide (L/W ratio = 1.0). Preoccipital ridge not joining hypostomal carina, from which it is separated by about 1.5–2 MOD at its terminal (difficult to see in holotype; described from non-type specimens). Mesoscutellum moderately bigibbous. Axilla small to intermediate in size, its lateral margin (L) less than half as long as mesoscutellar width (W) (L/W ratio = 0.3) and tip not extending beyond midlength of mesoscutellum; axilla with tip visible, but unattached to mesoscutellum for less than 1/4 the medial length of axilla; axilla with lateral margin relatively straight and without carina. Fore wing with three submarginal cells. Pygidial plate apically truncate.

MALE: Description as for female except for usual secondary sexual characters and as follows: F2 shorter, nearly as long as wide (L/W ratio = 0.8); S4 and S5 with much longer coppery to silvery subapical hairs, which individually are often darker apically; pygidial plate orange and V-shaped but apically rounded, with large deep punctures closely clustered.

#### Distribution.

Arizona to west Texas (Fig. 23).

#### Ecology.

HOST RECORDS: The host species of *E. barberiellus* is/are presently unknown.

FLORAL RECORDS: Labels of examined voucher specimens indicate floral associations with *Aster* (possibly in reference to a plant that is in a different genus now) (Compositae) and *Sphaeralcea*.

#### Discussion.

*Epeolus barberiellus* is most similar to *E. americanus*, from which it differs consistently only in integument coloration. Although sequenced representatives of both forms share the same BIN, specimens identified as *E. barberiellus* cluster separately from those identified as *E. americanus* (Suppl. material 2). Whereas *E. americanus* is widely distributed across North America, *E. barberiellus* appears to be restricted to the Southwestern United States (and possibly adjacent Mexico), where it replaces the much darker form that characterizes *E. americanus*. Taken together, these differences are indicative of divergence, and therefore the two forms are herein considered to be heterospecific. Brumley (1965) also considered *E. americanus* and *E. barberiellus* as separate species, but synonymized *E. asperatus* and *E. melectimimus* under *E. americanus*. In the present study, three valid species in the “*americanus* group” (*E. americanus*, *E. asperatus*, and *E. barberiellus*) are recognized, of which only *E. asperatus* has been assigned a separate BIN, suggesting that *E. americanus* and *E. barberiellus* are sister species.

The male of *E. barberiellus* is described here for the first time. Of the *Epeolus* in the “*americanus* group”, this appears to be the least commonly collected species.

#### Material studied.

**Type material.** Primary: USA: **New Mexico**: Mesilla Park, 22.iv.????, C.M. Barber (holotype ♀ [USNM, catalog number: 534039]).

#### DNA barcoded material with BIN-compliant sequences.

Available. BOLD:AAB9110. Specimens examined and sequenced.—USA: **New Mexico**: Sagebrush Valley Rd (32.9500°N; 104.8333°W) (Artesia), 01–10.v.2004, M.E. Irwin (1♂, BBSL).

#### Non-barcoded material examined.

USA: **Arizona**: 2 mi SW Apache (Cochise County), 19.iv.1961, Gertsch, Rozen, and Schrammel (1♀, AMNH); 31 mi N Wickenburg, 21.iv.1967, P. Torchio and N. Youssef (1♂, LACM); 40 mi S Kingman (Mohave County), 21.iv.1967, P. Torchio and N. Youssef (1♀, BBSL); **New Mexico**: 12 mi N Las Cruces (Doña Ana County), 11.iv.1965, F.D. Parker (1♂, BBSL); **Texas**: 9.4 mi E Cornudas (Hudspeth County), 27.iv.1998, T., S., and L. Griswold (1♀, BBSL).

### 11. Epeolus basili sp. n.

Taxon classificationAnimaliaHymenopteraApidae

http://zoobank.org/764C92DA-591F-4302-9337-C9C32D2AD4D8

**Figure 24.**
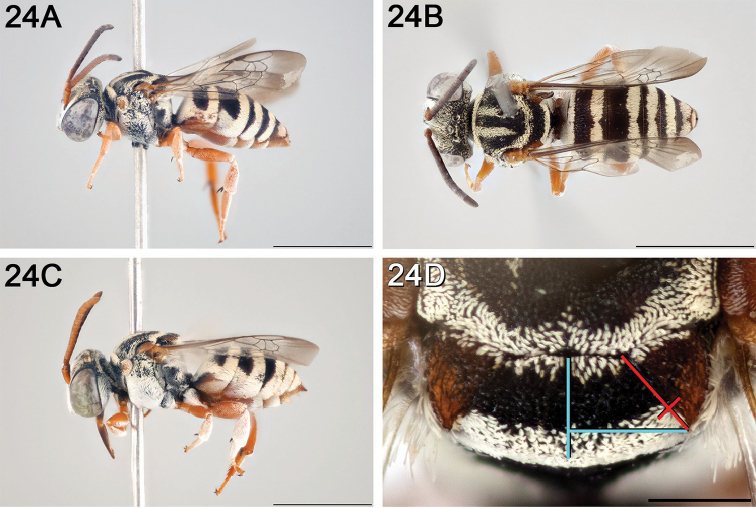
*Epeolus basili* **A** female holotype, lateral habitus (scale bar 3 mm) **B** female holotype, dorsal habitus (scale bar 3 mm) **C** male allotype, lateral habitus (scale bar 3 mm), and **D** female paratype axillae and mesoscutellum, dorsal view (scale bar 0.5 mm; blue lines indicate the posterior extent of the axilla relative to the length of the mesoscutellum; red lines indicate the extent of the free portion of the axilla relative to its entire medial length).

**Figure 25.**
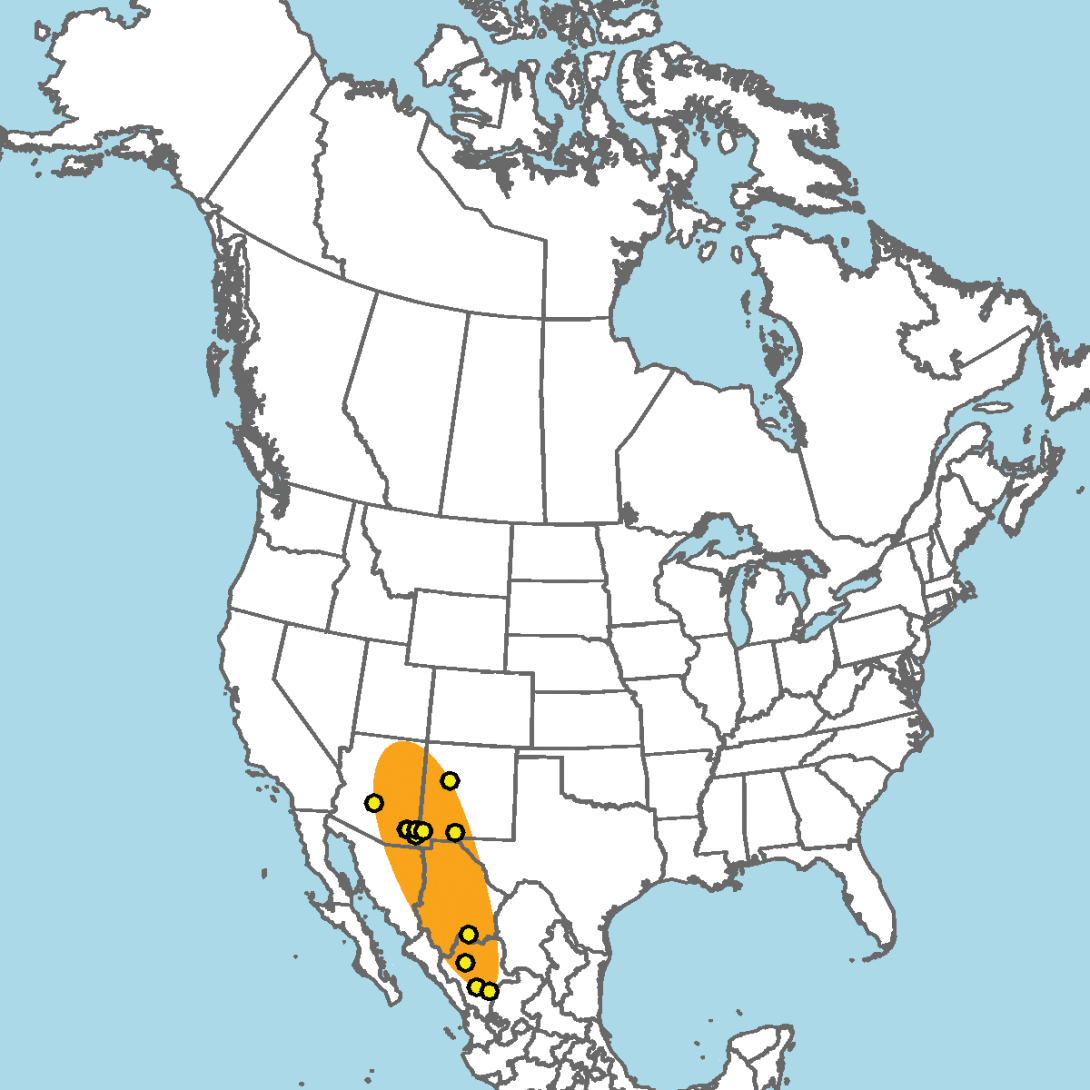
Approximate geographic range of *E. basili* (orange) based on occurrence records known to the author (yellow circles).

Figs 24 , 25 , 97D , 98B

#### Diagnosis.

The following morphological features in combination (excluding any that are specific to the opposite sex of the one being diagnosed) can be used to tell *E. basili* apart from all other North American *Epeolus* except *E. nebulosus*, *E. novomexicanus*, and *E. pusillus*: the axilla is large, with the tip extending well beyond the midlength of the mesoscutellum but at most to the band of pale tomentum along its posterior margin, dilated laterally, and usually ferruginous to some degree (rarely all black) whereas the mesoscutellum ranges from entirely black to partially ferruginous; the axilla’s free portion is clearly less than 2/5 as long as its entire medial length; the mesopleuron is closely (most i<1d) and evenly punctate, that of the female is obscured by white tomentum only in the upper half (with a large, sparsely hairy circle occupying much of the ventrolateral half) whereas that of the male (excluding the hypoepimeral area) is entirely obscured by white tomentum; the T1–T3 apical fasciae are complete or only very narrowly interrupted medially; the T2 fascia has lobe-like anterolateral extensions of tomentum; and the pseudopygidial area of the female is lunate with the apex at least 2 × and clearly <2.5 × the medial length. *Epeolus basili*, *E. nebulosus*, *E. novomexicanus*, and *E. pusillus* are all extremely similar to one another. Whereas in *E. pusillus* the flagellum, except sometimes F1, and metasomal sterna are consistently brown or black and clearly not the same reddish-orange color as the legs (tibiae to tarsi), in *E. basili* the flagellum, at least ventrally, is the same reddish-orange color as the legs (tibiae to tarsi) as are usually the metasomal sterna. In *E. nebulosus* and *E. novomexicanus* the T2–T4 fasciae are on or very little removed from the apical margin, and in both species as well as in *E. pusillus* the pseudopygidial area of the female is commonly less and no more than 2 × the medial length. By contrast, in *E. basili* the T2 and T3 (for female) or T2–T4 (for male) fasciae are narrowed medially and removed from the apical margin, and the pseudopygidial area of the female is ≥2 × the medial length. *Epeolus basili* is also similar to *E. scutellaris* in that the axilla is large, with the lateral margin arcuate, and that the apical fasciae are complete or only very narrowly interrupted medially. However, in *E. scutellaris* the pseudopygidial area of the female is even wider (the apex ~2.5–3 × the medial length) than in *E. basili*, and the mesopleuron of both the female and male is obscured by white tomentum only in the upper half (with a large, sparsely hairy circle occupying much of the ventrolateral half).

#### Description.

FEMALE: Length 7.0 mm; head length 1.8 mm; head width 2.5 mm; fore wing length 4.8 mm.

*Integument coloration.* Mostly black; notable exceptions as follows: at least partially ferruginous on mandible, labrum, antenna, pronotal lobe, tegula, axilla, legs, and metasomal sterna. Mandible with apex darker than rest of mandible; preapical tooth slightly lighter than mandibular apex (difficult to see in holotype; described from paratypes). Antenna brown and orange in part. Pronotal lobe and tegula pale ferruginous to amber. Wing membrane subhyaline, apically dusky. Legs more extensively reddish orange than brown or black. S1–S5 reddish orange.

*Pubescence.* Face with tomentum densest around antennal socket, slightly sparser on clypeus, upper paraocular and frontal areas, and vertexal area. Dorsum of mesosoma and metasoma with bands of off-white to pale yellow short appressed setae. Mesoscutum with paramedian band. Mesopleuron densely hairy, except for sparsely hairy circular patch occupying much of ventrolateral half of mesopleuron. Metanotum with tomentum uninterrupted, uniformly off white. T1 with discal patch quadrangular and very wide, the basal and apical fasciae only narrowly joined laterally. T1–T3 with apical fasciae complete (basal fascia of T1 also), narrowed medially, and removed from apical margin, most noticeably at midline; T2 with fascia with anterolateral extensions of tomentum. T4 with fascia complete. T5 with large, continuous patch of pale tomentum bordering and separate from pseudopygidial area. T5 with pseudopygidial area lunate, its apex twice as wide as medial length, indicated by silvery setae on flat disc of apicomedial region elevated from rest of tergum. S5 with apical fimbria of coppery to silvery hairs extending beyond apex of sternum by ~2/5 MOD.

*Surface sculpture.* Punctures dense. Labrum with larger and sparser punctures (i=1–2d) than clypeus (i<1d). Small impunctate shiny spot lateral to lateral ocellus. Mesoscutum, mesoscutellum, and axilla coarsely and densely rugose-punctate. Tegula densely punctate mesally (i≤1d), less so laterally (i=1–2d). Mesopleuron with ventrolateral half densely punctate (i≤1d) to rugose; mesopleuron with punctures more or less equally dense throughout. Metasomal terga with punctures very fine, dense (i≈1d), evenly distributed on disc.

*Structure.* Preapical tooth obtuse. Labrum with pair of small subapical denticles not preceded by carinae. Frontal keel not strongly raised. Scape with greatest length 1.9 × greatest width. F2 noticeably longer than wide (L/W ratio = 1.4). Preoccipital ridge not joining hypostomal carina, from which it is separated by no less than 1 MOD at its terminal. Mesoscutellum weakly bigibbous. Axilla large, its lateral margin (L) half as long as mesoscutellar width (W) (L/W ratio = 0.5) and tip extending well beyond midlength of mesoscutellum but not as far back as its posterior margin; axilla with tip clearly visible, but unattached to mesoscutellum for less than 2/5 the medial length of axilla; axilla with lateral margin arcuate. Fore wing with three submarginal cells. Pygidial plate apically truncate.

MALE: Description as for female except for usual secondary sexual characters and as follows: F2 shorter, but still longer than wide (L/W ratio = 1.2); mesopleuron (excluding hypoepimeral area) entirely obscured by white tomentum; S4 and S5 with much longer coppery to silvery subapical hairs; pygidial plate apically rounded, with large deep, well-separated punctures, with the interspaces shining.

#### Etymology.

This species is named in honor of my brother, Basil V. Onuferko (1986–2013).

#### Distribution.

Northwestern Mexico and southwestern United States (Fig. 25).

#### Ecology.

HOST RECORDS: This species has been collected east of Willcox, Arizona, USA in the presence of large numbers of *Colletes tectiventris* Timberlake (E. Wyman, personal communication, 2014).

FLORAL RECORDS: Labels of examined voucher specimens indicate floral associations with *Isocoma hartwegii* (A. Gray) Greene (Compositae), *I. tenuisecta* Greene, *Pectis papposa* Harv. & A. Gray (Compositae), *Psorothamnus scoparius* (A. Gray) Rydb. (Leguminosae), and *Wislizenia refracta* Engelm. (Cleomaceae).

#### Discussion.

Structurally, this species is indistinguishable from the other three members of the “*pusillus* group”, and although consistent, the features (differences in integument coloration and patterns of pubescence) that in combination may be used to distinguish *E. basili* from *E. nebulosus*, *E. novomexicanus*, and *E. pusillus* are subtle. Its status as a separate species is supported by a separate BIN and large barcode sequence divergence (>7.3%) from its nearest neighbor, *E. pusillus*. In the United States, *Epeolus basili* appears to be restricted to parts of the American Southwest, east of California.

#### Material studied.

**Type material.** Primary: USA: **Arizona**: 4 mi E Willcox (Cochise County), 29.viii.2013, J.S. Ascher (holotype ♀ [CCDB-22791 A05], AMNH).

Secondary: Mexico: **Chihuahua**: 9 mi S Hidalgo del Parral, 31.vii.1967, R.C. Gardner, C.R. Kovacic, and K. Lorenzen (paratype ♂, UCBME); **Durango**: Nombre de Dios, 01.viii.1951, P.D. Hurd (paratypes 1♀, 1♂, EMEC); Otinapa, 11.viii.1947, D. Rockefeller Exp. Michener (paratype ♀, AMNH); Tepehuanes, 1933, Wickham (paratype ♀, USNM).

USA: **Arizona**: 11 mi S San Simon, 02.ix.2013, G. Rowe (paratype ♀, PCYU); 1–3 mi SE Willcox (Cochise County), 25.viii.1994, J.G. Rozen and J.S. Ascher (paratype ♂, AMNH); 2 mi SE Willcox (Cochise County), 05.ix.1986, J.G. and B.L. Rozen (paratype ♀, AMNH); 4 mi E Willcox (Cochise County), 02.ix.2013, C. Lin (paratype ♂, AMNH), 02.ix.2013, Z. Soh (paratypes 2♂, AMNH), 03.ix.2015, R. González Vaquero (paratype ♂, PCYU), 06.ix.2012, J.G Rozen (paratypes 2♀, AMNH), 09.ix.1991, J.G. and B.L. Rozen (paratype ♀, AMNH), 11.ix.1991, J.G. and B.L. Rozen (paratypes 1♀, 2♂, AMNH), 16.ix.2012, E.S. Wyman (paratypes 2♂, AMNH), 16.ix.2012, J.G. and M.A. Rozen (paratype ♀, AMNH), 26.viii.1994, J.G. Rozen and J.S. Ascher (paratypes 3♂, AMNH), 27.viii.2013, E.S. Wyman (allotype ♂ [CCDB-22791 A11], AMNH), 27.viii.2013, E.S. Wyman (paratypes 8♂, AMNH), 27.viii.2013, W.J. Cromartie (paratype ♂, AMNH), 27.viii.2013, G. Rowe (paratypes 7♂ (1 barcoded [CCDB-24580 G03]), PCYU), 28.viii.1985, J.G. and B.L. Rozen (paratypes 8♂, AMNH), 29.viii.2013, J.S. Ascher (paratypes 3♂, AMNH), 30.viii.1993, J.G. Rozen (paratypes 1♀, 9♂, AMNH); E Moore Ranch Rd (32.2391°N; 109.7722°W) (Willcox), 29.viii.2017, R. Oram (paratype ♀, RSKM); Phoenix (Maricopa County), 13.x.1997, K.C. Rozen (paratypes 3♂, AMNH); San Simon (Cochise County), 01.ix.1976, R.M. Bohart (paratype ♂, UCBME); SE Willcox (Cochise County), 30.ix.2016, L. Packer (paratype ♀, PCYU); Willcox (Cochise County), 02.ix.2003, J.G. Rozen, J.S. Ascher, R.L. Staff, and R.E. Edwards (paratypes 2♂, AMNH), 22.ix.1984, J.G. Rozen (paratype ♀, AMNH), 26.ix.1980, J.G. Rozen (paratypes 6♀, AMNH), 28.viii.1958, P.D. Hurd (paratype ♂, UCBME), 28–29.viii.1988, K.V. Krombein and B. Norden (paratype ♂, USNM); **New Mexico**: 5 mi E Laguna (Valencia County), 07.viii.1966, C.R. Kovacic (paratype ♀, UCBME); 20 mi N Animas (Hidalgo County), 05.ix.1981, R.M. Bohart (paratype ♀, UCBME); Mesilla Park, 17.ix.????, T.D. Cockerell (paratype ♀, USNM).

#### DNA barcoded material with BIN-compliant sequences.

Available. BOLD:ACR5356. See Type material for specimens examined and sequenced (indicated by unique CCDB-plate and well number).

### 12. Epeolus bifasciatus

Taxon classificationAnimaliaHymenopteraApidae

Cresson, 1864

Figs 26 , 27 , 91A

Epeolus bifasciatus Cresson, 1864a. Proc. Entomol. Soc. Phil. 3: 38 (♂); Cresson, 1916. Mem. Am. Entomol. Soc. 1: 113 (♂) [lectotype designation].

#### Diagnosis.

Unique to *E. bifasciatus* among North American species of *Epeolus* are each of the following morphological features: the frontal area bears a pair of granulose protrusions, each located near the upper mesal margin of the compound eye; the pronotal collar is elongate, dilated laterally to about 2 × the medial length in dorsal view; and the dorsum of the metasoma has at most two bright orange-yellow fasciae (usually a basal fascia on T1 and always an apical fascia on T2). Similar species occur in Mexico and Central America, but their occurrence in Canada and the United States has not been confirmed.

**Figure 26.**
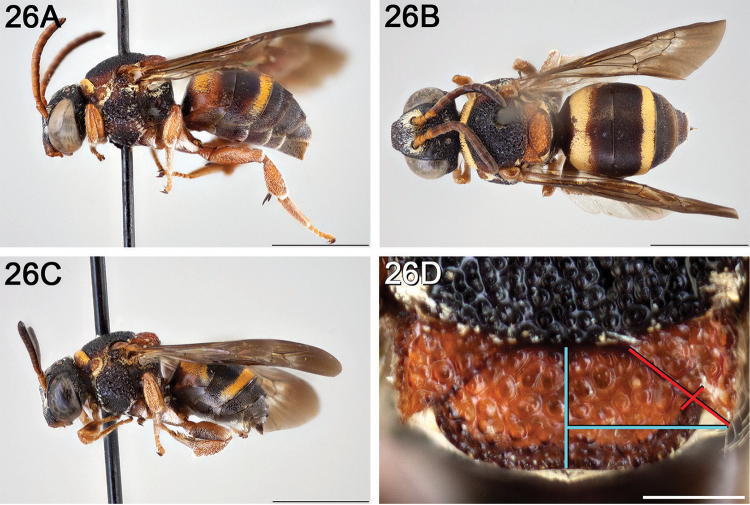
*Epeolus bifasciatus* **A** female, lateral habitus (scale bar 3 mm) **B** female, dorsal habitus (scale bar 3 mm) **C** male, lateral habitus (scale bar 3 mm), and **D** female axillae and mesoscutellum, dorsal view (scale bar 0.5 mm; blue lines indicate the posterior extent of the axilla relative to the length of the mesoscutellum; red lines indicate the extent of the free portion of the axilla relative to its entire medial length).

#### Redescription.

This species was recently redescribed (Onuferko 2017).

#### Distribution.

**Figure 27.**
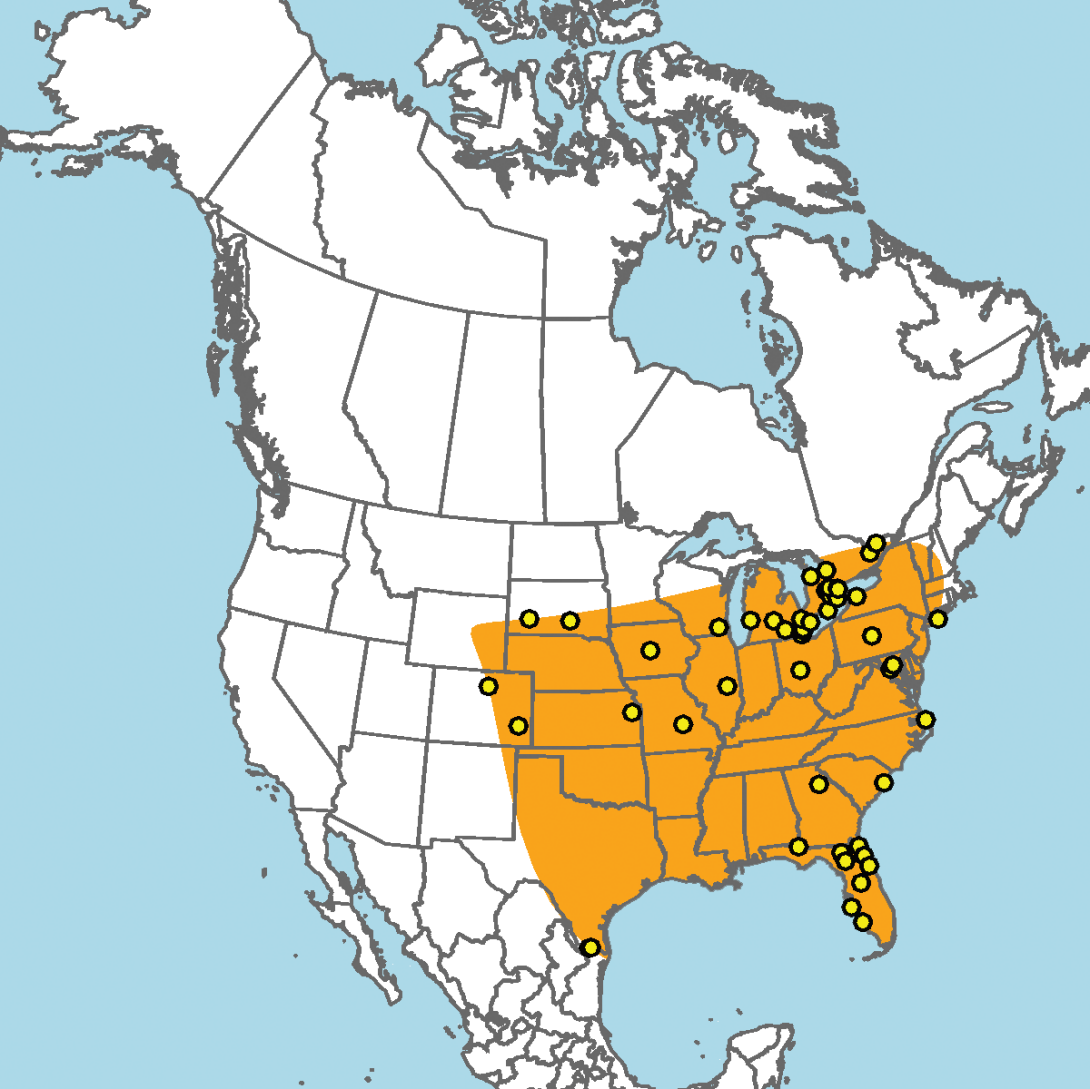
Approximate geographic range of *E. bifasciatus* (orange) based on occurrence records known to the author (yellow circles).

United States, east of the Continental Divide, into central Canada (Fig. 27).

#### Ecology.

See Onuferko (2017) for host and floral records. Floral associations are also indicated in Suppl. material 1, which includes newly discovered associations with *Coreopsis tinctoria* Nutt. (Compositae) and *Verbena hastata* L. (Verbenaceae) based on labels of examined voucher specimens.

#### Discussion.

*Epeolus bifasciatus* is the only species within the “Trophocleptria group” verified as occurring north of Mexico. Originally a genus, Trophocleptria Holmberg was later considered a subgenus of Epeolus (Michener 2000). Although its constituent species seem to form a natural group, a phylogenetic study by Rightmyer (2004) found that maintaining the subgeneric designation rendered Epeolus (Epeolus) paraphyletic, so Michener (2007) treated *Trophocleptria* as a distinct species group within *Epeolus*.

*Epeolus fumipennis* Say has been listed as occurring in Kansas (Snow 1879, in which E.T. Cresson was acknowledged for aiding in identification), but was probably confused with *E. bifasciatus*, a species that is common in that state (Ascher and Pickering 2017). Brumley (1965) examined specimens at the ANSP and KUNHM from the Midwestern and Southeastern United States labelled as *E. fumipennis* that according to him were clearly *E. bifasciatus*. The primary type of *E. fumipennis* was probably destroyed along with much of Thomas Say’s insect collection (LeConte 1859:v–vi, xix [footnote]), but the medially-narrowed ferruginous pronotal collar and yellow fasciae on T1 and T2 (contrasting with the whitish fasciae on the remaining terga), as well as its occurrence in Mexico, strongly suggest that this species is in the “Trophocleptria group”. However, in *E. fumipennis* the mesoscutum has distinct paramedian bands, which are absent in *E. bifasciatus*, and no specimens from Canada or the United States fitting such a description were seen.

#### Material studied.

**Type material.** Primary: USA: **Illinois**: (lectotype ♂ [ANSP, catalog number: 2658]).

#### DNA barcoded material with BIN-compliant sequences.

Available. BOLD:ADD5310. Specimens examined and sequenced.-Canada: **Ontario**: 1♀, 1♂ (PCYU).

USA: **Florida**: 1♂ (FSCA).

#### Non-barcoded material examined.

Canada: **Ontario**: 5♀, 6♂ (CNC, DEBU, PCYU, ROM); 2 km N Shiloh (43.7400°N; 80.2675°W) (Wellington County), 08.viii.2004, M. Buck (4♀, DEBU); 6 km NW Saint Williams (42.7050°N; 80.4606°W) (Hard.Norfolk Reg., Manestar Tract), 14.vii.2006, S.M. Paiero (5♀, 1♂, DEBU); Rondeau Park (South Point Trail, Kent County), 29.vi.2002, M. Buck (4♀, 1♂, DEBU); Toronto, 04.viii.2005, A. Cosens (1♂, PCYU).

USA: **Colorado**: Hasty (Bent County), 03.vii.1975, H.E. Evans (1♂, CUM); Longmont (40.1627°N; 105.1441°W) (Boulder County), 17.viii.2012, V. Scott (1♂, CUM); **Florida**: 2♂ (AMNH, PCYU); Caverns State Park (Jackson County), 16.vi.1999, C. Porter and L. Stange (1♀, FSCA); Lake City (Columbia County), 23.vi.2011, S. Lenberger (1♂, FSCA); Lovers Key State Rec Area (Lee County), 12.v.2008, C. Porter and L. Stange (1♀, FSCA); San Felasco Hammock Preserve State Park (Alachua County), 09–12.v.1979, G.B. Fairchild (1♀, FSCA); St Augustine Beach (St. Johns County), 24.v.1992, F.J. Santana (1♂, FSCA); **Georgia**: Athens (Whitehall Preserve, Clarke County), 14–19.v.1979, R.H. Turnbow, Jr. (1♂, FSCA); **Illinois**: 2♀ (AMNH); **Iowa**: Ames, 18.viii.1934, H.A. Scullen (1♀, CUM); **Kansas**: Baldwin, vii.????, J.C. Bridwell (1♀, CUM); **Maryland**: 2♀ (AMNH, BIML); **Michigan**: 5 km N West Olive (42.9884°N; 86.1423°W) (Ottawa County), 24.viii.2014, J. Gibbs (1♀, JBWM); East Lansing (42.7540°N; 84.4860°W) (Ingham County), 25.viii.2013, J. Gibbs (1♂, JBWM); Near Saline, 26.vi.1954, U.N. Lanham (1♂, CUM); **Missouri**: Rolla (Phelps County), 26.viii.1962, B. Vogel (2♀, CUM); **New York**: 1♂ (BIML); **North Carolina**: 1♂ (AMNH); **Ohio**: West Jefferson, G. Salt (2♀, NHMUK); **Pennsylvania**: 1♂ (BIML); **South Carolina**: 1♂ (DEBU); **South Dakota**: Oacoma (1 km W Chamberlain, Lyman County), 08.viii.2005, R.E. Wrigley (1♀, JBWM); **Texas**: Bentsen-Rio Grande Valley State Park, 01–13.vi.1976, C.C. Porter (1♂, FSCA); McAllen Botanical Gardens (McAllen), 03.vi.1976, C.C. Porter (1♂, FSCA); **Wisconsin**: 1♀ (PCYU).

### 13. Epeolus brumleyi sp. n.

Taxon classificationAnimaliaHymenopteraApidae

http://zoobank.org/9F7DC649-2303-414C-89B2-2333E3215DF0

**Figure 28.**
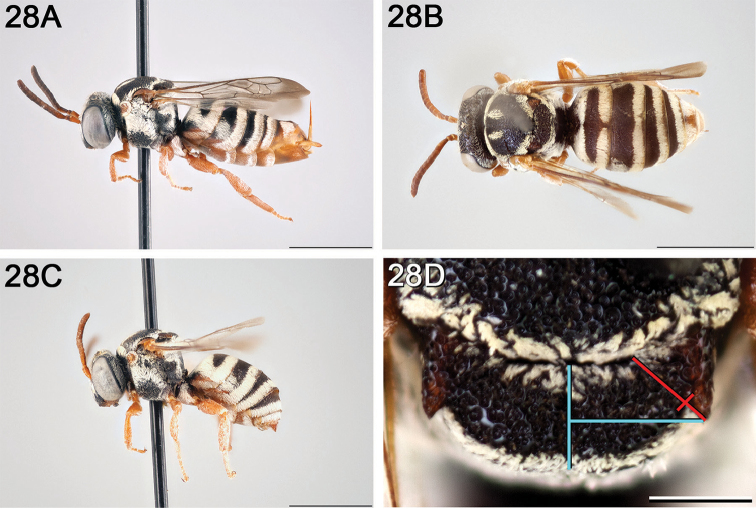
*Epeolus brumleyi* **A** female paratype, lateral habitus (scale bar 3 mm) **B** female holotype, dorsal habitus (scale bar 3 mm) **C** male paratype, lateral habitus (scale bar 3 mm), and **D** female paratype axillae and mesoscutellum, dorsal view (scale bar 0.5 mm; blue lines indicate the posterior extent of the axilla relative to the length of the mesoscutellum; red lines indicate the extent of the free portion of the axilla relative to its entire medial length).

**Figure 29.**
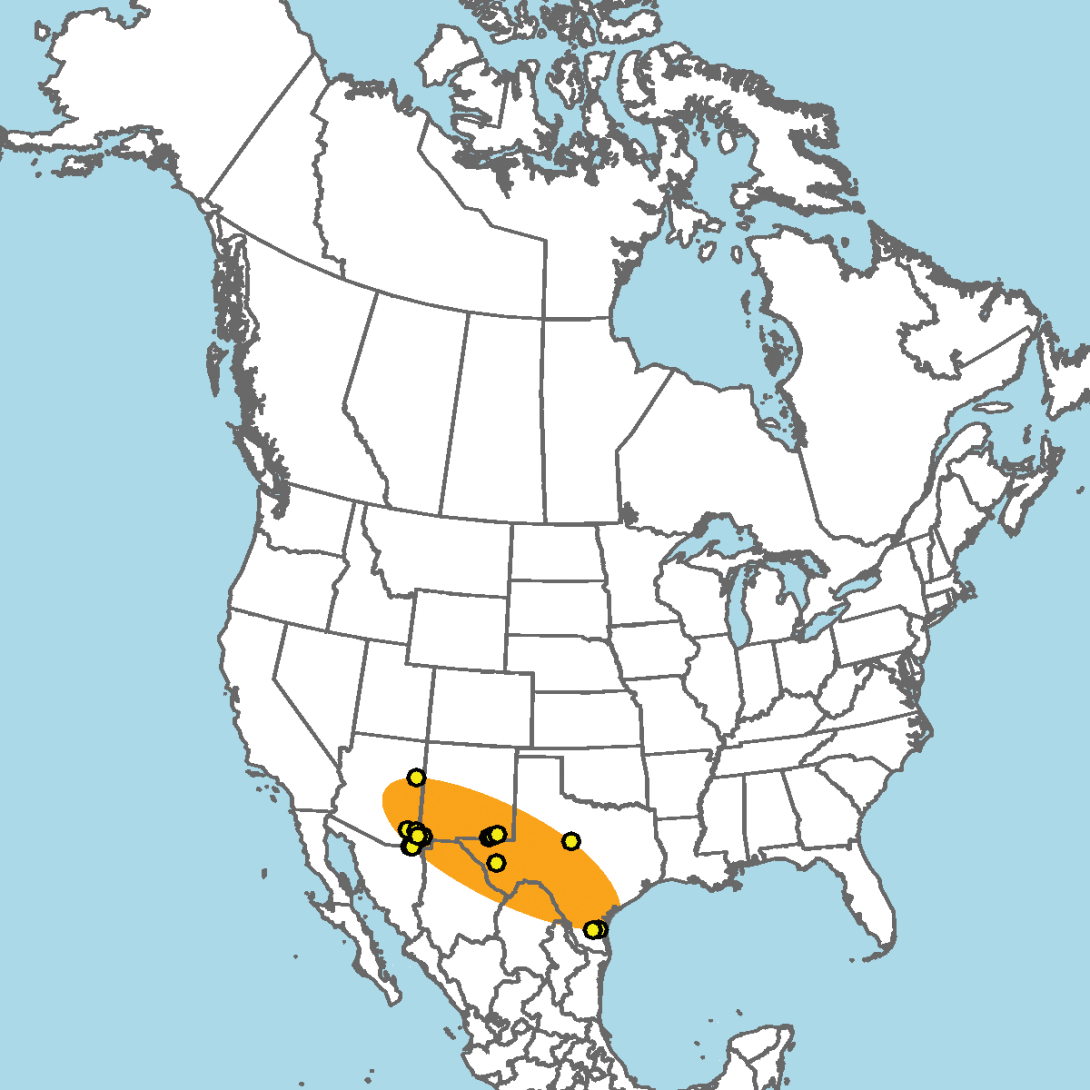
Approximate geographic range of *E. brumleyi* (orange) based on occurrence records known to the author (yellow circles).

Figs 2B , 28 , 29 , 103B

Epeolus brevicornus Brumley, 1965. M.S. thesis, Utah State University, Logan 38 (♀) [*nomen nudum*].

#### Diagnosis.

The following morphological features in combination can be used to tell *E. brumleyi* apart from all other North American *Epeolus*: the frontal carina is weakly convex, such that the supraclypeal area is barely protuberant in lateral view; the mesoscutum has distinct paramedian bands; the axilla is small to intermediate in size, not extending much beyond the midlength of the mesoscutellum (extending to <2/3 its length) but the free portion is at least 1/4 as long as (and less than 2/5) the entire medial length of the axilla, relatively straight along the medial margin, and ferruginous to some degree whereas the mesoscutellum is typically all black; the fore wing has three submarginal cells; the T1 basal and apical fasciae are subparallel; T2–T4 have complete fasciae; and the T2 fascia has a pair of anterolateral extensions of tomentum that are weakly convergent basally. *Epeolus brumleyi* most closely resembles *E. australis*, but in *E. australis* the frontal carina is strongly convex and the pygidial plate of the male is narrower (the medial length is ~1.5 × the basal width) than in *E. brumleyi* (the medial length ≈ the basal width).

#### Description.

FEMALE: Length 7.6 mm; head length 1.9 mm; head width 2.7 mm; fore wing length 5.8 mm.

*Integument coloration.* Mostly black; notable exceptions as follows: partially to entirely ferruginous on mandible, labrum, antenna, pronotal lobe, tegula, axilla, legs, metasomal terga (including pygidial plate), and metasomal sterna. Mandible with apex darker than rest of mandible; preapical tooth slightly lighter than mandibular apex (difficult to see in holotype because mandible closed; described from paratypes). Antenna brown and orange in part. Pronotal lobe and tegula pale ferruginous to amber. Wing membrane subhyaline, apically dusky. Legs more extensively reddish orange than brown or black.

*Pubescence.* Face with tomentum densest around antennal socket. Clypeus, upper paraocular and frontal areas, and vertexal area mostly exposed. Dorsum of mesosoma and metasoma with bands of off-white to pale yellow short appressed setae. Mesoscutum with paramedian band. Mesopleuron densely hairy, except for two almost entirely bare patches (one beneath base of fore wing (hypoepimeral area), a larger circular patch occupying much of ventrolateral half of mesopleuron). Metanotum with tomentum rubbed off medially in holotype, but uninterrupted and uniformly off white in paratypes. T1 with discal patch elliptical and very wide, the basal and apical fasciae only narrowly joined laterally. T1 with basal fascia complete and apical fascia interrupted medially, T2–T4 with fasciae complete, T2 with fascia with anterolateral extensions of sparser tomentum. T5 with two large patches of pale tomentum lateral to and contacting pseudopygidial area. T5 with pseudopygidial area lunate, its apex more than twice as wide as medial length, indicated by silvery setae on impressed disc of apicomedial region elevated from rest of tergum. S5 with apical fimbria of coppery to silvery hairs not extending beyond apex of sternum by much more than 1/4 MOD.

*Surface sculpture.* Punctures dense. Labrum with areas of sparser punctures (i=1–2d) than clypeus (i<1d). Small impunctate shiny spot lateral to lateral ocellus. Mesoscutum, mesoscutellum, and axilla coarsely and densely rugose-punctate. Tegula densely punctate mesally (i≤1d), less so laterally (i=1–2d). Mesopleuron with ventrolateral half densely punctate (i≤1d) to rugose, the interspaces shining; mesopleuron with punctures more or less equally dense throughout. Metasomal terga with punctures very fine, dense (i≈1d), evenly distributed on disc.

*Structure.* Preapical tooth blunt and obtuse. Labrum with submedial pair of small denticles, apex edentate. Frontal keel not strongly raised. Scape with greatest length 1.8 × greatest width. F2 as long as wide (L/W ratio = 1.0). Preoccipital ridge not joining hypostomal carina, from which it is separated by no less than 1 MOD at its terminal (difficult to see in holotype; described from paratypes). Mesoscutellum moderately bigibbous. Axilla small to intermediate in size, its lateral margin (L) less than half as long as mesoscutellar width (W) (L/W ratio = 0.4) and tip not extending beyond midlength of mesoscutellum; axilla with tip visible, but unattached to mesoscutellum for less than 2/5 the medial length of axilla; axilla with lateral margin relatively straight and without carina. Fore wing with three submarginal cells. Pygidial plate apically truncate.

MALE: Description as for female except for usual secondary sexual characters and as follows: F2 shorter, nearly as long as wide (L/W ratio = 0.9); S4 and S5 with much longer coppery to silvery subapical hairs; pygidial plate apically rounded, with large deep punctures closely clustered.

#### Etymology.

This species is named after its discoverer, Richard L. Brumley, who recognized it and five other *Epeolus* formally described here (*E. axillaris*, *E. chamaesarachae*, *E. diadematus*, *E. splendidus*, and *E. tessieris*) as new species.

#### Distribution.

Arizona to Texas and presumably Mexico, given the close proximity of some collection localities (e.g., Douglas, Arizona) to the Mexico–United States border (Fig. 29).

#### Ecology.

HOST RECORDS: Four representatives of this species were collected at a single site in southeast Arizona in the spring of 2016 (see Material studied), from or flying near patches of *Chamaesaracha* (A. Gray) Benth. (Solanaceae), which were visited by large numbers of *Colletes* (presumably the host species). Using Stephen’s (1954) key, collected females were identified as *C. scopiventer* Swenk (a species known only from females) whereas males were identified (based in part on examination of the terminalia, which were excised) as *C. wickhami* Timberlake (a species known only from males), and sequenced specimens of both sexes were assigned the same BIN (BOLD:AAJ7578).

FLORAL RECORDS: Labels of examined voucher specimens indicate floral associations with *Chamaesaracha coniodes* (Moric. ex Dunal) Britton and *Physalis* L. (Solanaceae).

#### Discussion.

*Epeolus brumleyi* is a southwestern species that exhibits very little intraspecific morphological variation. Adults have been collected in every month from March to September, and barcoded specimens collected in early May, June, and late August were assigned the same BIN.

#### Material studied.

**Type material.** Primary: USA: **Texas**: Davis Mountains, 10.vii.1942, E.C. Van Dyke (holotype ♀, CAS).

Secondary: USA: **Arizona**: 1 mi E Douglas (Cochise County), 08.v.1989, J.G. Rozen (paratype ♀ [CCDB-28315 G10], AMNH); 14 mi SW Apache (Cochise County), 14.v.1988, J.G. Rozen (paratype ♀, AMNH); 3 mi NE Portal (Cochise County), 18.viii.1970, J.G. Rozen (paratype ♂, AMNH); 3–7 mi S San Simon (Cochise County), 21.v.1988, J.G. Rozen (paratype ♀, AMNH); 9 mi E Douglas (Cochise County), 17.ix.1976, J.G. Rozen (paratype ♂, AMNH); Hwy 80 (31.4450°N; 109.4722°W) (~8 mi NE Douglas, Cochise County), 10.v.2016, T.M. Onuferko (allotype ♂, PCYU), 10.v.2016, T.M. Onuferko (paratypes 2♀ (1 barcoded [CCDB-24580 B11]), 1♂, PCYU); S Blue Sky Road (4 mi E Willcox, Cochise County), 30.viii.2015, J.S. Francis (paratype ♂ [CCDB-28238 A04], PCYU); **New Mexico**: 0.7 km E Longview Spring (32.1007°N; 104.6137°W) (Eddy County), 22.vi.2010, A. Druk and J.D. Herndon (paratype ♀, BBSL); 1 mi W Animas (Hidalgo County), 30.viii.1977, R.W. Brooks (paratype ♀, KUNHM); 1.1 km SW by W Oak Spring (32.1743°N; 104.4580°W) (Eddy County), 11.viii.2010, J.D. Herndon (paratype ♀, BBSL); 4 mi S Animas (Hidalgo County), 24.viii.1974, Rozen and Favreau (paratype ♂, AMNH); Loving (Eddy County), 28.v.1945, J.W. MacSwain (paratype ♂, BBSL); Walnut Canyon (32.1872°N; 104.3936°W) (2.6 km SE by S Cottonwood Spring, Eddy County), 03.vi.2010, A. Druk and J.D. Herndon (paratype ♀, BBSL); **Texas**: 18 km N Coleman (Coleman County), 01.vi.1989, B.N. Danforth (paratype ♀ [CCDB-28315 C09], KUNHM); 2 mi S Falfurrias (Brooks County), 13.iii.1999, J.L. Neff, A. Hook, and C. R. Riley (paratype ♂, CTMI); Davis Mountains, 28.vi.1942, E.C. Van Dyke (paratype ♂, BBSL), 17.iv.1954, R.H. Beamer (paratype ♂, BBSL); Sarita (Kenedy County), 15.iv.1976, J.E. Gillaspy (paratype ♀, BBSL).

#### DNA barcoded material with BIN-compliant sequences.

Available. BOLD:ACZ9234. See Type material for specimens examined and sequenced (indicated by unique CCDB-plate and well number).

### 14. Epeolus canadensis

Taxon classificationAnimaliaHymenopteraApidae

Mitchell, 1962

Figs 30 , 31 , 102B

Epeolus canadensis Mitchell, 1962. N. C. Agric. Exp. Stn. Tech. Bull. 152: 444 (♀).

#### Diagnosis.

The following morphological features in combination (excluding any that are specific to the opposite sex of the one being diagnosed) can be used to tell *E. canadensis* apart from all other North American *Epeolus* except *E. compactus* and *E. ferrarii*: in females, F2 is at least 1.2 × as long as wide; the mesoscutum has a small anteromedial patch of pale tomentum; the axilla is small to intermediate in size, not extending much beyond the midlength of the mesoscutellum (extending to <2/3 its length) but the free portion is more than 1/4 as long as the entire medial length of the axilla, and the axilla (except sometimes the tip) and mesoscutellum are black; the mesopleuron is closely (most i<1d) and evenly punctate; and the T2 fascia lacks lobe-like anterolateral extensions of tomentum, although it may be broader laterally. *Epeolus canadensis* differs from *E. compactus* and *E. ferrarii* in the shape of the T1 discal patch, which in *E. canadensis* is distinctly triangular or semicircular (the basal fascia is conspicuously arched and fully continuous with the longitudinal band) and its medial longitudinal extent is more than 1/3 the lateral extent. In *E. compactus* and *E. ferrarii* the shape of the T1 discal patch is variable but typically quadrangular with the basal and apical fasciae subparallel and separated by a distinct longitudinal band. In *E. compactus*, the medially-interrupted T1 basal and apical fasciae may be so broad laterally that they are joined, resulting in a diamond shape with concave sides. In *E. ferrarii* the discal patch may be trapezoidal or almost semicircular, but if at all semicircular its medial longitudinal extent is at most 1/3 the lateral extent and the basal fascia and longitudinal band are at least joined at somewhat of an angle.

**Figure 30.**
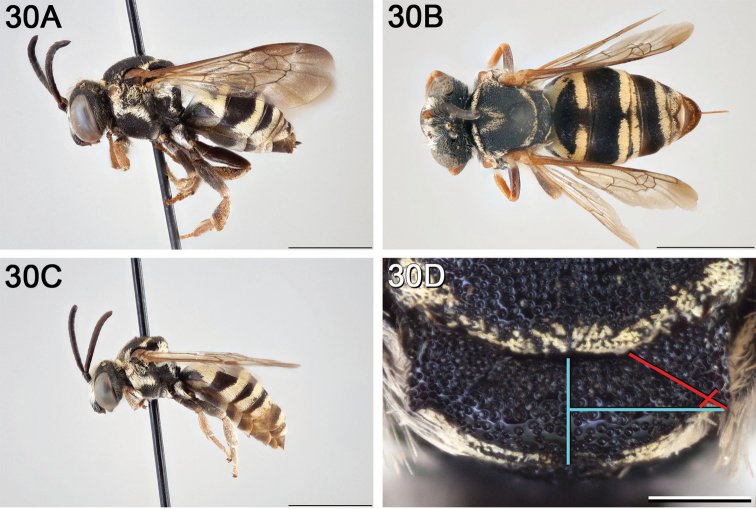
*Epeolus canadensis* **A** female, lateral habitus (scale bar 3 mm) **B** female holotype, dorsal habitus (scale bar 3 mm) **C** male, lateral habitus (scale bar 3 mm), and **D** female axillae and mesoscutellum, dorsal view (scale bar 0.5 mm; blue lines indicate the posterior extent of the axilla relative to the length of the mesoscutellum; red lines indicate the extent of the free portion of the axilla relative to its entire medial length).

#### Redescription.

This species was recently redescribed (Onuferko 2017).

#### Distribution.

**Figure 31.**
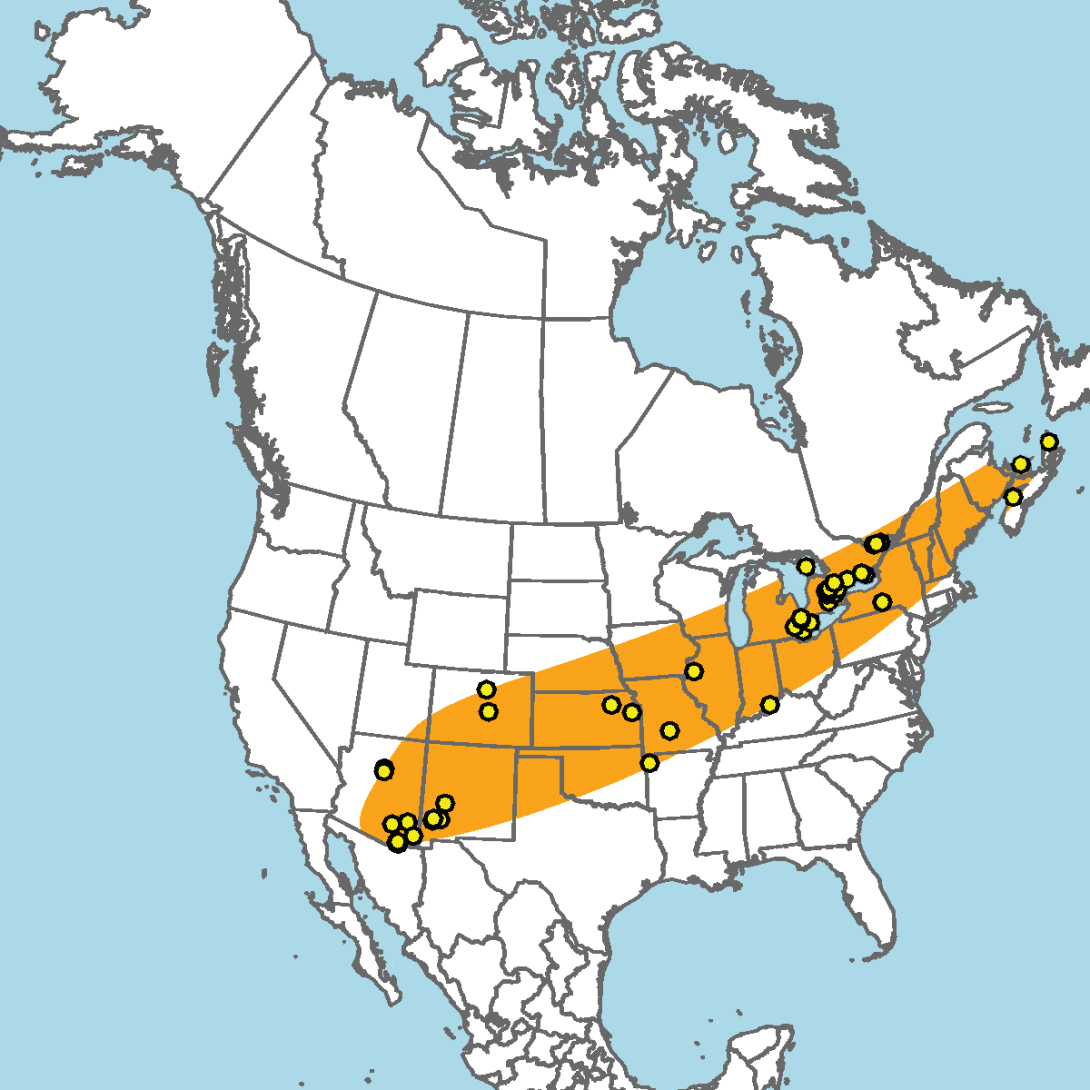
Approximate geographic range of *E. canadensis* (orange) based on occurrence records known to the author (yellow circles).

Atlantic Canada to southwestern United States (Fig. 31).

#### Ecology.

HOST RECORDS: An association between *Colletes kincaidii* Cockerell and *E. canadensis* hypothesized earlier (Onuferko 2017) seems more likely now based on new knowledge that the two species have been collected in co-occurrence near Six Mile Creek (Ithaca), New York, USA (J. Ascher, personal communication, 2017) and personal collections of the two species in early July, 2017 on the side of a road in Navan (east of Ottawa), Ontario, Canada. *Colletes kincaidii* females and males were collected from staghorn sumac (*Rhus typhina* L. (Anacardiaceae)) on the same dates *E. canadensis* were collected from daisy-like flowers (Compositae) closer to the ground.

FLORAL RECORDS: See Onuferko (2017) for floral records. Floral associations are also indicated in Suppl. material 1, which includes a newly discovered association with *Grindelia* Willd. (Compositae) based on the label of one examined voucher specimen.

#### Discussion.

Detailed morphological and taxonomic remarks about this species are given in Onuferko (2017).

#### Material studied.

**Type material.** Primary: Canada: **Nova Scotia**: Ingonish (Cape Breton Island), 07.viii.1928, G. Fairchild (holotype ♀ [MCZ, catalog number: 32859]).

Secondary: USA: **New York**: 9-Mile Creek (Ithaca), 10.vii.1937, P.P. Babiy (allotype ♂ [CUIC, catalog number: 00015611]).

#### DNA barcoded material with BIN-compliant sequences.

Available. BOLD:ADA0845. Specimens examined and sequenced.–Canada: **Ontario**: 1♀, 1♂ (DEBU); Navan (45.3982°N; 75.3623°W) (Caroltodd Dr & Whispering Willow Dr), 02.vii.2017, T.M. Onuferko (1♂, PCYU), 03.vii.2017, T.M. Onuferko (1♀, PCYU).

USA: **Arizona**: 1♂ (PCYU); Flagstaff (35.1737°N; 111.6756°W) (Coconino County), 01–03.vi.2017, T.M. Onuferko (1♀, PCYU); **New Mexico**: 2♂ (DEBU, PCYU).

#### Non-barcoded material examined.

Canada: **Nova Scotia**: 3♀, 4♂ (CNC); **Ontario**: 10♀, 15♂ (CNC, DEBU, PCYU, ROM); Forks of the Credit Provincial Park, vii.2002?, J. Grixti (1♂, PCYU); **Prince Edward Island**: 1♀ (CNC); **Quebec**: 3♀ (CNC).

USA: **Arizona**: 5♀, 3♂ (AMNH, CNC, PCYU); Flagstaff (35.1737°N; 111.6756°W) (Coconino County), 01–03.vi.2017, T.M. Onuferko (1♀, PCYU); Huachuca Mountains, 14.ix.1938, R.H. Crandall (1♀, 1♂, LACM); Santa Catalina Mountains (Pima County), J.L. Neff (1♂, LACM); **Arkansas**: 1♀ (FSCA); **Colorado**: Boulder (Boulder County), 12.ix.1965, U.N. Lanham (1♀, CUM); **Illinois**: 1♀ (KUNHM); **Kansas**: 2♀ (KUNHM); **Missouri**: 1♀ (KUNHM); **New Mexico**: 5♀, 5♂ (AMNH, BBSL, CNC).

### 15. Epeolus carolinus

Taxon classificationAnimaliaHymenopteraApidae

Mitchell, 1962

**Figure 32.**
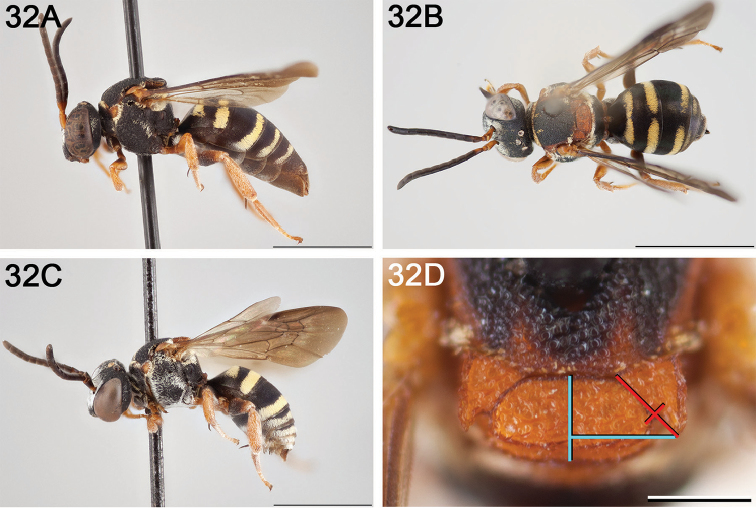
*Epeolus carolinus* **A** female, lateral habitus (scale bar 3 mm) **B** female, dorsal habitus (scale bar 3 mm) **C** male, lateral habitus (scale bar 3 mm), and **D** female axillae and mesoscutellum, dorsal view (scale bar 0.5 mm; blue lines indicate the posterior extent of the axilla relative to the length of the mesoscutellum; red lines indicate the extent of the free portion of the axilla relative to its entire medial length).

**Figure 33.**
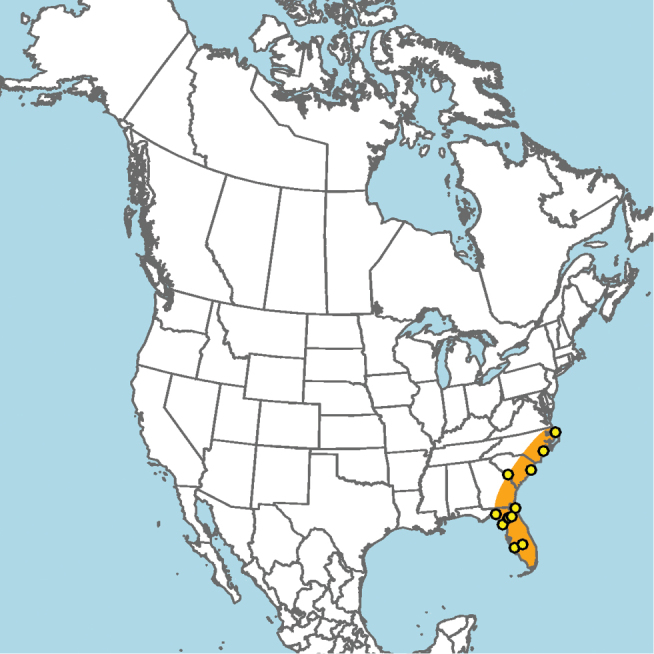
Approximate geographic range of *E. carolinus* (orange) based on occurrence records known to the author (yellow circles).

Figs 3C , 32 , 33 , 92B

Epeolus carolinus Mitchell, 1962. N. C. Agric. Exp. Stn. Tech. Bull. 152: 445 (♂).

#### Diagnosis.

The following morphological features in combination can be used to tell *E. carolinus* apart from all other North American *Epeolus*: the mandible has a blunt, obtuse preapical tooth; the axilla is elongate, extending well beyond the midlength of the mesoscutellum but not beyond its posterior margin, and the free portion is distinctly hooked; the mesopleuron is closely (most i<1d) and evenly punctate; and the metasomal fasciae are yellow to orange and interrupted medially. *Epeolus carolinus* resembles *E. deyrupi* in general appearance, but in *E. deyrupi* the axilla is larger, extending as far back as or beyond the posterior margin of the mesoscutellum, and dilated laterally but relatively straight along the medial margin, and the mesopleuron commonly has sparser punctures ventrolaterally (i≤2d) than that of *E. carolinus*, with the interspaces shining or somewhat dull due to tessellate surface microsculpture.

#### Redescription.

MALE: Length 6.5 mm; head length 1.8 mm; head width 2.4 mm; fore wing length 5.7 mm.

*Integument coloration*. Mostly black; notable exceptions as follows: partially to entirely ferruginous on mandible, antenna, pronotal lobe, tegula, axilla, mesoscutum, mesoscutellum, legs, and pygidial plate. Mandible with apex darker than rest of mandible; preapical tooth slightly lighter than mandibular apex (difficult to see in holotype; described from paratype). Antenna brown except scape, pedicel, and F1 extensively orange. Pronotal lobe and tegula pale ferruginous to amber. Mesoscutum with orange spot anterolaterally between pronotal lobe and tegula. Wing membrane subhyaline, apically dusky. Legs more extensively reddish orange than brown or black.

*Pubescence*. Face with tomentum densest around antennal socket. Tomentum slightly sparser on clypeus; upper paraocular and frontal areas, and vertexal area mostly exposed. Dorsum of mesosoma and metasoma with bands of off-white and yellow short appressed setae. Mesoscutum with paramedian band. Mesopleuron densely hairy, except for two sparsely hairy circular patches (one behind pronotal lobe, a larger one occupying much of ventrolateral half of mesopleuron). Metanotum with tomentum sparser medially, uniformly off white. T1 with discal patch quadrangular and very wide, the basal and apical fasciae only narrowly joined laterally by few sparsely scattered pale hairs (not joined in paratype and multiple non-type specimens). T1–T5 with apical fasciae interrupted medially, those of T2–T4 somewhat broader laterally, T2 with fascia without anterolateral extensions of tomentum. T6 with fascia complete. S4 and S5 with long coppery to silvery subapical hairs.

*Surface sculpture*. Punctures dense. Labrum with larger punctures than clypeus, but punctures of both equally dense (i<1d). Impunctate spot lateral to lateral ocellus absent. Mesoscutum, mesoscutellum, and axilla coarsely and densely rugose-punctate. Tegula very densely punctate mesally (i<1d), much less so laterally (i>2d). Mesopleuron with ventrolateral half densely punctate (i<1d) to rugose; mesopleuron with punctures more or less equally dense throughout. Metasomal terga with punctures very fine, dense (i≈1d), evenly distributed on disc.

*Structure*. Preapical tooth inconspicuous, blunt and obtuse. Labrum with pair of small subapical denticles not preceded by carinae. Frontal keel not strongly raised. Scape with greatest length 1.8 × greatest width. F2 noticeably longer than wide (L/W ratio = 1.4). Preoccipital ridge not joining hypostomal carina, from which it is separated by less than 1 MOD at its terminal (difficult to see in holotype; described from non-type specimens). Mesoscutellum weakly bigibbous. Axilla large, its lateral margin (L) more than half as long as mesoscutellar width (W) (L/W ratio = 0.6) and tip extending well beyond midlength of mesoscutellum but not as far back as its posterior margin; axilla with tip conspicuously diverging from side of mesoscutellum, distinctly hooked, and axilla with free portion 2/5 its medial length; axilla with lateral margin arcuate and carinate. Fore wing with three submarginal cells. Pygidial plate apically rounded, with large deep punctures more or less evenly spaced throughout, with the interspaces shining.

FEMALE: Description as for male except for usual secondary sexual characters and as follows: F2 even longer than wide (L/W ratio = 1.7); T5 with pseudopygidial area lunate, its apex more than twice as wide as medial length, indicated by silvery setae on flat disc of apicomedial region elevated from rest of tergum; S4 and S5 with much shorter hairs (S5 with apical fimbria of coppery to silvery hairs not extending beyond apex of sternum by more than 1/4 MOD); pygidial plate apically truncate, with small, denser punctures.

#### Distribution.

South Atlantic states (Fig. 33).

#### Ecology.

HOST RECORDS: The host species of *E. carolinus* is/are presently unknown.

FLORAL RECORDS: Mitchell (1962) indicated a floral association with *Eupatorium* L. (Compositae), and BugGuide (http://www.bugguide.net/) indicates an association with *Solidago fistulosa* Mill. Labels of examined voucher specimens further indicate associations with *Euthamia graminifolia* (L.) Nutt. (Compositae), *Heterotheca subaxillaris* (Lam.) Britton & Rusby (Compositae), and *Spermacoce* L. (Rubiaceae).

#### Discussion.

This southeastern species is quite variable in terms of integument coloration and pubescence on the metasomal terga. The mesoscutellum and disc of T1 range from entirely black to entirely ferruginous. The axillae appear to be at least partially ferruginous. Whereas T1 and T2 have prominent yellow fasciae, the fasciae on the remaining terga range from prominent to reduced or even absent. Adults of *Epeolus carolinus* are active in September and October.

#### Material studied.

**Type material.** Primary: USA: **North Carolina**: Kill Devil Hills, 12.ix.1956, T.B. Mitchell (holotype ♂ [USNM, catalog number: 534042]).

Secondary: USA: **North Carolina**: Kill Devil Hills, 13.ix.1956, T.B. Mitchell (paratype ♂, NHMUK); New River, 20–30.ix.1944, G.E. Bohart (paratype ♂, BBSL).

#### DNA barcoded material with BIN-compliant sequences.

Available. BOLD:ACM5698. Specimens examined and sequenced.–USA: **Florida**: Timucuan Ecological & Historic Preserve (30.3842°N; 81.4857°W) (Duval County), 15.x.2012, C. Pontifet (1♂, BIML); **South Carolina**: Prince George Estates (E Hwy 17, Georgetown County), 09.x.2006, S. Paiero and S.A. Marshall (1♂, DEBU).

#### Non-barcoded material examined.

USA: **Florida**: Archbold Biological Station (Highlands County), 11.x.1978, H.V. Weems, Jr. and S.J. Chance (2♀, LACM), 08.x.1964, P.H. Arnaud, Jr. (1♂, LACM); Cedar Key (Levy County), 27.x.1974, E.E. Grissell (3♀, 1♂, UCBME); Doyle Conner Bldg (Gainesville, Alachua County), 04.x.1995, C. Porter (1♂, FSCA), 12.x.1995, C. Porter (1♂, FSCA), 17.x.1995, C. Porter (2♂, FSCA); Gainesville (Alachua County), 13.x.??48 (1♀, LACM), 25.viii.1976, W.H. Pierce (1♂, UCBME); Mason Road (Melrose, Putnam County), 11.x.2009, J.S. Ascher and H.G. Hall (1♂, AMNH); Perry (Taylor County), 1983, L. Packer (1♀, PCYU); W Murdock (Charlotte County), 20.x.1983, L. Packer (2♀, 2♂, PCYU); **South Carolina**: Aiken Savannah River Site (33.3449°N; 81.6614°W), 17.x.2016, S. Breland (1♀, JBWM); Prince George Estates (E Hwy 17, Georgetown County), 09.x.2006, S. Paiero and S.A. Marshall (1♂, DEBU).

### 16. Epeolus chamaesarachae sp. n.

Taxon classificationAnimaliaHymenopteraApidae

http://zoobank.org/DE654BDC-F47F-4ECE-82DB-5FE0946A0EE2

**Figure 34.**
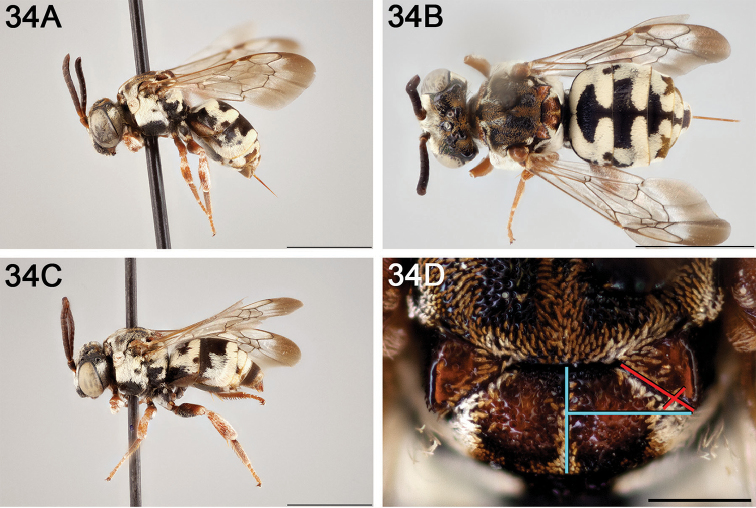
*Epeolus chamaesarachae* **A** female paratype, lateral habitus (scale bar 3 mm) **B** female holotype, dorsal habitus (scale bar 3 mm) **C** male paratype, lateral habitus (scale bar 3 mm), and **D** female paratype axillae and mesoscutellum, dorsal view (scale bar 0.5 mm; blue lines indicate the posterior extent of the axilla relative to the length of the mesoscutellum; red lines indicate the extent of the free portion of the axilla relative to its entire medial length).

**Figure 35.**
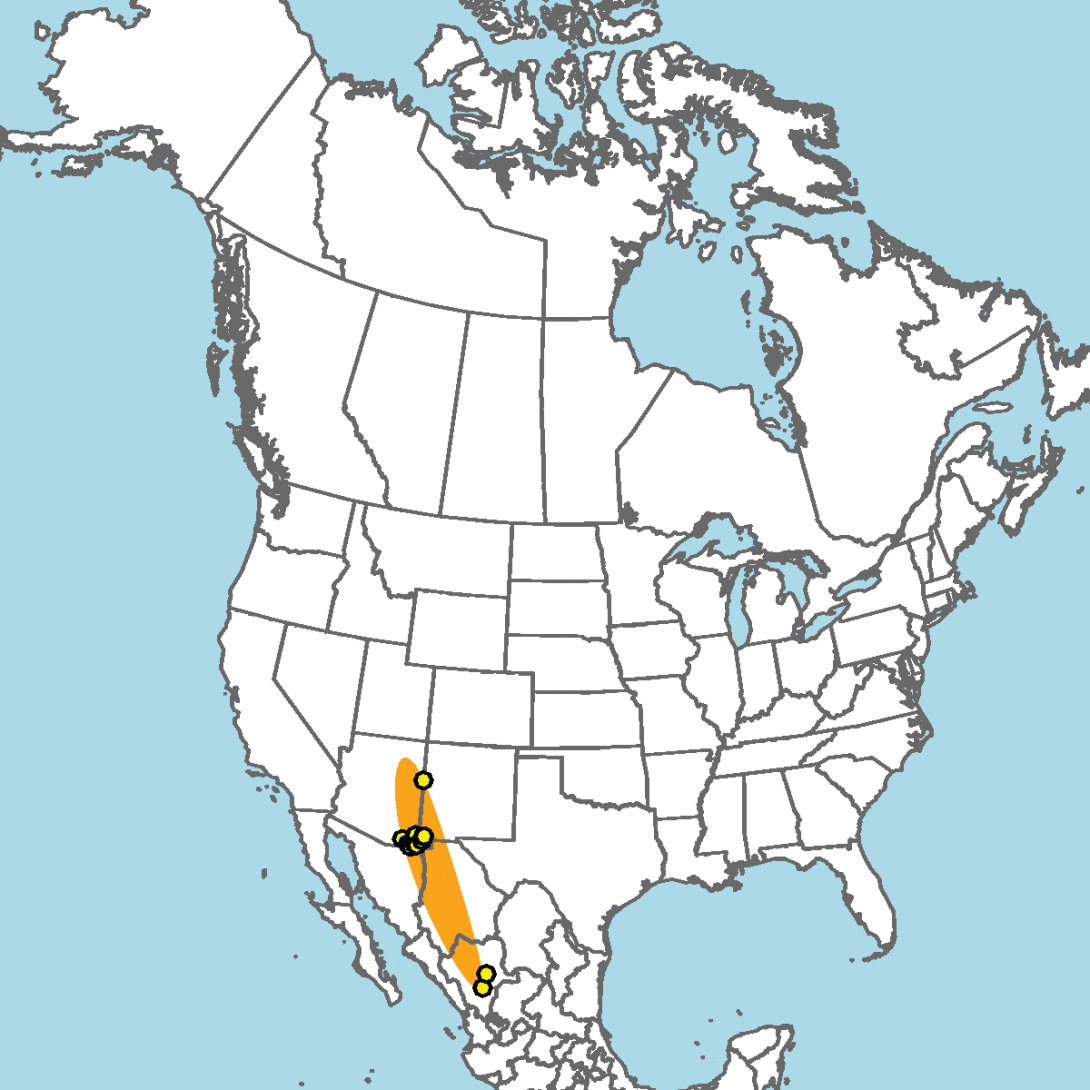
Approximate geographic range of *E. chamaesarachae* (orange) based on occurrence records known to the author (yellow circles).

Figs 1 , 34 , 35 , 91C , 92I

Epeolus lobus Brumley, 1965. M.S. thesis, Utah State University, Logan 51 (♀) [*nomen nudum*].

#### Diagnosis.

*Epeolus chamaesarachae* does not closely resemble any other species of *Epeolus* except *E. diadematus*. Unique in the genus to both species are each of the following morphological features: the vertexal area has two pairs of shiny (usually impunctate) protrusions, the mesoscutum is distinctly ornamented with mostly separate patches of (but some intermixed) pale and ferruginous tomentum, and the T2 fascia has two pairs of anterolateral extensions of tomentum. The difference is that in *E. chamaesarachae* the mesopleuron has sparser punctures ventrolaterally (most i>1d) whereas in *E. diadematus* the mesopleuron has denser (most i≤1d) and more numerous punctures ventrolaterally.

#### Description.

FEMALE: Length 7.0 mm; head length 2.0 mm; head width 2.6 mm; fore wing length 5.7 mm.

*Integument coloration.* Mostly black; notable exceptions as follows: partially to entirely ferruginous on mandible, antenna, pronotal collar, pronotal lobe, tegula, axilla, mesoscutellum, and legs. Mandible with apex darker than all but extreme base; preapical tooth lighter than mandibular apex (difficult to see in holotype; described from paratypes). Antenna dark brown except scape, pedicel, and F1 brownish orange in part. Pronotal lobe and tegula pale ferruginous to amber. Wing membrane subhyaline, apically dusky. Legs more extensively reddish orange than brown or black.

*Pubescence.* Face with tomentum densest around antennal socket. Vertexal area with tomentum mostly ferruginous. Dorsum of mesosoma with bands of off-white and ferruginous short appressed setae. Dorsum of metasoma with bands of off-white to pale yellow short appressed setae. Pronotal lobe entirely obscured by pale tomentum. Pronotal collar with tomentum black medially, pale and ferruginous laterally. Mesoscutum with paramedian band of pale tomentum; ferruginous and pale tomentum encircling black spots medially and laterally, respectively, on anterior margin; and ferruginous tomentum along medial mesoscutal line and parapsidal line. Mesopleuron with upper half densely hairy, although scrobe visible; ventrolateral half nearly bare. Metanotum with tomentum uninterrupted, off white laterally and black medially. T1 with median diamond-shaped black discal patch enclosed by pale tomentum, except for medial separation at apex. T1 with apical fascia with black spot posterolaterally. T2–T4 with fasciae interrupted medially, T2 with fascia with paired anterolateral extensions of tomentum. T3 and T4 with fasciae interrupted laterally, with medial portion on apical margin and lateral portion encircling black tomentum on apical margin. T5 with two large patches of pale tomentum lateral to and separate from pseudopygidial area. T5 with pseudopygidial area lunate, its apex more than twice as wide as medial length, indicated by silvery setae on disc of apicomedial region elevated from rest of tergum. S5 with apical fimbria of coppery to silvery hairs extending beyond apex of sternum by ~1/3 MOD.

*Surface sculpture.* Punctures dense, but those of head and mesosoma sparser in some areas, larger, deep, and distinct. Labrum with larger and sparser punctures (i=1–2d) than clypeus (i≤1d). Upper paraocular area and vertexal area with few punctures, the interspaces shining. Mesoscutum, mesoscutellum, and axilla coarsely and densely to sparsely punctate; the interspaces shining. Tegula densely punctate mesally (i≤1d), less so laterally (i=1–2d). Mesopleuron with denser (i≤1d) punctures in upper half than ventrolateral half, and ventrolateral half with most interspaces large (i>1d); the interspaces shining. Metasomal terga with punctures very fine, dense (i≈1d), evenly distributed on disc.

*Structure.* Labral apex with pair of small denticles (preceded by submedial pair of small denticles) separated by shallow concavity and between second pair of apical lobes. Frontal keel strongly raised. Vertexal area with two pairs of impunctate shiny protrusions. Scape with greatest length 1.6 × greatest width. F2 as long as wide (L/W ratio = 1.0). Preoccipital ridge not joining hypostomal carina, from which it is separated by about 1.5 MOD at its terminal. Mesoscutellum strongly bigibbous. Axilla intermediate in size, its lateral margin (L) nearly half as long as mesoscutellar width (W) (L/W ratio = 0.4–0.5) and tip not extending beyond midlength of mesoscutellum; axilla with tip conspicuously diverging from side of mesoscutellum, distinctly hooked, but unattached to mesoscutellum for less than 1/3 the medial length of axilla; axilla with lateral margin relatively straight and without carina. Fore wing with three submarginal cells. Pygidial plate apically truncate.

MALE: Description as for female except for usual secondary sexual characters and as follows: F2 shorter, nearly as long as wide (L/W ratio = 0.8); S4 and S5 with much longer coppery to silvery subapical hairs; pygidial plate apically rounded, with large deep punctures closely clustered apically and sparser basally, with the interspaces shining.

#### Etymology.

The name is in reference to the genus of flowers (*Chamaesaracha*) on which the holotype was collected.

#### Distribution.

Northwestern Mexico and southwestern United States (Fig. 35).

#### Ecology.

HOST RECORDS: The female PCYU paratype collected by H.T. Ngo (see Material studied) is labelled with the same collection information as three *Colletes* specimens (2♀, 1♂) of the presumed host species, which were barcoded and all share the same BIN (BOLD:AAJ7578). Using Stephen’s (1954) key, the two females were identified as *C. scopiventer* (a species known only from females) whereas the male was identified (based in part on examination of the terminalia, which were excised) as *C. wickhami* (a species known only from males).

FLORAL RECORDS: Labels of examined voucher specimens indicate floral associations with *Baccharis* L. (Compositae), *Chamaesaracha*, *Kallstroemia grandiflora* Torr. ex A. Gray (Zygophyllaceae), *Margaranthus solanaceous* Schltdl. (Solanaceae), *Sphaeralcea angustifolia* (Cav.) G. Don, and *Tidestromia lanuginosa* (Nutt.) Standl. (Amaranthaceae).

#### Discussion.

This species and the very similar *E. diadematus* are unusual among *Epeolus* in that the vertexal area has two pairs of shiny (usually impunctate) protrusions, and dorsally the mesosoma and metasoma have unique patterns of ferruginous (mesosoma only) and off-white to pale yellow short appressed setae. *Epeolus chamaesarachae* occurs in the Southwestern United States, and its flight season, based on material examined, is late summer.

#### Material studied.

**Type material.** Primary: USA: **Arizona**: Douglas Model Plane Airport (31.3433°N; 109.4980°W) (Cochise County), 24.viii.2010, T.L. Griswold (holotype ♀ [CCDB-28239 F07], BBSL).

Secondary: Mexico: **Durango**: Durango, 14.viii.1947, D. Rockefeller Exp. Michener (paratype ♂, AMNH); San Juan del Río, 30.vii.1947, D. Rockefeller Exp. Michener (paratype ♀, AMNH).

USA: **Arizona**: 1 mi E Douglas (Cochise County), 16.viii.1962, M. Statham (paratype ♂, AMNH), 27.viii.2007, H.T. Ngo (paratype ♀ [CCDB-22013 G05], PCYU); 1 mi E Douglas (31.3356°N; 109.4950°W) (Cochise County), 23.viii.2003, J.G. Rozen (paratype ♀, AMNH); 12 mi NW Douglas (Cochise County), 30.viii.1989, J.G. and B.L. Rozen and R. Foster (paratype ♀, AMNH); 14 mi SW Apache (Cochise County), 04.viii.1961, J.G. Rozen (paratype ♀, AMNH), 21.viii.2008, J.S. Ascher, J.G. Rozen, and M.A. Rozen (paratype ♂ [CCDB-22791 A09], AMNH); 25 mi SE Sanders (Apache County), 14.viii.1972, J.G. Rozen and R. McGinley (paratype ♂, AMNH); 8 mi NE Portal (Cochise County), 14.viii.1990, J.G. Rozen and J. Krieger (paratype ♀, AMNH); Douglas Model Plane Airport (31.3433°N; 109.4980°W) (Cochise County), 24.viii.2010, T.L. Griswold (allotype ♂ [CCDB-28239 F09], BBSL), 24.viii.2010, T.L. Griswold (paratype ♂, BBSL); Geronimo Trail at Sycamore Creek (31.4432°N; 109.1390°W) (Cochise County), 28.viii.2016, L. Packer (paratype ♀, PCYU); Tombstone (Cochise County), 17.viii.1975, J.G. Rozen (paratype ♀, AMNH); **New Mexico**: 16 mi S Animas (31.7211°N; 108.8224°W) (Hidalgo County), 03.ix.2011, J.G. Rozen and E.S. Wyman (paratype ♀ [CCDB-22791 A07], AMNH); 2.6 mi E Animas (31.9542°N; 108.7630°W) (NM Hwy 9, 2.6 mi E NM Hwy 338), 11.viii.1972, T.J. Zavortink (paratypes 1♀, 2♂, UCBME); 5.5 mi E Animas (31.9558°N; 108.7142°W) (Hidalgo County), 18–25.viii.2002, E. Elle (paratype ♂, AMNH).

#### DNA barcoded material with BIN-compliant sequences.

Available. BOLD:ACP9403. See Type material for specimens examined and sequenced (indicated by unique CCDB-plate and well number).

### 17. Epeolus compactus

Taxon classificationAnimaliaHymenopteraApidae

Cresson, 1878

**Figure 36.**
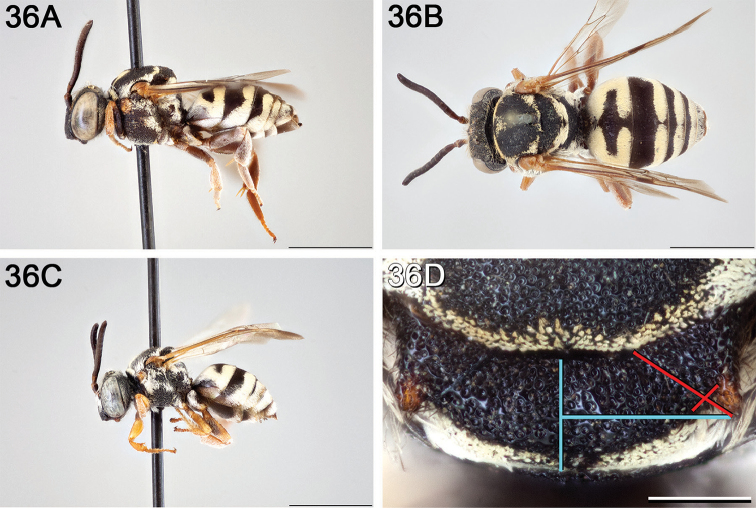
*Epeolus compactus* **A** female, lateral habitus (scale bar 3 mm) **B** female lectotype, dorsal habitus (scale bar 3 mm) **C** male, lateral habitus (scale bar 3 mm), and **D** female axillae and mesoscutellum, dorsal view (scale bar 0.5 mm; blue lines indicate the posterior extent of the axilla relative to the length of the mesoscutellum; red lines indicate the extent of the free portion of the axilla relative to its entire medial length).

**Figure 37.**
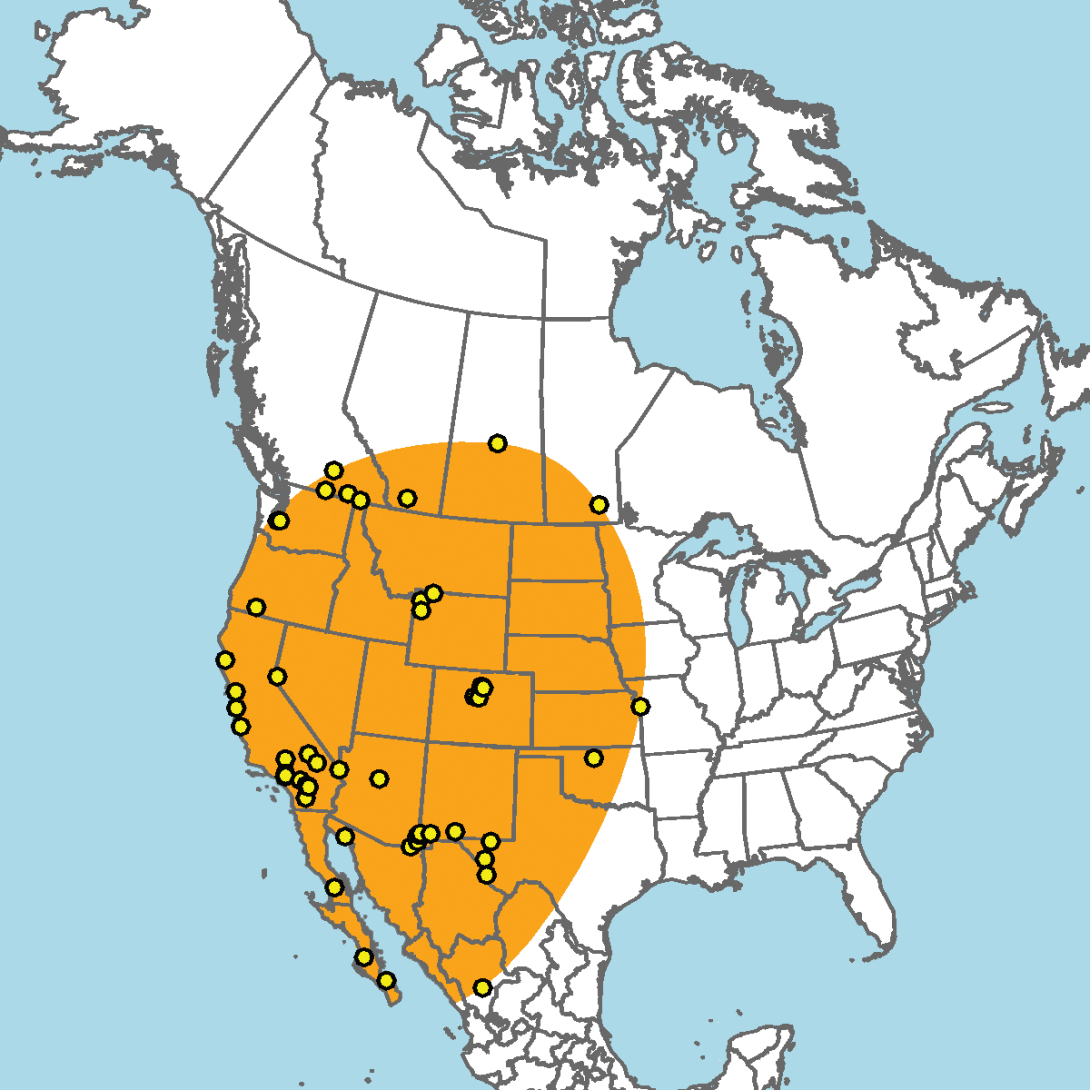
Approximate geographic range of *E. compactus* (orange) based on occurrence records known to the author (yellow circles).

**Figure 38.**
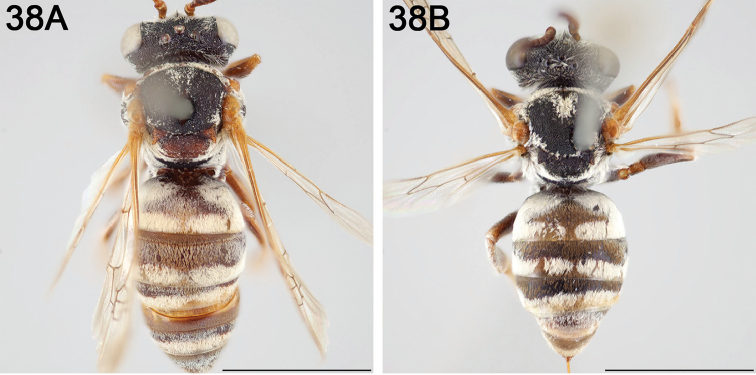
**A** *E. crucis* female holotype (herein synonymized under *E. compactus*), dorsal habitus, and **B** *E. compactus* typical female, dorsal habitus, in which the axilla, mesoscutellum, and discs of the metasomal terga (in terms of integument coloration and pubescence) are black or nearly black. Scale bars 3 mm.

Figs 3F , 36 , 37 , 38

Epeolus compactus Cresson, 1878. Trans. Am. Entomol. Soc. 7: 89 (♀, ♂); Cresson, 1916. Mem. Am. Entomol. Soc. 1: 115 (♀) [lectotype designation].
Epeolus crucis Cockerell, 1904. Ann. Mag. Nat. Hist. 13: 39 (♀), **syn. n.**
Epeolus hitei Cockerell, 1908. Entomologist 41: 60 (♀).
Triepeolus gabrielis Cockerell, 1909. Ann. Mag. Nat. Hist. 5: 26 (♂).
Epeolus geminatus Cockerell and Sandhouse, 1924. Proc. Calif. Acad. Sci. (4) 13: 315 (♀).

#### Diagnosis.

The following morphological features in combination (excluding any that are specific to the opposite sex of the one being diagnosed) can be used to tell *E. compactus* apart from all other North American *Epeolus* except *E. canadensis* and *E. ferrarii*: in females, F2 is at least 1.2 × as long as wide; the mesoscutum has a small anteromedial patch of pale tomentum; the axilla is small to intermediate in size, not extending much beyond the midlength of the mesoscutellum (extending to <2/3 its length) but the free portion is more than 1/4 as long as the entire medial length of the axilla, and the axilla (except sometimes the tip) and mesoscutellum are black; the mesopleuron is closely (most i<1d) and evenly punctate; and the T2 fascia lacks lobe-like anterolateral extensions of tomentum, although it may be broader laterally. *Epeolus compactus* is most similar to *E. ferrarii*, and in both species the T1 discal patch is typically quadrangular with the basal and apical fasciae subparallel and separated by a distinct longitudinal band, but in *E. ferrarii* the T2–T4 fasciae are not broadened medially into rounded lobes (as in *E. compactus*) but evenly broad or tapering until separated medially. *Epeolus canadensis* differs from both species in that the T1 discal patch is distinctly triangular or semicircular (the basal fascia is conspicuously arched and fully continuous with the longitudinal band) and its medial longitudinal extent is more than 1/3 the lateral extent. In *E. compactus*, the medially-interrupted T1 basal and apical fasciae may be so broad laterally that they are joined, resulting in a diamond shape but with concave sides; in *E. canadensis* the lateral sides are straight or convex.

#### Redescription.

This species was recently redescribed (Onuferko 2017).

#### Distribution.

Western North America (Fig. 37).

#### Ecology.

See Onuferko (2017) for host and floral records. Floral associations are also indicated in Suppl. material 1.

#### Discussion.

*Epeolus compactus* is a commonly collected species, widespread in Western North America. It is most similar to *E. canadensis* and *E. ferrarii*. In the original description of *E. crucis* Cockerell, the holotype was said to have been initially identified as *E. compactus* by W.J. Fox, but Cockerell (1904) considered it to be distinct, mainly because of differences in coloration and pubescence. The specimen (unusually) has abundant pale tomentum on the discs of the metasomal terga (Fig. 38A), but representatives of several species (e.g., *E. ainsliei*, *E. minimus*, and *E. novomexicanus*) exhibiting atypical abundance of pale tomentum on the mesosoma and metasoma were also observed. Despite the presence of pale tomentum, the discal patch is quadrangular/diamond-shaped (Fig. 38A) as is typical for *E. compactus* (Fig. 38B), and the fascia of T2 is separated medially into rounded lobes. In the *E. crucis* holotype, the axillae and mesoscutellum are (unusually) ferruginous, but it is not unprecedented for species of the genus to have representatives displaying atypical integument coloration. Interestingly, Brumley (1965) treated *E. crucis* as distinct, but the features listed as unique for that species are evident only in the holotype of *E. rufulus*. In fact, his key does not work for the holotypes of *E. crucis* and *E. novomexicanus*, which Brumley believed to be the same species. Unlike in *E. rufulus*, in the *E. crucis* holotype the axillae do not extend beyond the midlength of the mesoscutellum, and the axilla is not conspicuously diverging from the side of the mesoscutellum – the free portion is less than 1/3 as long as the entire medial length of the axilla. As a result of Brumley’s work, specimens of what are actually *E. rufulus* housed at various entomological institutions have been identified (or rather misidentified) as *E. crucis*.

#### Material studied.

**Type material.** Primary: USA: **California**: Mill Creek Canyon (San Bernardino County), 12.ix.1923, E.P. Van Duzee (*E. geminatus* holotype ♀ [CAS, catalog number: 01610]); San Gabriel Mountains (near Pasadena), 15.vii.1909, F. Grinnell, Jr. (*T. gabrielis* holotype ♂ [USNM, catalog number: 534044]); **Colorado**: Copeland Park (Boulder County), 06.ix.1907, G.M. Hite (*E. hitei* holotype ♀ [USNM, catalog number: 534045]); **New Mexico**: Las Cruces, C.H. Townsend (*E. crucis* holotype ♀ [USNM, catalog number: 534043]); **Texas**: G.W. Belfrage (*E. compactus* lectotype ♀ [ANSP, catalog number: 2227]).

Secondary: USA: **Colorado**: (*E. compactus* paralectotype ♀, AMNH).

#### DNA barcoded material with BIN-compliant sequences.

Available. BOLD:ACU6228. Specimens examined and sequenced.–Canada: **Manitoba**: Birds Hill Provincial Park (50.0114°N; 96.9028°W) (Division 12), 15.vii.2017, J. Gibbs (1♂, JBWM).

USA: **California**: 1♀ (PCYU); **Oregon**: 3♂ (PCYU); **Washington**: 1♀ (PCYU).

#### Non-barcoded material examined.

Canada: **Alberta**: 1♀ (KUNHM); **British Columbia**: 2♀, 1♂ (CNC); McIntyre Road (Oliver), 29.v.1958, H. and A. Howden (1♂, CNC); **Saskatchewan**: 1♂ (CNC).

Mexico: **Baja California**: 1 mi W San Borja, 12–13.vi.1967, E.L. Sleeper and E.M. Fisher (1♀, LACM); **Baja California Sur**: 6 km E Insurgentes, 24.iv.1975, E.M. Fisher (1♀, LACM); La Paz and vicinity, 11–14.vi.1975, H. Evans, W. Rubink, and D. Gwynne (1♀, CUM); **Durango**: Durango, 13.viii.1962, A.E. Michelbacher (1♀, EMEC); **Sonora**: 16 mi NW Puerto Peñasco, 29.iii.1965, C.J. McCoy (1♂, CUM).

USA: **Arizona**: 2♀, 1♂ (AMNH, PCYU); 15 mi S Bullhead City (Mohave County), 07.iv.1977, L. Bezark (1♀, UCBME); Oak Creek Valley Road (Yavapai County), 16.vi.1978, R.C. Miller (1♀, UCBME); **California**: 1♀, 3♂ (AMNH, FSCA); Andreas Canyon (Riverside County), 30.iii.1977, R.M. Bohart (1♂, UCBME); Arroyo Seco Campground (Monterey County), 19.v.1964, F.D. Parker (1♀, UCBME); 19.v.1964, R.M. Bohart (1♂, UCBME), 23.vii.1967, R.F. Denno (1♀, UCBME); Charlton Flats (San Gabriel Mountains), 08.ix.1977, A.S. Menke (1♀, UCBME); Felton Springs (Santa Cruz County), 16.vi.1973, R.M. Bohart (1♂, UCBME); Granite Mountains (San Bernardino County), 10.x.1977, N.J. Smith (1♀, UCBME), 10.x.1977, R.M. Bohart (1♀, UCBME); Mojave (Kern County), 23.v.1978, R.P. Meyer (2♂, UCBME); Peña Spring (San Diego County) (1♀, BBSL); Thousand Palms (Riverside County), 11.iv.1970, E.E. Grissell (1♀, UCBME); **Colorado**: 3♀ (AMNH, PCYU); **Nevada**: Kings Canyon (5 mi W Carson City), 07.viii.1975, B. Villegas (1♂, UCBME); **New Mexico**: 8♂ (AMNH, PCYU); Granite Gap (18 mi N Rodeo, Hidalgo County), 07.ix.1976, R.M. Bohart (1♀, UCBME); **Oklahoma**: 1♀ (FSCA); **Oregon**: 1♂ (PCYU); **Texas**: 7.6 mi S Van Horn (Culberson County), 27.iv.1979, R.R. Snelling (1♀, LACM); Rd 1108 (4–8 mi SE 652, Culberson County), 14.vi.2005, J.L. Neff and A. Hook (1♂, CTMI); Z H Canyon (30.0920°N; 104.6620°W) (Presidio County), 19.v.2005, J.L. Neff and A. Hook (1♀, CTMI); **Washington**: 1♀ (PCYU); **Wyoming**: 1♀, 2♂ (AMNH).

### 18. Epeolus deyrupi sp. n.

Taxon classificationAnimaliaHymenopteraApidae

http://zoobank.org/2434A23D-BC6F-49A0-B272-085804B1BCAE

Figs 39 , 40 , 92C

#### Diagnosis.

The following morphological features in combination can be used to tell *E. deyrupi* apart from all other North American *Epeolus*: the axilla is large, with the tip extending well beyond the midlength of the mesoscutellum, dilated laterally, and like the mesoscutellum ferruginous; the mesopleuron commonly has sparser punctures ventrolaterally (i≤2d) than in upper half, with the interspaces shining or somewhat dull due to tessellate surface microsculpture; and the T1–T3 apical fasciae are interrupted and (to varying degrees) brownish orange medially and off white laterally. *Epeolus deyrupi* resembles *E. andriyi*, *E. floridensis*, *E. howardi*, and *E. packeri* in that the axilla is large, with the lateral margin arcuate, and like the mesoscutellum ferruginous, and that the T1–T3 apical fasciae are interrupted medially. However, in *E. deyrupi* the pseudopygidial area of the female is wider (the apex >2 × the medial length) than in *E. andriyi*, *E. floridensis*, or *E. howardi* (the apex <2 × the medial length), and the T1 basal fascia is absent or reduced to a pair of small patches of pale tomentum whereas in *E. andriyi*, *E. floridensis*, and *E. howardi* T1 has a distinct, although often medially-interrupted, basal fascia. *Epeolus deyrupi* closely resembles *E. packeri*, but in *E. packeri* the mesopleuron has denser punctures ventrolaterally (most i<1d) than that of *E. deyrupi* and the metasomal terga have pale but not brownish orange pubescence.

#### Description.

FEMALE: Length 8.8 mm; head length 2.2 mm; head width 2.9 mm; fore wing length 6.1 mm.

*Integument coloration.* Mostly black; notable exceptions as follows: at least partially ferruginous on mandible, labrum, clypeus, antenna, pronotal collar, pronotal lobe, tegula, axilla, mesoscutum, mesoscutellum, metanotum, mesopleuron, metapleuron, propodeum, and legs. Mandible with apex darker than rest of mandible; preapical tooth lighter than mandibular apex (difficult to see in holotype because mandible closed; described from paratypes). Antenna brown and orange in part. Pronotal lobe and tegula pale ferruginous to amber. Mesoscutum reddish brown laterally and posteriorly. Wing membrane subhyaline, apically dusky. Legs more extensively reddish orange than brown or black.

*Pubescence.* Face with tomentum densest around antennal socket. Dorsum of mesosoma and metasoma with bands of off-white and brownish orange short appressed setae. Mesoscutum with paramedian band. Mesopleuron mostly bare, but tomentum moderately dense ventrally as well as between two almost entirely bare patches (one beneath base of fore wing (hypoepimeral area), a larger circular patch occupying much of ventrolateral half of mesopleuron). Metanotum with tomentum uninterrupted except for median bare patch in posterior half (also bare along posterior margin), uniformly off white. T1 with basal fascia reduced to pair of small patches of off-white tomentum; T1–T4 with apical fasciae brownish orange medially and off white laterally, and medially interrupted and removed from apical margin; T2 with fascia without anterolateral extensions of tomentum. T4 with fascia interrupted laterally. T5 with two patches of pale tomentum bordering and separate from pseudopygidial area. T5 with pseudopygidial area lunate, its apex more than twice as wide as medial length, indicated by silvery setae on disc of apicomedial region elevated from rest of tergum. S5 with apical fimbria of coppery to silvery hairs not extending beyond apex of sternum by more than 1/4 MOD.

*Surface sculpture.* Punctures dense. Labrum with larger and sparser punctures (i=1–2d) than clypeus (i<1d). Small impunctate shiny spot lateral to lateral ocellus. Mesoscutum, mesoscutellum, and axilla coarsely and densely rugose-punctate. Tegula densely punctate mesally (i≤1d), less so laterally (i=1–2d). Mesopleuron with denser (i≤1d) punctures in upper half than ventrolateral half (i≤2d), the interspaces somewhat dull due to tessellate surface microsculpture. Metasomal terga with punctures very fine, dense (i≈1d), evenly distributed on disc.

*Structure.* Preapical tooth obtuse. Labral apex with pair of small denticles, each preceded by longitudinal carina. Frontal keel not strongly raised. Scape with greatest length 1.7 × greatest width. F2 noticeably longer than wide (L/W ratio = 1.2). Preoccipital ridge not joining hypostomal carina, from which it is separated by less than 1 MOD at its terminal. Mesoscutellum moderately bigibbous. Axilla large, its lateral margin (L) more than half as long as mesoscutellar width (W) (L/W ratio = 0.6) and tip nearly extending as far back as apex of horizontal dorsal portion of mesoscutellum; axilla with tip clearly visible, but unattached to mesoscutellum for less than 2/5 the medial length of axilla; axilla with lateral margin arcuate. Fore wing with three submarginal cells. Pygidial plate apically truncate.

MALE: Description as for female except for usual secondary sexual characters and as follows: F2 shorter, not noticeably longer than wide (L/W ratio = 1.1); S4 and S5 with much longer coppery to silvery subapical hairs; pygidial plate with apex slightly concave and large deep punctures closely clustered basally and sparser apically, with the interspaces shining.

**Figure 39.**
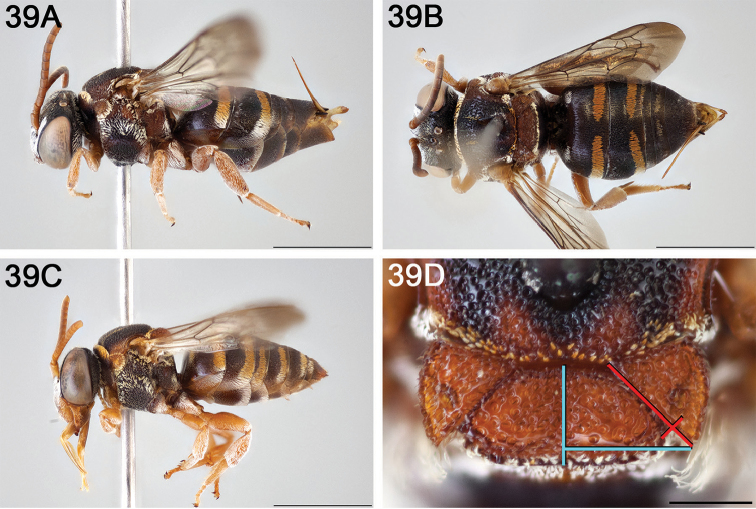
*Epeolus deyrupi* **A** female holotype, lateral habitus (scale bar 3 mm) **B** female holotype, dorsal habitus (scale bar 3 mm) **C** male allotype, lateral habitus (scale bar 3 mm), and **D** female paratype axillae and mesoscutellum, dorsal view (scale bar 0.5 mm; blue lines indicate the posterior extent of the axilla relative to the length of the mesoscutellum; red lines indicate the extent of the free portion of the axilla relative to its entire medial length).

#### Etymology.

This species is named after its discoverer, Dr. Mark A. Deyrup, who recognized it as a new species and brought his discovery to my attention.

#### Distribution.

**Figure 40.**
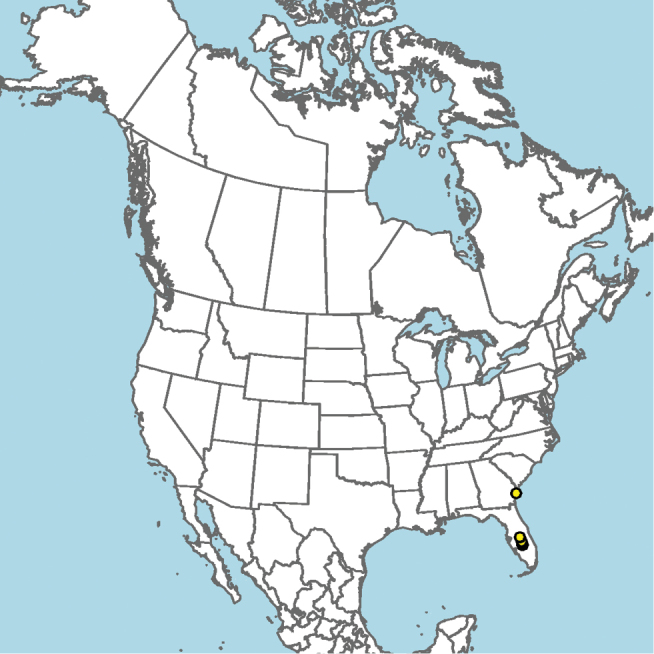
Occurrence records of *E. deyrupi* known to the author (yellow circles).

Florida and coastal Georgia (Fig. 40).

#### Ecology.

HOST RECORDS: The host species of *E. deyrupi* is/are presently unknown.

FLORAL RECORDS: Labels of examined voucher specimens indicate a floral association with *Sideroxylon tenax* L. (Sapotaceae).

#### Discussion.

*Epeolus deyrupi* is a southeastern species that exhibits very little intraspecific morphological variation. Most of the known specimens of this species were collected in Highlands County, Florida. Based on known records, adults of *E. deyrupi* are active in spring.

#### Material studied.

**Type material.** Primary: USA: **Florida**: Flamingo Villas Preserve (27.4423°N; 81.3782°W) (Highlands County), 26.v.2009, M. Deyrup, A. May, and H. Otte (holotype ♀ [CCDB-24583 F06], FSCA).

Secondary: USA: **Florida**: Allen David Broussard Catfish Creek Preserve State Park (27.8503°N; 81.4954°W) (Polk County), 08.vi.2009, M. Deyrup, A. May, and H. Otte (paratype ♂, ABS); Archbold Biological Station (27.1239°N; 81.3661°W) (Highlands County), 21.vi.2010, M. and L. Deyrup (paratype ♀, ABS); Archbold Biological Station (Highlands County), 29.v.1979, H.V. Weems, Jr. and S. Halkin (paratype ♀, LACM), 14.vi.2010, M. and L. Deyrup (paratype ♀, ABS); Flamingo Villas Preserve (27.4423°N; 81.3782°W) (Highlands County), 25.v.2009, M. Deyrup, A. May, and H. Otte (paratype ♀, ABS); Flamingo Villas Preserve (27.4487°N; 81.3767°W) (Highlands County), 01.vi.2009, M. Deyrup, A. May, and H. Otte (paratype ♀, ABS); Gould Rd Preserve (27.1336°N; 81.3256°W), 25.v.2009, M. Deyrup, A. May, and H. Otte (paratype ♀, PCYU), 26.v.2009, M. Deyrup, A. May, and H. Otte (paratype ♀, ABS); Lake Placid (Archbold Biological Station, Highlands County), 12.vi.1983, M. Deyrup (paratype ♀, ABS), 11.vi.1986, M. Deyrup (paratype ♀, ABS); The Nature Conservancy Tiger Creek Preserve (27.8077°N; 81.4816°W) (Polk County), 04.vi.2010, J. Dunlap, M. and N. Deyrup, and K. Dearborn (paratype ♀ [CCDB-24583 H04], PCYU); Tiger Creek Preserve (27.8133°N; 81.4868°W) (Polk County), 12.vi.2010, J. Dunlap, M. and N. Deyrup, and K. Dearborn (paratype ♀ [CCDB-24583 H02], USNM); **Georgia**: St Catherines Island (Liberty County), 24–27.vi.1989, Rozen, Quinter, and Eickwort (allotype ♂, AMNH), 27.vi.1974, R.O. Schuster and E.C. Teftner (paratype ♂, UCBME).

#### DNA barcoded material with BIN-compliant sequences.

Available. BOLD:ADF0241. See Type material for specimens examined and sequenced (indicated by unique CCDB-plate and well number).

### 19. Epeolus diadematus sp. n.

Taxon classificationAnimaliaHymenopteraApidae

http://zoobank.org/BB07B4CB-2B68-4F76-8230-7DF4F565EF72

Figs 41 , 42 , 92J

Epeolus torus Brumley, 1965. M.S. thesis, Utah State University, Logan 71 (♀) [*nomen nudum*].

#### Diagnosis.

*Epeolus diadematus* does not closely resemble any other species of *Epeolus* except *E. chamaesarachae*. Unique in the genus to both species are each of the following morphological features: the vertexal area has two pairs of shiny (usually impunctate) protrusions, the mesoscutum is distinctly ornamented with mostly separate patches of (but some intermixed) pale and ferruginous tomentum, and the T2 fascia has two pairs of anterolateral extensions of tomentum. The difference is that in *E. diadematus* the mesopleuron has denser punctures ventrolaterally (most i≤1d) whereas in *E. chamaesarachae* the mesopleuron has sparser (most i>1d) and fewer punctures ventrolaterally.

#### Description.

FEMALE: Length 6.9 mm; head length 2.0 mm; head width 2.6 mm; fore wing length 6.0 mm.

*Integument coloration.* Mostly black; notable exceptions as follows: partially to entirely ferruginous on mandible, antenna, pronotal collar, pronotal lobe, tegula, axilla, mesoscutellum, and legs. Mandible with apex darker than all but extreme base; preapical tooth lighter than mandibular apex. Antenna dark brown except scape, pedicel, and F1 brownish orange in part. Pronotal lobe and tegula pale ferruginous to amber. Wing membrane subhyaline, apically dusky. Legs more extensively reddish orange than brown or black.

*Pubescence.* Face with tomentum densest around antennal socket. Vertexal area with tomentum mostly ferruginous. Dorsum of mesosoma with bands of off-white and ferruginous short appressed setae. Dorsum of metasoma with bands of off-white to pale yellow short appressed setae. Pronotal collar with tomentum black medially, pale and ferruginous laterally. Mesoscutum with paramedian band of pale tomentum; ferruginous and pale tomentum encircling black spots medially and laterally, respectively, on anterior margin; and ferruginous tomentum along medial mesoscutal line and parapsidal line. Mesopleuron with upper half densely hairy, although scrobe visible; ventrolateral half nearly bare. Metanotum with tomentum uninterrupted, off white laterally and black medially. T1 with median diamond-shaped black discal patch enclosed by pale tomentum, except for medial separation at apex. T1 with apical fascia with black spot posterolaterally. T2–T4 with fasciae interrupted medially, T2 with fascia with paired anterolateral extensions of tomentum. T3 and T4 with fasciae interrupted laterally, with medial portion on apical margin and lateral portion encircling black tomentum on apical margin. T5 with two large patches of pale tomentum lateral to and separate from pseudopygidial area. T5 with pseudopygidial area lunate, its apex more than twice as wide as medial length, indicated by silvery setae on disc of apicomedial region elevated from rest of tergum. S5 with apical fimbria of coppery to silvery hairs extending beyond apex of sternum by ~1/3 MOD.

*Surface sculpture.* Punctures dense, but those of head and mesosoma sparser in some areas, larger, deep, and distinct. Labrum mostly with larger and sparser punctures (i=1–2d) than clypeus (i≤1d). Upper paraocular area and vertexal area sparsely punctate (i=1–2d), the interspaces shining. Mesoscutum, mesoscutellum, and axilla coarsely and densely rugose-punctate; the interspaces shining. Tegula densely punctate mesally (i=1–2d), much less so laterally (i>2d). Mesopleuron with denser (i<1d) punctures in upper half than ventrolateral half, although ventrolateral half with most interspaces small (i≤1d); the interspaces shining. Metasomal terga with punctures very fine, dense (i≈1d), evenly distributed on disc.

*Structure.* Labral apex with two pairs of small denticles (the middlemost pair preceded by submedial pair of small denticles and separated by shallow concavity). Frontal keel strongly raised. Vertexal area with two pairs of nearly impunctate shiny protrusions. Scape with greatest length 1.6 × greatest width. F2 as long as wide (L/W ratio = 1.0). Preoccipital ridge not joining hypostomal carina, from which it is separated by no less than 1 MOD at its terminal. Mesoscutellum strongly bigibbous. Axilla intermediate in size, its lateral margin (L) nearly half as long as mesoscutellar width (W) (L/W ratio = 0.4–0.5) and tip not extending much beyond midlength of mesoscutellum (extending to <2/3 its length); axilla with tip conspicuously diverging from side of mesoscutellum, distinctly hooked, but unattached to mesoscutellum for less than 1/3 the medial length of axilla; axilla with lateral margin somewhat arcuate. Fore wing with three submarginal cells. Pygidial plate mostly hidden in holotype, but apically truncate in paratypes.

MALE: Description as for female except for usual secondary sexual characters and as follows: F2 shorter, nearly as long as wide (L/W ratio = 0.8); S4 and S5 with much longer coppery to silvery subapical hairs; pygidial plate apically rounded, with large deep punctures closely clustered apically and sparser basally, with the interspaces shining.

**Figure 41.**
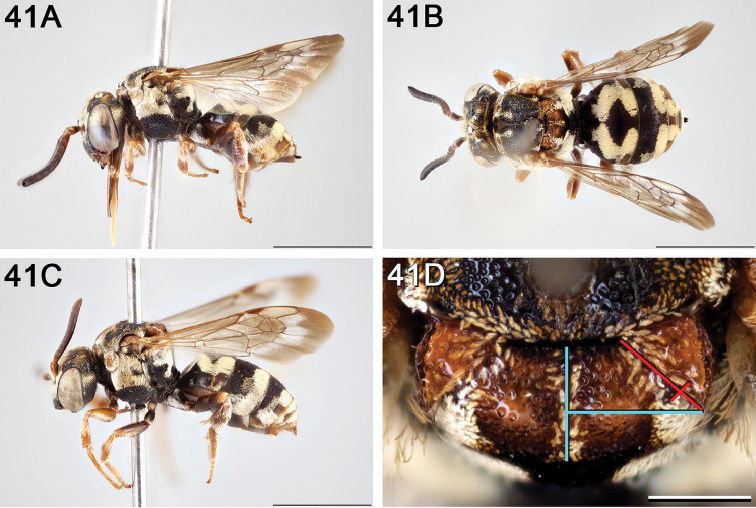
*Epeolus diadematus* **A** female holotype, lateral habitus (scale bar 3 mm) **B** female holotype, dorsal habitus (scale bar 3 mm) **C** male paratype, lateral habitus (scale bar 3 mm), and **D** female paratype axillae and mesoscutellum, dorsal view (scale bar 0.5 mm; blue lines indicate the posterior extent of the axilla relative to the length of the mesoscutellum; red lines indicate the extent of the free portion of the axilla relative to its entire medial length).

#### Etymology.

The name is in reference to the four shiny, usually impunctate, tubercles on the vertexal area of the head of this species. From the Latin, “diadema” (royal headband).

#### Distribution.

**Figure 42.**
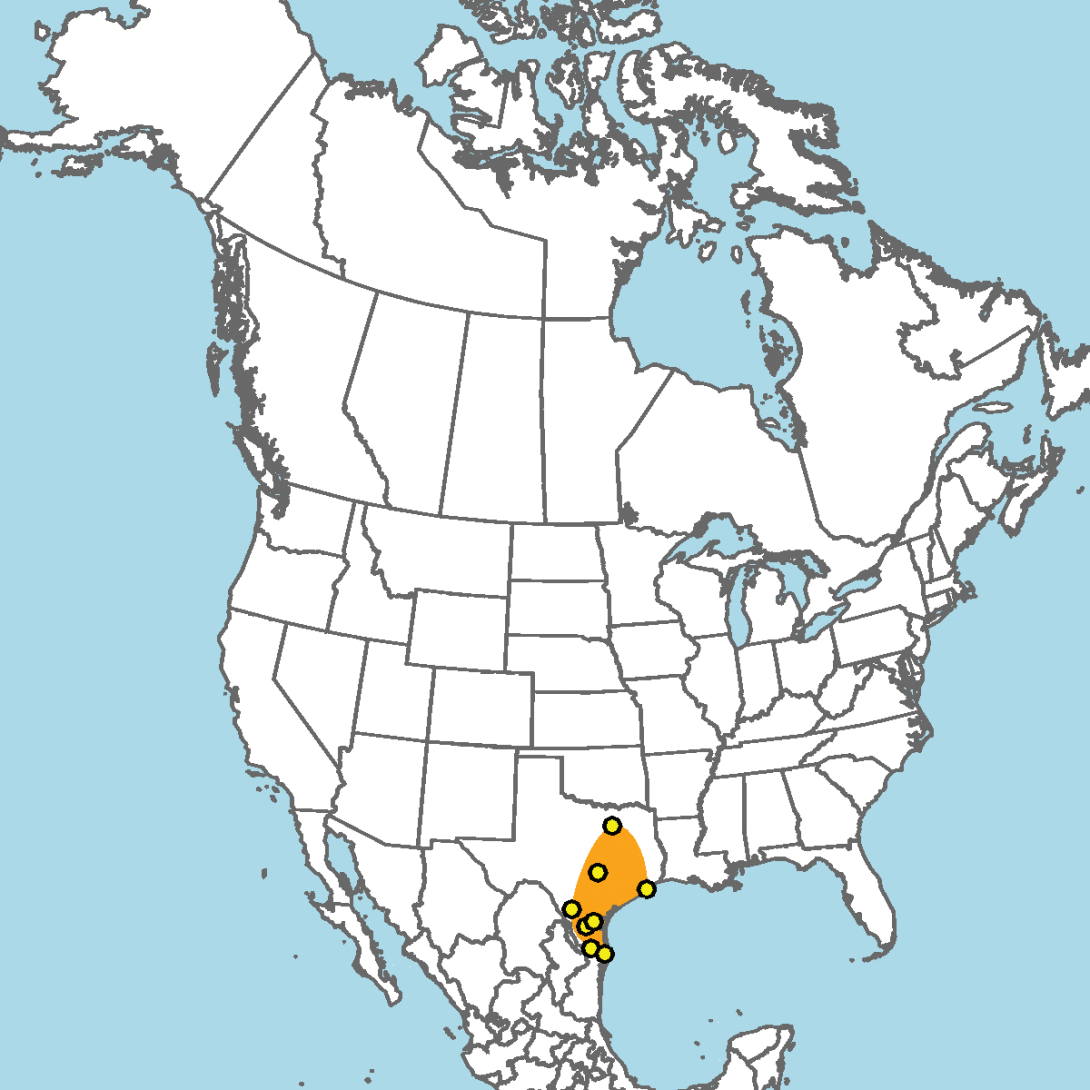
Approximate geographic range of *E. diadematus* (orange) based on occurrence records known to the author (yellow circles).

Texas and presumably Mexico, given the close proximity of some collection localities (e.g., Southmost, Texas) to the Mexico–United States border (Fig. 42).

#### Ecology.

HOST RECORDS: The host species of *E. diadematus* is/are presently unknown.

FLORAL RECORDS: The label of one examined voucher specimen indicates a floral association with *Engelmannia pinnatifida* A.Gray ex Nutt. (Compositae). This species has also been collected from *Aphanostephus riddellii* Torr. & A. Gray (Compositae) (J. Neff, personal communication, 2016).

#### Discussion.

This species and *E. chamaesarachae* are very similar in terms of integument coloration, pubescence, and structure, and are presumably sister species. Specimens of *E. diadematus* are distinct from those designated as *E. chamaesarachae* in that the mesopleuron has much denser punctation. The status of *E. diadematus* as a separate species is further supported by a separate BIN and large barcode sequence divergence (3.2%) from its nearest neighbor, *E. chamaesarachae* (Suppl. material 2). The ranges and flight seasons of these species also differ. With one exception, examined specimens of *E. diadematus* were collected in spring, and all are from Coastal or South Texas. By contrast, *E. chamaesarachae* occurs further west in the United States, and adults are active in late summer.

#### Material studied.

**Type material.** Primary: USA: **Texas**: McAllen Botanical Gardens (McAllen), 21.xi.1982, C. Porter (holotype ♀, FSCA).

Secondary: USA: **Texas**: 5 mi SE Realitos (27.3980°N; 98.5490°W) (Duval County), 22.iv.2005, J.L. Neff and A. Hook (paratype ♂, CTMI); Ben Bolt (Jim Wells County), 12.v.1952, M. Cazier, W. Gertsch, and R. Schrammel (paratype ♀, AMNH); Brackenridge Field Laboratory (Austin, Travis County), 28.iv.1989, A. Hook (paratype ♂, CTMI); Chaparral Wildlife Management Area (Dimmit County), 06.iv.2007, J.L. Neff and A. Hook (paratype ♂, CTMI), 11.iv.2003, J.L. Neff and A. Hook (paratype ♂, CTMI); Dallas, 22.v.??06, W.D. Pierce (paratypes 2♂, USNM); Galveston?, L. Packer (paratype ♀ [CCDB-30383 F06], PCYU); Southmost (Cameron County), 13.vi.1953, Univ. Kans. Mex. Expedition (allotype ♂, KUNHM).

#### DNA barcoded material with BIN-compliant sequences.

Available. BOLD:ADJ9659. See Type material for specimens examined and sequenced (indicated by unique CCDB-plate and well number).

### 20. Epeolus erigeronis

Taxon classificationAnimaliaHymenopteraApidae

Mitchell, 1962

Figs 43 , 44 , 92E

Epeolus erigeronis Mitchell, 1962. N. C. Agric. Exp. Stn. Tech. Bull. 152: 445 (♀).

#### Diagnosis.

The following morphological features in combination (excluding any that are specific to the opposite sex of the one being diagnosed) can be used to tell *E. erigeronis* apart from all other North American *Epeolus* except *E. ilicis* and *E. inornatus*: the mandible is simple; the axilla does not attain the midlength of the mesoscutellum but the free portion is distinctly hooked, with the tip unattached to the mesoscutellum for more than 1/3 of the entire medial length of the axilla; the pronotal collar and metasomal terga are black; the metasomal terga have rather fine punctures; and the pseudopygidial area of the female is distinctly campanulate with the apex <2 × the medial length and not in contact with two large patches of pale tomentum (one on each side) throughout its length (in contact only at apex, diverging basally). Although in all three species the mesopleuron is closely and evenly punctate, in *E. erigeronis* the punctures are more variable in size, with many smaller punctures among large ones, and most interspaces are narrower such that the surface appears to be very coarsely and densely rugose-punctate. By contrast, in *E. ilicis* and *E. inornatus* the mesopleuron has punctures that are similar in size and shiny interspaces that are commonly equal to the puncture diameters.

#### Redescription.

FEMALE: Length 8.6 mm; head length 2.2 mm; head width 3.0 mm; fore wing length 6.3 mm.

*Integument coloration.* Mostly black; notable exceptions as follows: partially to entirely ferruginous on mandible, labrum, antenna, pronotal lobe, tegula, and legs. Mandible with apex darker than all but extreme base. Antenna brown except scape, pedicel, and F1 orange in part. Pronotal lobe and tegula pale ferruginous to amber. Wing membrane subhyaline, apically dusky. Legs more extensively reddish orange than brown or black.

*Pubescence.* Face with tomentum densest around antennal socket. Tomentum slightly sparser on clypeus; upper paraocular and frontal areas, and vertexal area mostly exposed. Dorsum of mesosoma and metasoma with bands of off-white to pale yellow short appressed setae. Mesoscutum with paramedian band. Mesopleuron with upper half hairy, except beneath base of fore wing (hypoepimeral area); ventrolateral half nearly bare. Metanotum with tomentum uninterrupted except for median bare patch in posterior half, uniformly off white. T1 with discal patch quadrangular and very wide, the basal and apical fasciae only narrowly joined laterally. T1 and T2 with apical fasciae interrupted medially, those of T2 and T3 somewhat broader laterally, T2 with fascia with faint anterolateral extensions of sparser tomentum. T3 and T4 with fasciae complete. T5 with two large patches of pale tomentum lateral to and separate from pseudopygidial area. T5 with pseudopygidial area campanulate, its apex less than twice as wide as medial length, indicated by silvery setae on impressed disc of apicomedial region elevated from rest of tergum. S5 with apical fimbria of coppery to silvery hairs extending beyond apex of sternum by 1/3 MOD.

*Surface sculpture.* Punctures dense. Labrum with larger punctures than clypeus, but punctures of both equally dense (i<1d). Small impunctate matte spot lateral to lateral ocellus. Mesoscutum, mesoscutellum, and axilla coarsely and densely rugose-punctate. Tegula very densely punctate mesally (i<1d), less so laterally (i=1–2d). Mesopleuron with ventrolateral half coarsely and densely rugose-punctate (i<1d), the interspaces somewhat dull due to surface microsculpture; mesopleuron with many smaller punctures among large ones, punctures more or less equally dense throughout. Metasomal terga with punctures very fine, dense (i=1–2d), evenly distributed on disc; the interspaces shining somewhat.

*Structure.* Mandible without preapical tooth. Labrum with pair of small subapical denticles not preceded by carinae. Frontal keel not strongly raised. Scape with greatest length 1.8 × greatest width. F2 noticeably longer than wide (L/W ratio = 1.6). Preoccipital ridge not joining hypostomal carina, from which it is separated by no less than 1 MOD at its terminal. Mesoscutellum weakly bigibbous. Axilla intermediate in size, its lateral margin (L) nearly half as long as mesoscutellar width (W) (L/W ratio = 0.4–0.5) and tip attaining midlength of mesoscutellum; axilla with tip conspicuously diverging from side of mesoscutellum, distinctly hooked, and axilla with free portion 2/5 its medial length; axilla with lateral margin arcuate and carinate. Fore wing with three submarginal cells. Pygidial plate apically truncate.

MALE: Description as for female except for usual secondary sexual characters and as follows: F2 shorter, but still longer than wide (L/W ratio = 1.3); S4 and S5 with much longer coppery to silvery subapical hairs; pygidial plate apically rounded, with large deep punctures closely clustered basomedially and sparser apically and laterally, with the interspaces shining.

**Figure 43.**
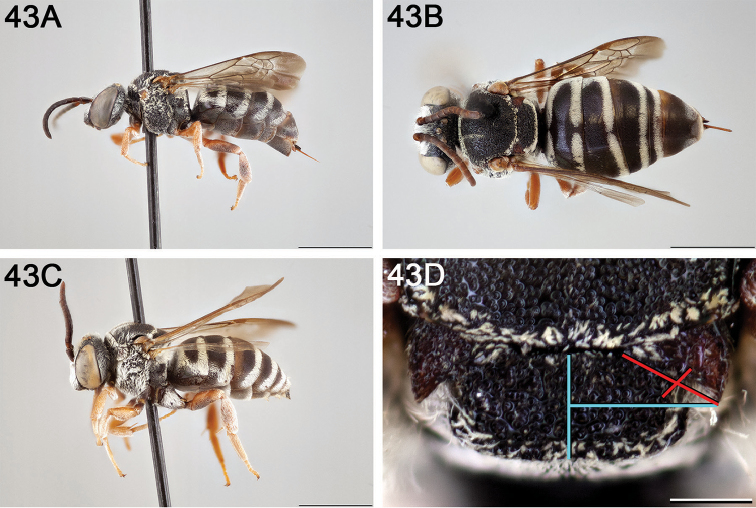
*Epeolus erigeronis* **A** female, lateral habitus (scale bar 3 mm) **B** female, dorsal habitus (scale bar 3 mm) **C** male, lateral habitus (scale bar 3 mm), and **D** female axillae and mesoscutellum, dorsal view (scale bar 0.5 mm; blue lines indicate the posterior extent of the axilla relative to the length of the mesoscutellum; red lines indicate the extent of the free portion of the axilla relative to its entire medial length).

#### Distribution.

**Figure 44.**
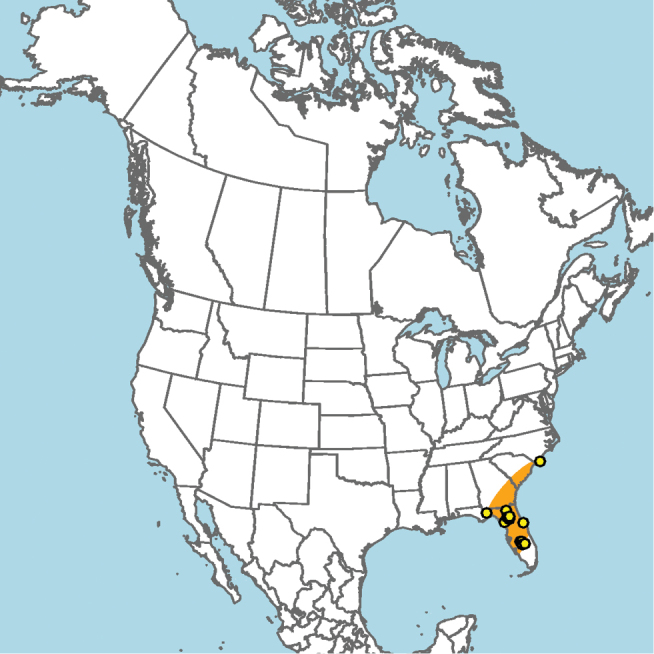
Approximate geographic range of *E. erigeronis* (orange) based on occurrence records known to the author (yellow circles).

South Atlantic states (Fig. 44).

#### Ecology.

HOST RECORDS: The host species of *E. erigeronis* is/are presently unknown.

FLORAL RECORDS: Mitchell (1962) indicated floral associations with *Erigeron quercifolius* Lam. (Compositae), *Hypericum* L. (Hypericaceae), and *Melilotus albus* Medik. (Leguminosae). Labels of examined voucher specimens further indicate associations with *Clinopodium ashei* (Weath.) Small (Lamiaceae), *Ilex glabra* (L.) A. Gray (Aquifoliaceae), and *Vaccinium darrowii* Camp (Ericaceae).

#### Discussion.

*Epeolus erigeronis* exhibits very little intraspecific morphological variation. However, in some specimens the axillae are partially ferruginous whereas in others they and the mesoscutellum are entirely black. Based on examined records, adults of *E. erigeronis* are active throughout spring.

Although BIN-compliant sequences are presently not available for *E. erigeronis*, four partial sequences (three 422 bp and one 394 bp in length) are available for specimens from North and South Florida, and these sequences form a distinct cluster that does not include any sequences from other *Epeolus* species in a NJ tree (Suppl. material 2).

#### Material studied.

**Type material.** Primary: USA: **Florida**: Levy County, 13.iv.1955, H.V. Weems, Jr. (holotype ♀, FSCA).

Secondary: USA: **Florida**: Alachua County, 15.iv.1955, R.A. Morse (paratype ♀, FSCA); Levy County, 13.iv.1955, H.V. Weems, Jr. (allotype ♂, FSCA); **North Carolina**: Southport, 24.vi.1928, T.B. Mitchell (paratype ♀, NHMUK).

#### DNA barcoded material with BIN-compliant sequences.

Unavailable.

#### Non-barcoded material examined.

USA: **Florida**: 5 mi S Paynes Prairie (SE Gainesville, Alachua County), 05–12.v.1996, B.D. Sutton (1♀, FSCA); Apalachicola National Forest (30.3292°N; 84.5052°W) (Wakulla County), 08–15.v.2005, Ronquist lab (1♀, PCYU); Archbold Biological Station (Highlands County), 10.v.1979, H.V. Weems, Jr. and S. Halkin (1♀, BBSL), 17–23.iv.2007, S.M. Paiero (1♂, DEBU), 17.v.2005, M. Deyrup (1♀, ABS), 08.iv.1980, H.V. Weems, Jr. and F.E. Lohrer (1♀, FSCA), 24.iii.1980, H.V. Weems, Jr. and F.E. Lohrer (1♂, FSCA); Archbold Biological Station (27.1838°N; 81.3532°W) (Highlands County), 23.v.2010, M. Deyrup (1♀, ABS), 28.v.2010, M. Deyrup (1♀, ABS); Austin Cary Forest (Gainesville, Alachua County), 10.vi.1976 (1♂, UCBME), 16.x.1977, G.B. Fairchild (1♀, UCBME), 17.v.1991, L.R. Davis, Jr. (1♀, FSCA), 20.vi.1978, G.B. Fairchild and H.V. Weems, Jr. (1♀, UCBME); Brighton, 07.iv.1937, H.I. Scudder (1♀, CAS); Flamingo Villas Preserve (27.4487°N; 81.3767°W) (Highlands County), 01.vi.2009, M. Deyrup, A. May, and H. Otte (1♀, ABS); Flamingo Villas Preserve (27.4515°N; 81.3854°W) (Highlands County), 05.v.2010, M. Deyrup and J. Dunlap (1♀, ABS); Highlands Hammock State Park, 14.iv.1968, H.V. Weems, Jr. (2♀, FSCA); Kincaid Road (SE Gainesville, Alachua County), 03.iv.1999, B.D. Sutton (1♀, FSCA); Lake Placid (27.2195°N; 81.3803°W) (Highlands County), 14.iv.2010, M. Deyrup and J. Dunlap (1♀, ABS); New Smyrna Beach, 14.iii.1943, R.L. Usinger (1♂, EMEC); Osceola National Forest (Baker County and Columbia County line), 13–26.iv.1977, J.R. Wiley (1♂, FSCA), 01.v.2011, S. Lenberger (1♀, FSCA); San Felasco State Hammock Preserve, 16.v.1977, G.B. Fairchild and H.V. Weems, Jr. (1♀, UCBME).

### 21. Epeolus ferrarii sp. n.

Taxon classificationAnimaliaHymenopteraApidae

http://zoobank.org/AB9DAE3B-CBB7-4540-AA11-8C1D123BB7F0

Figs 45 , 46

#### Diagnosis.

The following morphological features in combination (excluding any that are specific to the opposite sex of the one being diagnosed) can be used to tell *E. ferrarii* apart from all other North American *Epeolus* except *E. canadensis* and *E. compactus*: in females, F2 is at least 1.2 × as long as wide; the mesoscutum has a small anteromedial patch of pale tomentum; the axilla is small to intermediate in size, not extending much beyond the midlength of the mesoscutellum (extending to <2/3 its length) but the free portion is more than 1/4 as long as the entire medial length of the axilla, and the axilla (except sometimes the tip) and mesoscutellum are black; the mesopleuron is closely (most i<1d) and evenly punctate; and the T2 fascia lacks lobe-like anterolateral extensions of tomentum, although it is broader laterally. *Epeolus ferrarii* is most similar to *E. compactus*, and in both species the T1 discal patch is typically quadrangular with the basal and apical fasciae subparallel and separated by a distinct longitudinal band, but in *E. compactus* the T2–T4 fasciae are not evenly broad or tapering until separated medially (as in *E. ferrarii*) but broadened medially into rounded lobes, which may be joined or separated. *Epeolus canadensis* differs from both species in that the T1 discal patch is distinctly triangular or semicircular (the basal fascia is conspicuously arched and fully continuous with the longitudinal band) and its medial longitudinal extent is more than 1/3 the lateral extent. In *E. ferrarii* the discal patch may be trapezoidal or almost semicircular, but if at all semicircular its medial longitudinal extent is at most 1/3 the lateral extent and the basal fascia and longitudinal band are at least joined at somewhat of an angle.

#### Description.

MALE: Length 7.1 mm; head length 1.9 mm; head width 2.6 mm; fore wing length 6.0 mm.

*Integument coloration.* Mostly black; notable exceptions as follows: partially to entirely ferruginous on mandible, antenna, pronotal lobe, tegula, legs, and pygidial plate. Mandible with apex and preapical tooth darker than all but basal quarter. Antenna brown except F1 extensively orange. Pronotal lobe and tegula pale ferruginous to amber. Wing membrane subhyaline, apically dusky. Legs from tibia to tarsus extensively reddish orange. Pygidial plate orange along apical margin, otherwise dark brown.

*Pubescence.* Face with tomentum densest around antennal socket. Tomentum slightly sparser on clypeus; upper paraocular and frontal areas, and vertexal area mostly exposed. Dorsum of mesosoma and metasoma with bands of off-white to pale yellow short appressed setae. Mesoscutum with anteromedial horseshoe-shaped patch of pale tomentum. Mesopleuron densely hairy, except for two sparsely hairy circular patches (one behind pronotal lobe, a larger one occupying much of ventrolateral half of mesopleuron). Metanotum with tomentum uninterrupted, pale yellow laterally and black medially. T1 with median elliptical verging on semicircular discal patch. T1–T3 with apical fasciae medially interrupted, narrowed (broader laterally), and removed from apical margin; T2 with fascia without anterolateral extensions of tomentum. T4–T6 with fasciae complete, those of T4 and T5 somewhat narrowed medially. S4 and S5 with long coppery to silvery subapical hairs, which individually are often darker apically.

*Surface sculpture.* Punctures dense. Labrum with larger punctures than clypeus, but punctures of both equally dense (i≤1d). Small impunctate shiny spot lateral to lateral ocellus. Mesoscutum, mesoscutellum, and axilla coarsely and densely rugose-punctate. Tegula very densely punctate mesally (i<1d), less so laterally (i=1–2d). Mesopleuron with ventrolateral half coarsely and densely punctate (i<1d) to rugose; mesopleuron with punctures more or less equally dense throughout (only few i=1d ventrolaterally). Metasomal terga with punctures very fine, dense (i≈1d), evenly distributed on disc.

*Structure.* Labral apex with pair of small denticles, each preceded by longitudinal carina. Frontal keel not strongly raised. Scape with greatest length 1.8 × greatest width. F2 as long as wide (L/W ratio = 1.0). Preoccipital ridge not joining hypostomal carina, from which it is separated by about 1.5 MOD at its terminal (difficult to see in holotype; described from paratypes). Mesoscutellum weakly bigibbous. Axilla small to intermediate in size, its lateral margin (L) less than half as long as mesoscutellar width (W) (L/W ratio = 0.4) and tip not extending much beyond midlength of mesoscutellum (extending to <2/3 its length); axilla with tip clearly visible, but unattached to mesoscutellum for less than 2/5 the medial length of axilla; axilla with lateral margin relatively straight and without carina. Fore wing with three submarginal cells. Pygidial plate apically rounded, with large deep punctures closely clustered medially and sparser laterally, with the interspaces shining.

FEMALE: Description as for male except for usual secondary sexual characters and as follows: F2 slightly but not noticeably longer than wide (L/W ratio = 1.1); T5 with large, nearly continuous patch of pale tomentum bordering and separate from pseudopygidial area present only in female; T5 with pseudopygidial area lunate, its apex more than twice as wide as medial length, indicated by silvery setae on impressed disc of apicomedial region elevated from rest of tergum; S4 and S5 with much shorter hairs (S5 with apical fimbria of coppery to silvery hairs extending beyond apex of sternum by ~1/3 MOD); pygidial plate apically truncate, with small, denser punctures.

**Figure 45.**
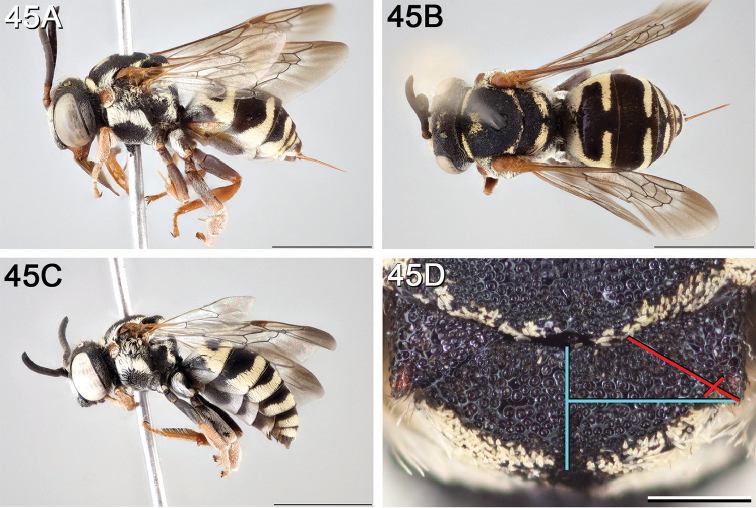
*Epeolus ferrarii* **A** female allotype, lateral habitus (scale bar 3 mm) **B** female allotype, dorsal habitus (scale bar 3 mm) **C** male holotype, lateral habitus (scale bar 3 mm), and **D** female paratype axillae and mesoscutellum, dorsal view (scale bar 0.5 mm; blue lines indicate the posterior extent of the axilla relative to the length of the mesoscutellum; red lines indicate the extent of the free portion of the axilla relative to its entire medial length).

#### Etymology.

This species is named in honor of my colleague, Rafael Ferrari, with whom I collected this species in Southwestern New Mexico, USA.

#### Distribution.

**Figure 46.**
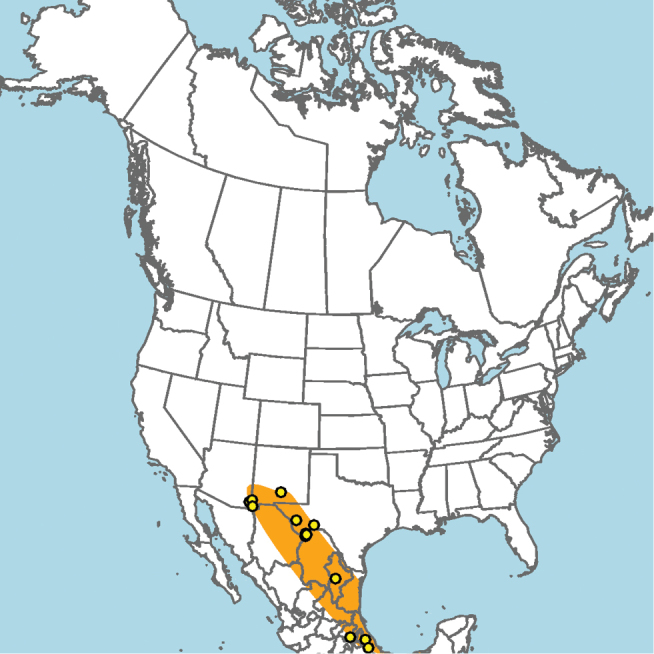
Approximate geographic range of *E. ferrarii* (orange) based on occurrence records known to the author (yellow circles).

Arizona and New Mexico to southeastern Mexico (Fig. 46).

#### Ecology.

HOST RECORDS: The host species of *E. ferrarii* is/are presently unknown.

FLORAL RECORDS: Labels of examined voucher specimens indicate a floral association with *Melilotus albus*.

#### Discussion.

*Epeolus ferrarii* is a cryptic species that most closely resembles *E. canadensis* and *E. compactus*, and can only be differentiated from these two species on the basis of very subtle differences in the patterns of pubescence on the metasomal terga. Its status as a separate species is supported by a separate BIN, but unusually its nearest neighbor is *E. splendidus* (a very different species, although presumably in the same species group), from which *E. ferrarii* exhibits a large barcode sequence divergence (3.9%). Although most species of *Epeolus* were described from a female name-bearing type, a male specimen is designated as the holotype of *E. ferrarii* because a barcode-compliant sequence is associated with it and because the collection locality is more precise than for the available female specimens, one of which is herein designated as the allotype.

#### Material studied.

**Type material.** Primary: USA: **New Mexico**: 47 km S Animas (31.5438°N; 108.8757°W) (Co Rd C001), 30.viii.2015, R. Ferrari and T.M. Onuferko (holotype ♂ [CCDB-24583 H08], PCYU).

Secondary: Guatemala: **Zacapa**: San Lorenzo, xi.1986, M. Sharkey (paratype ♂, CNC).

Mexico: **Chiapas**: Yerbabuena (20 mi N Bochil), 21.v.1969, W.R.M. Mason (paratype ♂, CNC); **Hidalgo**: 2 mi N Pachuca, 24.viii.1962, M.G. Naumann (paratype ♀, KUNHM); **Nuevo León**: Cola de Caballo, 20.vi.1976, D. Weems (paratype ♂, FSCA); **Puebla**: 5 mi NE Teziutlán, 20.vi.1961, Univ. Kans. Mex. Expedition (paratype ♀, KUNHM); **Veracruz**: 10 km N Coscomatepec, 09.vii.1974, J.A. Chemsak, E. and J. Linsley, and J. Powell (paratype ♀, EMEC).

USA: **Arizona**: Southwestern Research Station (5 mi W Portal, Cochise County), 01.viii.1956, C. and M. Cazier (paratype ♀, AMNH), 02.viii.1956, C. and M. Cazier (paratype ♀, AMNH); **New Mexico**: 47 km S Animas (31.5438°N; 108.8757°W) (Co Rd C001), 30.viii.2015, R. Ferrari and T.M. Onuferko (paratypes 2♂ (1 barcoded [CCDB-24580 G07]), PCYU), 30.viii.2015, C. Parsons (paratype ♂, PCYU); 5 mi N Alamogordo (Otero County), 24.iv.1965, O.W. Richards (paratype ♀, NHMUK); Granite Gap (18 mi N Rodeo, Hidalgo County), 07.ix.1976, R.M. Bohart (allotype ♀, UCBME), 07.ix.1976, R.M. Bohart (paratypes 1♀, 1♂, UCBME); **Texas**: 23 mi W Fort Davis, 01.vi.1959, W.R.M. Mason (paratype ♀, CNC); Big Bend National Park, 04.vi.1970, C.W. O’Brien (paratype ♀, LACM); Grapevine Spring (Big Bend National Park), 20.v.1959, W.R.M. Mason (paratype ♀, CNC); Dugout Wells (Big Bend National Park), 22.v.1959, J.F. McAlpine (paratypes 3♀, CNC); Sanderson, 28–29.iv.1959, W.R.M. Mason (paratype ♀, CNC).

#### DNA barcoded material with BIN-compliant sequences.

Available. BOLD:ADD6263. See Type material for specimens examined and sequenced (indicated by unique CCDB-plate and well number).

### 22. Epeolus flavofasciatus

Taxon classificationAnimaliaHymenopteraApidae

Smith, 1879

Figs 2C , 47 , 48

Epeolus flavofasciatus Smith, 1879. Descr. New Species Hymen.: 103 (♀, ♂), **new lectotype designation.**
Triepeolus flavofasciatus Cockerell 1904. Ann. Mag. Nat. Hist. 13: 36.
Triepeolus agaricifer Cockerell, 1907c. Ann. Mag. Nat. Hist. 20: 60 (♂).

#### Diagnosis.

The following morphological features in combination can be used to tell *E. flavofasciatus* apart from all other North American *Epeolus*: the dorsum of the mesosoma and metasoma have bright or pale yellow pubescence, the mesoscutum has distinct paramedian bands, the axilla does not attain the midlength of the mesoscutellum, and T1 has a median triangular or semicircular discal patch. *Epeolus canadensis* resembles *E. flavofasciatus* in that the integument is mostly black, the axilla does not attain the midlength of the mesoscutellum, and T1 has a median triangular or semicircular discal patch, but in *E. canadensis* the mesoscutum has a distinct anteromedial patch of pale tomentum instead of paramedian bands. *Epeolus flavofasciatus* is quite large for *Epeolus* (≥9 mm in length), and the pygidial plate of the male is narrower than that in most species, so males may be confused with *Triepeolus*. However, in *E. flavofasciatus* the mandible has a blunt, obtuse preapical tooth, whereas in all *Triepeolus* the mandible is simple.

#### Redescription.

FEMALE: Length 9.6 mm; head length 2.4 mm; head width 3.3 mm; fore wing length 8.5 mm.

*Integument coloration*. Mostly black; notable exceptions as follows: partially to entirely ferruginous on mandible, antenna, pronotal lobe, tegula, axilla, legs, and pygidial plate. Mandible with apex darker than all but extreme base; preapical tooth lighter than mandibular apex (difficult to see in the *E. flavofasciatus* lectotype because mandible closed; described from non-type specimens). Antenna brown except scape, pedicel, and F1 extensively orange. F2 with orange spot basally. Pronotal lobe and tegula pale ferruginous to amber. Wing membrane dusky subhyaline, slightly darker at apex. Legs more extensively reddish orange than brown or black.

*Pubescence*. Face with tomentum densest around antennal socket. Tomentum slightly sparser on clypeus; upper paraocular and frontal areas, and vertexal area mostly exposed. Dorsum of mesosoma and metasoma with bands of off-white and bright to pale yellow short appressed setae. Mesoscutum with paramedian band. Mesopleuron sparsely hairy except mesally with densely hairy sigmoid patch and ventrally. Metanotum with tomentum uninterrupted, uniformly black (uniformly pale yellow in the *E. agaricifer* holotype and multiple non-type specimens, uniformly black or to varying degrees bright or pale yellow laterally and black medially in other non-type specimens). T1 with median semicircular black discal patch enclosed by pale tomentum (basal fascia widely separated medially and with much tomentum rubbed off in the *E. flavofasciatus* lectotype, but conspicuously arched and narrowly interrupted medially in non-type specimens). T2–T4 with fasciae complete, T2 with fascia without anterolateral extensions of tomentum. T5 with two large patches of pale tomentum lateral to and separate from pseudopygidial area. T5 with pseudopygidial area lunate, its apex more than twice as wide as medial length, indicated by silvery setae on disc of apicomedial region elevated from rest of tergum. S5 with apical fimbria of coppery to silvery hairs not extending beyond apex of sternum by much more than 1/4 MOD.

*Surface sculpture*. Punctures dense. Labrum with larger punctures than clypeus, but punctures of both equally dense (i<1d). Small impunctate matte spot lateral to lateral ocellus. Mesoscutum, mesoscutellum, and axilla coarsely and densely rugose-punctate. Tegula very densely punctate mesally (i<1d), less so laterally (i=1–2d). Mesopleuron with ventrolateral half densely punctate (i<1d); mesopleuron with punctures more or less equally dense throughout. Metasomal terga with punctures very fine, dense (i≈1d), evenly distributed on disc.

*Structure*. Labral apex with pair of small denticles preceded by submedial pair of small denticles and separated by shallow concavity. Frontal keel not strongly raised. Scape with greatest length 1.7 × greatest width. F2 noticeably longer than wide (L/W ratio = 1.4). Preoccipital ridge not joining hypostomal carina, from which it is separated by about 1.5–2 MOD at its terminal (difficult to see in the *E. flavofasciatus* lectotype; described from non-type specimens). Mesoscutellum moderately bigibbous. Axilla small to intermediate in size, its lateral margin (L) less than half as long as mesoscutellar width (W) (L/W ratio = 0.4) and tip not extending beyond midlength of mesoscutellum; axilla with tip clearly visible, but unattached to mesoscutellum for less than 1/3 the medial length of axilla; axilla with lateral margin relatively straight and without carina. Fore wing with three submarginal cells. Pygidial plate apically truncate.

MALE: Description as for female except for usual secondary sexual characters and as follows: F2 shorter, but still longer than wide (L/W ratio = 1.2); S3–S5 with much longer coppery to silvery subapical hairs, which individually are often darker apically; pygidial plate unusually narrow (*Triepeolus*-like) and apically rounded, with large deep punctures closely clustered.

**Figure 47.**
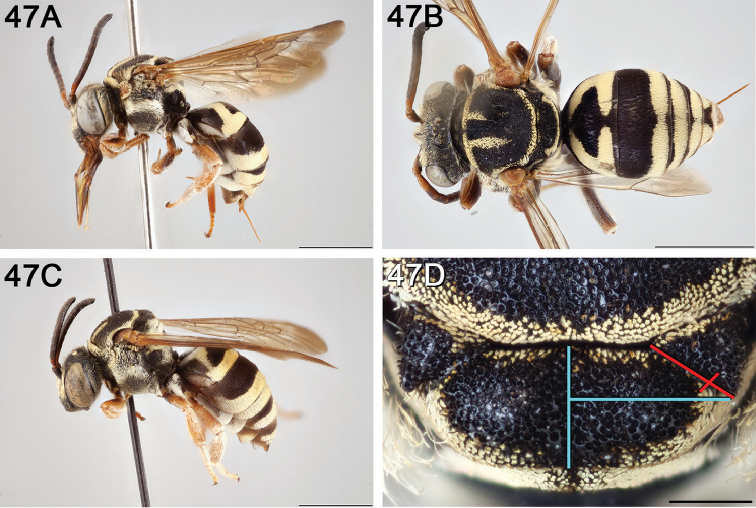
*Epeolus flavofasciatus* **A** female, lateral habitus (scale bar 3 mm) **B** female, dorsal habitus (scale bar 3 mm) **C** male, lateral habitus (scale bar 3 mm), and **D** female axillae and mesoscutellum, dorsal view (scale bar 0.5 mm; blue lines indicate the posterior extent of the axilla relative to the length of the mesoscutellum; red lines indicate the extent of the free portion of the axilla relative to its entire medial length).

#### Distribution.

**Figure 48.**
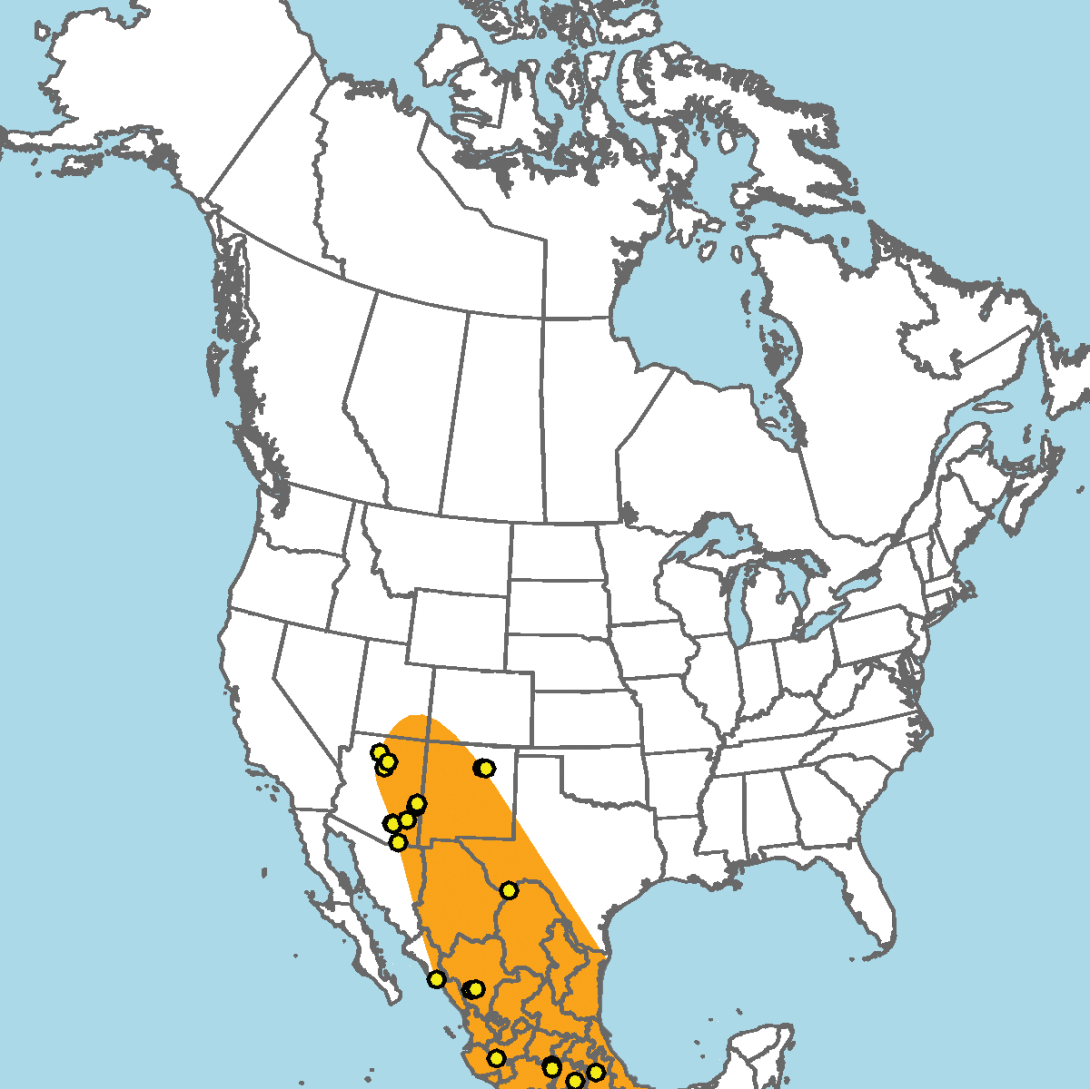
Approximate geographic range of *E. flavofasciatus* (orange) based on occurrence records known to the author (yellow circles).

Mexico, excluding the Baja California Peninsula, and southwestern United States to central America (Fig. 48).

#### Ecology.

HOST RECORDS: The host species of *E. flavofasciatus* is/are presently unknown.

FLORAL RECORDS: Labels of examined voucher specimens indicate floral associations with *Heterotheca subaxillaris* and *Vicia* L. (Leguminosae).

#### Discussion.

Smith (1879) described *E. flavofasciatus* from both sexes, represented by two syntypes (one female and one male) deposited at the NHMUK. Both specimens were examined, and the female is herein designated as the lectotype because it is in better condition, because most *Epeolus* spp. are represented by female name-bearing types, and because Smith (1879) provided a more complete description of the female. The male syntype at the NHMUK is herein designated as the lectoallotype. Cockerell (1907) described this species under the name *Triepeolus agaricifer*, which Rightmyer (2008) synonymized under *E. flavofasciatus*. I have examined the male holotype specimen of *T. agaricifer*, and agree with Rightmyer’s treatment. Two specimens (both males) were barcoded, one of which is from Southeast Arizona, USA (nearer the type locality of *T. agaricifer*: Beulah, New Mexico, USA) and the other is from Jalisco, Mexico (nearer the type locality of *E. flavofasciatus*: Oaxaca, Mexico), and both were assigned the same BIN. Brumley also described this species under the manuscript name *Epeolus artus* [*nomen nudum*] in 1965.

There is some intraspecific variation in the pubescence on the metanotum, which ranges from entirely yellow to medially or mostly black, and T1, in which the apical fascia is either complete or interrupted medially, with differences not conforming to any discernable geographic pattern. Based on examined records, the range of this species appears to be quite continuous from the American Southwest to Central America.

Among the examined specimens of this species is one that appears to be the first known example of bilateral gynandromorphism in *Epeolus* (see Material studied). Descriptions and images of the aberrant features exhibited by the specimen are published separately (Onuferko 2018).

#### Material studied.

**Type material.** Primary: Mexico: **Oaxaca**: (*E. flavofasciatus* lectotype ♀ [NHMUK, catalog number: 010812212]).

USA: **New Mexico**: Beulah, viii.????, T.D. Cockerell (*T. agaricifer* holotype ♂ [USNM, catalog number: 534034]).

Secondary: Mexico: **Oaxaca**: (*E. flavofasciatus* lectoallotype ♂ [NHMUK, catalog number: 010812250]).

#### DNA barcoded material with BIN-compliant sequences.

Available. BOLD:ACZ9233. Specimens examined and sequenced.–Mexico: **Jalisco**: 8 km N Atemajac de Brizuela, 08.x.2008, L. Packer (1♂, PCYU).

USA: **Arizona**: vic. Hannagan Meadow (33.6300°N; 109.3200°W) (Greenlee County), 19–20.vii.1998, B. Harris (1♀, LACM).

#### Non-barcoded material examined.

Guatemala: **Escuintla**: Volcán Pacaya, 30.xi.1975, S.W.T. Batra (1♀, USNM).

Mexico: **Chiapas**: San Cristóbal de las Casas, 29.v.1969, W.R.M. Mason (1♀, CNC); **Durango**: Coyotes (Durango Dist.), 08.viii.1947, D. Rockefeller Exp. Michener (1♀, BBSL);

Navíos (26 mi E El Salto), 02.viii.1964, L.A. Kelton (1♀, CNC); **Michoacán**: 17 mi N Hidalgo, 29.vii.1962, Univ. Kans. Mex. Expedition (2♀, KUNHM); Hidalgo, 12.vii.1963, F.D. Parker and L.A. Stange (1♂, UCBME); **Morelos**: 10 mi N Cuernavaca, 15.viii.1954, Univ. Kans. Mex. Expedition (1♀, KUNHM); **Sinaloa**: Las Palmitas, 13.ix.1977, E.I. Schlinger (2♀, EMEC); **Tlaxcala**: 8 mi WNW Apizaco, 18.vi.1961, Univ. Kans. Mex. Expedition (1♀, KUNHM).

USA: **Arizona**: Catalina Mountains (19 HkHy), 25.vii.1954, G.D. Butler (1♂, KUNHM); Catalina Mountains (24 HkHy), 26.vii.1954, G.D. Butler (1♂, KUNHM); Catalina Mountains (25 HkHy), 14.viii.1954, G. Bohart and G. Butler (1♂, KUNHM); Catalina Mountains (26 HkHy), 14.viii.1954, G. Bohart and G. Butler (1♂, KUNHM), 25.viii.1954, G.D. Butler (1♀, BBSL), 25.viii.1954, G.D. Butler (1♀, KUNHM); Flagstaff (Coconino County), 25.vii.1952, M. Cazier, W. Gertsch, and R. Schrammel (1 chimera, AMNH); Grand Canyon, 19.viii.??39 (1♀, BBSL); Mount Graham (Graham County), 29.viii.1995, J.G. Rozen and S.A. Budick (1♀, AMNH); Pinaleno Mountains (Graham County), 22.viii.1989, Rozen, Foster, and Brewster (1♀, AMNH); Ramsey Canyon (Huachuca Mountains, Cochise County), 1954, W.M. Mann (2♂, USNM); Rose Peak (30 mi N Clifton, Greenlee County), 16.viii.1964, C.D. Michener (1♂, KUNHM); San Francisco Mountains (Flagstaff, Coconino County), 15.viii.1934, E.L. Bell (1♀, AMNH); Santa Catalina Mountains (Pima County), J.L. Neff (1♂, LACM); **New Mexico**: Sapello Canyon (San Miguel County), 26.vii.??02 (1♂, USNM), 27.vii.??02 (1♀, USNM), 31.vii.-01.viii.1963, T.C. Emmel (1♀, LACM); **Texas**: Big Bend National Park (Brewster County), 14.viii.1976, R.T. Ross (1♂, UCBME).

### 23. Epeolus floridensis

Taxon classificationAnimaliaHymenopteraApidae

Mitchell, 1962

Figs 49 , 50 , 97B

Epeolus floridensis Mitchell, 1962. N. C. Agric. Exp. Stn. Tech. Bull. 152: 446 (♀).

#### Diagnosis.

The following morphological features in combination (excluding any that are specific to the opposite sex of the one being diagnosed) can be used to tell *E. floridensis* apart from all other North American *Epeolus*: the axilla is large, with the tip extending as far back as or beyond the posterior margin of the mesoscutellum, dilated laterally, and like the mesoscutellum ferruginous; the mesopleuron is closely (i≤1d) and evenly punctate; T1 is (with few exceptions) ferruginous and with a distinct, although sometimes medially-interrupted, basal fascia; the mesoscutum and metasomal terga have bands of pale gray to white short appressed setae; at least the T1–T3 apical fasciae are distinctly interrupted medially; and the pseudopygidial area of the female is lunate with the apex <2 × the medial length. *Epeolus floridensis* is similar to *E. howardi*, but in *E. howardi* the mesoscutum and metasomal terga have bands of bright or pale yellow short appressed setae and the metasomal terga (including T1) are black. *Epeolus floridensis* is also similar to *E. packeri*, but in *E. packeri* the T1 basal fascia is absent or reduced to a pair of small patches of pale tomentum, the metasomal terga (including T1) are black, and the pseudopygidial area of the female is lunate with the apex >2 × the medial length.

#### Redescription.

FEMALE: Length 7.5 mm; head length 2.1 mm; head width 2.7 mm; fore wing length 5.5 mm.

*Integument coloration.* Black in part, at least partially ferruginous on mandible, labrum, clypeus, antenna, pronotal collar, pronotal lobe, tegula, axilla, mesoscutum, mesoscutellum, metanotum, mesopleuron, metapleuron, propodeum, legs, T1, T5, pygidial plate, and metasomal sterna. Mandible with apex darker than rest of mandible; preapical tooth slightly lighter than mandibular apex. Antenna brown and orange in part. Pronotal lobe and tegula pale ferruginous to amber. Mesoscutum almost entirely reddish brown. Wing membrane subhyaline, apically dusky. Legs more extensively reddish orange than brown or black.

*Pubescence.* Face with tomentum densest around antennal socket. Tomentum slightly sparser on clypeus; upper paraocular and frontal areas, and vertexal area mostly exposed. Dorsum of mesosoma and metasoma with bands of off-white to pale gray short appressed setae. Mesoscutum with paramedian band. Mesopleuron sparsely hairy, but tomentum moderately dense along margins. Metanotum with tomentum uninterrupted, uniformly off white. T1 with discal patch quadrangular and very wide, the basal and apical fasciae only narrowly joined laterally by few sparsely scattered pale hairs. T1–T4 with apical fasciae interrupted medially and somewhat broader laterally, T2 with fascia without anterolateral extensions of tomentum. T5 with two patches of pale tomentum lateral to and contacting pseudopygidial area. T5 with pseudopygidial area lunate, its apex less than twice as wide as medial length, indicated by silvery setae on impressed disc of apicomedial region elevated from rest of tergum. S5 with apical fimbria of coppery to silvery hairs not extending beyond apex of sternum by more than 1/4 MOD.

*Surface sculpture.* Punctures dense. Labrum with larger and sparser punctures (i=1–2d) than clypeus (i<1d). Upper paraocular and frontal areas, and vertexal area with punctures equally dense. Impunctate spot lateral to lateral ocellus absent in holotype, but shiny spot present in non-type specimens. Mesoscutum, mesoscutellum, and axilla coarsely and densely rugose-punctate. Tegula densely punctate mesally (i≤1d), less so laterally (i=1–2d). Mesopleuron with ventrolateral half densely punctate (i≤1d), the interspaces shining; mesopleuron with punctures more or less equally dense throughout. Metasomal terga with punctures very fine, dense (i=1–2d), evenly distributed on disc; the interspaces shining somewhat.

*Structure.* Preapical tooth inconspicuous, blunt and obtuse. Labrum with pair of small subapical denticles not preceded by carinae. Frontal keel not strongly raised. Scape with greatest length 1.8 × greatest width. F2 noticeably longer than wide (L/W ratio = 1.6). Preoccipital ridge not joining hypostomal carina, from which it is separated by about 1.5 MOD at its terminal (difficult to see in holotype; described from non-type specimens). Mesoscutellum weakly bigibbous. Axilla large, its lateral margin (L) more than half as long as mesoscutellar width (W) (L/W ratio = 0.6) and tip extending as far back as apex of horizontal dorsal portion of mesoscutellum; axilla with tip clearly visible, but unattached to mesoscutellum for less than 1/3 the medial length of axilla; axilla with lateral margin arcuate. Fore wing with three submarginal cells. Pygidial plate apically truncate.

MALE: Description as for female except for usual secondary sexual characters and as follows: upper paraocular area very finely and sparsely punctate in part, the interspaces shining; F2 shorter, but still longer than wide (L/W ratio = 1.3); S4 and S5 with much longer coppery to silvery subapical hairs; pygidial plate apically rounded, with large deep punctures closely clustered basomedially and sparser apically and laterally, with the interspaces shining.

**Figure 49.**
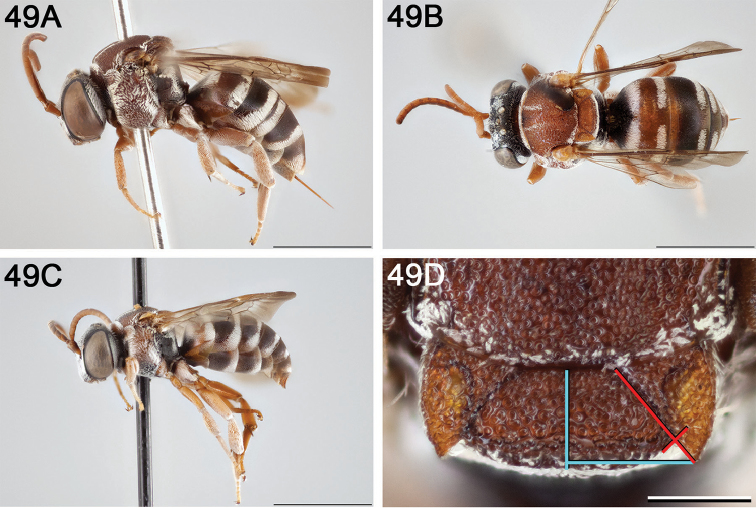
*Epeolus floridensis* **A** female, lateral habitus (scale bar 3 mm) **B** female holotype, dorsal habitus (scale bar 3 mm) **C** male, lateral habitus (scale bar 3 mm), and **D** female axillae and mesoscutellum, dorsal view (scale bar 0.5 mm; blue lines indicate the posterior extent of the axilla relative to the length of the mesoscutellum; red lines indicate the extent of the free portion of the axilla relative to its entire medial length).

#### Distribution.

**Figure 50.**
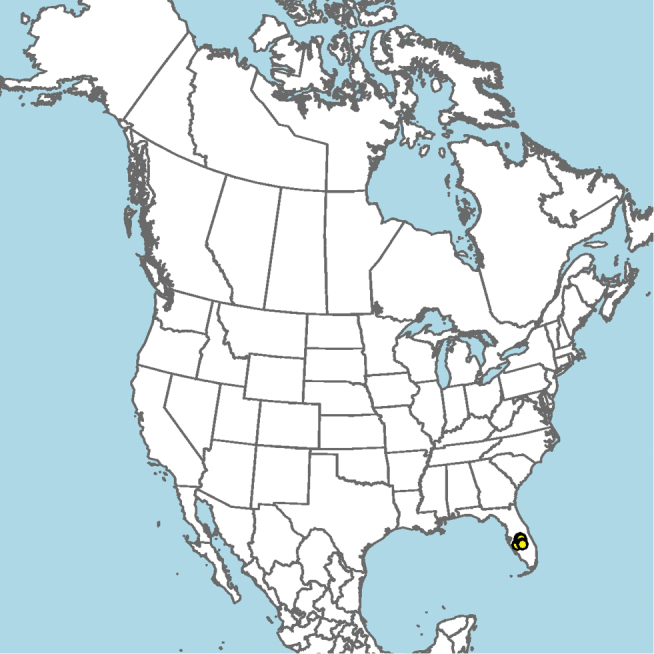
Occurrence records of *E. floridensis* known to the author (yellow circles).

Florida peninsula (Fig. 50).

#### Ecology.

HOST RECORDS: The host species of *E. floridensis* is/are presently unknown.

FLORAL RECORDS: Mitchell (1962) indicated a floral association with *Eriogonum tomentosum* Michx. (Polygonaceae). Labels of examined voucher specimens further indicate associations with *Licania michauxii* Prance (Chrysobalanaceae), *Ptilimnium capillaceum* (Michx.) Raf. (Apiaceae), and *Sabal etonia* Swingle ex Nash (Arecaceae).

#### Discussion.

*Epeolus floridensis* exhibits very little intraspecific morphological variation. However, one specimen was observed in which T1 is as dark as the remaining terga rather than bright ferruginous, the usual state. Also, in some males the upper paraocular area has comparatively fewer punctures than in females while in other specimens punctures are similarly dense between the sexes. Based on examined records, adults of *E. floridensis* appear to be most active in spring, although Mitchell (1962) lists some paratypes that were collected in mid-July.

#### Material studied.

**Type material.** Primary: USA: **Florida**: Arcadia (DeSoto County), 27.iv.1955, H.E. and M.A. Evans (holotype ♀ [CUIC, catalog number: 00015349]).

Secondary: USA: **Florida**: Arcadia (DeSoto County), 27.iv.1955, H.E. and M.A. Evans (allotype ♂ [CUIC, catalog number: 00015348]), 27.iv.1955, H.E. and M.A. Evans (paratypes 1♀, 1♂, NCSU).

#### DNA barcoded material with BIN-compliant sequences.

Available. BOLD:ACZ9059. Specimens examined and sequenced.–USA: **Florida**: Archbold Biological Station (Highlands County), 28.iv.-18.v.2008, S.M. Paiero (1♀, 1♂, DEBU); Lake Placid (Highlands County), 17.v.2014, S. Lenberger (1♀, FSCA).

#### Non-barcoded material examined.

USA: **Florida**: Archbold Biological Station (27.1838°N; 81.3532°W) (Highlands County), 28.v.2010, M. Deyrup (1♀, ABS); Lake Wales Ridge State Forest (27.6611°N; 81.3964°W) (Polk County), 30.iv.2009, M. Deyrup, A. May, and H. Otte (1♀, ABS); Lake Wales Ridge State Forest (27.6933°N; 81.4279°W) (Polk County), 30.iv.2009, M. Deyrup, A. May, and H. Otte (1♂, ABS); Lake Wales Ridge State Forest (27.6915°N; 81.4282°W) (Polk County), 06.v.2009, M. Deyrup, A. May, and H. Otte (1♀, ABS); N FWC Carter Creek (27.5313°N; 81.4104°W) (Highlands County), 15.v.2010, J. Dunlap, M. and N. Deyrup, and K. Dearborn (1♂, ABS); Saddle Blanket Lakes (27.6696°N; 81.5758°W) (Polk County), 07.v.2009, M. Deyrup (1♂, ABS); Saddle Blanket Lakes (27.6716°N; 81.5759°W) (Polk County), 08.v.2009, M. Deyrup, A. May, and H. Otte (1♀, ABS); Walk-In-The-Water State Forest (27.7613°N; 81.4877°W) (Polk County), 29.v.2010, M. Deyrup (1♀, ABS).

### 24. Epeolus gibbsi sp. n.

Taxon classificationAnimaliaHymenopteraApidae

http://zoobank.org/794CED5D-A243-46B5-9E4D-CDAD8CCD3788

**Figure 51.**
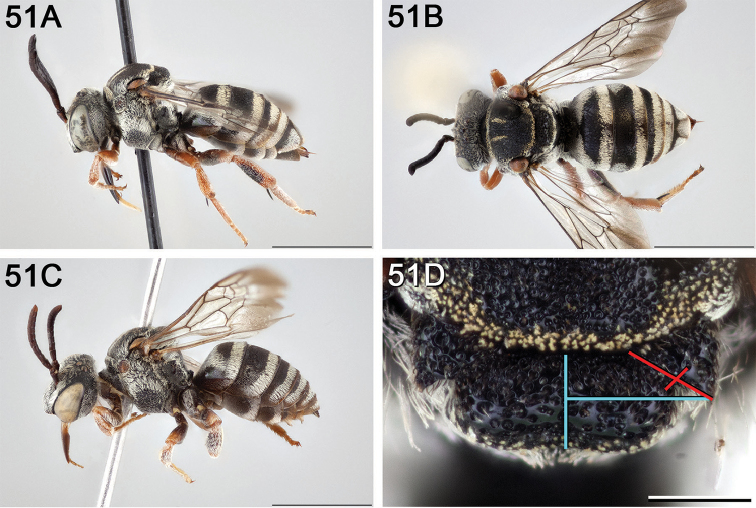
*Epeolus gibbsi* **A** female holotype, lateral habitus (scale bar 3 mm) **B** female holotype, dorsal habitus (scale bar 3 mm) **C** male allotype, lateral habitus (scale bar 3 mm), and **D** female holotype axillae and mesoscutellum, dorsal view (scale bar 0.5 mm; blue lines indicate the posterior extent of the axilla relative to the length of the mesoscutellum; red lines indicate the extent of the free portion of the axilla relative to its entire medial length).

**Figure 52.**
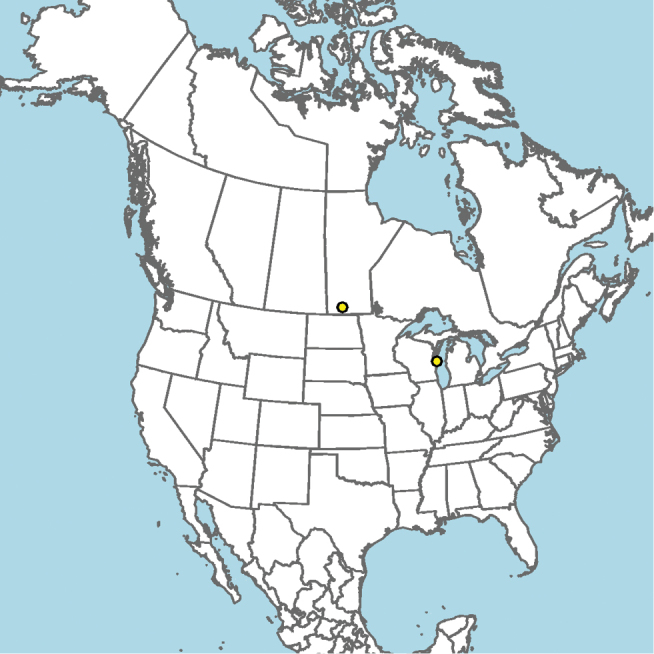
Occurrence records of *E. gibbsi* known to the author (yellow circles).

Figs 3D , 51 , 52 , 96C , 97F

#### Diagnosis.

The following morphological features in combination (excluding any that are specific to the opposite sex of the one being diagnosed) can be used to tell *E. gibbsi* apart from all other North American *Epeolus*: the mandible has a blunt, obtuse preapical tooth; in females, F2 is less than 1.2 × as long as wide; the axilla does not attain the midlength of the mesoscutellum but the free portion is distinctly hooked, with the tip unattached to the mesoscutellum for more than 1/3 of the entire medial length of the axilla; the mesopleuron is closely and evenly punctate (i≤1d), with the interspaces shining and punctures similar in size; the legs are usually darker, at least from the metacoxa to metatibia; the metasomal terga have rather fine punctures; S4 and S5 of the male have long curved coppery to silvery subapical hairs; and the pseudopygidial area of the female is distinctly campanulate with the apex <2 × the medial length and in contact with two large patches of pale tomentum (one on each side [the two are parallel to each other]) throughout its length. *Epeolus gibbsi* most closely resembles *E. ilicis* and *E. inornatus*, but in males of the latter S4 and S5 have short straight subapical hairs and in both *E. ilicis* and *E. inornatus* the mandible is simple, and in females of both species F2 is more than 1.2 × as long as wide and the pseudopygidial area is not in contact with two large patches of pale tomentum (one on each side) throughout its length (in contact only at apex, diverging basally).

#### Description.

FEMALE: Length 7.3 mm; head length 1.9 mm; head width 2.5 mm; fore wing length 5.8 mm.

*Integument coloration*. Mostly black; notable exceptions as follows: partially to entirely ferruginous on mandible, antenna, pronotal lobe, tegula, and legs. Mandible with apex darker than all but extreme base; preapical tooth lighter than mandibular apex (difficult to see in holotype; described from paratype). Antenna dark brown except scape and F1 reddish brown in part. Pronotal lobe dark brown to black. Tegula pale ferruginous to amber. Wing membrane subhyaline, apically dusky. Legs more extensively reddish orange than brown or black.

*Pubescence*. Face with tomentum densest around antennal socket. Tomentum slightly sparser on clypeus; upper paraocular and frontal areas, and vertexal area mostly exposed. Dorsum of mesosoma and metasoma with bands of off-white to pale yellow short appressed setae. Mesoscutum with paramedian band. Mesopleuron densely hairy, except for two sparsely hairy circular patches (one behind pronotal lobe, a larger one occupying much of ventrolateral half of mesopleuron). Metanotum with tomentum uninterrupted except for median bare patch in posterior half, uniformly off white. T1 with median elliptical verging on semicircular discal patch. T1 and T2 with apical fasciae interrupted medially, those of T2 and T3 somewhat broader laterally, T2 with fascia with anterolateral extensions of sparser tomentum. T3 and T4 with fasciae complete. T5 with two large patches of pale tomentum parallel to and contacting pseudopygidial area throughout its length. T5 with pseudopygidial area campanulate, its apex less than twice as wide as medial length, indicated by silvery setae on impressed disc of apicomedial region elevated from rest of tergum. S5 with apical fimbria of coppery to silvery hairs extending beyond apex of sternum by ~1/3 MOD.

*Surface sculpture*. Punctures dense. Labrum with larger punctures than clypeus, but punctures of both equally dense (i<1d). Impunctate spot lateral to lateral ocellus absent in holotype, but shiny spot present in some paratypes. Mesoscutum, mesoscutellum, and axilla coarsely and densely rugose-punctate. Tegula densely punctate (i≤2d). Mesopleuron with ventrolateral half densely punctate (i≤1d), the interspaces shining; mesopleuron with punctures similar in size and more or less equally dense throughout. Metasomal terga with punctures very fine, dense (i=1–2d), evenly distributed on disc; the interspaces shining somewhat.

*Structure*. Preapical tooth blunt and obtuse. Labrum with pair of small subapical denticles not preceded by carinae. Frontal keel not strongly raised. Scape with greatest length 1.8 × greatest width. F2 not noticeably longer than wide (L/W ratio = 1.1). Preoccipital ridge not joining hypostomal carina, from which it is separated by about 1 MOD at its terminal (difficult to see in holotype; described from paratype). Mesoscutellum strongly bigibbous. Axilla small to intermediate in size, its lateral margin (L) less than half as long as mesoscutellar width (W) (L/W ratio = 0.4) and tip attaining midlength of mesoscutellum; axilla with tip conspicuously diverging from side of mesoscutellum, distinctly hooked, and axilla with free portion 2/5 its medial length; axilla with lateral margin relatively straight and without carina. Fore wing with three submarginal cells. Pygidial plate apically truncate.

MALE: Description as for female except for usual secondary sexual characters and as follows: F2 shorter, as long as wide (L/W ratio = 1.0); S4 and S5 with much longer coppery to silvery subapical hairs; pygidial plate apically rounded, with large deep punctures closely clustered.

#### Etymology.

This species is named after its discoverer, Prof. Jason Gibbs, who collected the specimen herein designated as the holotype, recognized it as an unusual find, and brought his discovery to my attention.

#### Distribution.

Upper midwest and adjacent Canada (Fig. 52).

#### Ecology.

HOST RECORDS: The holotype of *E. gibbsi* was collected in an area where *Colletes brevicornis* and *C. kincaidii* were in abundance, the latter of which is likely associated with *E. minimus*, which was also present at the site, as was *E. ainsliei* and its tentative host *C. susannae* (J. Gibbs, personal communication, 2017).

FLORAL RECORDS: Unknown.

#### Discussion.

What Romankova (2004) identified as *E. ilicis*, which constituted a new record of that species in Canada, might actually be *E. gibbsi* and/or *E. inornatus*. Unfortunately, the vouchered material from that study (three specimens from Ontario) cannot be traced, so the presence of *E. ilicis* in Canada has not been confirmed in the present study. *Epeolus ilicis* has been reported from the New England states, though the only examined specimen from that region (a male from Massachusetts) that has been identified as *E. ilicis* (by Richard L. Brumley) appears to actually be *E. inornatus* based on the very short straight subapical hairs on S4 and S5. In Canada, *E. gibbsi* is only confirmed from southern Manitoba, so the specimens from southern Ontario studied by Romankova could represent any of the three species. The key presented in Onuferko (2017) still works for *E. ilicis*, but can also lead to *E. gibbsi* and *E. inornatus* with the modifications presented in Suppl. material 3 starting at couplet 4. Presently, only a single 422 bp sequence is available for *E. ilicis* (a male specimen from Florida, USA), which clusters with sequences of *E. zonatus* (Suppl. material 2), and all were assigned the same BIN. In addition to the diagnostic morphological features that separate *E. gibbsi* from other similar species (notably *E. erigeronis*, *E. ilicis*, and *E. inornatus*, for which only partial sequences 394 to 422 bp in length are available), the status of *E. gibbsi* as a separate species is supported by a separate BIN and large barcode sequence divergence (4.7%) from its nearest neighbor, *E. glabratus*. Based on the few known records, adults of *E. gibbsi* appear to be active in late spring/early summer.

#### Material studied.

**Type material.** Primary: Canada: **Manitoba**: Spruce Woods Provincial Park (49.6630°N; 99.2790°W) (Spirit Sands, Division 7), 07.vii.2017, J. Gibbs and Nozoe (holotype ♀ [CCDB-30345 D02], JBWM).

Secondary: USA: **Wisconsin**: Two Rivers, 26.vi.1911 (allotype ♂, CUM), 26.vi.1911 (paratypes 1♀, 6♂, CUM).

#### DNA barcoded material with BIN-compliant sequences.

Available. BOLD:ADI6791. See Type material for specimens examined and sequenced (indicated by unique CCDB-plate and well number).

### 25. Epeolus glabratus

Taxon classificationAnimaliaHymenopteraApidae

Cresson, 1878

Figs 53 , 54 , 93B

Epeolus glabratus Cresson, 1878. Trans. Am. Entomol. Soc. 7: 90 (♂).
Pyrrhomelecta glabrata Ashmead, 1899. Trans. Am. Entomol. Soc. 26: 66.

#### Diagnosis.

The following morphological features in combination (excluding any that are specific to the opposite sex of the one being diagnosed) can be used to tell *E. glabratus* apart from all other North American *Epeolus* except *E. lectoides*: the axilla is elongate, extending well beyond the midlength of the mesoscutellum but not as far back as its posterior margin, and the free portion is distinctly hooked; the mesopleuron has sparser punctures ventrolaterally (most i>1d) than in upper half, with the interspaces shining; the metasomal terga have minute, shallow punctures; T2–T4 are medially bare; and the pseudopygidial area of the female is distinctly campanulate with the apex <2 × the medial length. Whereas in *E. lectoides* the pronotal collar is black, as are sometimes the axilla and mesoscutellum, and the metasomal terga are black and fasciate, in *E. glabratus* the pronotal collar, axilla, mesoscutellum, and discs of T1 and T2 are ferruginous and the pale pubescence on the metasomal terga are commonly reduced to discrete lateral patches.

#### Redescription.

MALE: Length 8.4 mm; head length 1.8 mm; head width 2.5 mm; fore wing length 7.9 mm.

*Integument coloration.* Black in part, at least partially ferruginous on mandible, labrum, clypeus, antenna, pronotal collar, pronotal lobe, tegula, axilla, mesoscutum, mesoscutellum, mesopleuron, metapleuron, legs, T1, T2, pygidial plate, and metasomal sterna. Mandible with apex darker than rest of mandible; preapical tooth lighter than mandibular apex (difficult to see in holotype because mandible closed; described from non-type specimens). Antenna brown except scape, pedicel, and F1 extensively orange. F2 with orange spot basally. Pronotal lobe and tegula pale ferruginous to amber. Wing membrane dusky subhyaline, slightly darker at apex. Legs more extensively reddish orange than brown or black.

*Pubescence.* Face with tomentum densest around antennal socket. Tomentum slightly sparser on clypeus; upper paraocular and frontal areas, and vertexal area mostly exposed. Dorsum of mesosoma and metasoma with bands of off-white to pale yellow short appressed setae. Mesoscutum with paramedian band. Mesopleuron sparsely hairy, but tomentum dense ventrally as well as between two sparsely hairy patches (one beneath base of fore wing (hypoepimeral area), a larger circular patch occupying much of ventrolateral half of mesopleuron). Metanotum with tomentum uninterrupted except for median bare patch in posterior half, uniformly off white. T1 with discal patch quadrangular and very wide, the basal and apical fasciae only narrowly joined laterally. T1 with basal and apical fasciae and T2–T4 with apical fasciae widely separated medially, the apical fasciae reduced to pairs of small patches somewhat broader laterally; T2 with fascia without anterolateral extensions of tomentum, although few sparsely scattered pale hairs present. Remaining metasomal terga mostly hidden in holotype, but T5 and T6 with complete or narrowly interrupted fasciae in non-type specimens. S4 and S5 with long coppery to silvery subapical hairs.

*Surface sculpture.* Punctures dense, but those of head and mesosoma sparser in some areas, larger, deep, and distinct. Labrum with larger punctures than clypeus, but punctures of both equally dense (i<1d). Small impunctate shiny spot lateral to lateral ocellus. Mesoscutum, mesoscutellum, and axilla very coarsely and densely punctate; the interspaces shining. Tegula densely punctate (i≤2d). Mesopleuron mostly with denser (i≤1d) punctures in upper half than ventrolateral half (i>1d), the interspaces shining. Metasomal terga with punctures very fine, dense (i≥1d), somewhat evenly distributed on disc; the interspaces shining somewhat.

*Structure.* Preapical tooth blunt and obtuse. Labrum with pair of small subapical denticles, each preceded by small discrete longitudinal ridge. Frontal keel not strongly raised. Scape with greatest length 1.7 × greatest width. F2 noticeably longer than wide (L/W ratio = 1.3). Preoccipital ridge not joining hypostomal carina, from which it is separated by less than 1 MOD at its terminal (difficult to see in holotype; described from non-type specimens). Mesoscutellum moderately bigibbous. Axilla large, its lateral margin (L) more than half as long as mesoscutellar width (W) (L/W ratio = 0.6) and tip extending well beyond midlength of mesoscutellum but not as far back as its posterior margin; axilla with tip conspicuously diverging from side of mesoscutellum, distinctly hooked, and axilla with free portion 2/5 its medial length; axilla with lateral margin relatively straight and with tip carinate. Fore wing with three submarginal cells. Pygidial plate mostly hidden in holotype, but apically rounded, with large deep punctures more or less evenly spaced throughout with the interspaces shining in non-type specimens.

FEMALE: Description as for male except for usual secondary sexual characters and as follows: F2 even longer than wide (L/W ratio = 1.5); T5 with two large patches of pale tomentum lateral to and separate from pseudopygidial area present only in female; T5 with pseudopygidial area campanulate, its apex less than twice as wide as medial length, indicated by silvery setae on impressed disc of apicomedial region elevated from rest of tergum; S4 and S5 with much shorter hairs (S5 with apical fimbria of coppery to silvery hairs extending beyond apex of sternum by ~1/3 MOD); pygidial plate apically truncate, with small, denser punctures.

**Figure 53.**
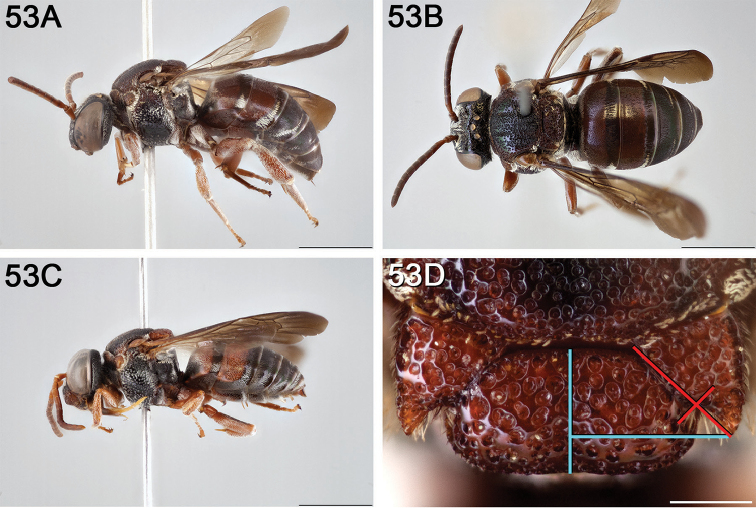
*Epeolus glabratus* **A** female, lateral habitus (scale bar 3 mm) **B** female, dorsal habitus (scale bar 3 mm) **C** male, lateral habitus (scale bar 3 mm), and **D** female axillae and mesoscutellum, dorsal view (scale bar 0.5 mm; blue lines indicate the posterior extent of the axilla relative to the length of the mesoscutellum; red lines indicate the extent of the free portion of the axilla relative to its entire medial length).

#### Distribution.

**Figure 54.**
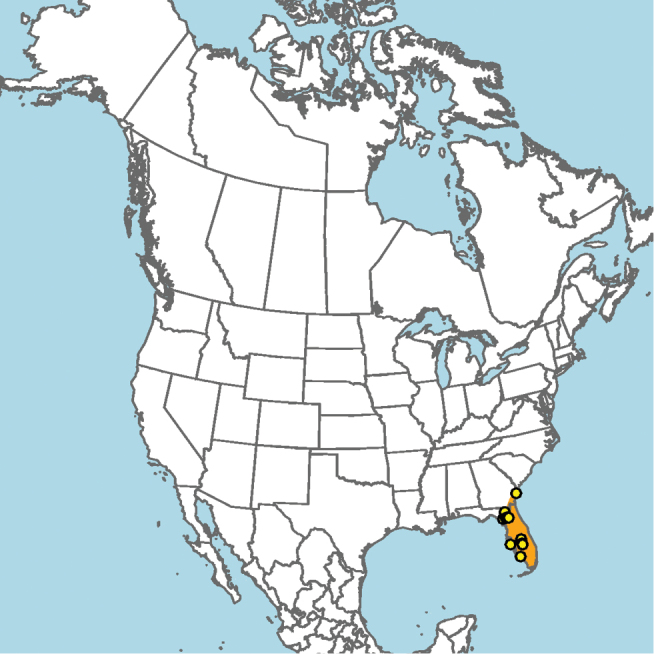
Approximate geographic range of *E. glabratus* (orange) based on occurrence records known to the author (yellow circles).

Florida and coastal Georgia (Fig. 54).

#### Ecology.

HOST RECORDS: The host species of *E. glabratus* is/are presently unknown.

FLORAL RECORDS: Mitchell (1962) indicated a floral association with *Vicia*. Labels of examined voucher specimens further indicate associations with *Coreopsis* L., *Hyptis mutabilis* (Rich.) Briq. (Lamiaceae), *Ilex glabra*, *Pluchea odorata* (L.) Cass. (Compositae), *Polygonella myriophylla* (Small) Horton (Polygonaceae), *Richardia brasiliensis* Gomes (Rubiaceae), *Serenoa repens* (W. Bartram) Small (Arecaceae), *Spermacoce verticillata* L., and *Verbena brasiliensis* Vell.

#### Discussion.

Sequenced specimens of *E. glabratus* share the same BIN as those of *E. lectoides*. There is virtually no divergence (<1%) between the barcode sequences of the two species, but the morphological differences are pronounced. Structurally, *E. glabratus* and *E. lectoides* are identical, but in *E. glabratus* the pronotal collar, axilla, mesoscutellum, and discs of T1 and T2 are ferruginous, whereas in *E. lectoides* at least the pronotal collar and metasomal terga are entirely black. *Epeolus glabratus* appears to be restricted to Florida and parts of Georgia, and the prevalence of red integument coloration among Florida Hymenoptera is a well-known unexplained phenomenon (Deyrup and Eisner 2003). Except in some examined specimens from Georgia, in *E. glabratus* the metasomal fasciae are lacking; the pale pubescence is instead reduced to discrete lateral patches. By contrast, in *E. lectoides* the metasomal terga are always fasciate. Although both species inhabit Florida, *E. glabratus* (with red coloration and reduced pubescence on the metasomal terga) appears to be present only on the peninsula whereas *E. lectoides* (with fasciae and black metasomal terga) appears to be restricted to the Florida panhandle. Since the marked abundance of red coloration is coupled with a general loss of pubescence in *E. glabratus*, and since these are features restricted to specimens from a particular geographical region, I have opted to treat *E. glabratus* and *E. lectoides* as heterospecific, despite the lack of evidence of genetic divergence.

#### Material studied.

**Type material.** Primary: USA: **Georgia**: H.K. Morrison (holotype ♂ [ANSP, catalog number: 2230]).

#### DNA barcoded material with BIN-compliant sequences.

Available. BOLD:AAF2273. Specimens examined and sequenced.–USA: **Florida**: Archbold Biological Station (27.1711°N; 81.3483°W) (Highlands County), 21–26.iv.2011, R.J. Pivar (1♂, DEBU); Archbold Biological Station (Highlands County), 07–13.v.1995, C. Darling (1♀, PCYU); N FWC Carter Creek (27.5313°N; 81.4104°W) (Highlands County), 11.v.2010, J. Dunlap, M. and N. Deyrup, and K. Dearborn (1♂, ABS).

#### Non-barcoded material examined.

USA: **Florida**: Archbold Biological Station (Highlands County), 14.iv.1963, J.G and B.L. Rozen (1♀, AMNH); Doyle Conner Bldg (Gainesville, Alachua County), 12.vi.1996, C. Porter (1♀, FSCA), 18.vi.1996, C. Porter (2♀, FSCA), 26.vi.1996, C. Porter (1♂, FSCA); Gainesville (Alachua County), 03–17.vii.1987, BRC Hymenoptera Team (1♀, PCYU), 07.vi.1976, W.H. Pierce (1♀, UCBME), 16.vi.1991, F.J. Santana (1♀, FSCA), 17.vi.1976, W.H. Pierce (1♀, 1♂, UCBME); Lake Alice (29.6442°N; 82.3630°W) (University of Florida, Gainesville, Alachua County), 05.vi.2007, J.S. Ascher and G. Hall (2♀, AMNH); Lake Placid (Highlands County), 17.v.2014, S. Lenberger (1♂, FSCA); Lake Wales Ridge State Forest (27.6611°N; 81.3964°W) (Polk County), 06.v.2009, M. Deyrup, A. May, and H. Otte (1♀, ABS); Naples (Golden Gate Estates Subdivision, Collier County), 25.v.2013, S. Lenberger (1♂, FSCA); Near Wilcox (Gilchrist County), 27.v.1981, C. Porter, L. Stange, and H. Greenbaum (1♀, FSCA); Newberry (Alachua County), 15.vii.1973, E.E. Grissell (1♂, UCBME); Royal Palm Park, 12–18.iv.1923 (1♂, AMNH); San Felasco State Hammock Preserve, 20.v.1977, G.B. Fairchild and H.V. Weems, Jr. (1♂, UCBME); Sarasota (Sarasota County), 31.v.1993, F.J. Santana (2♀, FSCA); U.S. Highway 41 S Lake City (Columbia County), 19.vi.2014, S. Lenberger (2♀, FSCA); **Georgia**: St Catherines Island (Liberty County), 10–15.v.1991, E. Quinter and A. Sharkov (1♂, AMNH); St Catherines Island (South Beach, Liberty County), 27.vi.1974, R.O. Schuster and E.C. Teftner (1♀, UCBME).

### 26. Epeolus howardi

Taxon classificationAnimaliaHymenopteraApidae

Mitchell, 1962

Figs 55 , 56

Epeolus howardi Mitchell, 1962. N. C. Agric. Exp. Stn. Tech. Bull. 152: 447 (♀).

#### Diagnosis.

The following morphological features in combination (excluding any that are specific to the opposite sex of the one being diagnosed) can be used to tell *E. howardi* apart from all other North American *Epeolus*: the axilla is large, with the tip extending as far back as or beyond the posterior margin of the mesoscutellum, dilated laterally, and like the mesoscutellum ferruginous; the mesopleuron is closely (i≤1d) and evenly punctate; the metasomal terga are black; T1 has a distinct, although sometimes medially-interrupted, basal fascia; the mesoscutum and metasomal terga have bands of bright or pale yellow short appressed setae; at least the T1–T3 apical fasciae are distinctly interrupted medially; and the pseudopygidial area of the female is lunate with the apex <2 × the medial length. *Epeolus howardi* most closely resembles *E. andriyi* and *E. floridensis*, but in *E. andriyi* the axillae are shorter, not extending as far back as the posterior margin of the mesoscutellum, and in *E. floridensis* the mesoscutum and metasomal terga have bands of pale gray to white short appressed setae and T1 is (with few exceptions) ferruginous. *Epeolus howardi* is also similar to *E. scutellaris*, but in *E. scutellaris* the T1–T3 apical fasciae are complete or only very narrowly interrupted medially, and the pseudopygidial area of the female is lunate with the apex >2 × the medial length.

#### Redescription.

FEMALE: Length 8.6 mm; head length 2.2 mm; head width 2.9 mm; fore wing length 6.0 mm.

*Integument coloration.* Black in part, at least partially ferruginous on mandible, labrum, clypeus, antenna, pronotal collar, pronotal lobe, tegula, axilla, mesoscutum, mesoscutellum, metanotum, mesopleuron, legs, T1, pygidial plate, and metasomal sterna. Mandible with apex darker than rest of mandible; preapical tooth slightly lighter than mandibular apex. Antenna brown and orange in part. Pronotal lobe and tegula pale ferruginous to amber. Mesoscutum reddish brown along lateral margin and with pair of reddish-brown markings near posterior margin between midline and parapsidal line. Wing membrane dusky subhyaline, slightly darker at apex. Legs more extensively reddish orange than brown or black. T1 dark in general, not contrasting strongly with remaining metasomal terga, but reddish brown laterally.

*Pubescence.* Face with tomentum densest around antennal socket. Clypeus, upper paraocular and frontal areas, and vertexal area mostly exposed. Dorsum of mesosoma and metasoma with bands of off-white to pale yellow short appressed setae. Mesoscutum with paramedian band. Mesopleuron sparsely hairy, but tomentum moderately dense along margins. Metanotum with tomentum uninterrupted, uniformly off white. T1 with discal patch quadrangular and very wide, the basal and apical fasciae only narrowly joined laterally by few sparsely scattered pale hairs. T1–T4 with apical fasciae interrupted medially and narrowed before becoming somewhat broader laterally, T2 with fascia without anterolateral extensions of tomentum. T5 with two patches of pale tomentum lateral to and contacting pseudopygidial area. T5 with pseudopygidial area lunate, its apex less than twice as wide as medial length, indicated by silvery setae on impressed disc of apicomedial region elevated from rest of tergum. S5 with apical fimbria of coppery to silvery hairs not extending beyond apex of sternum by more than 1/4 MOD.

*Surface sculpture.* Punctures dense. Labrum with larger punctures than clypeus, but punctures of both equally dense (i<1d). Upper paraocular area sparsely punctate in part, the interspaces shining. Small impunctate shiny spot lateral to lateral ocellus. Mesoscutum, mesoscutellum, and axilla coarsely and densely rugose-punctate. Tegula densely punctate mesally (i≤1d), less so laterally (i=1–2d). Mesopleuron with denser (i≤1d) punctures in upper half than ventrolateral half (i≤2d), the interspaces shining. Metasomal terga with punctures very fine, dense (i≈1d), evenly distributed on disc; the interspaces shining somewhat.

*Structure.* Preapical tooth inconspicuous, blunt and obtuse. Labrum with pair of small subapical denticles, each preceded by small discrete longitudinal ridge. Frontal keel not strongly raised. Scape with greatest length 1.8 × greatest width. F2 noticeably longer than wide (L/W ratio = 1.7). Preoccipital ridge not joining hypostomal carina, from which it is separated by less than 1 MOD at its terminal. Mesoscutellum weakly bigibbous. Axilla large, its lateral margin (L) more than half as long as mesoscutellar width (W) (L/W ratio = 0.7) and tip extending beyond apex of horizontal dorsal portion of mesoscutellum; axilla with tip clearly visible, but unattached to mesoscutellum for less than 2/5 the medial length of axilla; axilla with lateral margin arcuate. Fore wing with three submarginal cells. Pygidial plate apically truncate.

MALE: Description as for female except for usual secondary sexual characters and as follows: F2 shorter, but still longer than wide (L/W ratio = 1.3); S4 and S5 with much longer coppery to silvery subapical hairs; pygidial plate apically rounded, with large deep punctures more or less evenly spaced throughout, with the interspaces shining.

**Figure 55.**
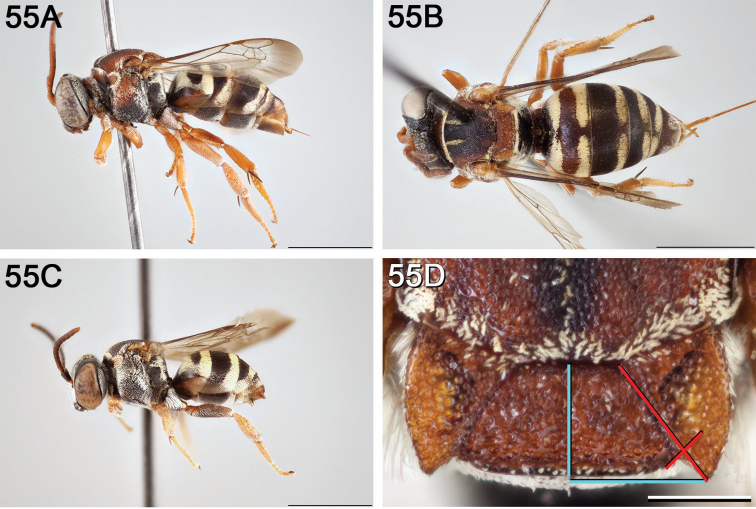
*Epeolus howardi* **A** female, lateral habitus (scale bar 3 mm) **B** female holotype, dorsal habitus (scale bar 3 mm) **C** male, lateral habitus (scale bar 3 mm), and **D** female axillae and mesoscutellum, dorsal view (scale bar 0.5 mm; blue lines indicate the posterior extent of the axilla relative to the length of the mesoscutellum; red lines indicate the extent of the free portion of the axilla relative to its entire medial length).

#### Distribution.

**Figure 56.**
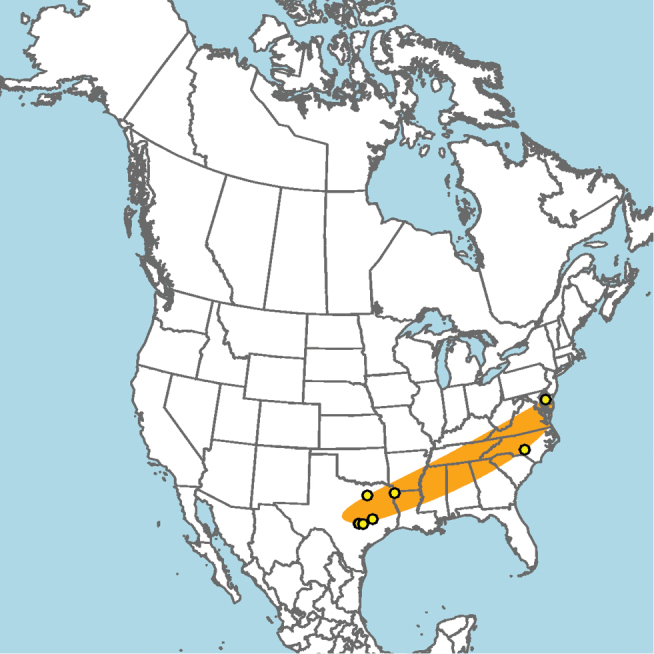
Approximate geographic range of *E. howardi* (orange) based on occurrence records known to the author (yellow circles).

Mid-Atlantic states to Texas (Fig. 56).

#### Ecology.

HOST RECORDS: According to Mitchell (1962), *Colletes howardi* Swenk is the suspected host of *E. howardi*.

FLORAL RECORDS: Mitchell (1962) indicated a floral association with *Dalea pinnata* (J.F.Gmel.) Barneby. Labels of examined voucher specimens further indicate associations with Heterotheca subaxillaris ssp. latifolia (Buckley) Semple, Symphyotrichum drummondii var. texanum (E.S. Burgess) G.L. Nesom, and *Xanthisma texanum* DC. (Compositae).

#### Discussion.

*Epeolus howardi* is a southeastern species that appears to be uncommon, or at least uncommonly collected. In general, there is little morphological variation among examined specimens except in integument coloration; the mesoscutum and mesopleuron range from varying degrees of ferruginous to entirely black, with differences not conforming to any discernable geographic pattern. Based on known records, adults of *E. howardi* are active in late summer and much of autumn.

#### Material studied.

**Type material.** Primary: USA: **North Carolina**: Southern Pines, 30.ix.1951, T.B. Mitchell (holotype ♀ [USNM, catalog number: 534046]).

#### DNA barcoded material with BIN-compliant sequences.

Available. BOLD:ADK0941. Specimens examined and sequenced.–USA: **Maryland**: Denton (38.9196°N; 75.8273°W) (Caroline County), 19.viii.2012, S. Westre (1♂, BIML).

#### Non-barcoded material examined.

USA: **Texas**: Austin (Travis County), 27.x.1981, J.L. Neff (1♀, CTMI); Brackenridge Field Laboratory (Austin, Travis County), 02.xi.1992, J.L. Neff (1♀, CTMI); Brazos County, 24.x.1960, A.H. Alex (1♀, USNM); Dallas, 15.x.??05, F.C. Bishopp (1♀, USNM); Sayersville (Bastrop County), 20.ix.1998, J.L. Neff (1♀, CTMI).

### 27. Epeolus ilicis

Taxon classificationAnimaliaHymenopteraApidae

Mitchell, 1962

**Figure 57.**
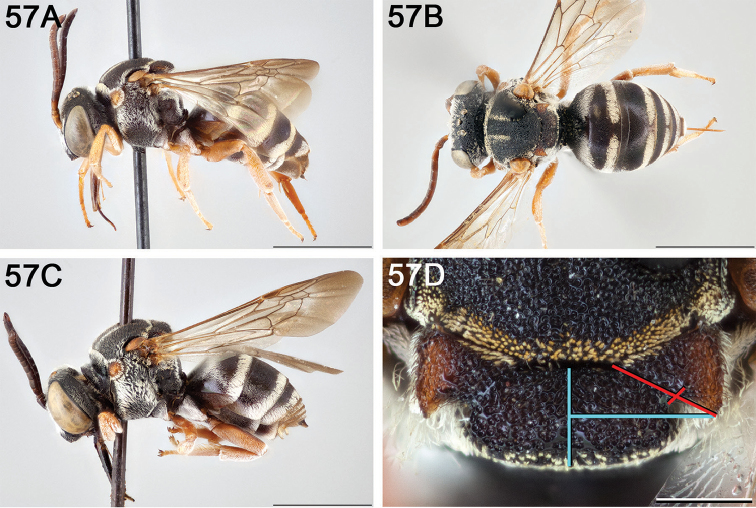
*Epeolus ilicis* **A** female, lateral habitus (scale bar 3 mm) **B** female holotype, dorsal habitus (scale bar 3 mm) **C** male paratype, lateral habitus (scale bar 3 mm), and **D** female axillae and mesoscutellum (photo of *E. vernalis* holotype [herein synonymized under *E. ilicis*]), dorsal view (scale bar 0.5 mm; blue lines indicate the posterior extent of the axilla relative to the length of the mesoscutellum; red lines indicate the extent of the free portion of the axilla relative to its entire medial length).

**Figure 58.**
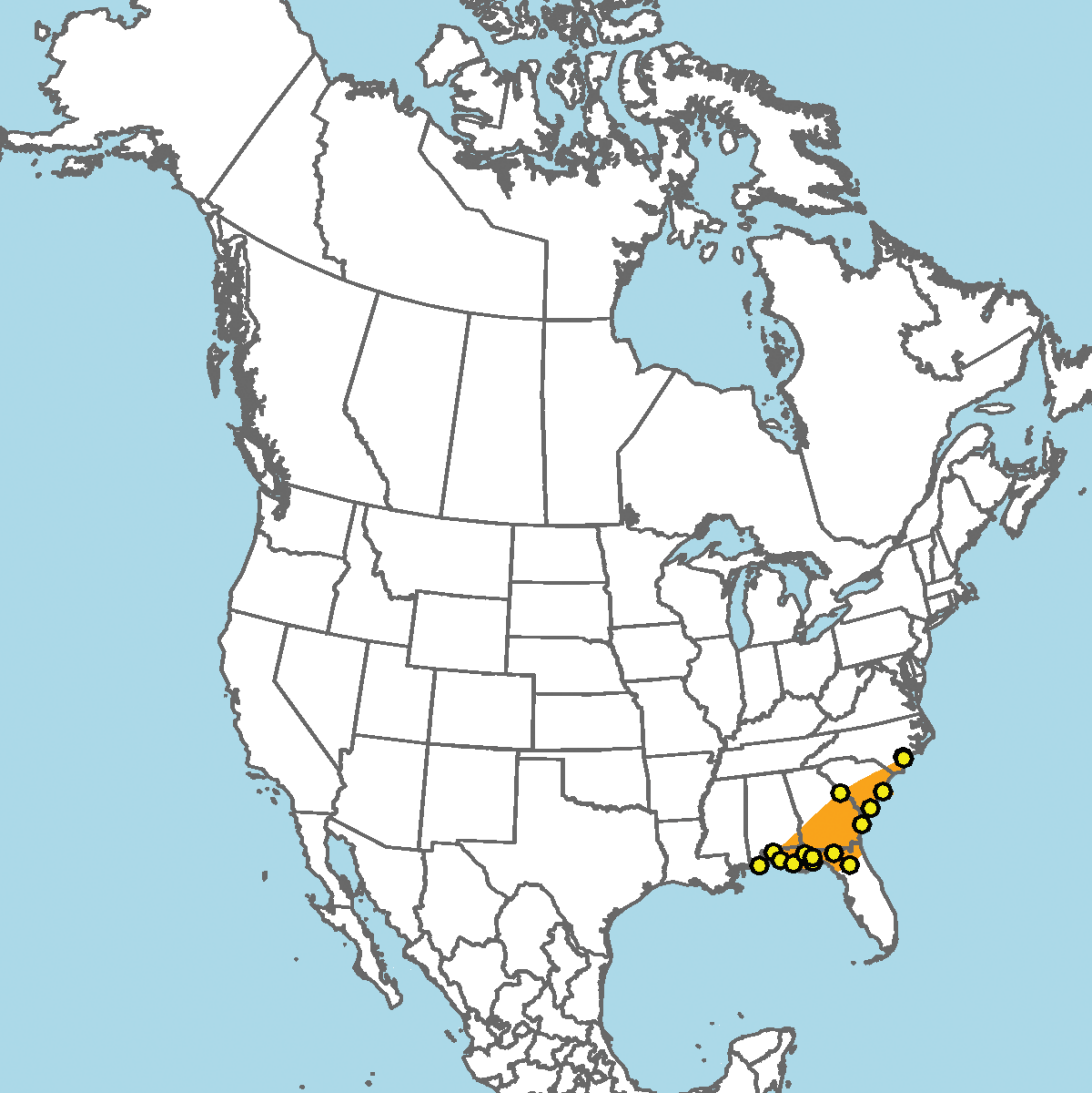
Approximate geographic range of *E. ilicis* (orange) based on occurrence records known to the author (yellow circles).

Figs 3E , 57 , 58 , 92F , 97G , 100A

Epeolus ilicis Mitchell, 1962. N. C. Agric. Exp. Stn. Tech. Bull. 152: 448 (♀).
Epeolus vernalis Mitchell, 1962. N. C. Agric. Exp. Stn. Tech. Bull. 152: 455 (♀), **syn. n.**
Epeolus weemsi Mitchell, 1962. N. C. Agric. Exp. Stn. Tech. Bull. 152: 455 (♂), **syn. n.**

#### Diagnosis.

The following morphological features in combination (excluding any that are specific to the opposite sex of the one being diagnosed) can be used to tell *E. ilicis* apart from all other North American *Epeolus* except *E. erigeronis* and *E. inornatus*: the mandible is simple; the axilla does not attain the midlength of the mesoscutellum but the free portion is distinctly hooked, with the tip unattached to the mesoscutellum for more than 1/3 of the entire medial length of the axilla; the pronotal collar and metasomal terga are black; the metasomal terga have rather fine punctures; and the pseudopygidial area of the female is distinctly campanulate with the apex <2 × the medial length and not in contact with two large patches of pale tomentum (one on each side) throughout its length (in contact only at apex, diverging basally). *Epeolus ilicis* is most similar to *E. inornatus*, and in both species the mesopleuron has punctures that are similar in size and shiny interspaces that are commonly equal to the puncture diameters. By contrast, in *E. erigeronis* the punctures are more variable in size, with many smaller punctures among large ones, and most interspaces are narrower such that the surface appears to be very coarsely and densely rugose-punctate. Whereas in *E. inornatus* the legs (and sometimes the pronotal lobe and tegula) are usually darker, at least from the metacoxa to metatibia, the dorsum of the mesosoma and metasoma have gray short appressed setae, and S4 and S5 of the male have short straight subapical hairs, in *E. ilicis* the pronotal lobe and legs are more extensively reddish orange than brown or black (at least the anterior surface of the metatibia and metatarsus are the same reddish orange color), the dorsum of the mesosoma and metasoma have gray but also usually some pale yellow short appressed setae, and S4 and S5 of the male have long curved coppery to silvery subapical hairs. *Epeolus ilicis* is also similar to *E. gibbsi*, but in *E. gibbsi* the mandible has a blunt, obtuse preapical tooth; in females F2 is less than 1.2 × as long as wide (it is more than 1.2 × as long as wide in female *E. ilicis*); and the pseudopygidial area of the female is in contact with two large patches of pale tomentum (one on each side [the two are parallel to each other]) throughout its length.

#### Redescription.

This species was recently redescribed (Onuferko 2017).

#### Distribution.

Southeastern United States (Fig. 58).

#### Ecology.

HOST RECORDS: Rozen (1989) described first instar *E. ilicis* based on two larvae recovered from the nest of *Colletes brimleyi* Mitchell on St. Catherines Island in Georgia, USA, from where conspecifics of the former have been recorded (see Material studied).

FLORAL RECORDS: Onuferko (2017) lists associations with five plant genera based on Mitchell (1962) and a record on Discover Life (Ascher and Pickering 2017 [then 2016]). Since the discovery of *E. inornatus*, a cryptic species very similar to *E. ilicis* whose name applies to at least one of Mitchell’s paratypes of *E. ilicis* (see Material studied under *E. inornatus*), my taxon concept of *E. ilicis* has changed. As a result, I have only been able to determine that records of *Ilex glabra* and *Prunus angustifolia* Marshall (Rosaceae), taken from the collection labels of the holotypes of *E. ilicis* and *E. weemsi* respectively, are associated with what is here understood to be the true *E. ilicis*.

#### Discussion.

Both the holotype of *E. ilicis* and the holotype of *E. vernalis* were examined, and the two appear to be the same species. In Mitchell’s (1962) key, the two species were differentiated on the basis of whether or not (and if so to what degree) the metasomal fasciae are interrupted medially, but the T1–T3 apical fasciae are interrupted medially (those of T1 and T2 are somewhat more widely separated medially) in both holotype specimens and the T4 fascia is complete in the *E. ilicis* holotype and only very narrowly interrupted in the *E. vernalis* holotype. Moreover, the type locality is the same for both (Holly Shelter [Pender County], North Carolina, USA), and the two specimens were collected only 12 days apart.

Presently, only a single 422 bp sequence is available for *E. ilicis* (a male specimen from Florida, USA), which clusters with sequences of *E. zonatus* (Suppl. material 2), and all were assigned the same BIN. The Florida specimen is most similar to the holotype of *E. weemsi*, which Mitchell (1962) described before noting that it might be the male of *E. vernalis*. In both the sequenced specimen and *E. weemsi* holotype, S4 and S5 have long curved coppery to silvery subapical hairs, which are absent in the very similar *E. inornatus* but present in all other North American male *Epeolus*. Whereas I have opted to treat *E. ilicis* and *E. zonatus* as heterospecific based on remarkably consistent differences in integument coloration coupled with a general loss of pubescence in *E. zonatus*, despite the apparent lack of evidence of genetic divergence, the extremely subtle differences in integument coloration and pubescence among the holotypes of *E. ilicis*, *E. vernalis*, and *E. weemsi* seem to fall within the range of intraspecific variation, and therefore *E. vernalis* and *E. weemsi* are herein synonymized under *E. ilicis*. Although the three names were published simultaneously, priority of the name should be given to *E. ilicis* because the holotype is in the best condition (those of *E. vernalis* and *E. weemsi* have broken antennae and in the latter much of the pubescence is discolored or rubbed off), it is female and most *Epeolus* spp. were described from female name-bearing types (the holotype of *E. weemsi* is male), and because an allotype and paratypes were designated for *E. ilicis* but not *E. vernalis* or *E. weemsi*. This species appears to be quite common in the Southeastern United States, where it may be confused with *E. erigeronis* or *E. inornatus*.

#### Material studied.

**Type material.** Primary: USA: **Florida**: Alachua County, 23.ii.1957, H.V. Weems, Jr. (*E. weemsi* holotype ♂, FSCA); **North Carolina**: Holly Shelter (Pender County), 30.v.1950, T.B. Mitchell (*E. ilicis* holotype ♀ [USNM, catalog number: 534048]), 18.v.1950, T.B. Mitchell (*E. vernalis* holotype ♀ [USNM, catalog number: 534607]).

Secondary: USA: **Georgia**: Fort Gordon (Richmond County), 25.iv.1959, R.R. Snelling (paratype ♂, NCSU); **South Carolina**: McClellanville, 12.v.??44, H.K. Townes (paratype ♂, NCSU), 19.v.??44, H. and G. Townes (paratype ♂, NCSU).

#### DNA barcoded material with BIN-compliant sequences.

Available. BOLD:ACM5887. Specimens examined and sequenced.–USA: **Florida**: Apalachicola National Forest (30.3291°N; 84.5052°W) (Forest Rd 366, Leon County), 15–20.v.2005, A. Deans, S. Joshi, and D. Murray (1♂, AMNH).

#### Non-barcoded material examined.

USA: **Alabama**: Bon Secour National Wildlife Refuge (Baldwin County), 05–07.v.1994, S.A. Marshall (1♀, DEBU); **Florida**: A.T. Slosson (1♀, AMNH); 3 mi NW Sopchoppy (near Sopchoppy River, Wakulla County), 19.iv.1979, G.B. Fairchild (3♀, FSCA); Blackwater River State Forest (4 mi N Munson, Santa Rosa County), 12.vi.1988, L. Stange and J. Wiley (1♀, FSCA); Destin (Okaloosa County), 17.v.1969, H.V. Weems, Jr. (1♀, FSCA); St. Andrews State Park (Bay County), 05–07.v.1987, L. Stange and J. Wiley (2♀, FSCA), 06–07.v.1987, L. Stange and J. Wiley (1♀, 1♂, FSCA); Suwannee River State Park, 13–25.iv.1977, J.R. Wiley (1♂, FSCA); Torreya State Park (Liberty County), 18.v.1970, H.V. Weems, Jr. (1♀, FSCA); **Georgia**: St. Catherines Island (Liberty County), 24–28.iv.1972, Thompson and Picchi (1♂, AMNH), 10–14.iv.1991, J.G. Rozen, E. Quinter, and A. Sharkov (1♀, AMNH); **South Carolina**: Hunting Island State Park (Beaufort County), 08.iv.1963, J.G. and B.L. Rozen (1♂, AMNH).

### 28. Epeolus inornatus sp. n.

Taxon classificationAnimaliaHymenopteraApidae

http://zoobank.org/AFC50A58-43E8-4BC2-A71B-0F85C431B390

**Figure 59.**
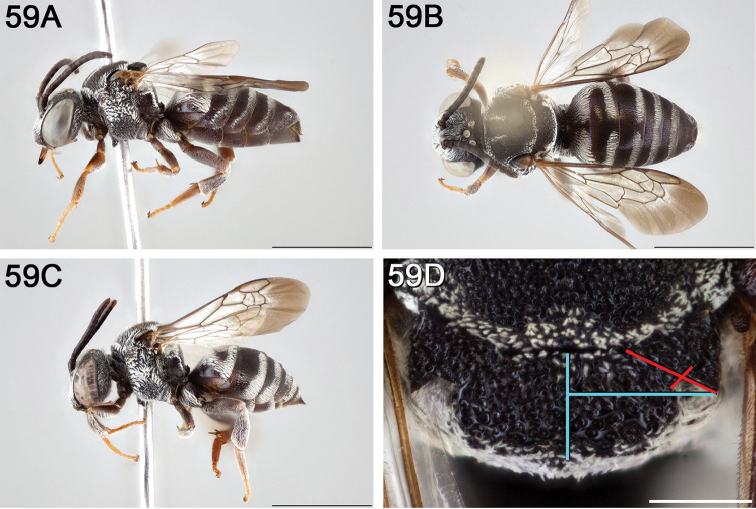
*Epeolus inornatus* **A** female holotype, lateral habitus (scale bar 3 mm) **B** female holotype, dorsal habitus (scale bar 3 mm) **C** male allotype, lateral habitus (scale bar 3 mm), and **D** female paratype axillae and mesoscutellum, dorsal view (scale bar 0.5 mm; blue lines indicate the posterior extent of the axilla relative to the length of the mesoscutellum; red lines indicate the extent of the free portion of the axilla relative to its entire medial length).

**Figure 60.**
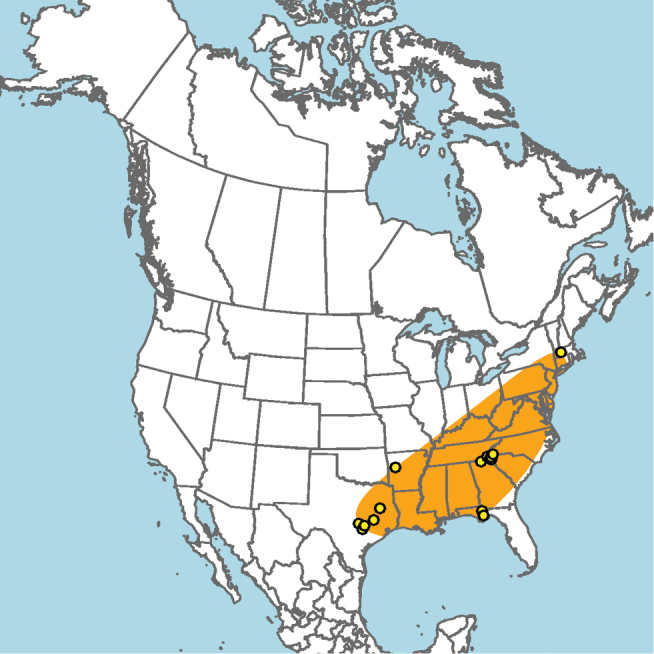
Approximate geographic range of *E. inornatus* (orange) based on occurrence records known to the author (yellow circles).

Figs 59 , 60 , 92G , 93C , 96D , 100B

#### Diagnosis.

The following morphological features in combination (excluding any that are specific to the opposite sex of the one being diagnosed) can be used to tell *E. inornatus* apart from all other North American *Epeolus* except *E. erigeronis* and *E. ilicis*: the mandible is simple; the axilla does not attain the midlength of the mesoscutellum but the free portion is distinctly hooked, with the tip unattached to the mesoscutellum for more than 1/3 of the entire medial length of the axilla; the pronotal collar and metasomal terga are black; the metasomal terga have rather fine punctures; and the pseudopygidial area of the female is distinctly campanulate with the apex <2 × the medial length and not in contact with two large patches of pale tomentum (one on each side) throughout its length (in contact only at apex, diverging basally). *Epeolus inornatus* is most similar to *E. ilicis*, and in both species the mesopleuron has punctures that are similar in size and shiny interspaces that are commonly equal to the puncture diameters. By contrast, in *E. erigeronis* the punctures are more variable in size, with many smaller punctures among large ones, and most interspaces are narrower such that the surface appears to be very coarsely and densely rugose-punctate. Whereas in *E. ilicis* the pronotal lobe and legs are more extensively reddish orange than brown or black (at least the anterior surface of the metatibia and metatarsus are the same reddish orange color), the dorsum of the mesosoma and metasoma have gray but also usually some pale yellow short appressed setae, and S4 and S5 of the male have long curved coppery to silvery subapical hairs, in *E. inornatus* the legs (and sometimes the pronotal lobe and tegula) are usually darker, at least from the metacoxa to metatibia, the dorsum of the mesosoma and metasoma have gray short appressed setae, and S4 and S5 of the male have short straight subapical hairs. *Epeolus inornatus* is also similar to *E. gibbsi*, but in *E. gibbsi* the mandible has a blunt, obtuse preapical tooth; in males S4 and S5 have long curved coppery to silvery subapical hairs, as in *E. ilicis* and all other Nearctic *Epeolus*; in females F2 is less than 1.2 × as long as wide (it is more than 1.2 × as long as wide in female *E. inornatus*); and the pseudopygidial area of the female is in contact with two large patches of pale tomentum (one on each side [the two are parallel to each other]) throughout its length.

#### Description.

FEMALE: Length 8.2 mm; head length 1.9 mm; head width 2.6 mm; fore wing length 5.7 mm.

*Integument coloration*. Mostly black; notable exceptions as follows: partially to entirely ferruginous on mandible, antenna, pronotal lobe, tegula, and legs. Mandible with apex darker than all but extreme base. Antenna dark brown except F1 reddish brown in part. Pronotal lobe dark brown to black. Tegula pale ferruginous to amber. Wing membrane subhyaline, apically dusky. Legs with brown or black more extensive than reddish orange.

*Pubescence*. Face with tomentum densest around antennal socket. Tomentum slightly sparser on clypeus; upper paraocular and frontal areas, and vertexal area mostly exposed. Dorsum of mesosoma and metasoma with bands of off-white to pale gray short appressed setae. Mesoscutum with paramedian band. Mesopleuron with upper half hairy, ventrolateral half nearly bare. Metanotum with tomentum uninterrupted except for median bare patch in posterior half, uniformly off white. T1 with median quadrangular black discal patch enclosed by pale tomentum, except for medial separation at apex. T2 with fascia interrupted medially and with faint anterolateral extensions of sparser tomentum. T3 and T4 with fasciae complete. T5 with two large patches of pale tomentum lateral to and contacting pseudopygidial area at apex, diverging from pseudopygidial area basally. T5 with pseudopygidial area campanulate, its apex less than twice as wide as medial length, indicated by silvery setae on impressed disc of apicomedial region elevated from rest of tergum. S5 with apical fimbria of coppery to silvery hairs not extending beyond apex of sternum by more than 1/4 MOD.

*Surface sculpture*. Punctures dense. Labrum with larger and sparser punctures (i=1–2d) than clypeus (i<1d). Small impunctate shiny spot lateral to lateral ocellus. Mesoscutum, mesoscutellum, and axilla coarsely and densely rugose-punctate. Tegula very densely punctate mesally (i<1d), less so laterally (i=1–2d). Mesopleuron with ventrolateral half densely punctate (i≤1d), the interspaces shining; mesopleuron with punctures similar in size and more or less equally dense throughout. Metasomal terga with punctures very fine, dense (i=1–2d), evenly distributed on disc; the interspaces shining somewhat.

*Structure*. Mandible without preapical tooth. Labrum with pair of small subapical denticles not preceded by carinae. Frontal keel not strongly raised. Scape with greatest length 1.9 × greatest width. F2 noticeably longer than wide (L/W ratio = 1.4). Preoccipital ridge not joining hypostomal carina, from which it is separated by no less than 1 MOD at its terminal. Mesoscutellum moderately bigibbous. Axilla small to intermediate in size, its lateral margin (L) less than half as long as mesoscutellar width (W) (L/W ratio = 0.4) and tip not extending beyond midlength of mesoscutellum; axilla with tip conspicuously diverging from side of mesoscutellum, distinctly hooked, and axilla with free portion 2/5 its medial length; axilla with lateral margin relatively straight and carinate. Fore wing with three submarginal cells. Pygidial plate apically truncate.

MALE: Description as for female except for usual secondary sexual characters and as follows: F2 shorter, not noticeably longer than wide (L/W ratio = 1.1); pygidial plate apically rounded, with large deep punctures closely clustered.

#### Etymology.

The name is in reference to the grayish pubescence and largely monochromatic dark brown or black integument of this species. From the Latin, “inornatus” (unadorned).

#### Distribution.

Mid-Atlantic states to Texas (Fig. 60).

#### Ecology.

HOST RECORDS: The host species of *E. inornatus* is/are presently unknown.

FLORAL RECORDS: Labels of examined voucher specimens indicate floral associations with *Quercus laevis* Walter (Fagaceae) and *Vaccinium arboreum* Marshall.

#### Discussion.

The specimens from Texas, USA that Brumley (1965) identified as *E. ilicis* are probably *E. inornatus*. Although BIN-compliant sequences are presently not available for *E. inornatus*, a single 421 bp sequence is available for a female specimen (the holotype) from East Texas, which does not cluster with the single sequence (422 bp in length) available for what is herein considered to be the true *E. ilicis* (a male specimen from Florida, USA) based on its greater resemblance to the holotype of that species (Suppl. material 2). Instead, the sequence from the Florida specimen clusters with sequences of *E. zonatus*, which is a visibly different bee, and all were assigned the same BIN. Whereas male *E. inornatus* are unique among *Epeolus* in having very short straight subapical hairs on S4 and S5 instead of the usual long curved coppery to silvery subapical hairs, females are practically indistinguishable from *E. ilicis* in terms of surface sculpture and structure. Although consistent, the features (differences in integument coloration and pubescence) that in combination may be used to distinguish female *E. inornatus* from *E. ilicis* are subtle. Based on known records, adults of *E. inornatus* appear to be most active in spring, the same time of year when adults of *E. ilicis* and *E. zonatus* are active.

#### Material studied.

**Type material.** Primary: USA: **Texas**: Lick Creek Park (College Station, Brazos County), 05–09.iv.2000, M. Buck (holotype ♀ [DEBU, catalog number: 00106728]).

Secondary: USA: **Arkansas**: Magazine Mountain (Logan County), 23.v.1991, J. Powell (paratype ♀, EMEC); **Florida**: Liberty County, 24.iv.1961, H.V. Weems, Jr. (paratype ♂, BBSL); Torreya State Park (Liberty County), 12.v.1968, H.V. Weems, Jr. (paratype ♂, FSCA); **Georgia**: 2 mi SE Blue Ridge (Fannin County), 29.vi.1982, J.B. Whitfield (paratype ♂, EMEC); Rabun Bald (Rabun County), 14.vii.1957, J.G. Chillcott (paratype ♀, CNC); Satolah (Rabun County), 01.vii.1957, J.R. Vockeroth (paratype ♀, CNC), 04.vii.1957, W.R.M. Mason (paratype ♂, CNC); **Massachusetts**: Amherst, spring 1929, L.A. Carruth (paratype ♂, USNM); **North Carolina**: Chestnut Bald (Pisgah National Forest, Haywood County), 02.viii.1957, J.G. Chillcott (paratype ♀, CNC); Highlands, 27.vi.1957, W.R.M. Mason (paratype ♀, CNC), 27.vi.1957, J.R. Vockeroth (paratypes 3♂, CNC), 29.vi.1957, J.R. Vockeroth (paratype ♀, CNC), 25.vi.1957, W.R.M. Mason (paratype ♂, CNC); Horse Cove (Highlands), 27.vi.1957, J.R. Vockeroth (paratype ♂, CNC); Wayah Bald (Macon County), 06.vii.1957, W.R.M. Mason (paratype ♀, CNC); Whiteside Mountain (Highlands), 29.vi.1957, W.R.M. Mason (paratype ♀, CNC); **South Carolina**: Mountain Rest, 14.vi.1957, W.R.M. Mason (paratype ♂, CNC); **Texas**: 2.5 mi S Delhi (29.7730°N; 97.4020°W) (Caldwell County), 19.iv.2007, J.L. Neff and A. Hook (paratype ♀, CTMI); 8 km SE Elkhart (Anderson County), 27.iv.1985, C.D. Michener (paratype ♂, KUNHM); Brackenridge Field Laboratory (Austin, Travis County), 13.v.1988, A. Hook (paratype ♂, CTMI); Lick Creek Park (College Station, Brazos County), 05–09.iv.2000, M. Buck (allotype ♂ [DEBU, catalog number: 00106727]); Stengl Lost Pines Biological Research Station (30.0800°N; 97.1830°W) (Bastrop County), 13.iv.2006, J.L. Neff (paratype ♀, CTMI).

#### DNA barcoded material with BIN-compliant sequences.

Unavailable.

#### Non-barcoded material examined.

USA: **North Carolina**: Whiteside Mountain (Macon County), 11.vii.1937, T.B. Mitchell (*E. ilicis* paratype ♂, NCSU).

### 29. Epeolus interruptus

Taxon classificationAnimaliaHymenopteraApidae

Robertson, 1900

Figs 61 , 62

Epeolus interruptus Robertson, 1900. Trans. Acad. Sci. St. Louis 10: 55 (♀).

#### Diagnosis.

Unique to *E. interruptus* among North American species of *Epeolus* are each of the following morphological features: the metanotum has a blunt median process and T1 has a wide triangular discal patch with concave lateral sides. *Epeolus interruptus* most closely resembles *E. tessieris* in that the mesoscutum has short paramedian bands; the axilla does not attain the midlength of the mesoscutellum and like the mesoscutellum is ferruginous (although both are occasionally black in *E. interruptus*); the mesopleuron commonly has sparser punctures ventrolaterally than in upper half, with the interspaces shining; and T1–T4 have medially-interrupted metasomal fasciae. However, in *E. tessieris* the metanotum is flat and T1 has a trapezoidal to nearly semicircular discal patch.

**Figure 61.**
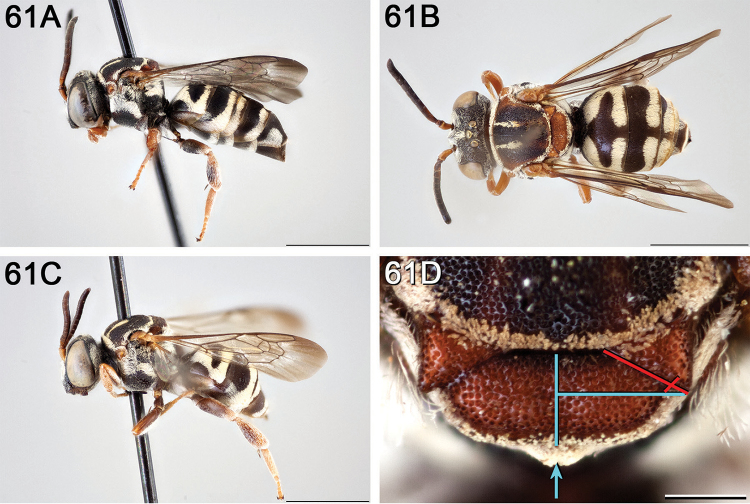
*Epeolus interruptus* **A** female, lateral habitus (scale bar 3 mm) **B** female, dorsal habitus (scale bar 3 mm) **C** male, lateral habitus (scale bar 3 mm), and **D** female axillae and mesoscutellum, dorsal view (scale bar 0.5 mm; blue arrow indicates blunt median process of metanotum; blue lines indicate the posterior extent of the axilla relative to the length of the mesoscutellum; red lines indicate the extent of the free portion of the axilla relative to its entire medial length).

#### Redescription.

This species was recently redescribed (Onuferko 2017).

#### Distribution.

**Figure 62.**
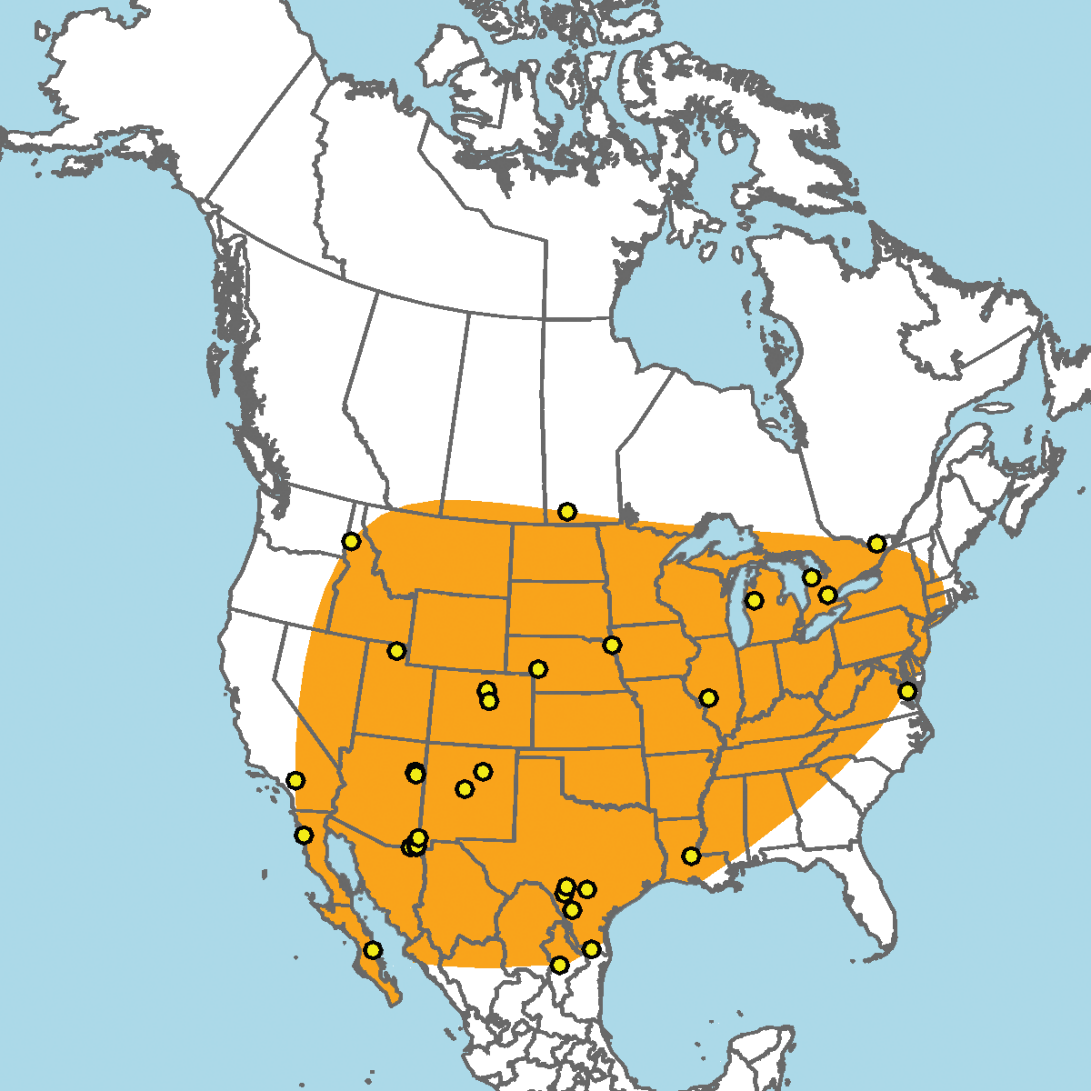
Approximate geographic range of *E. interruptus* (orange) based on occurrence records known to the author (yellow circles).

Central and western Canada, east of the Rocky Mountains, to northern Mexico (Fig. 62).

#### Ecology.

See Onuferko (2017) for host and floral records. Floral associations are also indicated in Suppl. material 1, which includes a newly discovered association with *Heterotheca villosa* (Pursh) Shinners based on the label of one examined voucher specimen.

#### Discussion.

Detailed morphological and taxonomic remarks about this species are given in Onuferko (2017).

#### Material studied.

**Type material.** Primary: USA: **Illinois**: Carlinville (Macoupin County), C.A. Robertson (holotype ♀ [INHS, catalog number: 44384]).

#### DNA barcoded material with BIN-compliant sequences.

Available. BOLD:ACZ9058. Specimens examined and sequenced.–USA: **Arizona**: 3♀, 2♂ (PCYU); **Utah**: 1♂ (BBSL); **Virginia**: 1♀ (CTMI).

#### Non-barcoded material examined.

Canada: **Manitoba**: 6♀ (CNC); **Ontario**: 1♀ (CNC).

Mexico: **Baja California**: 1♂ (EMEC); San Vicente, 08.vii.1963, J.D. Birchim (2♀, CAS); **Baja California Sur**: vic.Est. Microondas “Ligüí” (48 km S Loreto), 07.ix.1977, R.R. Snelling (1♀, 1♂, LACM); **Nuevo León**: Cola de Caballo, 18.vi.1975, H.V. Weems, Jr. (1♂, FSCA).

USA: **Arizona**: 2♀, 4♂ (AMNH, PCYU); 4.7 mi SE Portal (Cochise County), 03.ix.1978, R.E. Coville (1♀, EMEC); **California**: Colton, 26–28.v.1917, E.P. Van Duzee (1♂, CAS); **Colorado**: Boulder, 20.vii.1908, S.A. Rohwer (1♂, CAS); Eldorado Springs, 08.vii.1962, U.N. Lanham (1♂, CUM); Roxborough State Park (39.4356°N; 105.0760°W), 12.vi.2000, A.L. Hicks and V. Scott (1♂, CUM); **Idaho**: 5 mi E Harvard, 21.vii.1971, R.M. Bohart (1♂, UCBME); **Iowa**: 1♀ (AMNH); **Louisiana**: 1♂ (USNM); **Michigan**: G. H. Gordon Biological Station (44.0470°N; 85.6670°W) (Lake County), 28.vi.2015, J. Gibbs (1♂, JBWM); **Nebraska**: 1♀ (AMNH); **New Mexico**: 2♂ (BBSL, FMNH); **Texas**: 3♀ (AMNH, CTMI); 30 mi N Uvalde (Uvalde County), 21.vi.1983, W.J. Pulawski (1♂, CAS); McAllen Botanical Gardens (McAllen), 1973, C.C. Porter (1♂, FSCA), 20.iii.1976, C.C. Porter (1♀, FSCA); **Utah**: 1♂ (BBSL).

### 30. Epeolus lectoides

Taxon classificationAnimaliaHymenopteraApidae

Robertson, 1901

Figs 63 , 64

Epeolus lectoides Robertson, 1901. Can. Entomol. 33: 231 (♀).
Epeolus semilectus Cockerell, 1907a. Entomologist 40: 136 (♂).

#### Diagnosis.

The following morphological features in combination (excluding any that are specific to the opposite sex of the one being diagnosed) can be used to tell *E. lectoides* apart from all other North American *Epeolus* except *E. glabratus*: the axilla is elongate, extending well beyond the midlength of the mesoscutellum but not as far back as its posterior margin, and the free portion is distinctly hooked; the mesopleuron has sparser punctures ventrolaterally (most i>1d) than in upper half, with the interspaces shining; the metasomal terga have minute, shallow punctures; the T2–T4 fasciae are conspicuously narrowed or interrupted medially; and the pseudopygidial area of the female is distinctly campanulate with the apex <2 × the medial length. Whereas in *E. glabratus* the pronotal collar, axilla, mesoscutellum, and discs of T1 and T2 are ferruginous and the pale pubescence on the metasomal terga are commonly reduced to discrete lateral patches, in *E. lectoides* the pronotal collar is black, as are sometimes the axilla and mesoscutellum, and the metasomal terga are black and fasciate. *Epeolus lectoides* is also similar to *E. lectus*, but in *E. lectus* the metasomal terga have coarse, deep punctures and the T2–T4 fasciae are complete and evenly broad.

**Figure 63.**
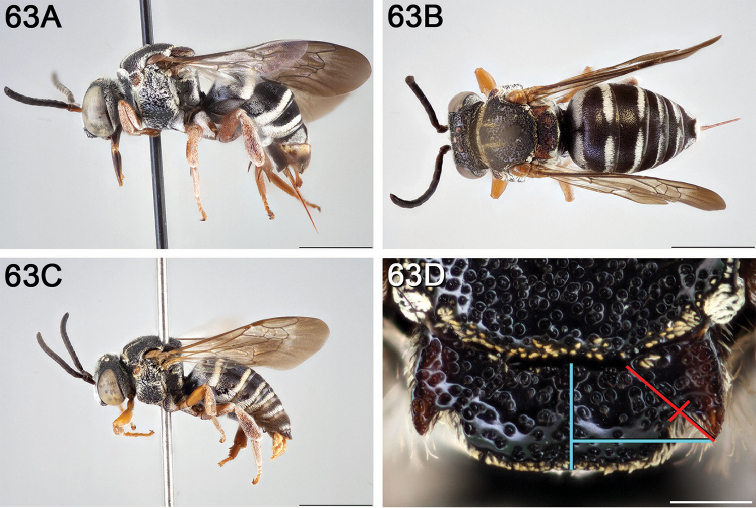
*Epeolus lectoides* **A** female, lateral habitus (scale bar 3 mm) **B** female, dorsal habitus (scale bar 3 mm) **C** male, lateral habitus (scale bar 3 mm), and **D** female axillae and mesoscutellum, dorsal view (scale bar 0.5 mm; blue lines indicate the posterior extent of the axilla relative to the length of the mesoscutellum; red lines indicate the extent of the free portion of the axilla relative to its entire medial length).

#### Redescription.

This species was recently redescribed (Onuferko 2017).

#### Distribution.

**Figure 64.**
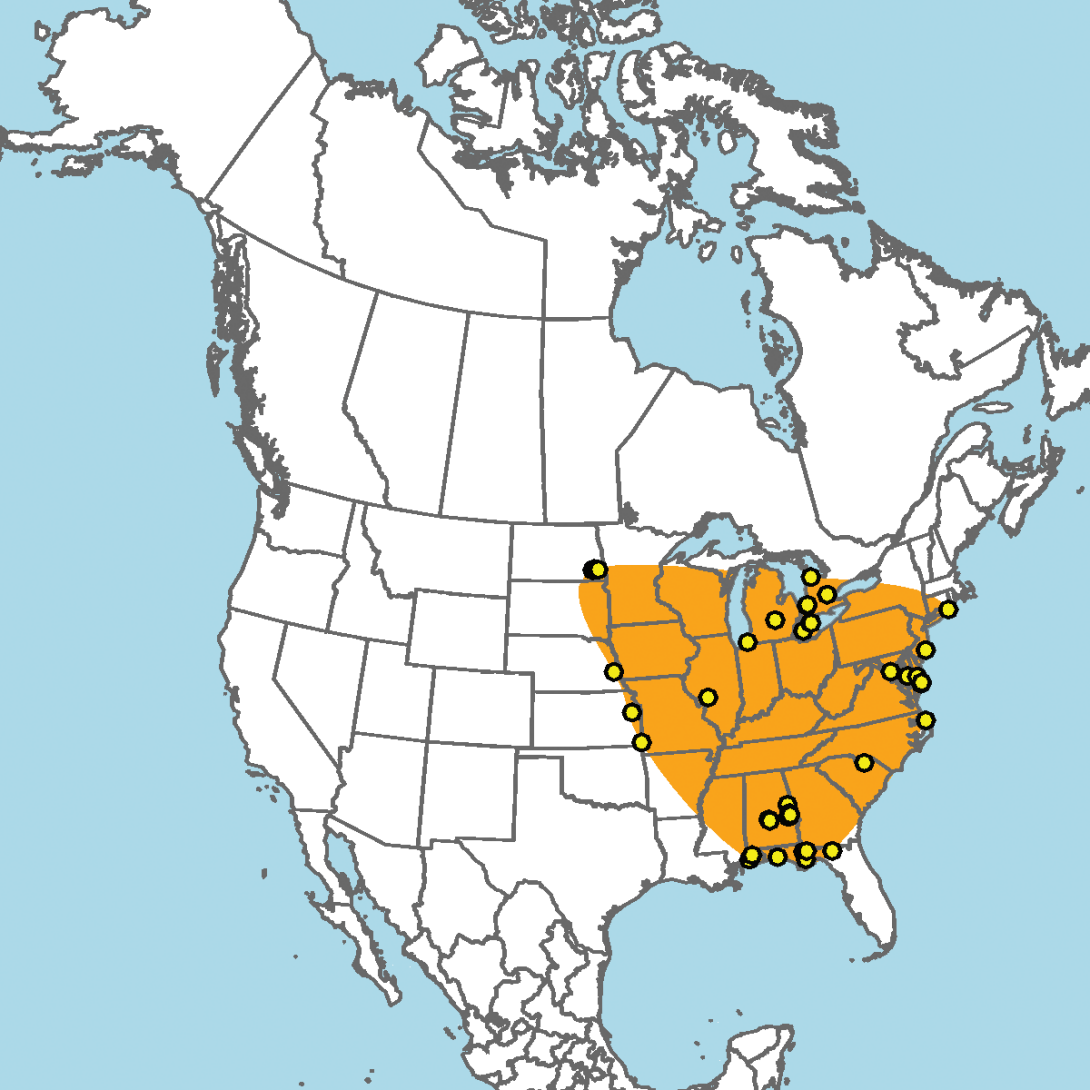
Approximate geographic range of *E. lectoides* (orange) based on occurrence records known to the author (yellow circles).

Eastern North America (Fig. 64).

#### Ecology.

See Onuferko (2017) for host and floral records. Floral associations are also indicated in Suppl. material 1, which includes newly discovered associations with *Aralia spinosa* L. (Araliaceae), *Castanea pumila* (L.) Mill. (Fagaceae), *Helenium amarum* (Raf.) H. Rock (Compositae), *Helianthella* Torr. & A. Gray (Compositae), *Helianthus* L. (Compositae), *Ligustrum* L. (Oleaceae), *Rudbeckia hirta* L. (Compositae), and *Vitex* L. (Lamiaceae) based on labels of examined voucher specimens.

#### Discussion.

Detailed morphological and taxonomic remarks about this species are given in Onuferko (2017).

#### Material studied.

**Type material.** Primary: USA: **Illinois**: Carlinville (Macoupin County), C.A. Robertson (*E. lectoides* holotype ♀ [INHS, catalog number: 44383]); **Virginia**: Falls Church, 04.vii.????, N. Banks (*E. semilectus* holotype ♂ [USNM, catalog number: 534053]).

#### DNA barcoded material with BIN-compliant sequences.

Available. BOLD:AAF2273. Specimens examined and sequenced.–Canada: **Ontario**: 2♂ (DEBU).

USA: **Alabama**: Tuskegee National Forest (32.4800°N; 85.6028°W) (Macon County), 24.vii.2016, C.H. Ray (1♀, 1♂, AUMNH); **Nebraska**: 1♂ (BIML); **South Carolina**: 1♀, 2♂ (PCYU).

#### Non-barcoded material examined.

Canada: **Ontario**: 15♀, 23♂ (DEBU, PCYU, ROM); Rondeau Provincial Park (42.2814°N; 81.8427°W) (Beach Access #10, near Visitor Centre), 08.viii.2017, R. Ferrari (1♀, 1♂, PCYU).

USA: **Alabama**: Auburn University Ornamental Horticulture Research Center (30.7018°N; 88.1454°W), 09.v.2016, Ray, Clem, and Chowdhury (2♂, AUMNH); Auburn (32.5701°N; 85.4603°W) (Lee County), 20.vi.2015, C.H. Ray (2♂, AUMNH); Autauga County (32.4757°N; 86.8597°W), 12.vi.2016, Ray and Chowdhury (2♂, AUMNH); Autauga County (32.3988°N; 86.7918°W), 12.vi.2016, Ray and Chowdhury (1♂, AUMNH); Grand Bay (30.4763°N; 88.3422°W) (Mobile County), 26.v.2010, S. Martin (1♀, AUMNH); Louise Kreher Forest Ecology Preserve (32.6654°N; 85.4845°W), 02.vii.2016, C.H. Ray (1♀, AUMNH); Randolph County (33.1164°N; 85.5435°W), 22.v.2016, C.H. Ray (1♀, AUMNH); Tuskegee National Forest (32.4788°N; 85.5639°W) (Macon County), 28.v.2016, C.H. Ray (2♀, 2♂, AUMNH); Tuskegee National Forest (32.4816°N; 85.6129°W) (Macon County), 13.viii.2016, C.H. Ray (1♀, AUMNH); Tuskegee National Forest (32.4701°N; 85.5840°W) (Macon County), 24.vii.2016, C.H. Ray (1♀, AUMNH); Tuskegee National Forest (32.4800°N; 85.6028°W) (Macon County), 24.vii.2016, C.H. Ray (1♀, 3♂, AUMNH); **Florida**: Greensboro (Gadsden County), 05.vi.2006, S. Lenberger (1♂, FSCA); Liberty County, 06.vi.2006, S. Lenberger (1♂, FSCA); Shalimar (Okaloosa County), 14.vi.2015, F.W. Eliand, II (1♀, AUMNH); Suwannee River State Park, 24.vi.-14.vii.1977, J.R. Wiley (1♂, FSCA); Torreya State Park (Liberty County), 16.v.1964, H.V. Weems, Jr. (1♀, FSCA); **Kansas**: 2♂ (USNM); **Maryland**: 1♀, 1♂ (BIML, DEBU); **Michigan**: Rose Lake State Wildlife Research Area (42.8075°N; 84.3630°W) (Shiawassee County), 04.vii.2014, J. Gibbs (1♂, JBWM), 13.vii.2014, J. Gibbs (1♂, JBWM); Warren Dunes State Park (41.9030°N; 86.6040°W) (Berrien County), 06.vii.2014, J. Gibbs (1♀, JBWM); **New Jersey**: 1♀ (BIML); **New York**: 1♀, 2♂ (AMNH); **North Carolina**: 2♀ (AMNH); **North Dakota**: 1♀ (AMNH); 11 mi W Walcott (Richland County), 12.vii.1990, J.R. Powers (1♀, EMEC); 7 mi SE Sheldon (Ransom County), 02.vii.1988, J.R. Powers (1♀, EMEC); **South Carolina**: 1♀ (BIML); **Virginia**: 1♀, 2♂ (BIML).

### 31. Epeolus lectus

Taxon classificationAnimaliaHymenopteraApidae

Cresson, 1878

**Figure 65.**
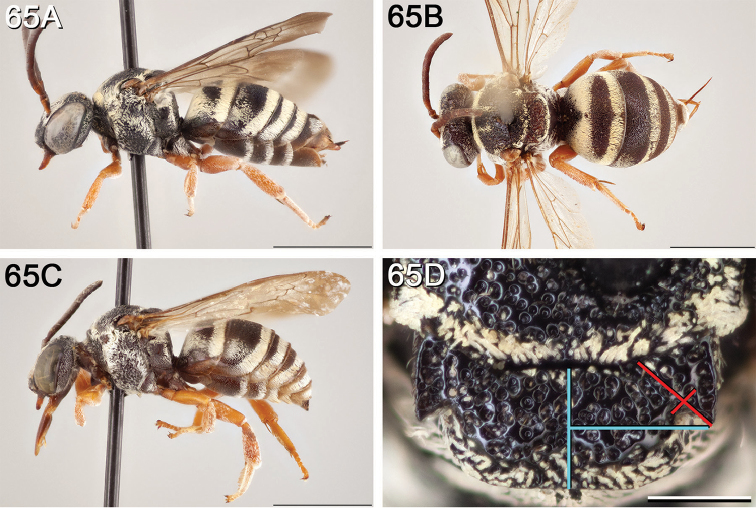
*Epeolus lectus* **A** female, lateral habitus (scale bar 3 mm) **B** female holotype, dorsal habitus (scale bar 3 mm) **C** male, lateral habitus (scale bar 3 mm), and **D** female axillae and mesoscutellum, dorsal view (scale bar 0.5 mm; blue lines indicate the posterior extent of the axilla relative to the length of the mesoscutellum; red lines indicate the extent of the free portion of the axilla relative to its entire medial length).

**Figure 66.**
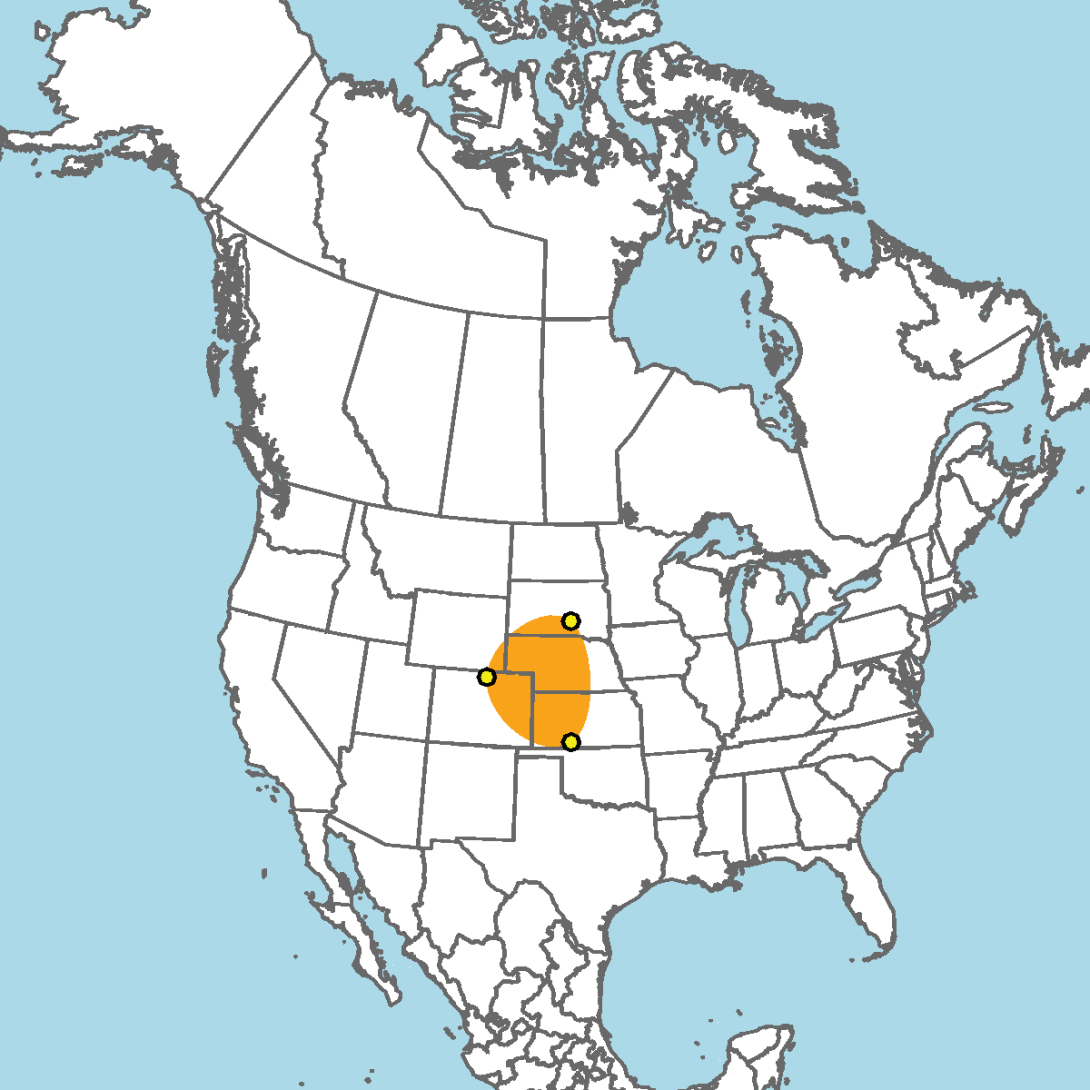
Approximate geographic range of *E. lectus* (orange) based on occurrence records known to the author (yellow circles).

Figs 65 , 66 , 91B , 92A , 93A

Epeolus lectus Cresson, 1878. Trans. Am. Entomol. Soc. 7: 88 (♀).
Epeolus agnatus Cresson, 1878. Trans. Am. Entomol. Soc. 7: 89 (♂).

#### Diagnosis.

The following morphological features in combination can be used to tell *E. lectus* apart from all other North American *Epeolus*: the mesopleuron has sparser punctures ventrolaterally (most i>1d) than in upper half, with the interspaces shining; the metasomal terga have coarse, deep punctures; and T2–T4 have complete and evenly broad fasciae. *Epeolus lectus* is most similar to *E. lectoides*, and in both species the free portion of the axilla is distinctly hooked and the pseudopygidial area of the female is distinctly campanulate with the apex <2 × the medial length, but in *E. lectoides* the metasomal terga have minute, shallow punctures and the T2–T4 fasciae are conspicuously narrowed or interrupted medially.

#### Redescription.

FEMALE: Length 9.2 mm; head length 2.3 mm; head width 3.1 mm; fore wing length 7.2 mm.

*Integument coloration.* Mostly black; notable exceptions as follows: partially to entirely ferruginous on mandible, antenna, tegula, axilla, mesoscutellum, legs, and metasomal sterna. Mandible with apex darker than all but extreme base; preapical tooth lighter than mandibular apex (difficult to see in the *E. lectus* holotype; described from non-type specimens). Flagellum brown and (except F1) slightly lighter than partially dark brown (otherwise orange) scape and F1 and entirely dark brown pedicel, primarily due to extensive pilosity on flagellum. F2 with orange spot basally. Wing membrane dusky subhyaline, slightly darker at apex. Legs from trochanter to tarsus extensively reddish orange, coxae brown.

*Pubescence.* Face with tomentum densest around antennal socket. Tomentum slightly sparser on clypeus; upper paraocular and frontal areas, and vertexal area mostly exposed. Dorsum of mesosoma and metasoma with bands of off-white to pale yellow short appressed setae. Mesoscutum with paramedian band. Mesopleuron with upper half sparsely hairy, ventrolateral half nearly bare. Metanotum with tomentum sparser medially, uniformly off white. T1 with discal patch elliptical and very wide, the basal and apical fasciae only narrowly joined laterally. T1 with basal and apical fasciae and T2–T3 with apical fasciae complete (T4 entirely retracted in the *E. lectus* holotype, but with complete fascia in non-type specimens), T2 with fascia with faint anterolateral extensions of sparser tomentum. T5 with two large patches of pale tomentum lateral to and contacting pseudopygidial area at apex. T5 with pseudopygidial area campanulate, its apex less than twice as wide as medial length, indicated by silvery setae on impressed disc of apicomedial region elevated from rest of tergum. S5 with apical fimbria of coppery to silvery hairs extending beyond apex of sternum by 1/3 MOD.

*Surface sculpture.* Punctures dense, but those of head and mesosoma sparser in some areas, larger, deep, and distinct. Labrum with larger punctures than clypeus, but punctures of both equally dense (i<1d). Small impunctate shiny spot lateral to lateral ocellus. Mesoscutum, mesoscutellum, and axilla very coarsely and densely punctate; the interspaces shining. Tegula very densely punctate mesally (i≤1d), less so laterally (i=1–2d). Upper half of mesopleuron and anterior margin with denser (i≤1d) punctures than rest of mesopleuron (i>1d), the interspaces shining. Metasomal terga with punctures coarse, dense (i≈1d), evenly distributed on disc; the interspaces shining somewhat.

*Structure.* Preapical tooth blunt and obtuse. Labrum with pair of small subapical denticles, each preceded by small discrete longitudinal ridge. Frontal keel not strongly raised. Scape with greatest length 1.7 × greatest width. F2 noticeably longer than wide (L/W ratio = 1.5). Preoccipital ridge not joining hypostomal carina, from which it is separated by no less than 1 MOD at its terminal (difficult to see in the *E. lectus* holotype; described from non-type specimens). Mesoscutellum moderately bigibbous. Axilla large, its lateral margin (L) half as long as mesoscutellar width (W) (L/W ratio = 0.5) and tip not extending much beyond midlength of mesoscutellum (extending to <2/3 its length in the *E. lectus* holotype and all examined non-type specimens; extending to ~2/3 its length in the *E. agnathus* holotype); axilla with tip conspicuously diverging from side of mesoscutellum, distinctly hooked, and axilla with free portion approximately half its medial length; axilla with lateral margin relatively straight and carinate. Fore wing with three submarginal cells. Pygidial plate apically truncate.

MALE: Description as for female except for usual secondary sexual characters and as follows: F2 shorter, but still longer than wide (L/W ratio = 1.2); S4 and S5 with much longer coppery to silvery subapical hairs; pygidial plate apically rounded, with large deep punctures closely clustered medially and sparser laterally, with the interspaces shining.

#### Distribution.

Great Plains and Mountain states east of the Continental Divide (Fig. 66).

#### Ecology.

HOST RECORDS: In late July 2015, I collected several specimens of this species near the Poudre River in the Roosevelt National Forest, Colorado, USA, where large numbers of *Colletes* females were collected and observed foraging on purple *Dalea* flowers. Using Stephen’s (1954) key, collected specimens were identified as being either *C. robertsonii* Dalla Torre or *C. timberlakei* Stephen, the females of which cannot be reliably distinguished morphologically, although the short triangular mesosomal spines and fine punctation on the tegulae of examined specimens coupled with their collection locality suggest they are *C. timberlakei*.

FLORAL RECORDS: The label of one examined voucher specimen indicates a floral association with Cryptantha cinerea var. jamesii (Torr.) Cronquist (Boraginaceae).

#### Discussion.

The names *Epeolus agnathus* and *E. lectus* were published simultaneously, although Cresson (1878) remarked that *E. agnathus* may be the male of *E. lectus* as the two specimens are structurally similar. Robertson (1902) synonymized *E. agnathus* under *E. lectus*, and separated both specimens from *E. lectoides* based on differences in metasomal pubescence and punctation (see diagnosis). I have examined the holotype specimens of *E. lectus* and *E. agnathus*, and agree with Robertson’s treatment. Although Robertson (1902) did not provide any justification for selecting the name *E. lectus* over *E. agnathus*, the holotype of the former is in better condition (that of *E. agnathus* is missing an antenna) and is female, the sex upon which most *Epeolus* species descriptions have been based. While Cresson’s *Epeolus* types include remarkably little collection data, the type locality of *E. agnathus* (Dakota Territory) is even more vague than that of *E. lectus* (Kansas).

In contrast to the similar and presumably closely related *E. lectoides*, *E. lectus* has a much more restricted range and is rare in collections. Both species are known from the Great Plains, although the range of *E. lectus* extends further west. In *E. lectus*, the metasoma has much coarser punctures than that of any other North American species in the genus, including *E. lectoides*, in which the metasoma has much finer and sparser punctures. In addition to this and other clear morphological differences (see diagnosis), the distinction between *E. lectus* and *E. lectoides* is supported by separate BINs for the two species.

#### Material studied.

**Type material.** Primary: USA: **Dakota**: H. Ulke (*E. agnathus* holotype ♂ [ANSP, catalog number: 2226]); **Kansas**: Wilson (*E. lectus* holotype ♀ [ANSP, catalog number: 2225]).

#### DNA barcoded material with BIN-compliant sequences.

Available. BOLD:ACZ8246. Specimens examined and sequenced.–USA: **Colorado**: Bellvue (40.6882°N; 105.3070°W) (N Cache La Poudre River and E Gordon Creek, Larimer County), 28.vii.2015, A.T. and T.M. Onuferko (2♀, PCYU).

#### Non-barcoded material examined.

USA: **Colorado**: Bellvue (40.6882°N; 105.3070°W) (N Cache La Poudre River and E Gordon Creek, Larimer County), 28.vii.2015, A.T. and T.M. Onuferko (3♀, PCYU); **Kansas**: 4 mi NW Coldwater (Comanche County), 12.vi.2002, G.A. Salsbury (1♀, KUNHM); **South Dakota**: Chamberlain (Brule County), 15.vi.1928, H.C. Severin (1♂, USNM).

### 32. Epeolus mesillae

Taxon classificationAnimaliaHymenopteraApidae

(Cockerell, 1895)

Figs 67 , 68 , 91D

Phileremus mesillae Cockerell, 1895. Psyche (suppl.) 7: 10 (♂), **new neotype designation.**
Epeolus mesillae Cockerell, 1934. Am. Mus. Novit. 697: 12.
Epeolus mesillae palmarum Linsley, 1939. Pan-Pac. Entomol. 15: 2 (♀), **syn. n.**

#### Diagnosis.

The following morphological features in combination can be used to tell *E. mesillae* apart from all other North American *Epeolus*: the axilla does not attain the midlength of the mesoscutellum and like the mesoscutellum is black, the fore wing has two submarginal cells, and T1–T4 have complete fasciae. Only in *E. americanus* and *E. asperatus* is the fore wing commonly with two submarginal cells, but in both species at least the T1 and T2 apical fasciae are interrupted or at least greatly narrowed medially. *Epeolus brumleyi* is similar to *E. mesillae* in axillar structure; in that in females F2 is shorter, as long as wide; and in that T1–T4 have complete fasciae. However, in *E. brumleyi* the axilla is commonly ferruginous in part and the fore wing has three submarginal cells.

#### Redescription.

MALE: Length 6.6 mm; head length 1.7 mm; head width 2.4 mm; fore wing length 4.9 mm.

*Integument coloration*. Mostly black; notable exceptions as follows: at least partially ferruginous on mandible, antenna, pronotal lobe, tegula, and legs. Mandible orange between dark brown base and reddish-brown apex; preapical tooth slightly lighter than mandibular apex (difficult to see in the *P. mesillae* neotype because mandible closed; described from non-type specimens). Flagellum brown, except F1 extensively orange, and slightly lighter than dark brown scape and pedicel. Pronotal lobe reddish brown. Tegula pale ferruginous to amber. Wing membrane hyaline throughout. Legs, except tarsi, with brown or black more extensive than reddish orange.

*Pubescence.* Face with tomentum densest on clypeus and around antennal socket, sparser on upper paraocular area and vertexal area. Dorsum of mesosoma and metasoma with bands of off-white to pale yellow short appressed setae. Mesoscutum with paramedian band partly obscured by surrounding pale tomentum. Mesopleuron almost entirely obscured by white tomentum, except where rubbed off in the *P. mesillae* neotype. Metanotum with tomentum uninterrupted, uniformly off white. T1 with discal patch elliptical, narrow, and short. T2–T6 each with complete fascia, those of T2 and T3 somewhat broader laterally, T2 with fascia with anterolateral extensions of sparser tomentum. S3–S5 with long coppery to silvery subapical hairs.

*Surface sculpture*. Punctures dense. Labrum and clypeus with punctures equally dense (i<1d). Small impunctate spot lateral to lateral ocellus. Mesoscutum, mesoscutellum, and axilla coarsely and densely rugose-punctate. Tegula densely punctate mesally (i≤1d), less so laterally (i=1–2d). Mesopleuron with ventrolateral half densely punctate (i<1d) to rugose; mesopleuron with punctures more or less equally dense throughout. Metasomal terga with punctures very fine, dense (i≈1d), evenly distributed on disc.

*Structure*. Labrum with pair of small subapical denticles, each preceded by small discrete longitudinal ridge. Frontal keel not strongly raised. Scape with greatest length 1.7 × greatest width. F2 nearly as long as wide (L/W ratio = 0.9). Preoccipital ridge not joining hypostomal carina, from which it is separated by about 1.5–2 MOD at its terminal (difficult to see in the *P. mesillae* neotype; described from non-type specimens). Mesoscutellum moderately bigibbous. Axilla small to intermediate in size, its lateral margin (L) less than half as long as mesoscutellar width (W) (L/W ratio = 0.3) and tip not extending beyond midlength of mesoscutellum; axilla with tip visible, but unattached to mesoscutellum for less than 1/3 the medial length of axilla; axilla with lateral margin relatively straight and without carina. Fore wing with two submarginal cells. Pygidial plate apically rounded, with large deep punctures closely clustered.

FEMALE: Description as for male except for usual secondary sexual characters and as follows: F2 slightly longer, as long as wide (L/W ratio = 1.0); wing membrane subhyaline, apically dusky; T5 with large, continuous patch of pale tomentum bordering and separate from pseudopygidial area present only in female; T5 with pseudopygidial area lunate, its apex more than twice as wide as medial length, indicated by silvery setae on impressed disc of apicomedial region elevated from rest of tergum; S3–S5 with much shorter hairs (S5 with apical fimbria of coppery to silvery hairs extending beyond apex of sternum by ~2/5 MOD); pygidial plate apically truncate, with small, denser punctures.

**Figure 67.**
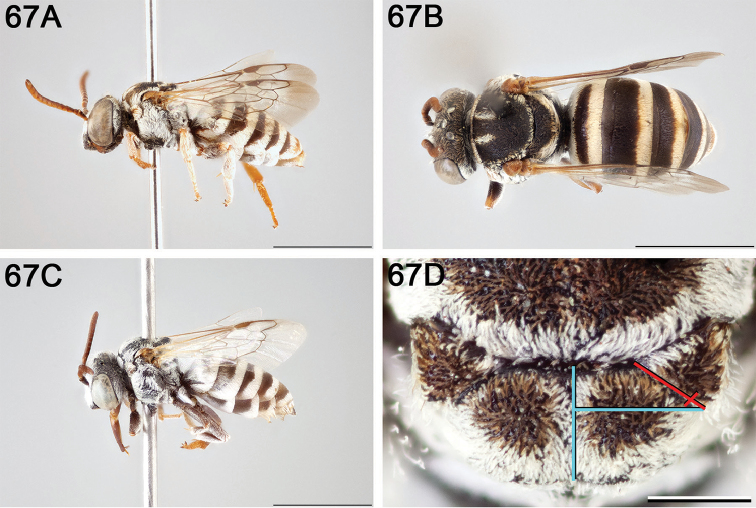
*Epeolus mesillae* **A** female, lateral habitus (scale bar 3 mm) **B** female, dorsal habitus (scale bar 3 mm) **C** male (photo of *P. mesillae* neotype), lateral habitus (scale bar 3 mm), and **D** female axillae and mesoscutellum, dorsal view (scale bar 0.5 mm; blue lines indicate the posterior extent of the axilla relative to the length of the mesoscutellum; red lines indicate the extent of the free portion of the axilla relative to its entire medial length).

#### Distribution.

**Figure 68.**
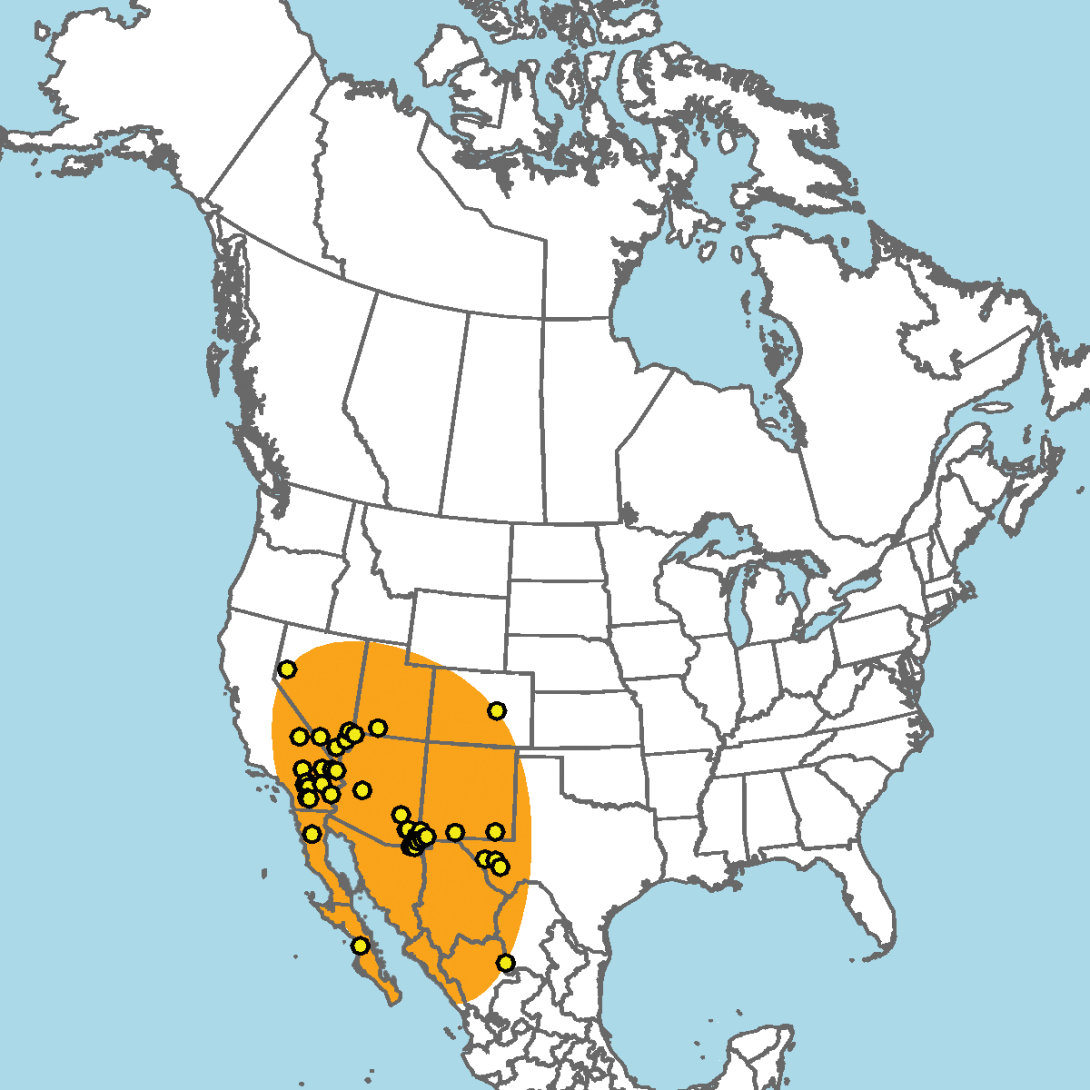
Approximate geographic range of *E. mesillae* (orange) based on occurrence records known to the author (yellow circles).

Known to occur in all major North American deserts (Fig. 68).

#### Ecology.

HOST RECORDS: *Colletes clypeonitens* Swenk is the presumed host of *E. mesillae* (Hurd and Linsley 1975). Personal observations support such an association. In Whitewater, California, USA, I have collected large numbers of female *E. mesillae* and male *C. clypeonitens* in an area dominated by creosote bush (*Larrea tridentata* (Sessé & Moc. ex DC.) Coville (Zygophyllaceae)) in late March 2016. Only one specimen (a female) of a different species of *Colletes* (*C. larreae* Timberlake) was taken at the same locality.

FLORAL RECORDS: Collection records from data contributors to Discover Life (Ascher and Pickering 2017) compiled by J. Pickering indicate the following floral associations: *Cryptantha flavoculata* (A. Nelson) Payson, *Erigeron canus* A. Gray, *Heterotheca villosa*, *Larrea tridentata*, and *Potentilla hippiana* Lehm. (Rosaceae). Labels of examined voucher specimens further indicate associations with *Baileya pleniradiata* Harv. & A. Gray ex A. Gray (Compositae), *Chaenactis stevioides* Hook. & Arn. (Compositae), *Dimorphocarpa wislizeni* (Engelm.) Rollins (Brassicaceae), *L. glutinosa* Engelm., *Melilotus* Mill., *Psoralea lanceolata* Pursh (Leguminosae), *Prosopis velutina* Wooton (Leguminosae), and *Tamarix gallica* L. (Tamaricaceae).

#### Discussion.

*Epeolus mesillae* was originally described under the now defunct genus *Phileremus* because the fore wing in this species has two rather than three submarginal cells, the typical state for most *Epeolus* species. Among North American *Epeolus*, *E. mesillae* exhibits unusual sexual dimorphism in that in females the fore wing and (to a lesser extent) hind wing are apically dusky whereas in males the wings are hyaline throughout. There is some variability in the pubescence on the metasomal terga among specimens, with some exhibiting more grayish-white than yellowish fasciae. Linsley (1939) recognized specimens from southern California as a distinct subspecies (*E. mesillae palmarum*) based on a larger body size and the presence of pale tomentum interspersed with darker tomentum on the discs of the metasomal terga, especially laterally. Specimens from across the range of this species exhibiting these features have been examined, as well as specimens from southern California in which the metasomal fasciae are clearly distinct from the all-dark discs. Specimens from near the type locality of *E. mesillae palmarum* were barcoded, and their sequences cluster closely with those from specimens from Southeast Arizona and adjacent Sonora, nearer the type locality (Las Cruces, New Mexico) of *E. mesillae mesillae*. Hence, I do not consider these to be distinct subspecies, and herein synonymize *E. mesillae palmarum* under *E. mesillae*, a change in taxonomic status first proposed by Brumley (1965).

I have not seen the male holotype of *P. mesillae* and do not know where it is housed, despite personally searching through the entomological collections where T.D. Cockerell deposited the types of other *Epeolus* species he described. In Brumley (1965), no reference was made to Cockerell’s holotype of *P. mesillae*, suggesting Brumley too was unable to find it. Moreover, no references in the literature to Cockerell’s type since the species’ original description could be found. In the same publication, another species was described under *Phileremus* – *P. verbesinae* (now *Neolarra verbesinae* (Cockerell)) –, which was redescribed by Michener (1939) who indicated that the type was in the T.D.A. Cockerell Collection. It is unclear if either specimen has since ended up in an institution that maintains a research collection, but that the holotype of *E. mesillae* has not been referenced since its original description strongly suggests it is unlikely to turn up in the future and to all intents and purposes has been lost. In my search for the holotype at the CUM, a male specimen of *E. mesillae* (labelled as *Phileremus mesillae* Ckll.) from Mesilla Park (the original type locality) collected by Cockerell from *Dimorphocarpa wislizeni* on May 7^th^ was discovered. The specimen, which is the property of the CUM, agrees with the original description, and was used to write the present redescription and diagnosis. Given that a synonymy under *E. mesillae* is proposed herein, it is sensible to have a neotype to serve as a point of reference for any future comparisons. Aside from the collection date, the specimen selected as the neotype of *Phileremus mesillae* fits the description of the original, which can no longer be traced. Hence, in this particular case the qualifying conditions for designating a neotype as listed under Article 75.3 of the International Commission on Zoological Nomenclature (ICZN) Code (http://iczn.org/iczn/index.jsp) seem to have been met.

#### Material studied.

**Type material.** Primary: USA: **California**: Edom (Riverside County), 28.iii.1936, E.G. Linsley (*E. mesillae palmarum* holotype ♀ [CAS, catalog number: 04789]); **New Mexico**: Mesilla Park, 07.v.????, T.D. Cockerell (*P. mesillae* neotype ♂, CUM).

Secondary: USA: **California**: 1 mi W Edom (Riverside County), 28.iii.1936, E.G. Linsley (*E. mesillae palmarum* allotype ♂ [CAS, catalog number: 04790]).

#### DNA barcoded material with BIN-compliant sequences.

Available. BOLD:AAF0161. Specimens examined and sequenced.–Mexico: **Sonora**: 30 km E Agua Prieta (31.3333°N; 109.2403°W), 25.iv.2006, R.L. Minckley (3♀, 1♂, PCYU), 03.v.2005, R.L. Minckley (2♂, PCYU).

USA: **Arizona**: Douglas R/C Flying Field (31.3430°N; 109.4980°W) (Cochise County), 28.iv.2016, T.M. Onuferko (1♀, PCYU); **California**: 31 km N Lucerne Valley (34.6840°N; 116.9605°W) (San Bernardino County), 27.iv.2013, Z.M. Portman (1♂, BBSL); Kelso Dunes (34.8940°N; 115.7020°W) (Baker, San Bernardino County), 30.iv.2013, A. Ruttan (1♂, PCYU); Tipton Road (33.9079°N; 116.6510°W) (~1.4 mi SW Whitewater, Riverside County), 26.iii.2016, T.M. Onuferko (1♀, PCYU).

#### Non-barcoded material examined.

Mexico: **Baja California**: Near La Zapopita Valle de Trinidad, 09–14.iv.1961, F.S. Truxal (2♂, LACM); **Baja California Sur**: 19 mi SW S. Miguel Comondu, 23.vi.1967, E.L. Sleeper and E.M. Fisher (1♂, LACM); **Sonora**: 30 km E Agua Prieta (31.3333°N; 109.2403°W), 03.v.2005, R.L. Minckley (1♀, 5♂, PCYU).

USA: **Arizona**: 11 mi NW Wickenberg, 18.iv.1993, J.G. Rozen (2♀, AMNH); 2 Km W Pima (32.9833°N; 110.2833°W) (Graham County), 25.iv.1996, R.L. Minckley (2♂, PCYU); 2 mi S Willcox (Cochise County), 07.v.1956, E. Ordway (1♀, AMNH); 2.5 mi S Willcox (Cochise County), 24.v.1956, E. Ordway (1♂, AMNH), 07.vi.1956, E. Ordway (1♂, AMNH); 4 mi E Willcox (Cochise County), 08.v.1986, J.G. Rozen (3♀, AMNH), 15.v.1986, J.G. Rozen (1♀, AMNH), 16.v.1986, J.G. Rozen (1♀, AMNH), 17.v.1986, J.G. Rozen (2♀, AMNH); 5 mi NE Douglas (Cochise County), 13.v.1987, J.G. Rozen (1♀, AMNH); Douglas R/C Flying Field (31.3430°N; 109.4980°W) (Cochise County), 23.iv.2016, T.M. Onuferko (2♀, PCYU), 28.iv.2016, T.M. Onuferko (1♀, PCYU); Beaver Dam (36.9028°N; 113.9145°W) (1.7 mi ENE Beaver Dam Wash, Mohave County), 10.v.2014, M.C. Orr (1♀, 1♂, BBSL); Skeleton Canyon Road (Cochise County), 12.v.1977, J.G. Rozen (1♂, AMNH); Southwestern Research Station (5 mi W Portal), 23.iv.1956, E. Ordway (1♀, AMNH); Willcox (Cochise County), 16.v.1985, J.G. Rozen (1♂, AMNH); **California**: 1 mi W Searchlight Junction (San Bernardino County), 21.iii.1971, R.F. Denno and R.W. Rust (1♂, UCBME); 18 mi W Blythe (Riverside County), 22.iv.1978, R.M. Bohart (1♂, UCBME); 25 mi E Twentynine Palms (34.0806°N; 115.5667°W) (Riverside County), 16.iv.2005, L. Packer (1♂, PCYU); 31 km N Lucerne Valley (34.6840°N; 116.9605°W) (San Bernardino County), 27.iv.2013, Z.M. Portman (1♂, BBSL); Borrego Springs (San Diego County), 31.iii.1973, C. Goodpasture (3♂, UCBME); Borrego Valley (San Diego County), 02.iv.1973, R.M. Bohart (1♂, UCBME); Darwin Falls (Inyo County), 12.v.1974, R.M. Bohart (1♀, UCBME); Goffs (San Bernardino County), 24.iv.1993, J.G. and B.L. Rozen (3♀, AMNH), 06.v.1993, J.G. and B.L. Rozen (1♀, AMNH); Morongo Valley (San Bernardino County), 27.iv.1962, O.C. La France (2♂, AMNH); Thousand Palms (Riverside County), 02.iv.1966, R.O. Schuster (1♂, UCBME); Tipton Road (33.9079°N; 116.6510°W) (~1.4 mi SW Whitewater, Riverside County), 26.iii.2016, T.M. Onuferko (6♀, PCYU); **Colorado**: Foster Ranch (El Paso County), 21.vi.1978, F.M. Brown (1♂, CUM); **Nevada**: 1 mi N Crystal (Nye County), 25.v.1999, L. Packer (1♀, PCYU); 2.8 mi E Wadsworth (Washoe County), 30.vi.1963, G.I. Stage (1♀, AMNH); E Las Vegas (36.0983°N; 115.0025°W) (Clark County), 29.iv.2001, A.L. Hicks and V. Scott (1♀, CUM); Overton (Clark County), 09.v.1958, R.C. Bechtel (1♀, AMNH); Sams Camp Wash (Lincoln County), 10.v.-11.vi.1984, R.C. Bechtel and J.B. Knight (1♀, BBSL); **New Mexico**: 10 mi S Animas (Hidalgo County), 15.v.2013, J.G. Rozen (1♂, AMNH); 15 mi E Animas (Hidalgo County), 15.v.2013, J.G. Rozen (3♂, AMNH); Carlsbad (Eddy County), 20.v.1969, Brothers, Krueger, and Michener (1♂, KUNHM); Road Forks (Hidalgo County), 16.v.2013, J.G. Rozen (2♂, AMNH); **Texas**: 20 km S Kent (Jeff Davis County), 30.iv.2003, L. Packer and G. Fraser (1♀, PCYU); 7.6 mi S Van Horn (Culberson County), 27.iv.1979, R.R. Snelling (1♂, LACM); Chihuahuan Desert Research Institute (Jeff Davis County), 29.iv.2003, L. Packer and G. Fraser (2♀, PCYU); **Utah**: Dry Fork (Kane County), 22.v.2000, O. Messinger (1♀, BBSL).

### 33. Epeolus minimus

Taxon classificationAnimaliaHymenopteraApidae

(Robertson, 1902)

Figs 69 , 70 , 101

Triepeolus minimus Robertson, 1902. Entomol. News 13: 81 (♀).
Argyroselenis minima Robertson, 1903. Can. Entomol. 35: 284.
Epeolus beulahensis Cockerell, 1904. Ann. Mag. Nat. Hist. 13: 40 (♀).
Epeolus lutzi Cockerell, 1921. Am. Mus. Novit. 23: 16 (♂).
Epeolus lutzi dimissus Cockerell, 1921. Am. Mus. Novit. 23: 16 (♀).
Epeolus arciferus Cockerell (in Cockerell and Sandhouse, 1924). Proc. Calif. Acad. Sci. (4) 13: 319 (♀).
Epeolus pilatei Cockerell (in Cockerell and Sandhouse, 1924). Proc. Calif. Acad. Sci. (4) 13: 320 (♀).
Epeolus eastwoodae Cockerell, 1937. Pan-Pac. Entomol. 13: 149 (♂).

#### Diagnosis.

The following morphological features in combination (excluding any that are specific to the opposite sex of the one being diagnosed) can be used to tell *E. minimus* apart from all other North American *Epeolus* except *E. banksi* and *E. olympiellus*: in females, F2 is at least 1.2 × as long as wide; the mesoscutum has distinct, evenly broad paramedian bands that may be joined posteriorly; the axilla is small to intermediate in size, not extending much beyond the midlength of the mesoscutellum (extending to <2/3 its length) but the free portion is more than 1/4 as long as the entire medial length of the axilla, and the axilla (except sometimes the tip) and mesoscutellum are black; the mesopleuron is closely (most i<1d) and evenly punctate; T1 has a quadrangular discal patch, in dorsal view the longitudinal band is at least half as wide as the breadth of the apical fascia; and the T2 fascia has lobe-like anterolateral extensions of tomentum. Whereas in *E. banksi* the mesoscutum and metasomal terga have bands of gray short appressed setae, in *E. minimus* the mesoscutum and metasomal terga have bands of off-white to pale yellow short appressed setae. In this respect, *E. minimus* more closely resembles *E. olympiellus*, but in *E. olympiellus* the T3 and T4 fasciae are broken or at least narrowed laterally, as well as medially, whereas in *E. minimus* the T3 and T4 fasciae are not broken laterally, and are complete or narrowly interrupted medially. *Epeolus minimus* is also similar to *E. axillaris*, but in *E. axillaris* the metanotum has a distinct posteromedial depression (as opposed to being flat) and the axilla is more elongate, extending well beyond the midlength of the mesoscutellum but not as far back as its posterior margin.

**Figure 69.**
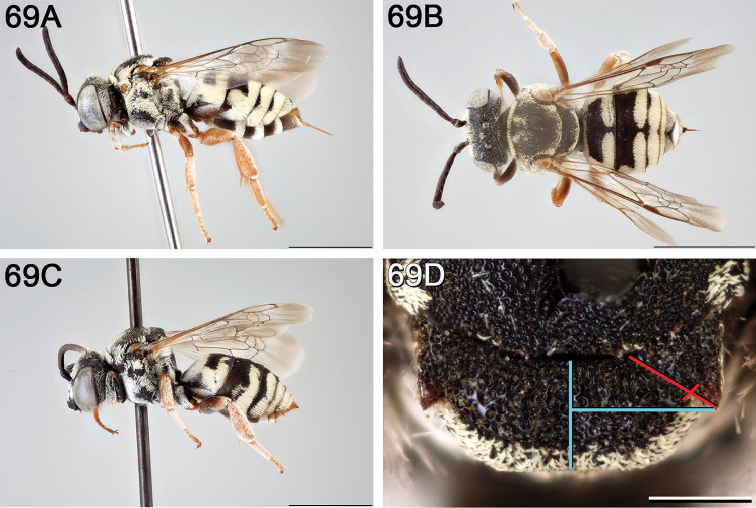
*Epeolus minimus* **A** female, lateral habitus (scale bar 3 mm) **B** female, dorsal habitus (scale bar 3 mm) **C** male, lateral habitus (scale bar 3 mm), and **D** female axillae and mesoscutellum, dorsal view (scale bar 0.5 mm; blue lines indicate the posterior extent of the axilla relative to the length of the mesoscutellum; red lines indicate the extent of the free portion of the axilla relative to its entire medial length).

#### Redescription.

This species was recently redescribed (Onuferko 2017).

#### Distribution.

**Figure 70.**
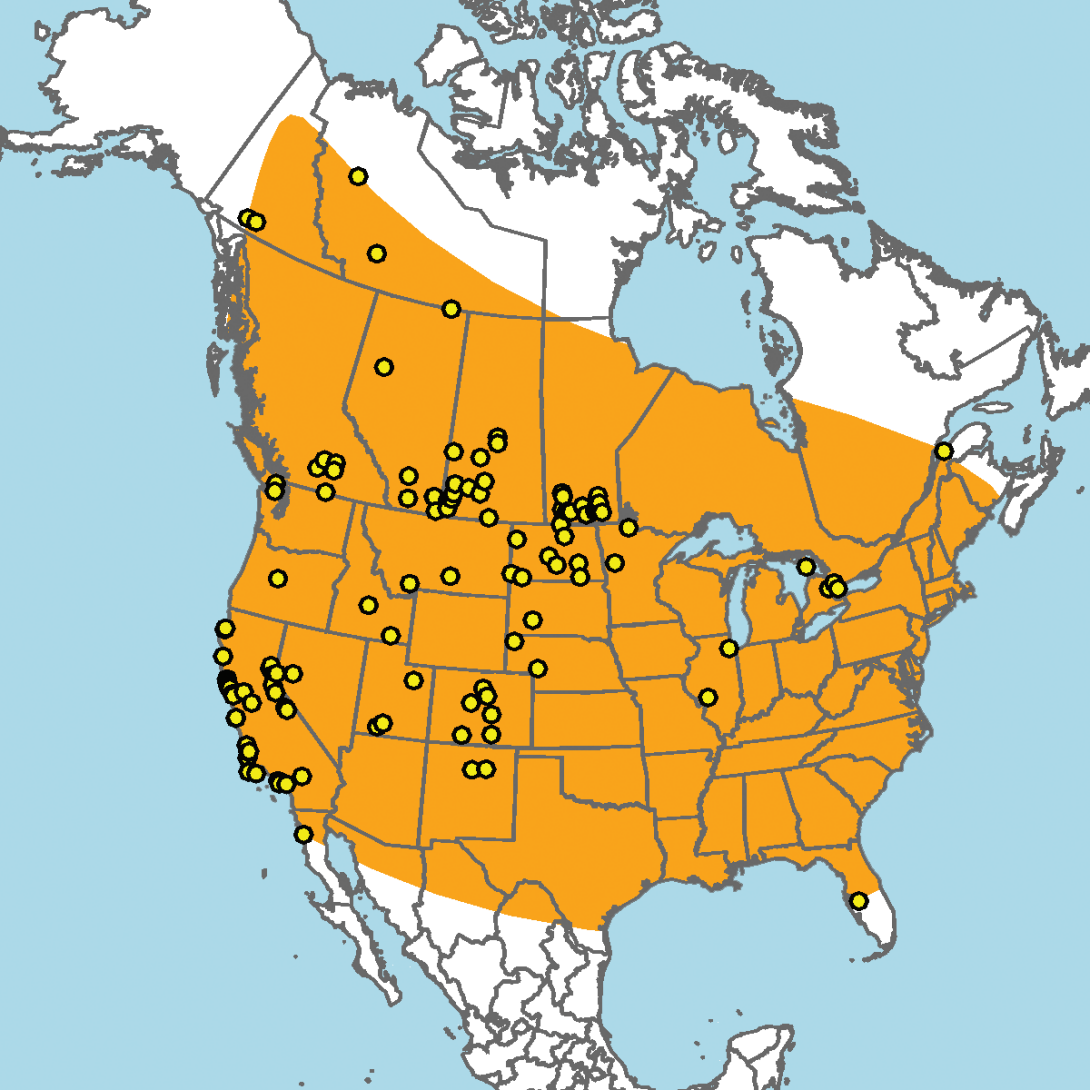
Approximate geographic range of *E. minimus* (orange) based on occurrence records known to the author (yellow circles).

Widely distributed across Canada and the United States, although apparently more common in the west; not known to occur in parts of northeastern North America or the high arctic (Fig. 70). Also, the single (perhaps mislabelled) examined specimen from Florida is an extreme outlier, and given the lack of other examined material from the Southern United States the record should be treated with some skepticism.

#### Ecology.

HOST RECORDS: Graenicher (1906) associated *E. minimus* (as *A. minima*) with *C. eulophi* Robertson based on detailed observations of a female of the former inspecting and entering the nest of a female of the latter in Lake Woods, Wisconsin, USA. However, according to Stephen (1954) Graenicher’s record of *C. eulophi* in Wisconsin is based on observations of *C. kincaidii*. *Epeolus minimus* has been collected with *C. kincaidii* in Birds Hill Provincial Park and Spruce Woods Provincial Park, Manitoba, Canada where no *C. eulophi* were collected or observed (J. Gibbs, personal communication, 2017), so the association between *E. minimus* and *C. kincaidii* seems likely.

FLORAL RECORDS: See Onuferko (2017). Floral associations are also indicated in Suppl. material 1, which includes newly discovered associations with Ericameria nauseosa var. nauseosa and *Medicago* L. (Leguminosae) based on labels of examined voucher specimens.

#### Discussion.

In Onuferko (2017), *E. minimus* is said to be similar to a Californian species yet to be formally recognized, which herein is formally described under the name *Epeolus axillaris*. Detailed morphological and taxonomic remarks about this species are given in Onuferko (2017). *Epeolus minimus* is among the most widespread and commonly collected *Epeolus* species in North America.

#### Material studied.

**Type material.** Primary: USA: **California**: Cuyler’s Cove (San Miguel Island), 27.vii.1937, T.D. Cockerell (*E. eastwoodae* holotype ♂ [CAS, catalog number: 04651]); Pacific Grove (Monterey County), ix.1920, F.E. Blaisdell (*E. arciferus* holotype ♀ [CAS, catalog number: 01614]); San Pedro, 25.x.1909, G.R. Pilate (*E. pilatei* holotype ♀ [CAS, catalog number: 01615]); **Colorado**: Leadville, 03–05.viii.1919 (*E. lutzi dimissus* holotype ♀ [AMNH, catalog number: 25099]); Walsenburg, 14.vi.1919 (*E. lutzi* holotype ♂ [AMNH, catalog number: 25098]); **Illinois**: Carlinville (Macoupin County), C.A. Robertson (*T. minimus* holotype ♀ [INHS, catalog number: 62276]); **New Mexico**: Beulah, 11.vii.????, T.D. Cockerell (*E. beulahensis* holotype ♀ [USNM, catalog number: 534040]).

#### DNA barcoded material with BIN-compliant sequences.

Available. BOLD:AAD3554. Specimens examined and sequenced.–Canada: **Alberta**: 2♂ (PCYU); **British Columbia**: Haynes’ Lease Ecological Reserve (49.0930°N; 119.5200°W), 29.vi.-01.vii.2011, G.A. Gielens (1♀, RSKM); **Manitoba**: Spruce Woods Provincial Park (49.6630°N; 99.2790°W) (Spirit Sands, Division 7), 07.vii.2017, J. Gibbs and Nozoe (1♀, 1♂, JBWM); **Ontario**: 1♀, 1♂ (PCYU); **Saskatchewan**: 3♂ (PCYU); Saskatchewan Landing Provincial Park (50.6950°N; 107.9030°W), 03.vii.2013, A. Fortney and M. Anderson (1♂, RSKM); **Yukon**: 1♂ (RSKM).

USA: **California**: 1♂ (EMEC); **Colorado**: 2♀ (PCYU); Morrison (39.6677°N; 105.1968°W) (SE Red Rocks Amphitheatre), 16.vi.2017, T.M. Onuferko (1♂, PCYU); **Idaho**: 2♀, 1♂ (BBSL, PCYU); **Utah**: 1♀ (BBSL).

#### Non-barcoded material examined.

Mexico: **Baja California**: San Vicente, 08.vii.1963, J. Powell (3♂, EMEC).

Canada: **Alberta**: 15♀, 10♂ (CNC); **British Columbia**: 16♀, 1♂ (CNC, ROM); **Manitoba**: 4♀, 7♂ (CNC, DEBU, ROM); Birds Hill Provincial Park (50.0100°N; 96.9100°W) (Division 12), 15.vii.2017, J. Gibbs and Nozoe (1♂, JBWM); Erickson, 03.viii.1983, D.H. Pengelly (1♀, JBWM); Fort Whyte (Winnipeg), 20.vi.1991, B.G. Elliot (1♂, JBWM), 13.vii.1991, B.G. Elliot (1♂, JBWM); Portage la Prairie, 29.vi.1976, T.D. Galloway (1♀, JBWM); Spruce Woods Provincial Park (49.6630°N; 99.2790°W) (Spirit Sands, Division 7), 07.vii.2017, J. Gibbs and Nozoe (1♂, JBWM); Winnipeg, 21.vi.1979, T.D. Galloway (1♂, JBWM), 06.vii.1991, B.G. Elliot (1♂, JBWM); Winnipeg Beach, 15.vii.1989, T.D. Galloway (1♀, JBWM), 15.vii.1989, T.D. Galloway (2♂, JBWM); **Northwest Territories**: 7♀ (CNC); **Ontario**: 9♀, 6♂ (CNC, PCYU, ROM); Caledon (Forks of the Credit Provincial Park), 25.vii.1968, P. MacKay (1♂, PCYU); **Quebec**: 3♂ (CNC); **Saskatchewan**: 9♀, 10♂ (CNC, PCYU); Borden Bridge, 01.viii.1976, T.D. Galloway (1♀, JBWM); Ernfold, 05.vii.1984, T.D. Galloway (1♀, JBWM); Sands Hills (7 km W Piapot), 26.vii.2003, D. Larson (1♂, PCYU); **Yukon**: 2♀ (CNC).

USA: **California**: 4♀, 4♂ (EMEC, UCR); 2 mi S Asilomar (Monterey County), 26.ix.1959, C.W. O’Brien (1♂, AMNH); Antioch (Contra Costa County), 20.ix.1958, J.R. Powers (1♀, AMNH), 28.viii.1976, N.J. Smith (1♀, UCBME); Bodega Head (Sonoma County), 14.v.1977, W.M. Oldham (1♂, UCBME); Carnelian Bay (Lake Tahoe), 29.vii.1962, R.M. Bohart (1♀, UCBME); Carson Pass (Alpine County), 13.vii.1966, R.M. Bohart (1♂, UCBME), 16.vii.1968, R.M. Bohart (1♂, UCBME), 16.vii.1968, W.W. Harberts (1♂, UCBME); Chipmunk Flat (Tuolumne County), 09.viii.1960, C.A. Toschi (1♀, AMNH); Dune Lakes (3 mi S Oceano, San Luis Obispo County), 01.vi.1972, J. Powell (1♂, EMEC), 03–04.x.1972, J. Powell (18♂, EMEC), 07.vi.1973, J. Powell (1♂, EMEC), 11.vii.1973, R. Coville (1♂, EMEC), 12.vii.1973, J. Powell (2♂, EMEC), 02.v.1974, J. Powell (1♂, EMEC); Holcomb Valley (San Bernardino County) (1♂, BBSL); Hot Creek (8 air mi E Mammoth Lakes, Mono County), 24.viii.1977, J. Powell (2♂, EMEC); Inglenook Fen (5 mi N Fort Bragg, Mendocino County), 27.v.1976, R. Coville (1♀, 3♂, EMEC); Inglenook Fen (Mendocino County), 22.vii.1972, E.I. Schlinger (1♂, EMEC); Lanphere-Christensen Dunes Preserve (4 mi W Arcata, Humboldt County), 26.vii.1975, M.E. Buegler and E.I. Schlinger (1♂, EMEC); Lobos Creek (San Francisco County), 10.v.1979, J. Powell (1♀, 3♂, EMEC), 15.vi.1960, G.I. Stage (1♀, AMNH); Mad River Beach (Humboldt County), 26.vi.1969, J. Powell (1♀, EMEC); McClures Beach (Marin County), 27.vi.1969, R.W. Thorp (1♀, UCBME); North Beach (Point Reyes National Seashore, Marin County), 10.v.1980, K. Standow (1♀, EMEC), 30.viii.1974, P.A. Opler (2♀, 1♂, EMEC); North Fork, Del Puerto Creek (Del Puerto Canyon, Stanislaus County), 25.v.1974, E. Schlinger (1♂, EMEC); Point Reyes National Seashore (Marin County), 03.iii.1968, R.W. Thorp (1♀, UCBME), 23.vii.1974, P.A. Opler (1♀, EMEC); San Bruno Mountain (San Mateo County), 23.v.1961, G.I. Stage (1♀, AMNH), 23.v.1961 (1♀, AMNH), 23.viii.1960, G.I. Stage (1♂, AMNH); San Francisco Bay Salt Marshes, viii.1907?, Thompson (3♂, EMEC); San Francisco Sand Dunes, 25.vi.1954, J.G. Rozen (1♂, EMEC), 25.vi.1954, P.D. Hurd (1♀, EMEC); Santa Cruz Island (Christi Beach, Santa Barbara County), 23.ix.1968, R.W. Thorp (1♀, UCBME); Sierra Valley (Sierra County), 06.vii.1972, R.M. Bohart (1♀, UCBME); Simonton Cove (San Miguel Island, Santa Barbara County), 11.vii.1970, A.A. Grigarick and R.C. Schuster (1♀, UCBME); Toms Place (Mono County), 01.ix.1965, A.J. Slater (1♀, EMEC); Yuba Pass (Sierra County), 11.viii.1978, R.M. Bohart (1♂, KUNHM); **Colorado**: Rock Creek Park (Colorado Springs), 19.viii.1937 (1♀, 1♂, AMNH); **Florida**: 1♀ (PCYU); **Idaho**: 1♀ (PCYU); Daniels Reservoir (Oneida County), 11.vii.1997, F.D. Parker (3♂, BBSL); **Illinois**: 1♂ (FMNH); **Minnesota**: Detroit, 26.viii.1924, O.A. Stevens (1♀, AMNH); **Montana**: 1♀ (KUNHM); 11 mi SE Ennis (Madison County), 18.viii.1966, D.R. Miller (1♀, UCBME); **Nebraska**: Cedar Point Biological Station (8 mi N Ogallala, Keith County), 11–18.vii.1988, J.G. Rozen and E. Quinter (1♀, AMNH); Fort Robinson (Dawes County), 11.viii.1971, J.G., B.L., and K.C. Rozen (3♀, AMNH), 12.viii.1971, J.G., B.L., and K.C. Rozen (2♀, AMNH), 09–11.viii.1972, J.G. Rozen, K.C. Rozen, and R. McGinley (2♀, 4♂, AMNH); Warbonnet Canyon (Sioux County), 24.vii.1968, R.R. Snelling (1♂, LACM); **Nevada**: Fallon, 01.vi.1930, E.L. Bell (1♀, AMNH), 06.vi.1930, E.L. Bell (1♀, AMNH), 10.vi.1930, E.L. Bell (1♀, AMNH); Mount Rose Summit (Washoe County), 09.vii.1964, R.M. Bohart (1♀, UCBME); **New Mexico**: Santa Fe, 09.vi.1931, F.E. Lutz (1♀, 1♂, AMNH); **North Dakota**: Gascoyne, 19.vi.1918, O.A. Stevens (1♀, AMNH); Jamestown, 16.viii.1913, O.A. Stevens (1♀, AMNH); Marmarth, 04.vii.1949, O.A. Stevens (3♂, AMNH); McKenzie, 05.viii.1913, O.A. Stevens (1♀, AMNH); Monango, 03.vii.1913, O.A. Stevens (1♀, AMNH); Pleasant Lake, 11.viii.1913, O.A. Stevens (1♀, AMNH); Washburn, 23.vii.1926, O.A. Stevens (3♀, 2♂, AMNH); Williston, 09.viii.1915, O.A. Stevens (1♂, AMNH); **Oregon**: 1♂ (KUNHM); **South Dakota**: 1♀ (BIML); **Utah**: Indian Canyon (Duchesne County), 18.vii.1965, G.F. Knowlton (1♀, UCBME); NE Ruby’s Inn (Garfield County), 17.viii.1995, V.J. Tepedino and F.D. Parker (2♀, BBSL).

### 34. Epeolus nebulosus sp. n.

Taxon classificationAnimaliaHymenopteraApidae

http://zoobank.org/6C689E06-558F-4C7B-97CE-4BD60D1614E7

Figs 71 , 72 , 99A

#### Diagnosis.

The following morphological features in combination (excluding any that are specific to the opposite sex of the one being diagnosed) can be used to tell *E. nebulosus* apart from all other North American *Epeolus* except *E. basili*, *E. novomexicanus*, and *E. pusillus*: the axilla is large, with the tip extending well beyond the midlength of the mesoscutellum but at most to the band of pale tomentum along its posterior margin, dilated laterally, and ferruginous to some degree whereas the mesoscutellum is typically all black; the axilla’s free portion is clearly less than 2/5 as long as its entire medial length; the mesopleuron is closely (most i<1d) and evenly punctate, that of the female is obscured by white tomentum only in the upper half (with a large, sparsely hairy circle occupying much of the ventrolateral half) whereas that of the male (excluding the hypoepimeral area) is entirely obscured by white tomentum; T2–T4 have complete and evenly broad fasciae; the T2 fascia has lobe-like anterolateral extensions of tomentum; and the pseudopygidial area of the female is lunate and wider than long (the apex ≤2 × the medial length). *Epeolus basili*, *E. nebulosus*, *E. novomexicanus*, and *E. pusillus* are all extremely similar to one another. *Epeolus nebulosus* is most similar to *E. novomexicanus*, but in *E. novomexicanus* the mesoscutum usually has distinct paramedian bands and at least the integument beneath the T1 apical fascia is ferruginous, as are sometimes the rest of the tergum and other terga, whereas in *E. nebulosus* the mesoscutum is entirely obscured by pale tomentum and the metasomal terga (excluding the brown translucent apical margins) are entirely black. In *E. basili* the metasomal terga are also ferruginous to some degree, and the T2 and T3 (for female) or T2–T4 (for male) fasciae are narrowed medially and removed from the apical margin (in *E. nebulosus* the T2–T4 fasciae are on or very little removed from the apical margin), and the pseudopygidial area of the female is ≥2 × the medial length. Whereas in *E. pusillus* the flagellum, except sometimes F1, and metasomal sterna are consistently brown or black and clearly not the same reddish-orange color as the legs (tibiae to tarsi), in *E. nebulosus* the flagellum, at least ventrally, is the same reddish-orange color as the legs (tibiae to tarsi) as are usually the metasomal sterna. *Epeolus nebulosus* is also similar to *E. scutellaris* in that the axilla is large, with the lateral margin arcuate, and that the apical fasciae are complete. However, in *E. scutellaris* the pseudopygidial area of the female is much wider (the apex ~2.5–3 × the medial length) than in *E. nebulosus*, and the mesopleuron of both the female and male is obscured by white tomentum only in the upper half (with a large, sparsely hairy circle occupying much of the ventrolateral half).

#### Description.

MALE: Length 7.2 mm; head length 2.0 mm; head width 2.7 mm; fore wing length 5.5 mm.

*Integument coloration.* Mostly black; notable exceptions as follows: at least partially ferruginous on mandible, antenna, pronotal lobe, tegula, axilla, legs, pygidial plate, and metasomal sterna. Mandible with apex darker than rest of mandible; preapical tooth slightly lighter than mandibular apex. Antenna brown and orange in part. Pronotal lobe and tegula pale ferruginous to amber. Wing membrane subhyaline, apically dusky. Legs more extensively reddish orange than brown or black. S1–S6 reddish orange.

*Pubescence.* Face with tomentum densest on clypeus and around antennal socket, slightly sparser on upper paraocular area and vertexal area. Dorsum of mesosoma and metasoma with bands of off-white to pale yellow short appressed setae. Mesoscutum largely obscured by pale tomentum. Mesopleuron (excluding hypoepimeral area) entirely obscured by white tomentum. Metanotum with tomentum uninterrupted, uniformly off white. T1 with narrow and short discal patch largely obscured by pale tomentum. T2–T6 each with complete fascia, T2 with fascia with wide basomedially convergent anterolateral extensions of tomentum. S4 and S5 with long coppery to silvery subapical hairs, which individually are often darker apically.

*Surface sculpture.* Punctures dense. Labrum with larger and sparser punctures (i=1–2d) than clypeus (i<1d) (difficult to see in holotype because clypeus entirely obscured by tomentum; described from paratypes with hair removed). Small impunctate shiny spot lateral to lateral ocellus. Mesoscutum, mesoscutellum, and axilla coarsely and densely rugose-punctate. Tegula densely punctate mesally (i≤1d), less so laterally (i=1–2d). Mesopleuron with ventrolateral half densely punctate (i<1d) to rugose; mesopleuron with punctures more or less equally dense throughout (not visible in holotype because mesopleuron entirely obscured by tomentum; described from paratypes). Metasomal terga with punctures very fine, dense (i≈1d), evenly distributed on disc.

*Structure.* Preapical tooth obtuse. Labrum with pair of small subapical denticles not preceded by carinae (difficult to see in holotype; described from paratypes). Frontal keel not strongly raised. Scape with greatest length 2.0 × greatest width. F2 noticeably longer than wide (L/W ratio = 1.2). Preoccipital ridge not joining hypostomal carina, from which it is separated by about 1.5 MOD at its terminal. Mesoscutellum weakly bigibbous. Axilla large, its lateral margin (L) half as long as mesoscutellar width (W) (L/W ratio = 0.5) and tip extending well beyond midlength of mesoscutellum but not as far back as its posterior margin; axilla with tip clearly visible, but unattached to mesoscutellum for less than 2/5 the medial length of axilla; axilla with lateral margin arcuate. Fore wing with three submarginal cells. Pygidial plate apically rounded, with large deep, well-separated punctures, with the interspaces shining.

FEMALE: Description as for male except for usual secondary sexual characters and as follows: F2 even longer than wide (L/W ratio = 1.5); mesopleuron densely hairy, except for two almost entirely bare patches (one beneath base of fore wing (hypoepimeral area), a larger circular patch occupying much of ventrolateral half of mesopleuron); T5 with large, continuous patch of pale tomentum bordering and contacting pseudopygidial area present only in female; T5 with pseudopygidial area lunate, its apex twice as wide as medial length, indicated by silvery setae on disc of apicomedial region elevated from rest of tergum; S4 and S5 with much shorter hairs (S5 with apical fimbria of coppery to silvery hairs not extending beyond apex of sternum by much more than 1/4 MOD); pygidial plate apically truncate, with small, denser punctures.

**Figure 71.**
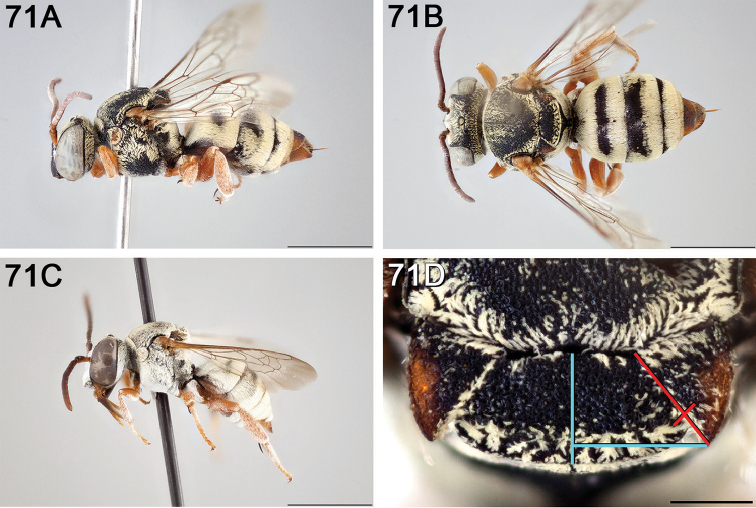
*Epeolus nebulosus* **A** female allotype, lateral habitus (scale bar 3 mm) **B** female allotype, dorsal habitus (scale bar 3 mm) **C** male holotype, lateral habitus (scale bar 3 mm), and **D** female allotype axillae and mesoscutellum, dorsal view (scale bar 0.5 mm; blue lines indicate the posterior extent of the axilla relative to the length of the mesoscutellum; red lines indicate the extent of the free portion of the axilla relative to its entire medial length).

#### Etymology.

The name is in reference to the pale tomentum obscuring much of the integument of this species. From the Latin, “nebulosus” (hazy).

#### Distribution.

**Figure 72.**
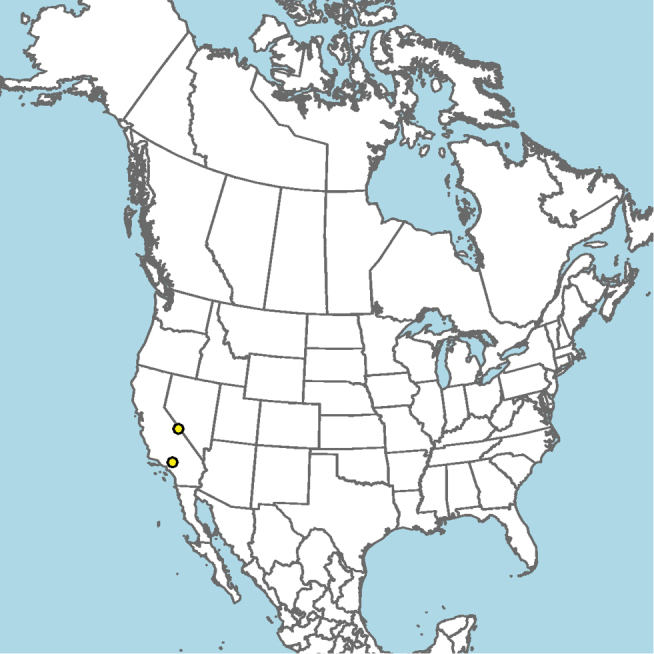
Occurrence records of *E. nebulosus* known to the author (yellow circles).

California and probably western Nevada (Fig. 72).

#### Ecology.

HOST RECORDS: The host species of *E. nebulosus* is/are presently unknown.

FLORAL RECORDS: Labels of examined voucher specimens indicate a floral association with *Ericameria nauseosa*.

#### Discussion.

*Epeolus nebulosus* is a cryptic species within the “*pusillus* group” that closely resembles some specimens of *E. novomexicanus*, and the ranges of the two species overlap to some extent. The morphological differences (in integument coloration and patterns of pubescence) among the four members of the “*pusillus* group” are subtle. The status of *E. nebulosus* as a separate species is further supported by a separate BIN and large barcode sequence divergence (>3.2%) from its nearest neighbor, *E. novomexicanus*. Although most species of *Epeolus* were described from a female name-bearing type, a male specimen is designated as the holotype of *E. nebulosus* because a barcode-compliant sequence is associated with it and because much of the pubescence is discolored or rubbed off in the available female specimen, which is herein designated as the allotype. Since this species is described from very few specimens, efforts should be made to collect additional representatives of *E. nebulosus* for DNA barcoding to determine if the morphological differences between it and *E. novomexicanus* reported here are consistent.

#### Material studied.

**Type material.** Primary: USA: **California**: Gilbert Pass on Hwy 168 (37.4305°N; 117.9388°W) (N Deep Springs Valley, Inyo County), 14.ix.2013, M.C. Orr (holotype ♂ [CCDB-28239 F01], BBSL).

Secondary: USA: **California**: 3.2 km S Pearblossom (Los Angeles County), 07.xi.1977, R.R. Snelling (allotype ♀, LACM); Gilbert Pass on Hwy 168 (37.4305°N; 117.9388°W) (N Deep Springs Valley, Inyo County), 14.ix.2013, M.C. Orr (paratypes 2♂, BBSL).

#### DNA barcoded material with BIN-compliant sequences.

Available. BOLD:ACZ0767. See Type material for specimens examined and sequenced (indicated by unique CCDB-plate and well number).

### 35. Epeolus novomexicanus

Taxon classificationAnimaliaHymenopteraApidae

Cockerell, 1912

**Figure 73.**
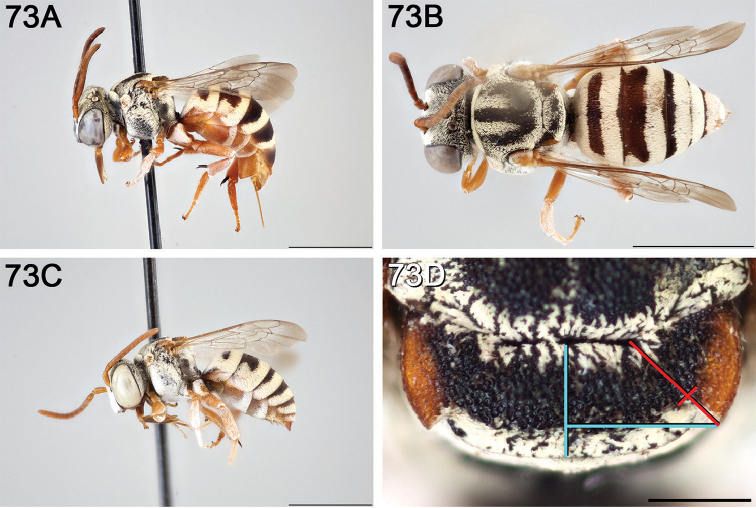
*Epeolus novomexicanus* **A** female, lateral habitus (scale bar 3 mm) **B** female, dorsal habitus (scale bar 3 mm) **C** male, lateral habitus (scale bar 3 mm), and **D** female axillae and mesoscutellum, dorsal view (scale bar 0.5 mm; blue lines indicate the posterior extent of the axilla relative to the length of the mesoscutellum; red lines indicate the extent of the free portion of the axilla relative to its entire medial length).

**Figure 74.**
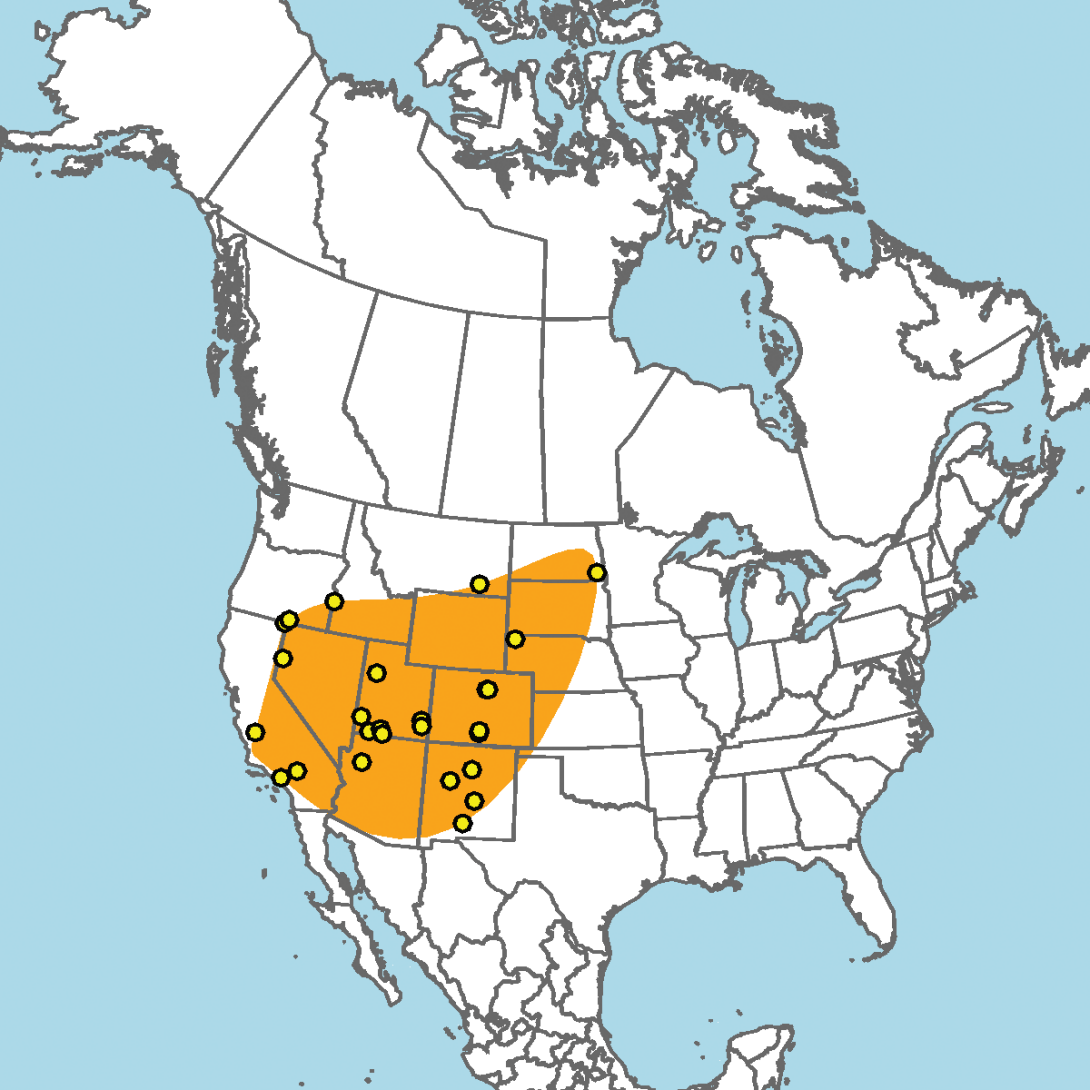
Approximate geographic range of *E. novomexicanus* (orange) based on occurrence records known to the author (yellow circles).

Figs 73 , 74 , 97E , 99B

Epeolus novomexicanus Cockerell, 1912. Ann. Mag. Nat. Hist. (8) 10: 487 (♂).

#### Diagnosis.

The following morphological features in combination (excluding any that are specific to the opposite sex of the one being diagnosed) can be used to tell *E. novomexicanus* apart from all other North American *Epeolus* except *E. basili*, *E. nebulosus*, and *E. pusillus*: the axilla is large, with the tip extending well beyond the midlength of the mesoscutellum but at most to the band of pale tomentum along its posterior margin, dilated laterally, and ferruginous to some degree whereas the mesoscutellum is typically all black; the axilla’s free portion is clearly less than 2/5 as long as its entire medial length; the mesopleuron is closely (most i<1d) and evenly punctate, that of the female is obscured by white tomentum only in the upper half (with a large, sparsely hairy circle occupying much of the ventrolateral half) whereas that of the male (excluding the hypoepimeral area) is entirely obscured by white tomentum; T2–T4 have complete and evenly broad fasciae; the T2 fascia has lobe-like anterolateral extensions of tomentum; and the pseudopygidial area of the female is lunate and wider than long (the apex ≤2 × the medial length). *Epeolus basili*, *E. nebulosus*, *E. novomexicanus*, and *E. pusillus* are all extremely similar to one another. *Epeolus novomexicanus* is most similar to *E. nebulosus*, but in *E. nebulosus* the mesoscutum is entirely obscured by pale tomentum and the metasomal terga (excluding the brown translucent apical margins) are entirely black whereas in *E. novomexicanus* the mesoscutum usually has distinct paramedian bands and at least the integument beneath the T1 apical fascia is ferruginous, as are sometimes the rest of the tergum and other terga. In *E. basili* the metasomal terga are also ferruginous to some degree, but the T2 and T3 (for female) or T2–T4 (for male) fasciae are narrowed medially and removed from the apical margin (in *E. novomexicanus* the T2–T4 fasciae are on or very little removed from the apical margin), and the pseudopygidial area of the female is ≥2 × the medial length. Whereas in *E. pusillus* the flagellum, except sometimes F1, and metasomal sterna are consistently brown or black and clearly not the same reddish-orange color as the legs (tibiae to tarsi), in *E. novomexicanus* the flagellum, at least ventrally, is the same reddish-orange color as the legs (tibiae to tarsi) as are usually the metasomal sterna. *Epeolus novomexicanus* is also similar to *E. scutellaris* in that the axilla is large, with the lateral margin arcuate, and that the apical fasciae are complete. However, in *E. scutellaris* the pseudopygidial area of the female is much wider (the apex ~2.5–3 × the medial length) than in *E. novomexicanus*, and the mesopleuron of both the female and male is obscured by white tomentum only in the upper half (with a large, sparsely hairy circle occupying much of the ventrolateral half).

#### Redescription.

MALE: Length 6.1 mm; head length 1.7 mm; head width 2.3 mm; fore wing length 4.4 mm.

*Integument coloration.* Mostly black; notable exceptions as follows: at least partially ferruginous on mandible, labrum, antenna, pronotal lobe, tegula, axilla, legs, metasomal terga (including pygidial plate), and metasomal sterna. Mandible with apex darker than rest of mandible; preapical tooth slightly lighter than mandibular apex (difficult to see in holotype because mandible closed; described from non-type specimens). Antenna brown and orange in part. Pronotal lobe and tegula pale ferruginous to amber. Wing membrane subhyaline, apically dusky. Legs more extensively reddish orange than brown or black. S1–S6 reddish orange.

*Pubescence.* Face with tomentum partly rubbed off in holotype, but white and densest around antennal socket in non-type specimens. Tomentum slightly sparser on clypeus; upper paraocular and frontal areas, and vertexal area mostly exposed. Dorsum of mesosoma and metasoma with bands of off-white to pale yellow short appressed setae. Mesoscutum with paramedian band partly obscured by surrounding pale tomentum. Mesopleuron (excluding hypoepimeral area) entirely obscured by white tomentum (except where rubbed off in holotype). Metanotum with tomentum uninterrupted, uniformly off white. T1 with narrow and short discal patch partly obscured by pale tomentum. T2–T5 each with complete fascia (T6 mostly retracted in holotype, but with complete fascia in non-type specimens), T2 with fascia with wide basomedially convergent anterolateral extensions of tomentum. S4 and S5 with long coppery to silvery subapical hairs, which individually are often darker apically.

*Surface sculpture.* Punctures dense. Labrum with larger and sparser punctures (i=1–2d) than clypeus (i<1d). Small impunctate shiny spot lateral to lateral ocellus. Mesoscutum, mesoscutellum, and axilla coarsely and densely rugose-punctate. Tegula densely punctate (i≤2d). Mesopleuron with ventrolateral half densely punctate (i<1d) to rugose; mesopleuron with punctures more or less equally dense throughout. Metasomal terga with punctures very fine, dense (i≈1d), evenly distributed on disc.

*Structure.* Preapical tooth obtuse. Labrum with pair of small subapical denticles not preceded by carinae. Frontal keel not strongly raised. Scape with greatest length 1.8 × greatest width. F2 as long as wide (L/W ratio = 1.0). Preoccipital ridge not joining hypostomal carina, from which it is separated by no less than 1 MOD at its terminal. Mesoscutellum weakly bigibbous. Axilla large, its lateral margin (L) half as long as mesoscutellar width (W) (L/W ratio = 0.5) and tip extending well beyond midlength of mesoscutellum but not as far back as its posterior margin; axilla with tip clearly visible, but unattached to mesoscutellum for less than 2/5 the medial length of axilla; axilla with lateral margin arcuate. Fore wing with three submarginal cells. Pygidial plate apically rounded, with large deep punctures closely clustered.

FEMALE: Description as for male except for usual secondary sexual characters and as follows: F2 noticeably longer than wide (L/W ratio = 1.5); mesopleuron densely hairy, except for two sparsely hairy circular patches (one behind pronotal lobe, a larger one occupying much of ventrolateral half of mesopleuron); T5 with large, continuous patch of pale tomentum bordering and contacting pseudopygidial area present only in female; T5 with pseudopygidial area lunate, its apex less than twice as wide as medial length, indicated by silvery setae on impressed disc of apicomedial region elevated from rest of tergum; S4 and S5 with much shorter hairs (S5 with apical fimbria of coppery to silvery hairs extending beyond apex of sternum by ~1/3 MOD); pygidial plate apically truncate, with small, denser punctures.

#### Distribution.

Western North America (Fig. 74).

#### Ecology.

HOST RECORDS: Torchio (1965) reported an association between *E. pusillus* (identified as such by R. Brumley) and *C. ciliatoides* Stephen (identified as such by W. Stephen, who in 1954 described the species) based on observations of females of the former entering the nests of females of the latter from an aggregation near Delta, Utah, USA. Brumley (1965) noted that a series of *E. pusillus* specimens taken from the Great Basin (primarily Utah) differed from other members of that species in having a reddish orange labrum, clypeus, antenna, mesopleuron, and metasomal terga and/or sterna; broader metasomal fasciae; and often denser pubescence on the mesoscutum. Herein, specimens matching that description are recognized as a separate albeit closely-related species, *E. novomexicanus*, which Brumley (1965) considered to be synonymous with *E. crucis*, a name herein synonymized under *E. compactus*.

FLORAL RECORDS: Labels of examined voucher specimens indicate floral associations with *Chrysothamnus* (possibly in reference to plants that now are in the genus *Ericameria*), *Erigeron* L., *Haplopappus* Cass. (Compositae), *Helianthus*, *Lupinus* L. (Leguminosae), *Machaeranthera* Nees (Compositae), and *Senecio spartioides* Torr. & A. Gray.

#### Discussion.

Brumley (1965) considered *E. novomexicanus* and *E. rufulus* to be synonyms of *E. crucis*, a name which herein is recognized as a synonym of *E. compactus*. Here, *E. novomexicanus* and *E. rufulus* are considered to be valid names associated with two very different species, with the former most closely resembling *E. basili*, *E. nebulosus*, and *E. pusillus*. Although sequenced specimens of *E. novomexicanus* and *E. pusillus* share the same BIN, and were previously all regarded as *E. pusillus* (Onuferko 2017), the difference in coloration and pubescence between the two forms is as pronounced as, if not more than, that between the true *E. pusillus* and sequenced representatives of the two members of the “*pusillus* group” (*E. basili* and *E. nebulosus*) that were assigned separate BINs. Hence, with strong molecular support for partitioning this species group into three distinct clusters in which four distinct forms can be recognized morphologically, I have opted to treat *E. novomexicanus* and *E. pusillus* as heterospecific. The holotypes (both males) of *E. nebulosus* and *E. novomexicanus* are similarly covered in dense tomentum and closely resemble one another, and it should be noted that sequenced specimens resembling the holotypes of both species but from nearer the type locality of *E. novomexicanus* were assigned a BIN that is not shared with *E. nebulosus* but is instead shared with *E. pusillus*.

#### Material studied.

**Type material.** Primary: USA: **New Mexico**: Santa Fe, 02.viii.1912, T.D. Cockerell (holotype ♂ [USNM, catalog number: 534049]).

#### DNA barcoded material with BIN-compliant sequences.

Available. BOLD:AAX7180. Specimens examined and sequenced.–USA: **Utah**: 4.17 mi SE Wig Mountain (40.2876°N; 113.0390°W) (Toole County), 26.ix.2005, T.L. Griswold (1♀, BBSL); Beef Basin Rd (38.0846°N; 109.5765°W) (N Cottonwood Creek, San Juan County), 03.x.2014, M.C. Orr (1♀, BBSL).

#### Non-barcoded material examined.

USA: **Arizona**: Near Hyde Park (Coconino County), 28.ix.1964, Timberlake (1♂, USNM); **California**: 8 mi W Coalinga (Fresno County), 28.ix.1957, R.R. Snelling (1♂, LACM); Los Angeles County, ix.????, Coquillett (1♀, USNM); Sugar Loaf Mountain (Modoc County), 12.ix.1969, E.E. Grissell and R.F. Denno (1♀, 1♂, UCBME); Victorville, 28.ix.1938, Timberlake (1♂, USNM); **Colorado**: Boulder (Boulder County), 28.viii.1976, U.N. Lanham (1♂, CUM); Great Sand Dunes National Monument (Alamosa County), 22.ix.1979, F.M. Brown (1♀, CUM); Great Sand Dunes National Monument (37.6629°N; 105.6212°W) (Alamosa County), 24.viii.2000, A.L. Hicks and V. Scott (1♀, 5♂, CUM); White Rocks (Boulder County), 24.vii.1934, C.H. Hicks (1♀, CUM); **Idaho**: Homedale, 16.viii.1974, R.M. Bohart (1♀, 1♂, UCBME); **Montana**: Ashland (Rosebud County), 11.viii.1970, D.R. Miller (1♀, USNM); **Nebraska**: Smiley Canyon (42.7964°N; 103.4045°W) (Fort Robinson State Park, Sioux County), 05.ix.1999, A.L. Hicks and V. Scott (1♀, CUM); **Nevada**: The Needle Rocks (N end Pyramid Lake, Washoe County), 15.ix.1983, J. Doyen (1♂, EMEC); **New Mexico**: Laguna, 07.viii.1966, D.R. Miller (1♀, 1♂, UCBME); Near Tecolote, 05.ix.??30 (1♀, USNM); White Sands National Monument (near Alamogordo), 01.ix.1940, H.G. Rodeck (1♂, CUM); **North Dakota**: 1 mi SE McLeod (Ransom County), 26.viii.1972, J.R. Powers (1♀, EMEC); **Oregon**: Deep Creek (1 mi E Adel, Lake County), 13.ix.1969, R.F. Denno and E.E. Grissell (2♂, UCBME); **Utah**: 0.5 mi S Springdell (Uinta National Forest), 22.viii.1963, C.W. O’Brien (1♂, AMNH); 1 mi N Kitchen Corral Spr 12S (Kane County), 10.ix.2002, L. Topham (1♀, BBSL); 13.2 mi N Blanding (San Juan County), 24.viii.??67, J.C. Hall (1♂, USNM); 16 mi W Tropic (37.3913°N; 112.2575°W) (Garfield County), 28.vii.2008, T.L. Griswold (1♀, BBSL); Beryl (Iron County), 27.ix.1953, M. Cazier (1♀, AMNH).

### 36. Epeolus olympiellus

Taxon classificationAnimaliaHymenopteraApidae

Cockerell, 1904

Figs 75 , 76

Epeolus olympiellus Cockerell, 1904. Ann. Mag. Nat. Hist. 13: 41 (♂).
Epeolus tristicolor Viereck, 1905. Can. Entomol. 37: 280 (♀).
Epeolus humillimus Cockerell, 1918. Ann. Mag. Nat. Hist. (9) 1: 160 (♂).
Epeolus rufomaculatus Cockerell and Sandhouse, 1924. Proc. Calif. Acad. Sci. (4) 13: 314 (♀).
Epeolus rubrostictus Cockerell and Sandhouse, 1924. Proc. Calif. Acad. Sci. (4) 13: 318 (♀).

#### Diagnosis.

The following morphological features in combination (excluding any that are specific to the opposite sex of the one being diagnosed) can be used to tell *E. olympiellus* apart from all other North American *Epeolus* except *E. banksi* and *E. minimus*: in females, F2 is at least 1.2 × as long as wide; the mesoscutum has distinct, evenly broad paramedian bands that may be joined posteriorly; the axilla is small to intermediate in size, not extending much beyond the midlength of the mesoscutellum (extending to <2/3 its length) but the free portion is more than 1/4 as long as the entire medial length of the axilla, and the axilla (except sometimes the tip) and mesoscutellum are black; the mesopleuron is closely (most i<1d) and evenly punctate; T1 has a quadrangular discal patch, in dorsal view the longitudinal band is at least half as wide as the breadth of the apical fascia; and the T2 fascia has lobe-like anterolateral extensions of tomentum. Whereas in *E. banksi* the mesoscutum and metasomal terga have bands of gray short appressed setae, in *E. olympiellus* the mesoscutum and metasomal terga have bands of off-white to pale yellow short appressed setae. In this respect, *E. olympiellus* more closely resembles *E. minimus*, but in *E. minimus* the T3 and T4 fasciae are not broken laterally, and are complete or narrowly interrupted medially, whereas in *E. olympiellus* the T3 and T4 fasciae are broken or at least narrowed laterally, as well as medially. Whereas throughout most of its range *E. minimus* exhibits reddish-orange coloration on the labrum, antenna, pronotal lobe, and/or legs, except foreleg, from trochanters to tarsi, in *E. olympiellus* the labrum, antenna, and legs from coxae to femora are brown or black. *Epeolus olympiellus* is also similar to *E. axillaris*, but in *E. axillaris* the metanotum has a distinct posteromedial depression (as opposed to being flat) and the axilla is more elongate, extending well beyond the midlength of the mesoscutellum but not as far back as its posterior margin.

**Figure 75.**
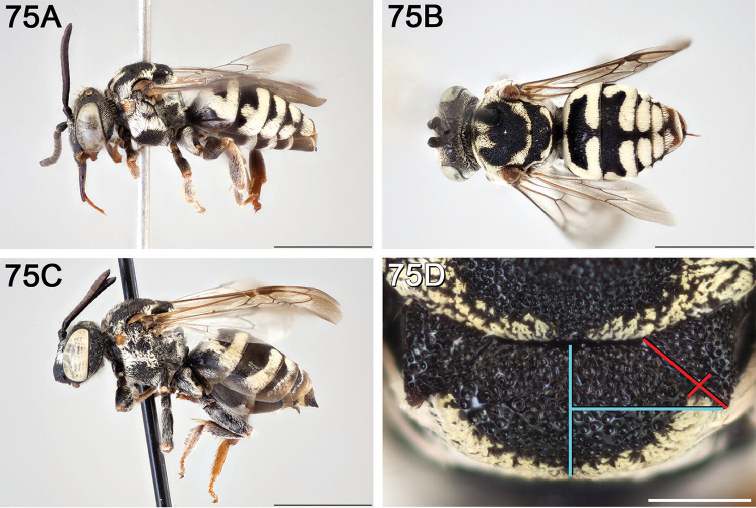
*Epeolus olympiellus* **A** female, lateral habitus (scale bar 3 mm) **B** female, dorsal habitus (scale bar 3 mm) **C** male, lateral habitus (scale bar 3 mm), and **D** female axillae and mesoscutellum, dorsal view (scale bar 0.5 mm; blue lines indicate the posterior extent of the axilla relative to the length of the mesoscutellum; red lines indicate the extent of the free portion of the axilla relative to its entire medial length).

#### Description.

This species was recently redescribed (Onuferko 2017).

#### Distribution.

**Figure 76.**
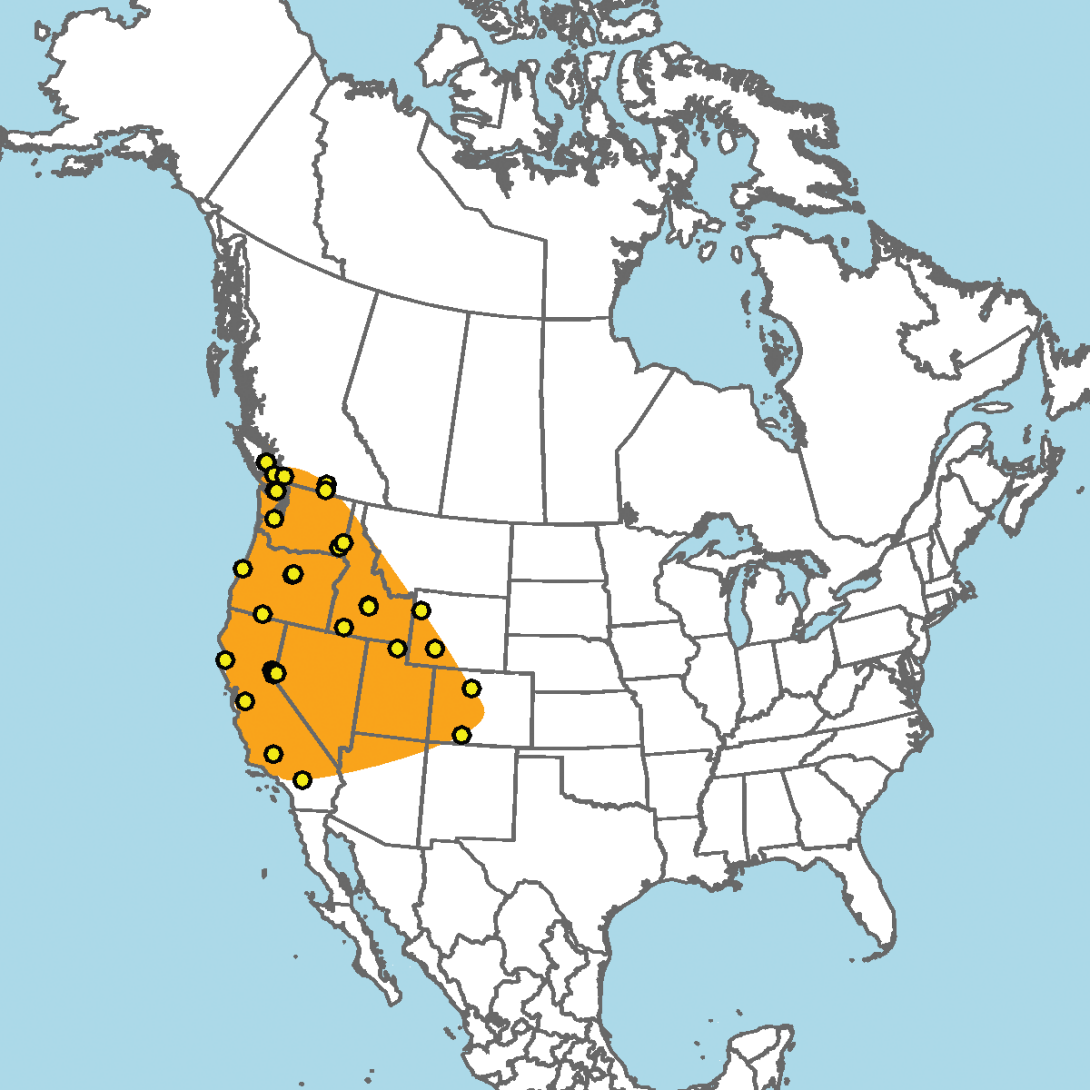
Approximate geographic range of *E. olympiellus* (orange) based on occurrence records known to the author (yellow circles).

United States west of the Rocky Mountains to southern British Columbia (Fig. 76).

#### Ecology.

See Onuferko (2017) for host and floral records. Floral associations are also indicated in Suppl. material 1.

#### Discussion.

Detailed morphological and taxonomic remarks about this species are given in Onuferko (2017).

#### Material studied.

**Type material.** Primary: Canada: **British Columbia**: Nanaimo (Nanaimo Biological Station), 24.vi.1920, E.P. Van Duzee (*E. rubrostictus* holotype ♀ [CAS, catalog number: 01613]); Vancouver (*E. tristicolor* holotype ♀ [ANSP, catalog number: 10123]).

USA: **Utah**: Logan, 14.vii.1922, E.P. Van Duzee (*E. rufomaculatus* holotype ♀ [CAS, catalog number: 01609]); **Washington**: Pullman, 02.viii.1908, W.M. Mann (*E. humillimus* holotype ♂ [USNM, catalog number: 534047]); Olympia, 02.vii.1896, T. Kincaid (*E. olympiellus* holotype ♂ [USNM, catalog number: 534051]).

#### DNA barcoded material with BIN-compliant sequences.

Available. BOLD:AAC6215. Specimens examined and sequenced.–USA: **California**: 2♀, 4♂ (PCYU); **Colorado**: 2♀, 1♂ (PCYU); **Idaho**: 5♀ (PCYU); **Oregon**: 2♀, 1♂ (PCYU); **Washington**: 1♂ (PCYU); **Wyoming**: 2♀ (AMNH, BBSL).

#### Non-barcoded material examined.

Canada: **British Columbia**: 5♀, 5♂ (CNC).

USA: **California**: 4♀, 2♂ (PCYU); 17.2 mi S Livermore (on Mines Road, Alameda County), 22.v.1976, M.L. Siri and R.B. Kimsey (1♂, UCBME); Boca (Nevada County), 21.vi.1962, E.J. Montgomery (1♂, UCBME), 31.vii.1967, R.M. Bohart (1♂, UCBME); Carnelian Bay (Lake Tahoe), 24.vi.1973, R.M. Bohart (1♀, UCBME); Dollar Lake Trail (San Bernardino Mountains), 11.vii.1966, R.M. Bohart (1♂, UCBME); Hwy 99, 1.7 mi S Hwy 223 (Kern County), 16.ix.1999, G.R. Ballmer (1♀, UCR); **Colorado**: 3♀, 4♂ (PCYU); 6 mi ESE Kremmling (Grand County), 20.vii.1982, P. Robinson (1♀, CUM); **Idaho**: 6♀ (PCYU); Grasmere (Owyhee County), 07.vii.1968, A.R. Gittins (1♂, UCBME); Ketchum (43.7630°N; 114.4003°W) (Blaine County), 25.vi.2007, J. Gibbs (1♀, JBWM); **Nevada**: Mount Rose Summit (Washoe County), 09.vii.1964, R.M. Bohart (1♂, UCBME); **Oregon**: 1♀, 1♂ (PCYU); Hwy 26 (44.5500°N; 120.3472°W) (Wheeler County), 28.vi.2007, J. Gibbs (1♀, JBWM).

### 37. Epeolus packeri sp. n.

Taxon classificationAnimaliaHymenopteraApidae

http://zoobank.org/935F1E70-F185-48A2-9DCB-0E14AD9F849A

**Figure 77.**
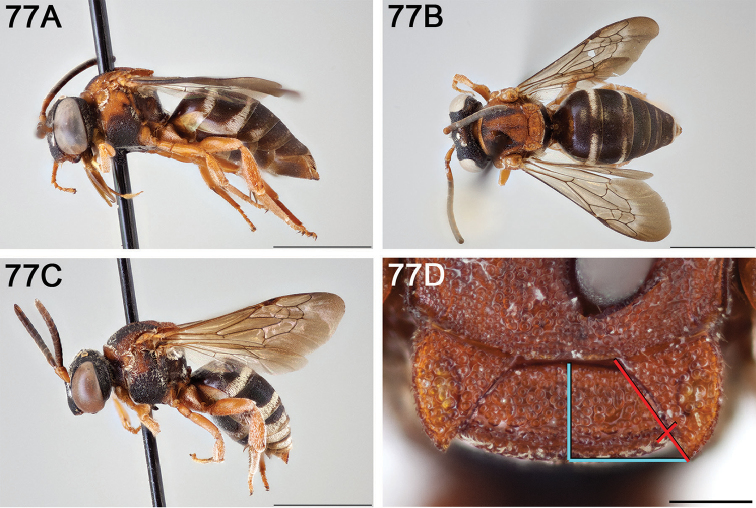
*Epeolus packeri* **A** female holotype, lateral habitus (scale bar 3 mm) **B** female holotype, dorsal habitus (scale bar 3 mm) **C** male paratype, lateral habitus (scale bar 3 mm), and **D** female paratype axillae and mesoscutellum, dorsal view (scale bar 0.5 mm; blue lines indicate the posterior extent of the axilla relative to the length of the mesoscutellum; red lines indicate the extent of the free portion of the axilla relative to its entire medial length).

**Figure 78.**
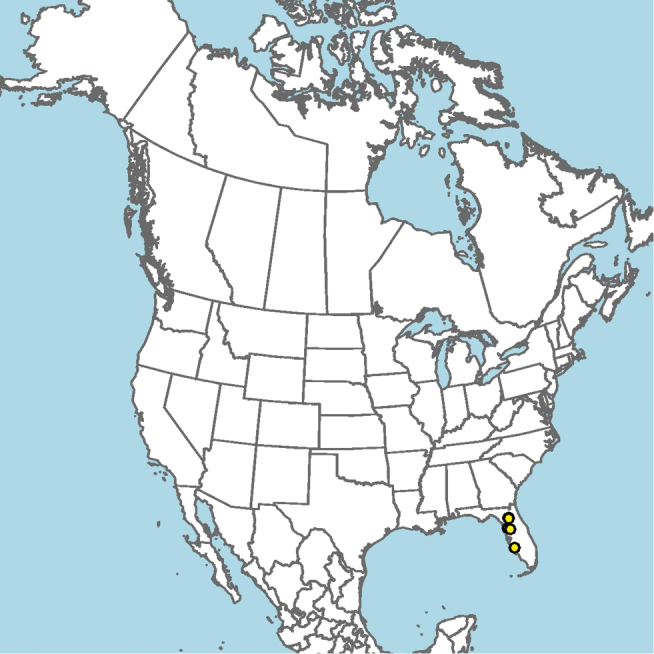
Occurrence records of *E. packeri* known to the author (yellow circles).

Figs 77 , 78 , 92D , 97A

#### Diagnosis.

The following morphological features in combination can be used to tell *E. packeri* apart from all other North American *Epeolus*: the pronotal collar is predominantly ferruginous; the axilla is large, with the tip extending as far back as or beyond the posterior margin of the mesoscutellum, dilated laterally, and like the mesoscutellum ferruginous; the mesopleuron is closely (most i<1d) and evenly punctate; the metasomal terga have pale but not brownish orange pubescence; and the T1–T3 apical fasciae are interrupted medially and commonly reduced to discrete lateral patches. *Epeolus packeri* resembles *E. andriyi*, *E. deyrupi*, *E. floridensis*, and *E. howardi* in that the axilla is large, with the lateral margin arcuate, and like the mesoscutellum ferruginous, and that the T1–T3 apical fasciae are interrupted medially. However, in *E. packeri* the pseudopygidial area of the female is wider (the apex >2 × the medial length) than in *E. andriyi*, *E. floridensis*, or *E. howardi* (the apex <2 × the medial length), and the T1 basal fascia is absent or reduced to a pair of small patches of pale tomentum whereas in *E. andriyi*, *E. floridensis*, and *E. howardi* T1 has a distinct, although often medially-interrupted, basal fascia. *Epeolus packeri* closely resembles *E. deyrupi*, but in *E. deyrupi* the mesopleuron commonly has sparser punctures ventrolaterally (i≤2d) than that of *E. packeri*, with the interspaces shining or somewhat dull due to tessellate surface microsculpture, and the T1–T3 apical fasciae are (to varying degrees) brownish orange medially and off white laterally. *Epeolus packeri* is also similar to *E. scutellaris*, but in *E. scutellaris* the pronotal collar is predominantly black and the T1–T3 apical fasciae are complete or only very narrowly interrupted medially.

#### Description.

FEMALE: Length 8.3 mm; head length 2.0 mm; head width 2.8 mm; fore wing length 6.2 mm.

*Integument coloration*. Black in part, at least partially ferruginous on mandible, labrum, lower paraocular area, antenna, pronotal collar, pronotal lobe, tegula, axilla, mesoscutum, mesoscutellum, metanotum, mesopleuron, metapleuron, propodeum, legs, and metasomal sterna. Mandible with apex darker than rest of mandible; preapical tooth slightly lighter than mandibular apex (difficult to see in holotype; described from paratype). Antenna brown except scape, pedicel, and F1 extensively orange. F2 with orange spot basally. Pronotal lobe and tegula pale ferruginous to amber. Mesoscutum reddish orange except medially on anterior margin and along parapsidal line. Wing membrane dusky subhyaline, slightly darker at apex. Legs more extensively reddish orange than brown or black.

*Pubescence*. Face with tomentum densest on paraocular area around antennal socket, otherwise almost entirely bare. Mesoscutum without pale tomentum. Dorsum of metasoma with bands of off-white short appressed setae. Mesopleuron nearly bare, except along margins. Metanotum with tomentum uninterrupted except for median bare patch in posterior half, uniformly off white. T1 and T2 with apical fasciae medially interrupted, narrowed (broader laterally), and removed from apical margin; T2 with fascia without anterolateral extensions of tomentum. Metasoma otherwise without fasciae, although T3 and T4 with few sparsely scattered pale hairs present on apical impressed areas. T5 with pseudopygidial area lunate, its apex more than twice as wide as medial length, indicated by silvery setae on flat disc of apicomedial region elevated from rest of tergum. S5 with apical fimbria of coppery to silvery hairs extending beyond apex of sternum by 1/3 MOD.

*Surface sculpture*. Punctures dense. Labrum with larger and sparser punctures (i=1–2d) than clypeus (i<1d). Small impunctate matte spot lateral to lateral ocellus. Mesoscutum, mesoscutellum, and axilla coarsely and densely rugose-punctate. Tegula densely punctate posteriorly (i=1–2d), sparsely punctate (i>2d) to impunctate anteriorly and along margins. Mesopleuron with ventrolateral half densely punctate (i≤1d) to rugose; mesopleuron with punctures more or less equally dense throughout. Metasomal terga with punctures very fine, dense (i≈1d), evenly distributed on disc.

*Structure*. Preapical tooth blunt and obtuse. Labral apex with pair of small denticles, each preceded by longitudinal carina. Frontal keel not strongly raised. Scape with greatest length 1.9 × greatest width. F2 noticeably longer than wide (L/W ratio = 1.4). Preoccipital ridge not joining hypostomal carina, from which it is separated by less than 1 MOD at its terminal (difficult to see in holotype; described from paratype). Mesoscutellum moderately bigibbous. Axilla large, its lateral margin (L) more than half as long as mesoscutellar width (W) (L/W ratio = 0.7) and tip extending slightly beyond apex of horizontal dorsal portion of mesoscutellum; axilla with tip clearly visible, but unattached to mesoscutellum for less than 2/5 the medial length of axilla; axilla with lateral margin arcuate. Fore wing with three submarginal cells. Pygidial plate apically truncate.

MALE: Description as for female except for usual secondary sexual characters and as follows: face with more abundant pale tomentum, densest from midlength of clypeus to upper paraocular and frontal areas; F2 shorter, but still longer than wide (L/W ratio = 1.2); S4 and S5 with much longer coppery to silvery subapical hairs; pygidial plate apically rounded, with large deep punctures closely clustered basally and sparser apically, with the interspaces shining.

#### Etymology.

This species is named in honor of my dissertation adviser, Prof. Laurence Packer, who collected the first specimen of this species I have seen.

#### Distribution.

Florida peninsula (Fig. 78).

#### Ecology.

HOST RECORDS: The host species of *E. packeri* is/are presently unknown.

FLORAL RECORDS: Labels of examined voucher specimens indicate a floral association with *Solidago*.

#### Discussion.

In Mitchell’s (1962) keys to female and male *Epeolus*, this species comes out as *E. floridensis* in which T1 is not bright ferruginous but black. However, in *E. floridensis* the dorsum of the mesosoma and metasoma has more abundant pale pubescence, and the pseudopygidial area is conspicuously narrower. Moreover, all examined specimens of *E. floridensis* (adults) were collected in spring whereas all those identified as *E. packeri* were collected in October.

In terms of surface sculpture, structure, and the width of the pseudopygidial area, *E. packeri* is most similar to *E. scutellaris*, and sequenced representatives of both forms share the same BIN. The two are considered to be heterospecific based on the marked abundance of red coloration coupled with a loss of pubescence (the same rationale for treating *E. glabratus* as distinct from *E. lectoides*) in *E. packeri*, features that are common in Florida Hymenoptera and constitute an unexplained regional phenomenon (Deyrup and Eisner 2003).

#### Material studied.

**Type material.** Primary: USA: **Florida**: Homosassa Tract (Citrus County), 19.x.2002, J. Mosley (holotype ♀, FSCA).

Secondary: USA: **Florida**: Butterfly Garden W McGuire Center for Lepidoptera Research (Gainesville, Alachua County), 20.x.2009, C. Whitehill (paratypes 2♂, FSCA); Gainesville (Alachua County), 14.x.2012, S. Lenberger (paratype ♂ [CCDB-30383 D04], FSCA); Gainesville (Paynes Prairie, Alachua County), 13–23.x.1997, L. Masner (allotype ♂, PCYU); Homosassa Tract (Citrus County), 19.x.2002, J. Mosley (paratypes 1♀, 1♂, ABS); W Murdoch, 20.x.1983, L. Packer (paratype ♀, PCYU); Withlacoochee State Forest (Citrus County), 19.x.2002, J. Mosley (paratypes 2♂, ABS).

#### DNA barcoded material with BIN-compliant sequences.

Available. BOLD:AAG5250. See Type material for specimens examined and sequenced (indicated by unique CCDB-plate and well number).

### 38. Epeolus pusillus

Taxon classificationAnimaliaHymenopteraApidae

Cresson, 1864

Figs 79 , 80 , 98A

Epeolus pusillus Cresson, 1864b. Proc. Entomol. Soc. Phil. 2: 398 (♀).

#### Diagnosis.

The following morphological features in combination (excluding any that are specific to the opposite sex of the one being diagnosed) can be used to tell *E. pusillus* apart from all other North American *Epeolus* except *E. basili*, *E. nebulosus*, and *E. novomexicanus*: the axilla is large, with the tip extending well beyond the midlength of the mesoscutellum but at most to the band of pale tomentum along its posterior margin, dilated laterally, and usually ferruginous to some degree (rarely all black) whereas the mesoscutellum is entirely black; the axilla’s free portion is clearly less than 2/5 as long as its entire medial length; the mesopleuron is closely (most i<1d) and evenly punctate, that of the female is obscured by white tomentum only in the upper half (with a large, sparsely hairy circle occupying much of the ventrolateral half) whereas that of the male (excluding the hypoepimeral area) is entirely obscured by white tomentum; the T1–T3 apical fasciae are complete or only very narrowly interrupted medially; the T2 fascia has lobe-like anterolateral extensions of tomentum; and the pseudopygidial area of the female is lunate and wider than long (the apex ≤2 × the medial length). *Epeolus basili*, *E. nebulosus*, *E. novomexicanus*, and *E. pusillus* are all extremely similar to one another. Whereas in *E. basili* the flagellum, at least ventrally, is the same reddish-orange color as the legs (tibiae to tarsi) as are usually the metasomal sterna, in *E. pusillus* the flagellum, except sometimes F1, and metasomal sterna are consistently brown or black and clearly not the same reddish-orange color as the legs (tibiae to tarsi). Whereas in *E. nebulosus* and *E. novomexicanus* the longitudinal extent of the T1 discal patch is less than or equal to the breadth of the apical fascia and the T2–T4 fasciae are on or very little removed from the apical margin and more or less evenly broad, in *E. pusillus* the longitudinal extent of the T1 discal patch is no less (and usually greater) than the breadth of the apical fascia and the T1–T3 apical fasciae are removed from the apical margin and commonly narrowed or narrowly interrupted medially. *Epeolus pusillus* is also similar to *E. scutellaris* in that the axilla is large, with the lateral margin arcuate, and that the apical fasciae are complete or only very narrowly interrupted medially. However, in *E. scutellaris* the pseudopygidial area of the female is much wider (the apex ~2.5–3 × the medial length) than in *E. pusillus*, and the mesopleuron of both the female and male is obscured by white tomentum only in the upper half (with a large, sparsely hairy circle occupying much of the ventrolateral half). Despite the species name ‘*pusillus*’, meaning very small in Latin, the size range overlaps too much with other species to be diagnostic.

**Figure 79.**
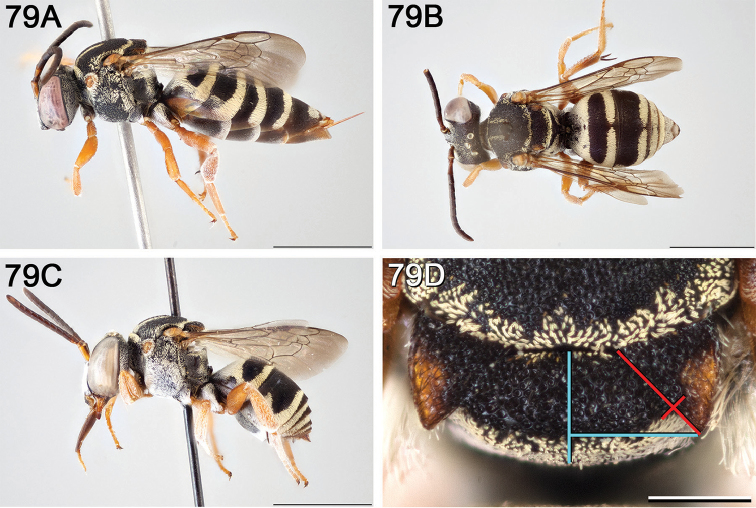
*Epeolus pusillus* **A** female, lateral habitus (scale bar 3 mm) **B** female, dorsal habitus (scale bar 3 mm) **C** male, lateral habitus (scale bar 3 mm), and **D** female axillae and mesoscutellum, dorsal view (scale bar 0.5 mm; blue lines indicate the posterior extent of the axilla relative to the length of the mesoscutellum; red lines indicate the extent of the free portion of the axilla relative to its entire medial length).

#### Description.

This species was recently redescribed (Onuferko 2017).

#### Distribution.

**Figure 80.**
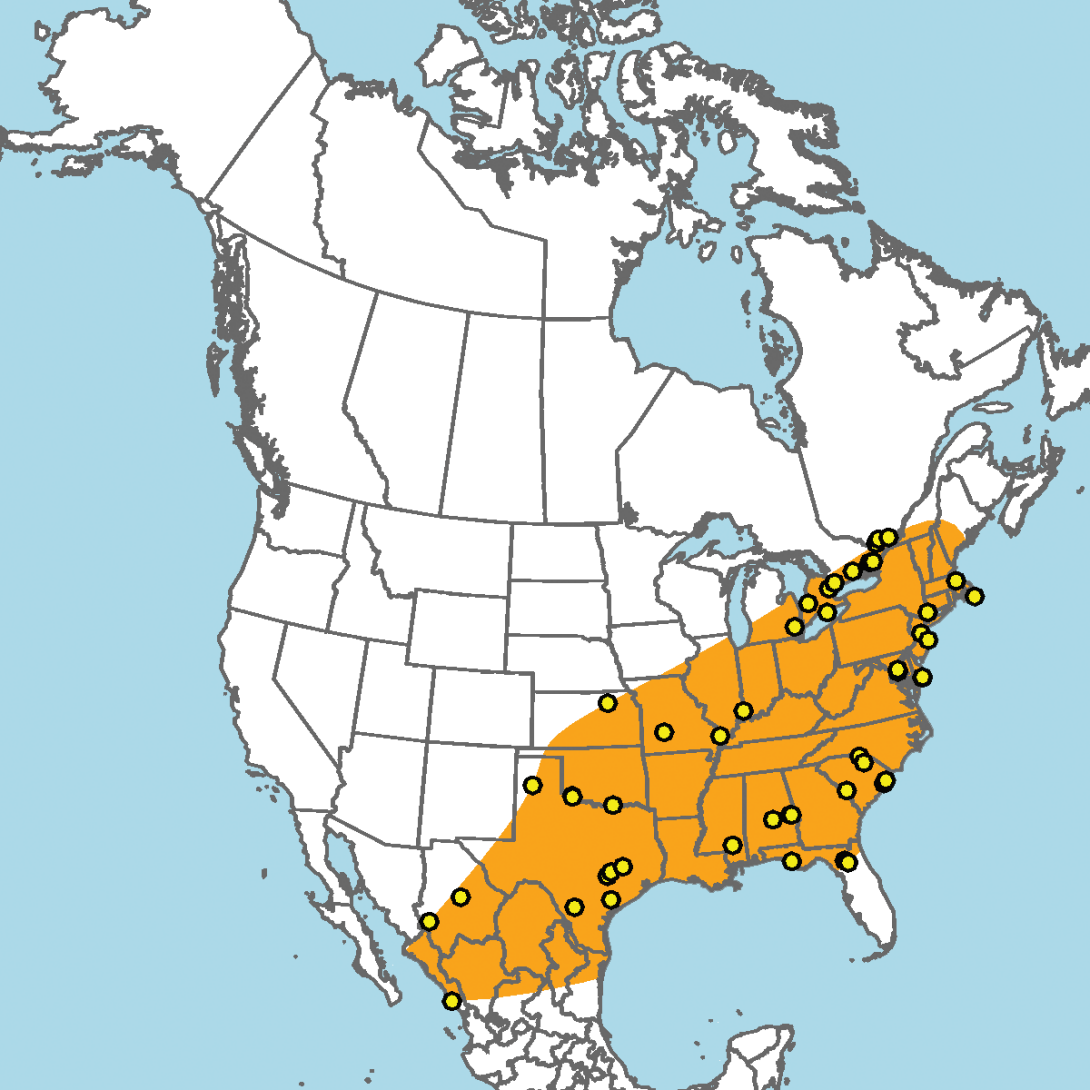
Approximate geographic range of *E. pusillus* (orange) based on occurrence records known to the author (yellow circles).

Eastern North America to Mexico (Fig. 80).

#### Ecology.

HOST RECORDS: Rozen and Favreau (1968) associated *E. pusillus* with *C. compactus compactus* Cresson based on observations of a female of the former entering and emerging from a nest of a female of the latter and subsequent discovery of an *Epeolus* egg upon excavation of the nest. Ascher et al. (2014) noted that the small size and flight season of *E. pusillus* suggest and additional or alternative association with *C. americanus* Cresson.

FLORAL RECORDS: See Onuferko (2017). Floral associations are also indicated in Suppl. material 1, which includes newly discovered associations with *Callirhoe involucrata* (Torr. & A. Gray) A. Gray (Malvaceae), *Heterotheca subaxillaris*, *Rudbeckia fulgida* Aiton, and *R. hirta* based on labels of examined voucher specimens.

#### Discussion.

In Onuferko (2017), barcoded specimens from Utah were regarded as *E. pusillus*, but are now considered to be *E. novomexicanus*, with sequenced representatives of both species sharing the same BIN. Detailed morphological and taxonomic remarks about this species are given in Onuferko (2017).

#### Material studied.

**Type material.** Primary: USA: **Massachusetts**: F.G. Sanborn (holotype ♀ [ANSP, catalog number: 2228]).

#### DNA barcoded material with BIN-compliant sequences.

Available. BOLD:AAX7180. Specimens examined and sequenced.–Canada: **Ontario**: 1♂ (PCYU).

USA: **Alabama**: Autauga County (32.4345°N; 86.5817°W), 19.x.2016, C.H. Ray (1♂, AUMNH); Lee County (32.5553°N; 85.3747°W), 11.x.2016, C.H. Ray (1♂, AUMNH); **Maryland**: 1♂ (BIML); **North Carolina**: 1♂ (BIML); **South Carolina**: Aiken Savannah River Site (33.3594°N; 81.6652°W), 30.ix.2016, S. McCann (1♂, JBWM); S Murrells Inlet, 04.x.2016, T.M. Onuferko (1♀, PCYU).

#### Non-barcoded material examined.

Canada: **Ontario**: 13♀, 23♂ (CNC, DEBU, PCYU, ROM); Caledon (Forks of the Credit Provincial Park), 03.ix.1969, P. MacKay (1♀, PCYU); King, 13.vii.2000, J. Grixti (1♀, PCYU), 23.viii.2002, A. Gravel (1♀, PCYU); Norfolk County (42.6369°N; 80.5472°W), 03.ix.2008, A. Taylor (1♀, PCYU); Norwood, 24.viii.1982, T.D. Galloway (1♀, JBWM); Osprey Marsh (Frontenac County), 03.xi.2001 (1♀, PCYU); Queen’s University Biological Station, 03.ix.2001 (1♀, PCYU); **Quebec**: 1♀ (CNC).

Mexico: **Chihuahua**: 17 mi N Chihuahua, 25.viii.1965, A. Raske (1♀, EMEC); Cuiteco, 14.ix.1969, T.A. Sears, R.C. Gardner, and C.S. Glaser (1♂, UCBME); **Sinaloa**: Mazatlán, 06.viii.1964, W.R.M. Mason (1♀, CNC), 27.iii.1979, L.D. French (1♀, UCBME), 28.iii.1979, L.D. French (1♂, UCBME).

USA: **Alabama**: Auburn (32.5701°N; 85.4603°W) (Lee County), 18.x.2014, C.H. Ray (1♂, AUMNH); Lee County (32.5553°N; 85.3747°W), 11.x.2016, C.H. Ray (1♂, AUMNH); **Florida**: 1♂ (AMNH); Alachua (Alachua County), 05.v.1974, E.E. Grissell (1♀, UCBME), 29.iv.1974, E.E. Grissell (2♀, UCBME); St. Andrews State Park (Panama City), 14.x.2000, C. Porter and L. Stange (1♀, 3♂, FSCA); **Illinois**: 1♀ (FMNH); **Indiana**: 1♂ (USNM); **Kansas**: Riley County (1♂, USNM); **Maryland**: 2♀, 8♂ (BIML); **Massachusetts**: 2♀, 4♂ (BIML); **Mississippi**: 1♂ (AMNH); **New Jersey**: 1♀ (AMNH); Seaside Park, Weiss and West (1♀, CNC); **New York**: 2♀ (AMNH); **Oklahoma**: 1♂ (USNM); Lake Texoma (2 mi E Willis), vii.1965, R.M. Bohart (1♀, UCBME); **South Carolina**: 1♀, 1♂ (BIML, DEBU); **Texas**: 17 mi N Vernon (Wilbarger County), 02.iv.1979, R.J. McGinley (1♂, USNM); Canyon (Randall County), 21.vi.1969, R.M. Bohart (1♀, UCBME); Cotulla, 12.v.1906, J.C. Crawford (1♂, USNM); Dickinson (Galveston County), vi.1929, F.M. Hull (1♀, CNC); Lee County (1♂, USNM); Lick Creek Park (College Station, Brazos County), 22.ix.1990, J. Woolley and J. Huber (1♂, CNC); Stengl “Lost Pines” Biological Research Station (30.0800°N; 97.1830°W), 16.v.2013, J.L. Neff (1♂, CTMI); Victoria, 01.iv.1907, J.D. Mitchell (1♂, USNM).

### 39. Epeolus rufulus

Taxon classificationAnimaliaHymenopteraApidae

Cockerell, 1941

Figs 81 , 82 , 96B

Epeolus rufulus Cockerell, 1941. Can. Entomol. 73: 36 (♀).

#### Diagnosis.

The following morphological features in combination can be used to tell *E. rufulus* apart from all other North American *Epeolus* except *E. attenboroughi*: the mandible has a blunt, obtuse preapical tooth; the preoccipital ridge does not join the hypostomal carina; the mesoscutum is covered in pale tomentum, which is densest anteromedially; the axilla is elongate, extending well beyond the midlength of the mesoscutellum but not as far back as its posterior margin, and the free portion is distinctly hooked; the mesopleuron is closely (most i<1d) and evenly punctate; and T1–T4 have complete apical fasciae. Whereas in *E. attenboroughi* T1 has a comparatively narrow discal patch (the longitudinal band is more than half as wide as the breadth of the apical fascia in dorsal view) and in females F2 is not noticeably longer than wide, in *E. rufulus* the discal patch is so wide that the longitudinal band is barely visible in dorsal view and in females F2 is more than 1.2 × as long as wide. *Epeolus rufulus* is also similar to *E. ainsliei* in that in both species the axilla is dilated laterally and the free portion is distinctly hooked, and the T1–T4 apical fasciae are complete; however, in *E. ainsliei* the mandible is simple, the preoccipital ridge joins the hypostomal carina, and the mesoscutum has distinct paramedian bands.

#### Redescription.

FEMALE: Length 7.6 mm (difficult to gauge in holotype because head detached and glued to collection label, and much of pronotum missing; given instead for non-type specimen most similar in size); head length 1.9 mm; head width 2.6 mm; fore wing length >5.1 mm (margins of both very worn in holotype).

*Integument coloration*. Black in part, at least partially ferruginous on mandible, labrum, clypeus, antenna, pronotal lobe, tegula, axilla, mesoscutum, mesoscutellum, metanotum, mesopleuron, metapleuron, propodeum, legs, metasomal terga (including pygidial plate), and metasomal sterna. Mandible with apex darker than all but extreme base; preapical tooth lighter than mandibular apex (difficult to see in holotype; described from non-type specimen). Antenna brown and orange in part. Pronotal lobe and tegula pale ferruginous to amber. Mesoscutum orange along lateral margin and with pair of orange markings near posterior margin between midline and parapsidal line. Wing membrane subhyaline, apically dusky. Legs entirely reddish orange (both forelegs missing in holotype, but entirely reddish orange in non-type specimens).

*Pubescence*. Face with tomentum densest around antennal socket. Clypeus, upper paraocular and frontal areas, and vertexal area mostly exposed. Dorsum of mesosoma and metasoma with bands of off-white to pale yellow short appressed setae. Mesoscutum sparsely covered in pale tomentum. Mesopleuron with upper half sparsely hairy; ventrolateral half nearly bare, except along margins. Metanotum with tomentum rubbed off medially in holotype, but uninterrupted and uniformly off white in non-type specimens. T1 with discal patch quadrangular and very wide, the basal and apical fasciae only narrowly joined laterally. T1 with basal and apical fasciae and T2–T4 with apical fasciae complete, those of T2 and T3 somewhat broader laterally, T2 with fascia without anterolateral extensions of tomentum. T5 with pseudopygidial area lunate, its apex more than twice as wide as medial length, indicated by silvery setae on impressed disc of apicomedial region elevated from rest of tergum. S5 with apical fimbria of coppery to silvery hairs extending beyond apex of sternum by ~2/5 MOD.

*Surface sculpture*. Punctures dense. Labrum and clypeus with punctures equally dense (i<1d). Impunctate spot lateral to lateral ocellus absent. Mesoscutum, mesoscutellum, and axilla coarsely and densely rugose-punctate. Tegula very densely punctate mesally (i<1d), less so laterally (i=1–2d). Mesopleuron with ventrolateral half densely punctate (i<1d) to rugose; mesopleuron with punctures more or less equally dense throughout. Metasomal terga with punctures very fine, dense (i≈1d), evenly distributed on disc.

*Structure*. Preapical tooth blunt and obtuse. Labrum with pair of small subapical denticles not preceded by carinae. Frontal keel not strongly raised. Scape with greatest length 1.9 × greatest width. F2 noticeably longer than wide (L/W ratio = 1.6). Preoccipital ridge not joining hypostomal carina, from which it is separated by no less than 1 MOD at its terminal (not visible in holotype because head detached and glued to collection label; described from non-type specimens). Mesoscutellum weakly bigibbous. Axilla large, its lateral margin (L) more than half as long as mesoscutellar width (W) (L/W ratio = 0.6) and tip extending well beyond midlength of mesoscutellum but not as far back as its posterior margin; axilla with tip conspicuously diverging from side of mesoscutellum, distinctly hooked, and axilla with free portion 2/5 its medial length; axilla with lateral margin arcuate and carinate. Fore wing with three submarginal cells. Pygidial plate apically truncate.

MALE: Description as for female except for usual secondary sexual characters and as follows: F2 shorter, as long as wide (L/W ratio = 1.1); mesopleuron almost entirely obscured by white tomentum; S4 and S5 with much longer coppery to silvery subapical hairs; pygidial plate apically rounded, with large deep, well-separated punctures, with the interspaces shining.

**Figure 81.**
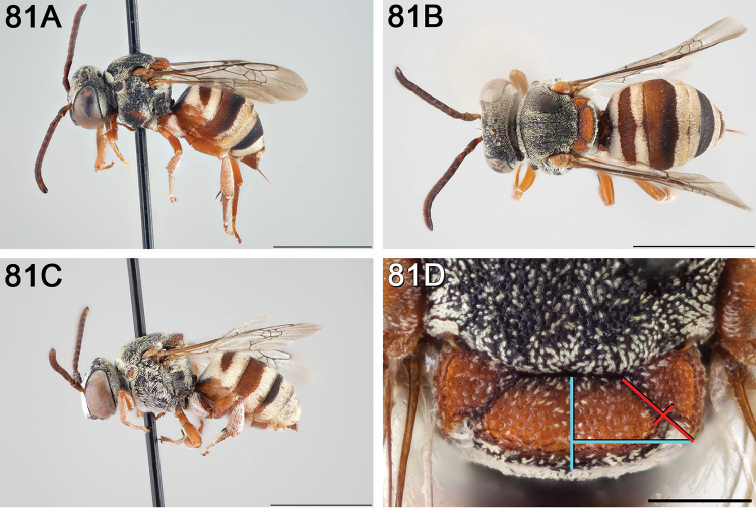
*Epeolus rufulus* **A** female, lateral habitus (scale bar 3 mm) **B** female, dorsal habitus (scale bar 3 mm) **C** male, lateral habitus (scale bar 3 mm), and **D** female axillae and mesoscutellum, dorsal view (scale bar 0.5 mm; blue lines indicate the posterior extent of the axilla relative to the length of the mesoscutellum; red lines indicate the extent of the free portion of the axilla relative to its entire medial length).

#### Distribution.

**Figure 82.**
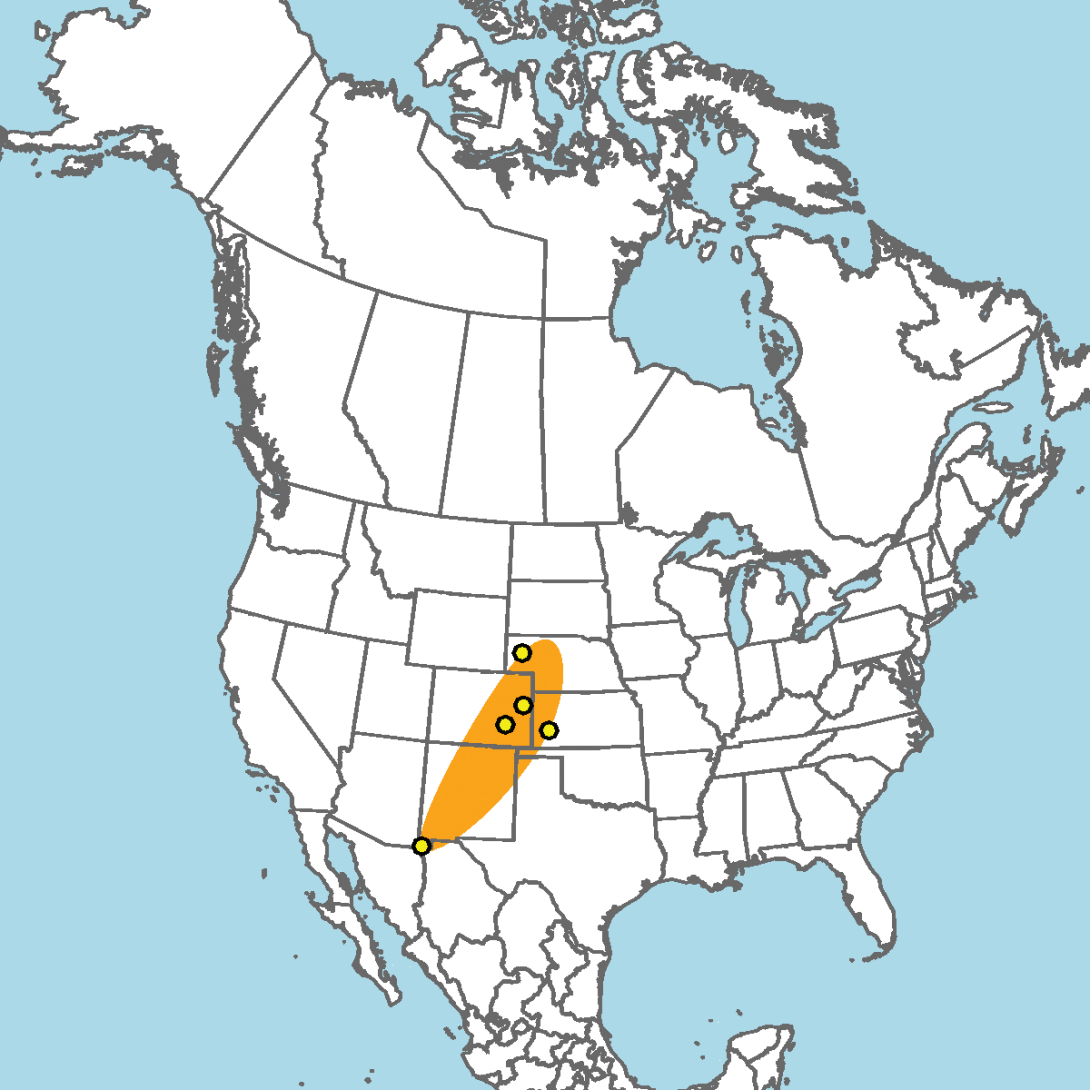
Approximate geographic range of *E. rufulus* (orange) based on occurrence records known to the author (yellow circles).

Great Plains to American southwest and presumably Mexico, given the close proximity of one collection locality (near Cloverdale, New Mexico) to the Mexico–United States border (Fig. 82).

#### Ecology.

HOST RECORDS: The host species of *E. rufulus* is/are presently unknown.

FLORAL RECORDS: The label of one examined voucher specimen indicates a floral association with Heterotheca subaxillaris ssp. latifolia.

#### Discussion.

In his unpublished thesis, Brumley (1965) synonymized *Epeolus rufulus* under *E. crucis*, treating the latter as a valid species. Herein, *E. crucis* is synonymized under *E. compactus* for reasons described in the Discussion of *E. compactus*. Also synonymized under *E. crucis* was *E. novomexicanus*, but morphological comparisons suggest that the type of *E. novomexicanus* belongs to the “*pusillus* group”. *Epeolus rufulus* is similar in overall appearance to *E. ainsliei* and *E. attenboroughi*, and the ranges of the three species overlap to some extent.

*Epeolus rufulus* appears to be uncommon, or at least uncommonly collected. The male of *E. rufulus* is described here for the first time. There is very little morphological variation among the few examined specimens, and in all the mesoscutum lacks distinct paramedian bands and is instead sparsely covered in pale tomentum.

#### Material studied.

**Type material.** Primary: USA: **Colorado**: Crowley, 01.ix.1932, M.T. James (holotype ♀ [CUM, catalog number: 0000043]).

#### DNA barcoded material with BIN-compliant sequences.

Available. BOLD:ADI5469. Specimens examined and sequenced.–USA: **Colorado**: Stratton (39.2645°N; 102.6681°W) (Kit Carson County), 22.viii.2014, A. Carper (1♂, CUM).

#### Non-barcoded material examined.

USA: **Kansas**: Finney (37.9411°N; 100.8811°W) (3.2 km S Garden City), 13.ix.2001, R.W. Brooks (1♀, KUNHM); **Nebraska**: 2 mi S Alliance (Box Butte County), 13.viii.1959, W.E. LaBerge (1♂, BBSL); **New Mexico**: ~6 mi E Cloverdale (31.4250°N; 108.8144°W) (Hidalgo County), 21.viii.2004, D. Yanega (1♀, UCR).

### 40. Epeolus scutellaris

Taxon classificationAnimaliaHymenopteraApidae

Say, 1824

Figs 83 , 84 , 97C

Epeolus scutellaris Say, 1824. In Keating, Narr. Long’s 2nd Exped., v. 2: 355 (♀); Onuferko, 2017. Can. J. Arthropod Identif. No 30: 44 (♀) [neotype designation].
Epeolus vernoniae Cockerell, 1907a. Entomologist 40: 136 (♂).

#### Diagnosis.

The following morphological features in combination (excluding any that are specific to the opposite sex of the one being diagnosed) can be used to tell *E. scutellaris* apart from all other North American *Epeolus*: the pronotal collar is predominantly black; the axilla is large, with the tip extending to or beyond the band of pale tomentum along the posterior margin of the mesoscutellum, dilated laterally, and ferruginous to some degree whereas the mesoscutellum ranges from entirely black to entirely ferruginous; the mesopleuron is closely (most i<1d) and evenly punctate and obscured by white tomentum only in the upper half (with a large, sparsely hairy circle occupying much of the ventrolateral half); the T1–T3 apical fasciae are complete or only very narrowly interrupted medially; and the pseudopygidial area of the female is lunate with the apex clearly >2 × the medial length. *Epeolus scutellaris* resembles *E. basili*, *E. nebulosus*, *E. novomexicanus*, and *E. pusillus* in that the axilla is large, with the lateral margin arcuate, and that the apical fasciae are complete or only very narrowly interrupted medially. However, in *E. scutellaris* the pseudopygidial area of the female is wider (the apex ~2.5–3 × the medial length) than in the four members of the “*pusillus* group” (the apex clearly <2.5 × the medial length). In all four members of the “*pusillus* group”, the mesopleuron of the male (excluding the hypoepimeral area) is entirely obscured by white tomentum and lacks the sparsely hairy circular area present in both sexes of *E. scutellaris*. *Epeolus scutellaris* is most similar to *E. packeri* in terms of surface sculpture and structure, but in *E. packeri* the pronotal collar is predominantly ferruginous, the T1 basal fascia is absent or reduced to a pair of small patches of pale tomentum, and the T1–T3 apical fasciae are interrupted medially and commonly reduced to discrete lateral patches. *Epeolus scutellaris* is also similar to *E. andriyi* and *E. howardi*, but in *E. andriyi* and *E. howardi* the T1–T3 apical fasciae are distinctly interrupted medially, and the pseudopygidial area of the female is lunate with the apex <2 × the medial length.

**Figure 83.**
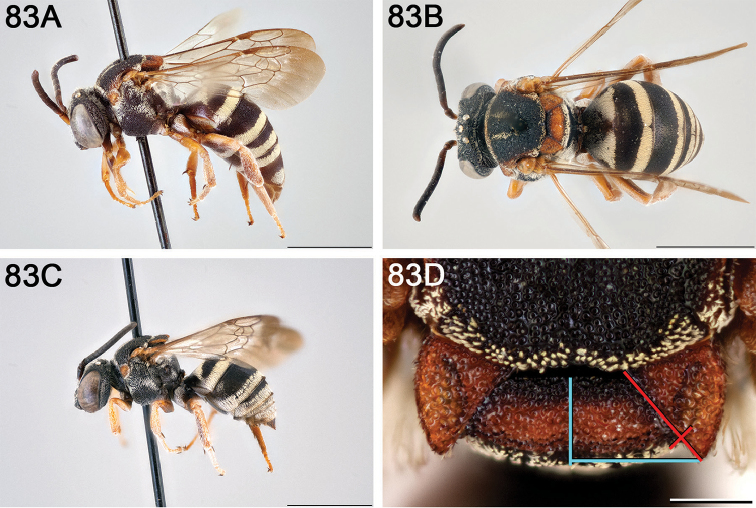
*Epeolus scutellaris* **A** female neotype, lateral habitus (scale bar 3 mm) **B** female neotype, dorsal habitus (scale bar 3 mm) **C** male, lateral habitus (scale bar 3 mm), and **D** female axillae and mesoscutellum, dorsal view (scale bar 0.5 mm; blue lines indicate the posterior extent of the axilla relative to the length of the mesoscutellum; red lines indicate the extent of the free portion of the axilla relative to its entire medial length).

#### Description.

This species was recently redescribed (Onuferko 2017).

#### Distribution.

**Figure 84.**
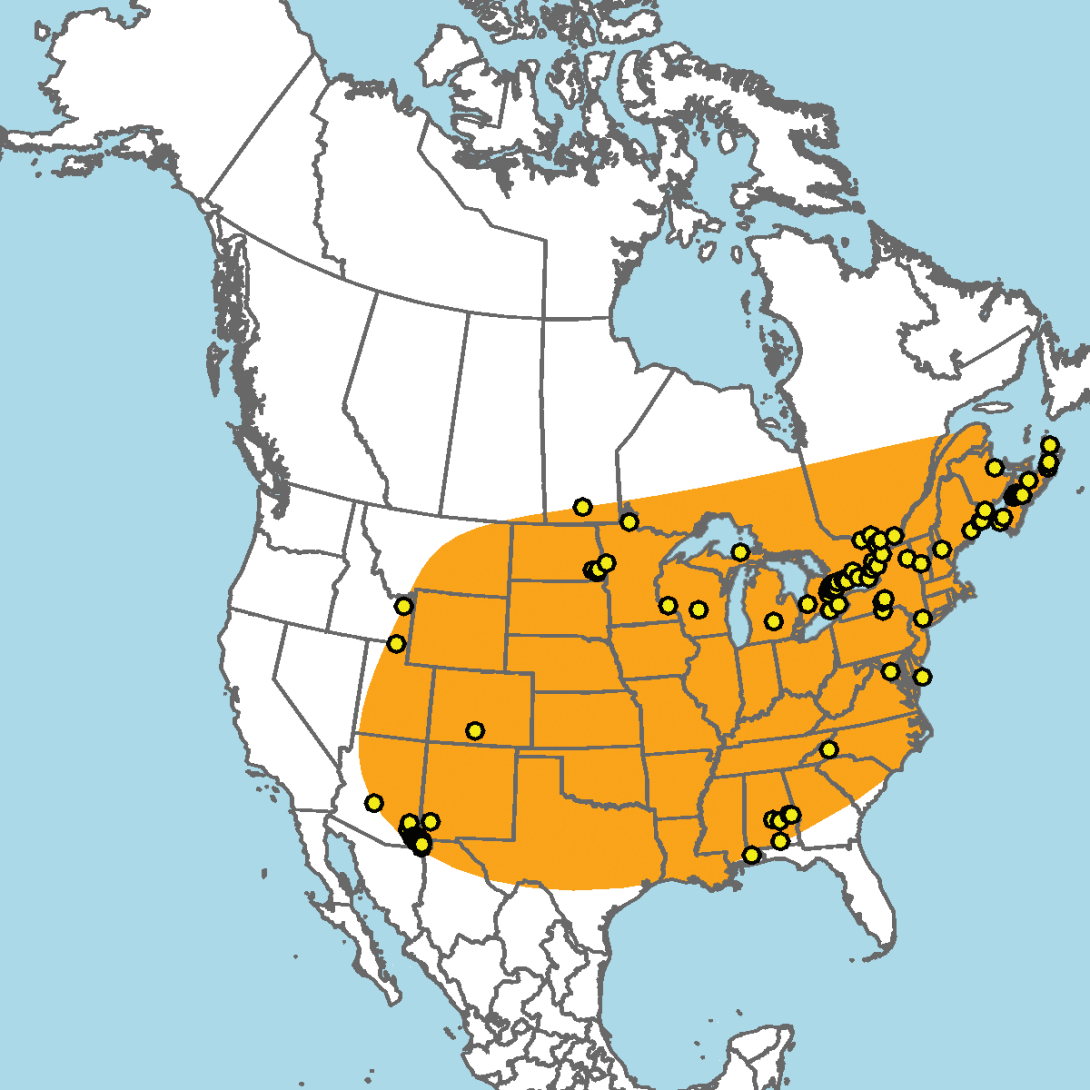
Approximate geographic range of *E. scutellaris* (orange) based on occurrence records known to the author (yellow circles).

Widely distributed across the contiguous United States, excluding peninsular Florida and the west coast, and southern Canada (Maritime to Prairie provinces) (Fig. 84).

#### Ecology.

See Onuferko (2017) for host and floral records. Floral associations are also indicated in Suppl. material 1, which includes newly discovered associations with *Chrysothamnus* (possibly in reference to plants that now are in the genus *Ericameria*), *Erigeron*, and *Heterotheca subaxillaris* based on labels of examined voucher specimens.

#### Discussion.

In Onuferko (2017), *E. scutellaris* is said to be similar to two species from Florida yet to be formally recognized, which herein are formally described under the names *Epeolus deyrupi* and *E. packeri*. Detailed morphological and taxonomic remarks about this species are given in Onuferko (2017).

#### Material studied.

**Type material.** Primary: USA: **New York**: Keene Valley (Essex County), 12.viii.1917, H. Notman (*E. scutellaris* neotype ♀, AMNH); **Virginia**: Falls Church, 04.ix.????, N. Banks (*E. vernoniae* holotype ♂, AMNH).

#### DNA barcoded material with BIN-compliant sequences.

Available. BOLD:AAG5250. Specimens examined and sequenced.–Canada: **Nova Scotia**: 1♀ (RSKM); **Ontario**: 1♀, 1♂ (PCYU).

USA: **Alabama**: Autauga County (32.4345°N; 86.5817°W), 19.x.2016, C.H. Ray (1♂, AUMNH); Lee County (32.5553°N; 85.3747°W), 09.x.2016, C.H. Ray (1♀, AUMNH); Montgomery (32.3135°N; 86.1744°W) (Montgomery County), 01.x.2016, A. Jeon (1♀, AUMNH); **Idaho**: 2♂ (AMNH).

#### Non-barcoded material examined.

Canada: **Manitoba**: Canadian Forces Base Portage la Prairie, 03.ix.1974, T.D. Galloway (2♀, JBWM); **New Brunswick**: 1♀, 1♂ (CNC); **Nova Scotia**: 11♀, 8♂ (CNC, PCYU, RSKM); Brooklyn Street (near Kentville, Kings County), 15.ix.2005, C. Sheffield and S. Westby (1♀, PCYU); Port Hawkesbury Station (Cape Breton Island), 03.ix.1985, L. Packer (1♀, PCYU); **Ontario**: 29♀, 29♂ (CNC, PCYU, ROM); Lambton County, 29.viii.2007, A. Taylor (1♀, PCYU); Marshlands Conservation Area (Kingston, Frontenac County), 20.viii.2016, J. Gibbs (2♀, JBWM); Norwood, 24.viii.1982, T.D. Galloway (1♀, JBWM); Ottawa Airport, 03.ix.1985, L. Packer (1♀, PCYU); Rockwood, 22.ix.1972, T.D. Galloway (1♀, JBWM); **Quebec**: 3♀, 2♂ (CNC).

USA: **Alabama**: Auburn (32.6005°N; 85.5102°W) (Lee County), 15.x.2016, C.H. Ray (1♀, AUMNH); Autauga County (32.4345°N; 86.5817°W), 19.x.2016, C.H. Ray (1♀, AUMNH); Covington County (31.2550°N; 86.2887°W), 05.xi.2016, C.H. Ray (2♀, AUMNH); Lee County (32.5553°N; 85.3747°W), 09.x.2016, C.H. Ray (3♀, AUMNH); Mobile Botanical Gardens (30.7010°N; 88.1606°W) (Mobile County), 27.ix.2016, C.H. Ray (1♂, AUMNH); **Arizona**: 17 mi S Safford, 22.viii.1986, R.R. Snelling (1♂, LACM); 4 mi E Willcox (Cochise County), 28.viii.1985, J.G and B.L. Rozen (2♂, AMNH); 5 mi S Apache (Cochise County), 12.ix.1976, R.M. Bohart (1♂, UCBME); 5 mi W Portal (Cochise County), 31.viii.2003, J.S. Ascher (1♂, AMNH); Near Portal (Cochise County), 08.ix.2011, A. Payne (1♀, AMNH); Phoenix (Maricopa County), 13.x.1997, K.C. Rozen (1♂, AMNH); W Turkey Creek (Chiricahua Mountains), 02.ix.2003, J.G. Rozen, J.S. Ascher, R.L. Staff, and R.E. Edwards (1♀, AMNH); **Colorado**: 2.4 mi N Hooper (Saguache County), 24.viii.1967, R.R. Snelling (1♂, LACM); **Maine**: 2♀, 1♂ (BIML); **Maryland**: 4♂ (BIML); **Michigan**: 1♀ (BIML); East Lansing (Ingham County), 03.ix.2016, J. Gibbs (1♂, JBWM); **Minnesota**: 3 mi E Glyndon (Clay County), 15.ix.1986, J.R. Powers (1♂, EMEC); Wabasha (Wabasha County), 17.viii.1995, J.R. Powers (2♀, 5♂, EMEC); **New Hampshire**: North Conway, Bequaert (1♂, EMEC); **New Jersey**: 1♂ (AMNH); **New Mexico**: 17 mi S Animas (Hidalgo County), 24.viii.1994, J.G. Rozen and J.S. Ascher (1♂, AMNH); 17 mi S Animas (Hidalgo County), 30.viii.1994, J.G. Rozen and J.S. Ascher (1♀, AMNH); 26 mi S Animas (Hidalgo County), 22.viii.1997, J.G. Rozen and B. McAdams (1♂, AMNH); 27–32 mi S Animas (Hidalgo County), 24.viii.1994, J.G. Rozen and J.S. Ascher (1♂, AMNH); 29–31 mi S Animas (Hidalgo County), 30.viii.1994, J.G. Rozen and J.S. Ascher (1♂, AMNH); 3 mi S Rodeo (Hidalgo County), 07.ix.2003, J.S. Ascher (1♂, AMNH); Cienega (Hidalgo County), 28.viii.1997, J.G. Rozen and B. McAdams (1♂, AMNH); Rodeo (Hidalgo County), 07.ix.1976, R.M. Bohart (1♂, UCBME); U.S. Route 180 (11 mi SE Mangas, Grant County), 04.ix.2011, J.G Rozen and E.S. Wyman (1♂, AMNH); **New York**: Cornell Botanic Gardens (42.4497°N; 76.4711°W) (Cornell University, Tompkins County), 19.viii.2012, J. Gibbs (1♀, JBWM); Lime Hollow (42.5650°N; 76.2550°W) (Cortland County), 03.ix.2011, J. Gibbs (1♀, JBWM); Mundy Wildflower Garden (42.4510°N; 76.4690°W) (Cornell University, Tompkins County), 18.viii.2012, J. Gibbs (1♂, JBWM); **North Carolina**: 1♂ (AMNH); **North Dakota**: 1 mi SE McLeod (Ransom County), 19.viii.1988, J.R. Powers (1♀, EMEC), 10.ix.1997, J.R. Powers (1♀, EMEC); 11 mi W Walcott (Richland County), 08.ix.1987, J.R. Powers (2♀, EMEC), 02.ix.1996, J.R. Powers (1♀, EMEC); 7 mi SE Sheldon (Ransom County), 19.viii.1980, J.R. Powers (2♀, 1♂, EMEC), 28.viii.1981, J.R. Powers (1♀, EMEC), 09.viii.2000, J.R. Powers (1♂, EMEC), 26.vii.1985, J.R. Powers (1♂, EMEC); **Pennsylvania**: Wilawana, 08–10.1934, R.H. Crandall (1♂, LACM); **Utah**: Cornish (Cache County), 04.ix.1982, R.M. Bohart (1♀, UCBME); **Vermont**: 1♀, 2♂ (AMNH); **Wisconsin**: 1♀ (FMNH).

### 41. Epeolus splendidus sp. n.

Taxon classificationAnimaliaHymenopteraApidae

http://zoobank.org/28EE92F6-A0DE-403A-B0B8-157239AEAB5F

Figs 85 , 86 , 102A

Epeolus politus Brumley, 1965. M.S. thesis, Utah State University, Logan 60 (♀) [*nomen nudum*].

#### Diagnosis.

The following morphological features in combination can be used to tell *E. splendidus* apart from all other North American *Epeolus*: the propodeum (except the textured metapostnotum) is highly polished and (except along the lateral margins) hairless, and T1 has a complete white basal fascia whereas T1–T4 have complete bright yellow apical fasciae. As in *E. canadensis*, *E. compactus*, and *E. ferrarii*, in *E. splendidus* the mesoscutum has a small anteromedial patch of tomentum, although it is bright rather than pale yellow. However, in *E. splendidus* T1 lacks a distinct black discal patch and in females F2 is shorter, as long as wide. In all four species, the axilla does not attain the midlength of the mesoscutellum, and the axilla (except sometimes the tip) and mesoscutellum are black.

#### Description.

FEMALE: Length 8.4 mm; head length 2.1 mm; head width 3.0 mm; fore wing length 6.6 mm.

*Integument coloration.* Mostly black; notable exceptions as follows: partially to entirely ferruginous on mandible, antenna, pronotal lobe, tegula, legs, metasomal terga (including pygidial plate), and metasomal sterna. Mandible with apex and preapical tooth darker than rest of mandible. Antenna brown except scape and pedicel orange in part. Pronotal lobe and tegula pale ferruginous to amber. Wing membrane subhyaline, apically dusky. Legs with brown or black more extensive than reddish orange.

*Pubescence.* Face with tomentum densest around antennal socket. Dorsum of mesosoma and metasoma with bands of off-white and bright yellow short appressed setae. Pronotal collar with tomentum sparser medially, uniformly bright yellow. Mesoscutum with anteromedial chevron-shaped patch of bright yellow tomentum. Mesopleuron with upper half densely hairy, except beneath base of fore wing (hypoepimeral area); ventrolateral half sparsely hairy. Metanotum with tomentum uninterrupted, uniformly off white. T1 with broad, off-white basal fascia, complete bright yellow apical fascia, and narrow and extremely short discal patch of dark brown tomentum. T2–T4 each with complete bright yellow fascia, T2 and T3 with fasciae with anterolateral spots of sparser off-white tomentum. T5 covered in off-white tomentum except for line of separation from pseudopygidial area. T5 with pseudopygidial area lunate, its apex more than twice as wide as medial length, indicated by silvery setae on flat disc of apicomedial region elevated from rest of tergum. S5 with apical fimbria of coppery to silvery hairs extending beyond apex of sternum by ~2/5 MOD.

*Surface sculpture.* Punctures dense. Labrum with larger and sparser punctures (i=1–2d) than clypeus (i<1d). Small impunctate shiny spot lateral to lateral ocellus. Mesoscutum, mesoscutellum, and axilla coarsely and densely rugose-punctate. Tegula very densely punctate mesally (i<1d), less so laterally (i=1–2d). Mesopleuron with ventrolateral half densely punctate (i≤1d) to rugose; mesopleuron with punctures more or less equally dense throughout. Metasomal terga with punctures very fine, dense (i≈1d), evenly distributed on disc.

*Structure.* Labrum with pair of small subapical denticles, each preceded by small discrete longitudinal ridge. Frontal keel not strongly raised. Scape with greatest length 1.9 × greatest width. F2 as long as wide (L/W ratio = 1.0). Preoccipital ridge not joining hypostomal carina, from which it is separated by no less than 1 MOD at its terminal. Mesoscutellum weakly bigibbous. Axilla small to intermediate in size, its lateral margin (L) less than half as long as mesoscutellar width (W) (L/W ratio = 0.3) and tip not extending beyond midlength of mesoscutellum; axilla with tip clearly visible, but unattached to mesoscutellum for less than 1/3 the medial length of axilla; axilla with lateral margin relatively straight and without carina. Fore wing with three submarginal cells. Pygidial plate apically truncate.

MALE: Description as for female except for usual secondary sexual characters and as follows: F2 shorter, nearly as long as wide (L/W ratio = 0.95); S4 and S5 with much longer coppery to silvery subapical hairs; pygidial plate apically rounded, with large deep punctures closely clustered basomedially and sparser apically and laterally, with the interspaces shining.

**Figure 85.**
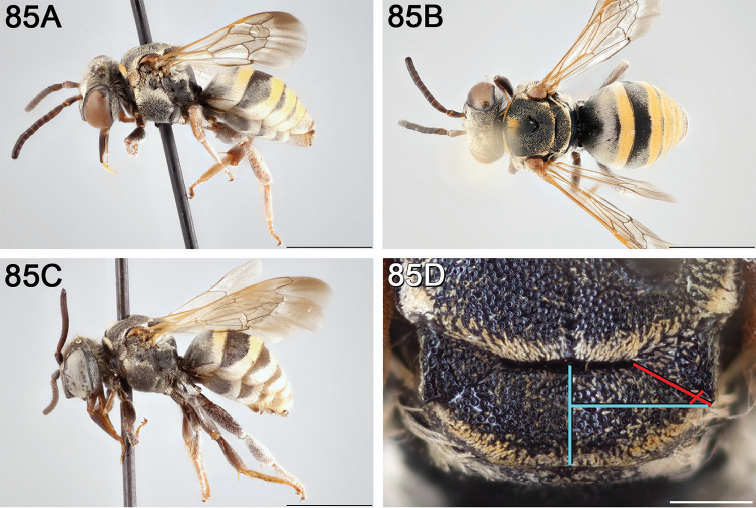
*Epeolus splendidus* **A** female holotype, lateral habitus (scale bar 3 mm) **B** female holotype, dorsal habitus (scale bar 3 mm) **C** male paratype, lateral habitus (scale bar 3 mm), and **D** female paratype axillae and mesoscutellum, dorsal view (scale bar 0.5 mm; blue lines indicate the posterior extent of the axilla relative to the length of the mesoscutellum; red lines indicate the extent of the free portion of the axilla relative to its entire medial length).

#### Etymology.

The name is in reference to the uniquely smooth, shiny propodeum of this species. From the Latin, “splendidus” (bright).

#### Distribution.

**Figure 86.**
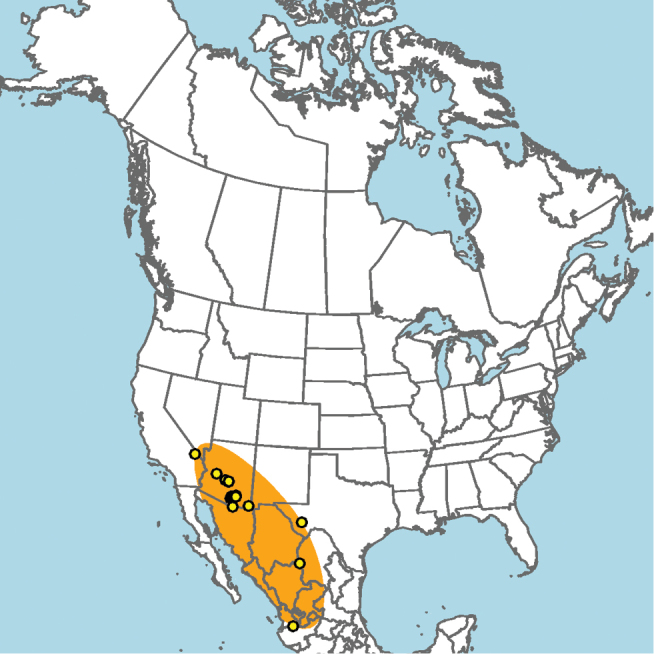
Approximate geographic range of *E. splendidus* (orange) based on occurrence records known to the author (yellow circles).

Known to occur in all major hot North American deserts (Fig. 86).

#### Ecology.

HOST RECORDS: The female PCYU paratype (see Material studied) was collected in the spring of 2015 along the Catalina Highway in Pima County, Arizona, USA where possible host *Colletes* visiting *Eriogonum* Michx. were collected and observed. Using Stephen’s (1954) key, collected females were identified as *C. wootoni* Cockerell (one of which was sequenced and assigned the same BIN [BOLD:AAI9255] as a male from New Mexico whose terminalia were excised for identification) whereas collected males (one of which was sequenced and assigned the following BIN: BOLD:ABZ4837) were identified (based in part on examination of the terminalia, which were excised) as *C. eulophi*.

FLORAL RECORDS: Labels of examined voucher specimens indicate floral associations with *Baileya* Harv. & A. Gray ex A. Gray, *Encelia farinosa* A. Gray ex Torr. (Compositae), *Eriogonum inflatum* Torr. & Frém., *Larrea* Cav., *Parkinsonia* L. (Leguminosae), and *Prosopis velutina*, and BugGuide (http://www.bugguide.net/) indicates an association with *Erigeron*.

#### Discussion.

This southwestern species was identified as unique by Brumley (1965), and the colors and patterns of pubescence on the mesosoma and metasoma clearly set it apart from other *Epeolus* in North America. There is very little morphological variation among examined specimens, and sequenced material was assigned the same BIN. Based on known records, adults of *E. splendidus* are active in spring.

#### Material studied.

**Type material.** Primary: USA: **Arizona**: Usery Mountains (Mesa, Maricopa County), iv.2009, J. Alcock (holotype ♀ [CCDB-28230 D07], AMNH).

Secondary: Mexico: **Durango**: Reserva de la Biósfera de Mapimí (26.6803°N; 103.7408°W), 24.iii.1995, R. López (paratype ♂, BBSL); **Jalisco**: Plan de Barrancas, 24.iii.1962, F.D. Parker (paratype ♂, UCBME).

USA: **Arizona**: 11 mi SW Congress (Yavapai County), 29.iv.1990, J.G. Rozen (paratype ♂, AMNH); 14 mi SW Apache (Cochise County), 22.v.1988, J.G. Rozen (paratype ♀, AMNH); 2 mi E Tanque Verde (Pima County), 14.iii.??54, F. Werner (paratype ♂, LACM); 20 mi NE Mesa (Maricopa County), 28.iv.1988, P. Robinson (paratype ♂, CUM); 8 km E Robles Junction (32.0667°N; 111.2500°W) (Tucson, Pima County), 15–27.iv.1996, D. Yanega (paratype ♀, UCR); Arizona-Sonora Desert Museum/Tucson Mountain Park (Pima County), 11–12.iv.1988, K. Krombein and B. Norden (paratypes 2♂, USNM); Catalina Hwy (32.3631°N; 110.7137°W) (Santa Catalina Mountains, Coronado National Forest), 29.v.2015, A.T. Onuferko (paratype ♀ [CCDB-22013 E11], PCYU); E Calle del Prado & N Palo Verde Ave (Tucson, Pima County), 09.iv.1997, R. Minckley (paratype ♂, BBSL); Mouth of Bear Canyon (Tucson, Santa Catalina Mountains), 29.iii.1964, F.G. Werner (allotype ♂, KUNHM); Nogales (Santa Cruz County), 20.iv.1967, P. Torchio and N. Youssef (paratype ♂, BBSL); Phoenix (33.6185°N; 111.9917°W) (Maricopa County), 17–19.iv.2009, J.G. Rozen (paratype ♀, AMNH); Sabino Canyon (near Tucson, Pima County), 03.iv.1972, B. Simpson (paratype ♂, LACM); Tucson (Pima County), 07.v.1987, J.G. Rozen (paratype ♀, AMNH); **California**: Clark Mountain (35.5217°N; 115.6428°W) (San Bernardino County), 23.v.2001, D. Yanega (paratype ♂, UCR); **Texas**: Alpine (Brewster County), 29.v.1952, M. Cazier, W. Gertsch, and R. Schrammel (paratype ♀, AMNH).

#### DNA barcoded material with BIN-compliant sequences.

Available. BOLD:ACX0474. See Type material for specimens examined and sequenced (indicated by unique CCDB-plate and well number).

### 42. Epeolus tessieris sp. n.

Taxon classificationAnimaliaHymenopteraApidae

http://zoobank.org/D50F6D82-115B-4DF0-B638-80022CDB537B

Figs 87 , 88 , 92H

Epeolus cretus Brumley, 1965. M.S. thesis, Utah State University, Logan 42 (♀) [*nomen nudum*].

#### Diagnosis.

The following morphological features in combination can be used to tell *E. tessieris* apart from all other North American *Epeolus* except *E. interruptus*: the axilla does not attain the midlength of the mesoscutellum, its tip is unattached to the mesoscutellum for less than 1/3 of the entire medial length of the axilla, and like the mesoscutellum is ferruginous; the mesopleuron has sparser punctures ventrolaterally (most i≥1d) than in upper half, with the interspaces shining; and T1–T4 have medially-interrupted metasomal fasciae. Whereas in *E. interruptus* the metanotum has a blunt median process and T1 has a wide triangular discal patch with concave lateral sides, in *E. tessieris* the metanotum is flat and T1 has a trapezoidal to nearly semicircular discal patch.

#### Description.

FEMALE: Length 5.8 mm; head length 1.7 mm; head width 2.3 mm; fore wing length 4.8 mm.

*Integument coloration.* Mostly black; notable exceptions as follows: partially to entirely ferruginous on mandible, labrum, antenna, pronotal lobe, tegula, axilla, mesoscutellum, and legs. Mandible with apex darker than rest of mandible; preapical tooth lighter than mandibular apex (difficult to see in holotype because mandible closed; described from paratypes). Antenna brown and orange in part. Pronotal lobe and tegula pale ferruginous to amber. Wing membrane subhyaline, apically dusky. Legs more extensively reddish orange than brown or black.

*Pubescence.* Face with tomentum densest around antennal socket. Dorsum of mesosoma and metasoma with bands of off-white to pale yellow short appressed setae. Mesoscutum with paramedian band. Mesopleuron with upper half hairy, except beneath base of fore wing (hypoepimeral area); ventrolateral half nearly bare. Metanotum with tomentum sparser medially, uniformly off white. T1 with median trapezoidal verging on semicircular black discal patch enclosed by pale tomentum, except for medial separations at base and apex. T2–T4 with fasciae interrupted medially and narrowed before becoming somewhat broader laterally, T2 with fascia with anterolateral extensions of sparser tomentum. T5 with two large patches of pale tomentum anterolateral to and separate from pseudopygidial area. T5 with pseudopygidial area lunate, its apex more than twice as wide as medial length, indicated by silvery setae on impressed disc of apicomedial region elevated from rest of tergum. S5 with apical fimbria of coppery to silvery hairs not extending beyond apex of sternum by much more than 1/4 MOD.

*Surface sculpture.* Punctures dense, except those of mesopleuron. Labrum with larger punctures than clypeus, but punctures of both equally dense (i≤1d). Small impunctate shiny spot lateral to lateral ocellus. Mesoscutum, mesoscutellum, and axilla coarsely and densely rugose-punctate. Tegula densely punctate (i≤2d). Mesopleuron with denser (i≤1d) punctures in upper half than ventrolateral half (i>1d, largely impunctate areas below line of pale tomentum), the interspaces shining. Metasomal terga with punctures very fine, dense (i≈1d), evenly distributed on disc.

*Structure.* Preapical tooth blunt and obtuse. Labrum with submedial pair of very small denticles, apex with pair of small points separated by shallow concavity (difficult to see in holotype; described from paratypes). Frontal keel not strongly raised. Scape with greatest length 1.8 × greatest width. F2 noticeably longer than wide (L/W ratio = 1.4). Preoccipital ridge not joining hypostomal carina, from which it is separated by about 1.5 MOD at its terminal. Mesoscutellum moderately bigibbous. Axilla small to intermediate in size, its lateral margin (L) less than half as long as mesoscutellar width (W) (L/W ratio = 0.4) and not extending beyond midlength of mesoscutellum; axilla with tip visible, but unattached to mesoscutellum for less than 1/3 the medial length of axilla; axilla with lateral margin relatively straight and without carina. Fore wing with three submarginal cells. Pygidial plate apically truncate.

MALE: Description as for female except for usual secondary sexual characters and as follows: F2 shorter, not noticeably longer than wide (L/W ratio = 1.1); S4 and S5 with much longer coppery to silvery subapical hairs; pygidial plate apically rounded, with large deep punctures closely clustered basomedially and sparser apically and laterally, with the interspaces shining.

**Figure 87.**
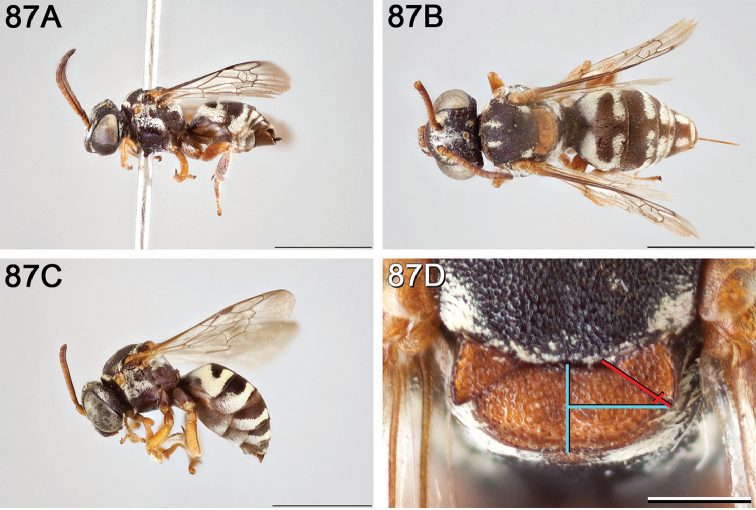
*Epeolus tessieris* **A** female holotype, lateral habitus (scale bar 3 mm) **B** female paratype, dorsal habitus (scale bar 3 mm) **C** male paratype, lateral habitus (scale bar 3 mm), and **D** female paratype axillae and mesoscutellum, dorsal view (scale bar 0.5 mm; blue lines indicate the posterior extent of the axilla relative to the length of the mesoscutellum; red lines indicate the extent of the free portion of the axilla relative to its entire medial length).

#### Etymology.

This species is named in honor of my wife, biologist Stéphanie Tessier. The name is in the genitive case and declined as *mulier*, a Latin noun with a consonant stem.

#### Distribution.

**Figure 88.**
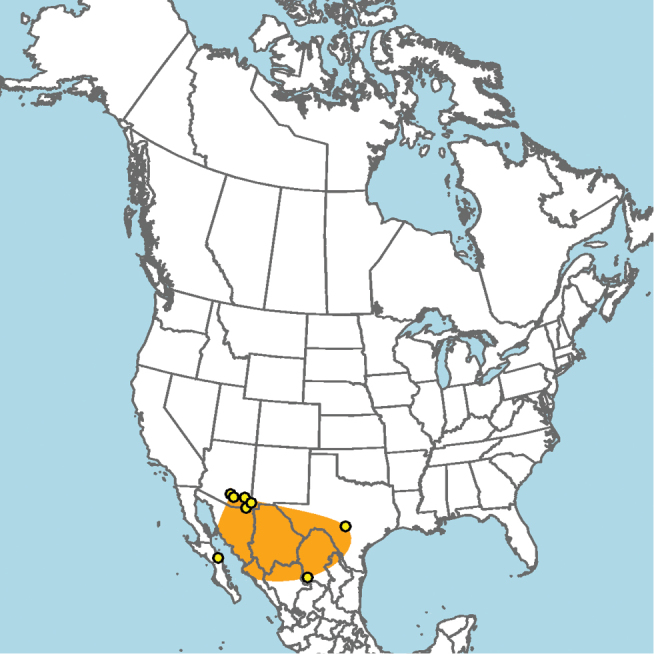
Approximate geographic range of *E. tessieris* (orange) based on occurrence records known to the author (yellow circles).

Northern Mexico and bordering U.S. States (Fig. 88).

#### Ecology.

HOST RECORDS: The host species of *E. tessieris* is/are presently unknown.

FLORAL RECORDS: Labels of examined voucher specimens indicate floral associations with *Cuscuta umbellata* Kunth (Convolvulaceae), *Marshallia* Schreb. (Compositae), and *Pectis papposa*.

#### Discussion.

Of the *Epeolus* Brumley (1965) identified as new, this appears to be the least commonly collected species. Among examined specimens, there is notable variability in punctation density of the mesopleuron, but the smooth, shiny interspaces are usually greater than puncture diameters. Although BIN-compliant sequences are presently not available for *E. tessieris*, 421 bp sequences are available for two specimens (a female from Arizona, USA and a male from Coahuila, Mexico), and there is virtually no divergence (<1%) between the two. Moreover, these sequences do not cluster closely with any sequences from other *Epeolus* species in a NJ tree (Suppl. material 2).

#### Material studied.

**Type material.** Primary: USA: **Arizona**: 3 mi W Marana (Pima County), 13.ix.1962, J.C. Bequaert (holotype ♀, CAS).

Secondary: Mexico: **Baja California Sur**: Playa El Coyote (26 km SSE Mulegé), 08.ix.1977, E. Fisher and R. Westcott (paratype ♂, CAS); **Coahuila**: 7 km SE Zapata, 25.viii.1991, J.G. Rozen (paratype ♂, KUNHM).

USA: **Arizona**: 1 mi E Douglas (Cochise County), 17.viii.1962, M.A. Cazier (paratype ♂, UCBME); 3 mi W Marana (Pima County), 13.ix.1962, J.C. Bequaert (allotype ♂, KUNHM); 4 mi E Willcox (Cochise County), 30.viii.2004, J.G. Rozen and J.S. Ascher (paratype ♀, AMNH); Tucson (Pima County), 27.x.1939, R.H. Crandall (paratype ♀, LACM); **New Mexico**: 1 mi N Rodeo (Hidalgo County), 22.viii.1964, J.H. Puckle, M.A. Mortenson, and M.A. Cazier (paratype ♂, EMEC); **Texas**: Kerrville, 31.v.??06, F.C. Pratt (paratype ♀, USNM).

#### DNA barcoded material with BIN-compliant sequences.

Unavailable.

### 43. Epeolus zonatus

Taxon classificationAnimaliaHymenopteraApidae

Smith, 1854

Figs 89 , 90 , 97H

Epeolus zonatus Smith, 1854. Cat. Hym. Brit. Mus. 2: 257 (♀, ♂), **new lectotype designation.**

#### Diagnosis.

The following morphological features in combination (excluding any that are specific to the opposite sex of the one being diagnosed) can be used to tell *E. zonatus* apart from all other North American *Epeolus* except *E. erigeronis*, *E. ilicis*, and *E. inornatus*: the mandible is simple; the axilla does not attain the midlength of the mesoscutellum but the free portion is distinctly hooked, with the tip unattached to the mesoscutellum for more than 1/3 of the entire medial length of the axilla; and the pseudopygidial area of the female is distinctly campanulate with the apex <2 × the medial length. Whereas in *E. erigeronis*, *E. ilicis*, and *E. inornatus* the pronotal collar and metasomal terga are black, as are sometimes the axilla and mesoscutellum, in *E. zonatus* the pronotal collar, axilla, mesoscutellum, T1, and T2 are ferruginous. Also, in *E. zonatus* the dorsum of the mesosoma and metasoma is commonly with much less pale pubescence.

#### Redescription.

FEMALE: Length 9.7 mm; head length 2.3 mm; head width 3.1 mm; fore wing length 6.2 mm.

*Integument coloration.* Black in part, at least partially ferruginous on mandible, labrum, clypeus, antenna, pronotal collar, pronotal lobe, tegula, axilla, mesoscutum, mesoscutellum, metanotum, mesopleuron, legs, T1, T2, and metasomal sterna. Mandible with apex darker than all but extreme base. Antenna brown and orange in part. Pronotal lobe and tegula pale ferruginous to amber. Mesoscutum reddish-brown along lateral margin and with pair of reddish-brown markings near posterior margin between midline and parapsidal line. Wing membrane dusky subhyaline, slightly darker at apex. Legs more extensively reddish orange than brown or black.

*Pubescence.* Face with tomentum densest around antennal socket. Clypeus, upper paraocular and frontal areas, and vertexal area mostly exposed. Mesoscutum without pale tomentum. Dorsum of metasoma with bands of off-white short appressed setae. Mesopleuron nearly bare, except along margins. Metanotum with tomentum sparser medially, uniformly off white. T1 with discal patch quadrangular and very wide, the basal and apical fasciae at most only narrowly joined laterally (not joined in lectotype and multiple non-type specimens). T1 with basal and apical fasciae and T2–T3 with apical fasciae widely separated medially, the apical fasciae reduced to pairs of small patches somewhat broader laterally, T2 with fascia without anterolateral extensions of tomentum. T4 with fascia much more narrowly interrupted medially than on preceding terga. T5 with two faint patches of pale tomentum lateral to and contacting pseudopygidial area at apex, diverging from pseudopygidial area basally. T5 with pseudopygidial area campanulate, its apex less than twice as wide as medial length, indicated by silvery setae on impressed disc of apicomedial region elevated from rest of tergum. S5 with apical fimbria of coppery to silvery hairs extending beyond apex of sternum by ~2/5 MOD.

*Surface sculpture*. Punctures dense. Labrum with larger and sparser punctures (i=1–2d) than clypeus (i<1d). Small impunctate matte spot lateral to lateral ocellus. Mesoscutum, mesoscutellum, and axilla coarsely and densely rugose-punctate. Tegula very densely punctate mesally (i<1d), less so laterally (i=1–2d). Mesopleuron with denser (i≤1d) punctures in upper half than ventrolateral half (i≤2d), the interspaces shining; mesopleuron with punctures similar in size throughout. Metasomal terga with punctures very fine, dense (i=1–2d), evenly distributed on disc; the interspaces shining somewhat.

*Structure*. Mandible without preapical tooth. Labrum with pair of small subapical denticles not preceded by carinae. Frontal keel not strongly raised. Scape with greatest length 1.9 × greatest width. F2 noticeably longer than wide (L/W ratio = 1.4). Preoccipital ridge not joining hypostomal carina, from which it is separated by no less than 1 MOD at its terminal. Mesoscutellum moderately bigibbous. Axilla intermediate in size, its lateral margin (L) nearly half as long as mesoscutellar width (W) (L/W ratio = 0.4–0.5) and tip not extending beyond midlength of mesoscutellum; axilla with tip conspicuously diverging from side of mesoscutellum, distinctly hooked, and axilla with free portion 2/5 its medial length; axilla with lateral margin relatively straight and carinate. Fore wing with three submarginal cells. Pygidial plate apically truncate.

MALE: Description as for female except for usual secondary sexual characters and as follows: F2 shorter, not noticeably longer than wide (L/W ratio = 1.1); S4 and S5 with much longer coppery to silvery subapical hairs; pygidial plate apically rounded, with large deep punctures more or less evenly spaced throughout, with the interspaces shining.

**Figure 89.**
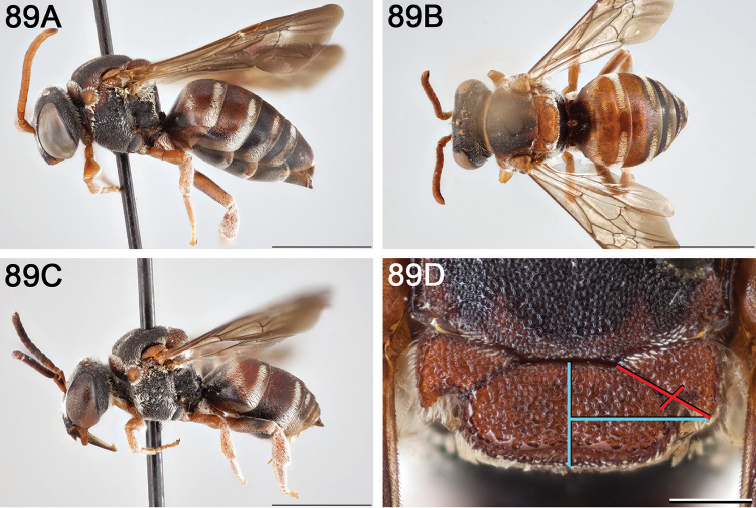
*Epeolus zonatus* **A** female, lateral habitus (scale bar 3 mm) **B** female, dorsal habitus (scale bar 3 mm) **C** male, lateral habitus (scale bar 3 mm), and **D** female axillae and mesoscutellum, dorsal view (scale bar 0.5 mm; blue lines indicate the posterior extent of the axilla relative to the length of the mesoscutellum; red lines indicate the extent of the free portion of the axilla relative to its entire medial length).

#### Distribution.

**Figure 90.**
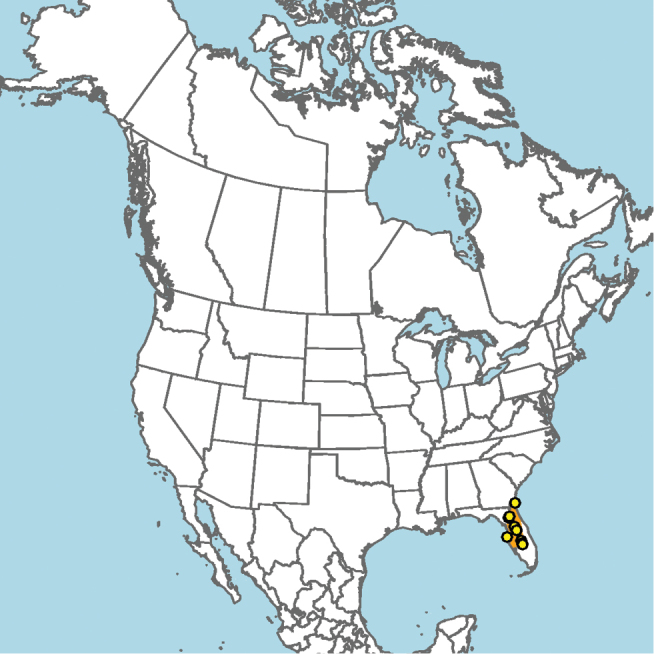
Approximate geographic range of *E. zonatus* (orange) based on occurrence records known to the author (yellow circles).

Florida and coastal Georgia (Fig. 90).

#### Ecology.

HOST RECORDS: The host species of *E. zonatus* is/are presently unknown.

FLORAL RECORDS: Mitchell (1962) indicated floral associations with *Crataegus* L. (Rosaceae) and *Prunus* L. Labels of examined voucher specimens further indicate associations with *Ambrosia artemisiifolia* L. (Compositae), *Aralia spinosa*, *Clinopodium ashei*, *Ilex cassine* L., *I. glabra*, *Licania michauxii*, *Persea borbonia* (L.) Spreng. (Lauraceae), *Prunus angustifolia*, and *Serenoa repens*.

#### Discussion.

Smith (1854) described *E. zonatus* from both sexes, represented by three syntypes (all females) deposited at the NHMUK. The male description is actually based on a female specimen (see *E. zonatus* paralectotype [catalog number: 010812211] under Type material) of another species (*E. bifasciatus*). All three specimens were examined, and one of the two females of the true *E. zonatus* is herein designated as the lectotype, the one that is in better condition that fits Smith’s (1854) original description of the female.

Structurally, *E. zonatus* and *E. ilicis* are identical, but in *E. zonatus* the pronotal collar, axilla, mesoscutellum, and discs of T1 and T2 are ferruginous, whereas in *E. ilicis* at least the pronotal collar and metasomal terga are entirely black. These are the exact same features that separate *E. glabratus* (another species restricted to peninsular Florida and coastal Georgia) from *E. lectoides*. Presently, only a single 422 bp sequence is available for *E. ilicis* (a male specimen from Florida, USA), which clusters with sequences of *E. zonatus* (Suppl. material 2), and all were assigned the same BIN. However, as the morphological differences between the two species are consistent, and because there appears to be little overlap in the ranges of both species, I have opted to treat *E. ilicis* and *E. zonatus* as heterospecific, despite the apparent lack of evidence of genetic divergence. This is another example of red-marked Hymenoptera in Florida with black congeners elsewhere (see Deyrup and Eisner 2003).

#### Material studied.

**Type material.** Primary: USA: **Florida**: (*E. zonatus* lectotype ♀ [NHMUK, catalog number: 010812210]).

Secondary: USA: **Florida**: St. Johns Bluff (*E. zonatus* paralectotypes 2♀ (1 numbered [NHMUK, catalog number: 010812211])).

#### DNA barcoded material with BIN-compliant sequences.

Available. BOLD:ACM5887. Specimens examined and sequenced.–USA: **Florida**: Archbold Biological Station (Highlands County), 17–23.iv.2007, S.M. Paiero (1♀, DEBU); **Georgia**: Cumberland Island National Seashore (30.8264°N; 81.4369°W) (Camden County), 02.iv.2012, D. Hoffman (1♀, RSKM).

#### Non-barcoded material examined.

USA: **Florida**: A. Bolter (1♀, LACM); A.T. Solsson (1♂, AMNH); Alachua County, v.??49 (1♀, FMNH); Archbold Biological Station (Highlands County), 17–23.iv.2007, S.M. Paiero (1♂, DEBU); Archbold Biological Station (near Lake Annie, Highlands County), 14.iii.2016, M. Deyrup (1♂, ABS); Austin Cary Forest (Gainesville, Alachua County), 20.v.1976, G.B. Fairchild (1♀, UCBME); Dunedin (Pinellas County), 04.iv.1914 (2♀, AMNH); Gainesville (Alachua County), 02.iv.1976, W.H. Pierce (1♀, UCBME); Lake Louisa State Park (12 km S Clermont, Lake County), 05.iv.2014, K.A. Williams (2♀, FSCA); Lake Placid (Archbold Biological Station, Highlands County), 07.iv.1984, R.M. Bohart (2♂, UCBME); Leesburg (Lake County), 01–11.iii.1954, M. Statham (1♂, AMNH); N FWC Carter Creek (27.5313°N; 81.4104°W) (Highlands County), 11.v.2010, J. Dunlap, M. and N. Deyrup, and K. Dearborn (2♂, ABS).

##### Key to species of the genus *Epeolus* in Canada and the United States of America

**Table:** 

1	Axilla in dorsal view with tip extending to or beyond 2/3 the length of mesoscutellum (minimum posterior extent shown in Fig. 26D) (see also Figs 4D, 8D, 12D, 16D, 18D, 24D, 32D, 39D, 49D, 53D, 55D, 63D, 71D, 73D, 77D, 79D, 81D, 83D)	**2**
–	Axilla in dorsal view with tip extending to less than 2/3 the length of mesoscutellum (maximum posterior extent shown in Fig. 20D) (see also Figs 6D, 10D, 14D, 22D, 28D, 30D, 34D, 36D, 41D, 43D, 45D, 47D, 51D, 57D, 59D, 61D, 65D, 67D, 69D, 75D, 85D, 87D, 89D)	**21**
2	Head with frontal area bearing pair of granulose protrusions, each located near upper mesal margin of compound eye (Fig. 91A). T1 without apical fascia, usually with bright orange-yellow basal fascia; T2 with bright orange-yellow apical fascia (Fig. 26A–C)	***E. bifasciatus* Cresson**
–	Head with frontal area without protrusions (Fig. 91B). Metasomal terga with white to pale gray or pale yellow short appressed setae; IF with bright orange-yellow short appressed setae, THEN T1 with well-developed apical fascia (Figs 32A–C, 39A–C)	**3**
3	Axilla with free portion ~2/5 its entire medial length or longer (Figs 4D, 12D, 81D); IF borderline (0.35< *x* <0.4), THEN axilla with lateral margin relatively straight (Fig. 18D); IF borderline (0.35< *x* <0.4) and axilla with lateral margin arcuate, THEN axilla with free portion distinctly hooked (i.e., concave, not relatively straight along medial margin) (Figs 32D, 53D, 63D)	**4**
–	Axilla with free portion clearly less than 2/5 its entire medial length. Axilla with lateral margin usually distinctly arcuate. Figs 8D, 16D, 24D, 39D, 49D, 55D, 71D, 73D, 77D, 79D, 83D)	**11**
4	Mesopleuron with punctures in ventrolateral half sparse (most i>1d), the interspaces shining (Fig. 92A)	**5**
–	Mesopleuron with punctures in ventrolateral half dense (most i≤1d) or mesopleuron rugose with punctures ill-defined, the interspaces shining or dull due to surface microsculpture (Fig. 92B)	**7**
5	Metasomal terga with punctures large and deep (Fig. 93A). T2–T4 with fasciae complete and evenly broad (Fig. 65B)	***E. lectus* Cresson (in part)**
–	Metasomal terga with punctures minute and shallow (Fig. 93B). If fasciate, T2–T4 with fasciae conspicuously narrowed or interrupted medially (Fig. 63B)	**6**
6	Pronotal collar, axilla, mesoscutellum, and discs of T1 and T2 ferruginous. Metasomal terga with pale pubescence commonly reduced to discrete lateral patches. Fig. 53	***E. glabratus* Cresson**
–	At least pronotal collar and metasomal terga entirely black. Metasomal terga fasciate. Fig. 63A–C	***E. lectoides* Robertson**
7	Metanotum with distinct posteromedial depression (Fig. 94A). T2 fascia with lobe-like anterolateral extensions of tomentum (Fig. 18A–C) [west of Continental Divide]	***E. axillaris* sp. n.**
–	Metanotum without depression (Fig. 94B). T2 fascia without lobe-like anterolateral extensions of tomentum (Figs 4A–C, 32A–C, 81A–C), although fascia may be broader laterally with sparser pale hairs basally (Fig. 12A–C) [east of Continental Divide]	**8**
8	Head with preoccipital ridge joining hypostomal carina (approximately at 2/5 length of proboscidial fossa) (Fig. 95A). Mandible simple (assess only if mandible fully extended) (Fig. 3A)	***E. ainsliei* Crawford**
–	Head with preoccipital ridge not joining hypostomal carina (Fig. 95B). Mandible with small, obtuse preapical tooth (assess only if mandible fully extended) (Fig. 3B, C)	**9**
9	Mesoscutum with paramedian band. Metasomal fasciae bright yellow to brownish orange and interrupted medially. Fig. 32B [Southeastern United States]	***E. carolinus* Mitchell**
–	Mesoscutum largely obscured by pale tomentum in anterior half, tomentum densest anteromedially or evenly dense throughout mesoscutum. Metasomal fasciae off white to pale yellow and complete. Figs 12B, 81B [Great Plains and parts of American Southwest]	**10**
10	F2 of female less than 1.2 × as long as wide (Fig. 96A). T1 in dorsal view with longitudinal band more than half as wide as breadth of apical fascia (Fig. 12B)	***E. attenboroughi* sp. n.**
–	F2 of female more than 1.2 × as long as wide (Fig. 96B). T1 in dorsal view with discal patch so wide that longitudinal band barely visible (its width less than half the breadth of apical fascia) (Fig. 81B)	***E. rufulus* Cockerell**
11	T1–T3 with apical fasciae distinctly interrupted medially, T4 with fascia interrupted or narrowed medially (Figs 8B, 39B, 49B, 55B, 77B). Axilla and mesoscutellum ferruginous (Figs 8D, 39D, 49D, 55D, 77D)	**12**
–	T1–T3 with apical fasciae complete or only very narrowly interrupted medially, T4 with fascia complete (Figs 16B, 24B, 71B, 73B, 79B, 83B). Axilla and mesoscutellum color variable, may be entirely black (Fig. 16D) or partially to entirely ferruginous (Figs 24D, 71D, 73D, 79D, 83D)	**16**
12	T1 with basal fascia absent or reduced to pair of small patches of pale tomentum (Figs 39B, 77B). T5 with pseudopygidial area of female with apex more than twice as wide as medial length (Fig. 97A). T1 without longitudinal band (Figs 39A, C, 77A, C)	**13**
–	T1 with basal fascia well developed, complete or narrowly interrupted medially (Figs 8B, 49B, 55B). T5 with pseudopygidial area of female with apex less than twice as wide as medial length (Fig. 97B). T1 with (Figs 8C, 49C, 55A, C) or without (Figs 8A, 49A) longitudinal band	**14**
13	T1–T4 with apical fasciae brownish orange, at least medially (usually off white laterally) (Fig. 39A–C), those of T1 and T2 particularly well-developed. Mesopleuron commonly with punctures in ventrolateral half sparse (i≤2d), the interspaces shining or somewhat dull due to tessellate surface microsculpture (Fig. 92C) [adults active from late spring to early summer]	***E. deyrupi* sp. n.**
–	T1–T4 with bands of pale pubescence rather uniformly off white, usually reduced to discrete lateral patches that peter out medially (Fig. 77A–C). Mesopleuron with punctures in ventrolateral half dense (most i<1d) (Fig. 92D) [adults active in autumn]	***E. packeri* sp. n.**
14	Mesoscutum and metasomal terga with bands of pale gray to white short appressed setae. T1 with few exceptions ferruginous. Fig. 49A–C	***E. floridensis* Mitchell**
–	Mesoscutum and metasomal terga with bands of bright or pale yellow short appressed setae. T1 black. Figs 8A–C, 55A–C	**15**
15	Axilla with tip not extending as far back as posterior margin of mesoscutellum, mesoscutellum dark brown or black basally (Fig. 8D)	***E. andriyi* sp. n.**
–	Axilla with tip extending as far back as or beyond posterior margin of mesoscutellum, axilla and mesoscutellum entirely red (Fig. 55D)	***E. howardi* Mitchell**
16	Axilla with tip well short of band of pale tomentum along posterior margin of mesoscutellum (Fig. 16D), axilla and mesoscutellum entirely black. T2 fascia without anterolateral extensions of tomentum (Fig. 16A–C)	***E. autumnalis* Robertson**
–	Axilla with tip extending to or beyond band of pale tomentum along posterior margin of mesoscutellum (may be just short of band at apicomedial extent of mesoscutellum) (Figs 24D, 71D, 73D, 79D, 83D), axilla with few exceptions ferruginous to some degree. T2 fascia with (Figs 24A–C, 71A–C, 73A–C, 79A–C) or without (Fig. 83A, B) anterolateral extensions of tomentum	**17**
17	Mesopleuron of male obscured by white tomentum only in upper half (although hypoepimeral area usually with sparser tomentum), with a large, sparsely hairy circle occupying much of ventrolateral half (Fig. 83C). T5 with pseudopygidial area of female with apex clearly more than twice as wide as medial length (~2.5–3 × the medial length) (Fig. 97C). Axilla with tip extending to or beyond band of pale tomentum along posterior margin of mesoscutellum, mesoscutellum entirely black to entirely ferruginous (Fig. 83D)	***E. scutellaris* Say**
–	Mesopleuron of male (excluding hypoepimeral area) entirely obscured by white tomentum (Figs 24C, 71C, 73C, 79C). T5 with pseudopygidial area of female with apex about twice as wide as medial length or less (clearly <2.5 × the medial length) (Fig. 97D, E). Axilla with tip at most extending to band of pale tomentum along posterior margin of mesoscutellum, mesoscutellum entirely black (Figs 24D, 71D, 73D, 79D)	**18**
18	Flagellum, except sometimes F1, and metasomal sterna (excluding apical margins) brown or black, clearly not the same reddish-orange color as legs from tibiae to tarsi (Fig. 98A). T1 with longitudinal extent of discal patch no less (and usually greater) than breadth of apical fascia (Fig. 79B). T1–T3 with apical fasciae removed from apical margin, commonly narrowed or narrowly interrupted medially (Fig. 79A–C) [southern Canada and much of contiguous U.S., east of the Rocky Mountains]	***E. pusillus* Cresson**
–	Metasomal sterna reddish brown or reddish orange (Fig. 98B); IF brown or black, THEN rarely entire flagellum also brown or black. T1 with longitudinal extent of discal patch variable, but may be less than breadth of apical fascia (Figs 71B, 73B). T1–T3 with apical fasciae on apical margin and evenly broad (Figs 71A–C, 73A–C) or as above (Fig. 24A–C) [U.S., Great Plains to West Coast]	**19**
19	T2 and T3 (for female) or T2–T4 (for male) with fasciae removed from apical margin, commonly narrowed or narrowly interrupted medially (Fig. 24A–C). T5 with pseudopygidial area of female with apex at least twice as wide as medial length (Fig. 97D). T1 with longitudinal extent of discal patch greater than breadth of apical fascia, at least medially (Fig. 24B)	***E. basili* sp. n.**
–	T2–T4 with fasciae on or very little removed from apical margin, more or less evenly broad (Figs 71A–C, 73A–C). T5 with pseudopygidial area of female with apex commonly less and no more than twice as wide as medial length (Fig. 97E). T1 with longitudinal extent of discal patch variable, but commonly less than breadth of apical fascia (Figs 71B, 73B)	**20**
20	Metasomal terga (excluding brown translucent apical margins) black (Figs 71B, 99A). Mesoscutum obscured by pale tomentum (Fig. 71B, C)	***E. nebulosus* sp. n.**
–	At least T1 with integument beneath apical fascia ferruginous (Fig. 99B), T1 basally and other terga sometimes partially to entirely ferruginous as well (Fig. 73B). Mesoscutum with well-defined paramedian band (Fig. 73B) or obscured by pale tomentum	***E. novomexicanus* Cockerell**
21	Head with vertexal area with two pairs of shiny (usually impunctate) protrusions (Fig. 91C). T2 fascia with two pairs of anterolateral extensions of tomentum (Figs 1, 34A–C, 41A, C)	**22**
–	Head with vertexal area without protrusions (Fig. 91D). T2 fascia with single pair of anterolateral extensions of tomentum (Figs 6A–C, 10A–C, 14A–C, 20A, C, 28A–C, 43A–C, 51A–C, 59A–C, 61A–C, 65A–C, 69A–C, 75A–C, 87A–C) or without (Figs 22A–C, 30A–C, 36A–C, 38, 45A–C, 47A–C, 57A–C, 67A–C, 85A–C, 89A–C) anterolateral extensions of tomentum	**23**
22	Mesopleuron with punctures in ventrolateral half sparse (most i>1d), the interspaces shining (Fig. 92I) [Southwestern United States]	***E. chamaesarachae* sp. n.**
–	Mesopleuron with punctures in ventrolateral half dense (most i≤1d) (Fig. 92J) [Coastal and South Texas]	***E. diadematus* sp. n.**
23	Axilla with free portion about 2/5 its medial length or longer and distinctly hooked (i.e., concave, not relatively straight along medial margin) (minimum free extent shown in Fig. 65D) (see also Figs 43D, 51D, 57D, 59D, 89D). T5 with pseudopygidial area of female distinctly campanulate, with apex less than twice as wide as medial length (Fig. 97F–H)	**24**
–	Axilla with free portion less than 2/5 its entire medial length (usually ≤ 1/3) and relatively straight along medial margin (maximum free extent shown in Fig. 75D) (see also Figs 6D, 10D, 14D, 20D, 22D, 28D, 30D, 36D, 45D, 47D, 61D, 67D, 69D, 85D, 87D). T5 with pseudopygidial area of female lunate (Fig. 97I) or present as very narrow transverse band (Fig. 61B), with apex at least twice as wide as medial length	**29**
24	Axilla and mesoscutellum entirely ferruginous. T1 and T2 ferruginous. Fig. 89	***E. zonatus* Smith**
–	Axilla and mesoscutellum at least partially dark brown or black. T1 and T2 black. Figs 43, 51, 57, 59, 65	**25**
25	Mesopleuron very coarsely and densely rugose-punctate AND punctures of varying size, few if any interspaces as large as puncture diameters (Fig. 92E)	***E. erigeronis* Mitchell**
–	Mesopleuron with larger interspaces (i≈1d) typically more numerous (Fig. 92A, F); IF most interspaces small (i<1d), THEN mesopleuron more finely and minutely punctate AND punctures of similar size throughout mesopleuron (Fig. 92G)	**26**
26	Metasomal terga with punctures large and deep (Fig. 93A). T2–T4 with fasciae complete and evenly broad (Fig. 65A–C)	***E. lectus* Cresson (in part)**
–	Metasomal terga with punctures minute and shallow (Fig. 93C). T2–T4 with fasciae commonly narrowed or interrupted medially (Figs 51B, 57B, 59B)	**27**
27	Mandible with blunt, obtuse preapical tooth (Fig. 3D). F2 of female less than 1.2 × as long as wide (Fig. 96C). Legs with brown or black more extensive than reddish orange, at least from metacoxa to metatibia (Fig. 51A, C). S4 and S5 of male with long curved coppery to silvery subapical hairs, many extending beyond apex of sternum by 1 MOD or more (Fig. 51C). T5 of female with two large patches of pale tomentum parallel to and contacting pseudopygidial area nearly throughout its length (Fig. 97F)	***E. gibbsi* sp. n.**
–	Mandible simple (Fig. 3E). F2 of female more than 1.2 × as long as wide (Fig. 96D). Legs extensively reddish orange (Fig. 57A, C) or brown or black (Fig. 59A, C); IF male and legs with brown or black more extensive than reddish orange from metacoxa to metatibia, THEN S4 and S5 with short straight subapical hairs, extending little (clearly by <1 MOD) if at all beyond apex of sternum (Fig. 100B). T5 of female with two large patches of pale tomentum lateral to and separate from pseudopygidial area, or contacting pseudopygidial area at apex, diverging from it basally (Fig. 97G)	**28**
28	Pronotal lobe and legs more extensively reddish orange than brown or black, metatibia with anterior surface same reddish orange color as metatarsus (Fig. 57A, C). Pronotal collar, mesoscutum, and metasomal terga with bands of gray to pale yellow short appressed setae (Fig. 57). S4 and S5 of male with long curved coppery to silvery subapical hairs, many extending beyond apex of sternum by 1 MOD or more (Fig. 100A)	***E. ilicis* Mitchell**
–	Pronotal lobe black to partially or entirely reddish orange. Legs usually darker (with brown or black more extensive than reddish orange), at least from metacoxa to metatibia (Fig. 59A, C). Pronotal collar, mesoscutum, and metasomal terga with bands of gray short appressed setae (Fig. 59). S4 and S5 of male with short straight subapical hairs, extending little (clearly by <1 MOD) if at all beyond apex of sternum (Fig. 100B)	***E. inornatus* sp. n.**
29	Mesoscutum with anteromedial patch of bright or pale yellow tomentum, usually chevron-, horseshoe-, or V-shaped and narrowed anterolaterally (Figs 30B, 36B, 38, 45B) but sometimes semicircular (Fig. 85B)	**30**
–	Mesoscutum with gray or bright to pale yellow paramedian band (usually parallel and not joined except sometimes posteriorly) (Figs 6B, 10B, 14B, 20B, 28B, 47B, 61B, 67B, 69B, 75B, 87B) or largely obscured by pale tomentum (Fig. 101B); IF joined posteriorly (i.e., U- or V-shaped), THEN not distinctly narrowed anterolaterally (Fig. 101A)	**33**
30	Propodeum with posterior surface highly polished and (except along lateral margin) hairless (Fig. 102A). T1 with broad, transverse off-white basal fascia, discal patch greatly reduced or absent; T1–T4 with complete bright yellow apical fasciae, terga otherwise covered in brown (and laterally sometimes off-white) tomentum (Fig. 85A–C)	***E. splendidus* sp. n.**
–	Propodeum with posterior surface dull due to surface microsculpture and with long erect hairs submedially (Fig. 102B). T1 with median black or nearly black discal patch surrounded by pale tomentum; T1–T4 with complete or medially-interrupted pale yellow apical fasciae, terga otherwise covered in black or nearly black tomentum (Figs 30A–C, 36A–C, 38B, 45A–C)	**31**
31	T1 discal patch triangular or semicircular (with lateral sides straight or convex), basal fascia fully continuous with longitudinal band AND discal patch more elongate, its medial longitudinal extent (measured as if apical fascia were complete) more than 1/3 the lateral extent. Fig. 30B	***E. canadensis* Mitchell**
–	T1 discal patch quadrangular (basal and apical fasciae subparallel and separated by longitudinal band) (Figs 36B, 45B) or diamond-shaped (Fig. 38) with basal and apical fasciae broadly joined laterally; IF discal patch almost semicircular, THEN shorter, its medial longitudinal extent (measured as if apical fascia were complete) at most 1/3 the lateral extent	**32**
32	T2–T4 with fasciae broadened before becoming narrowed or separated into rounded lobes medially, and usually narrowed before becoming somewhat broader laterally (Figs 36B, 38)	***E. compactus* Cresson**
–	T2–T4 with fasciae not broadened into rounded lobes medially, and somewhat broader laterally and complete or tapering until separated medially (Fig. 45B)	***E. ferrarii* sp. n.**
33	T1 with median triangular or semicircular discal patch (basal fascia conspicuously arched, apical fascia straight) AND longitudinal band at least half as wide as breadth of apical fascia in dorsal view (Fig. 47B)	***E. flavofasciatus* Smith**
–	T1 not as above; IF discal patch triangular, THEN so wide that longitudinal band barely visible in dorsal view (its width less than half the breadth of apical fascia) (Fig. 61B)	**34**
34	T2–T4 with apical fasciae complete, evenly broad (Figs 14B, 28B, 67B)	**35**
–	T1 and T2 with apical fasciae broken or at least greatly narrowed medially, those of T3 and T4 broken or complete (Figs 6B, 10B, 20B, 22B, 61B, 69B, 75B, 87B)	**37**
35	Fore wing with two submarginal cells, apically dusky in female, hyaline throughout in male (Fig. 67A, C). Axilla with free portion commonly less than 1/4 as long as its entire medial length (Fig. 67D). Mesopleuron almost entirely obscured by tomentum, at least in male (Fig. 67C). Axilla and mesoscutellum black (Fig. 67)	***E. mesillae* (Cockerell)**
–	Fore wing with three submarginal cells, subhyaline, apically dusky in both sexes (Fig. 14A, 28A). Axilla with free portion at least 1/4 as long as its entire medial length (Figs 14D, 28D). Mesopleuron obscured by tomentum only in upper half, with a large, sparsely hairy circle occupying much of ventrolateral half (Figs 14A, C, 28A, C). At least axilla ferruginous in part (Figs 14A, B, D, 28B, D)	**36**
36	Frontal carina strongly convex, such that supraclypeal area distinctly protuberant in lateral view (Fig. 103A). Pygidial plate of male narrow, with medial length ~1.5 × basal width (Fig. 2A). T2 fascia with anterolateral extensions of tomentum strongly convergent basally (angle from apical fascia <45°) (Fig. 14B).	***E. australis* Mitchell**
–	Frontal carina weakly convex, such that supraclypeal area barely protuberant in lateral view (Fig. 103B). Pygidial plate of male broad, with medial length ≈ basal width (Fig. 2B). T2 fascia with anterolateral extensions of tomentum not so strongly convergent basally (angle from apical fascia 45° to 90°) (Fig. 28B)	***E. brumleyi* sp. n.**
37	Mesopleuron with punctures in ventrolateral half well separated (i>1d), usually upper half more densely punctate than ventrolateral half (Fig. 92H). Axilla and mesoscutellum (except sometimes in *E. interruptus*) ferruginous (Figs 61, 87). T1 with discal patch variable; IF forming rounded triangle with lateral sides concave (Fig. 61B), THEN mesosomal features may exhibit alternative states (see below)	**38**
–	Mesopleuron with most interspaces between punctures small (i≤1d) (Fig. 92K) or mesopleuron rugose, with punctures ill-defined (Fig. 92L). Axilla (except sometimes the tip) and mesoscutellum black (Figs 6, 10, 20, 22, 69, 75). T1 with discal patch quadrangular (Figs 6B, 10B, 20B, 22B, 69B, 75B)	**39**
38	Metanotum with blunt median process, usually covered in pale tomentum but visible nonetheless (Fig. 61D). T1 with discal patch forming rounded triangle with lateral sides concave (Fig. 61B)	***E. interruptus* Robertson**
–	Metanotum without process (Fig. 87D). T1 with discal patch trapezoidal, sometimes almost semicircular, with lateral sides not distinctly concave (Fig. 87B)	***E. tessieris* sp. n.**
39	F2 of female at most 1.1 × as long as wide (Fig. 96E). Axilla with free portion at most 1/4 as long as its entire medial length (Figs 6D, 10D, 22D). T2 fascia with (Figs 6A–C, 10A–C) or without (Fig. 22A–C) anterolateral extensions of tomentum	**40**
–	F2 of female at least 1.2 × as long as wide (Fig. 96F). Axilla with free portion more than 1/4 as long as its entire medial length (Figs 20D, 69D, 75D). T2 fascia with anterolateral extensions of tomentum (Figs 20A, C, 69A–C, 75A–C)	**42**
40	Legs extensively reddish orange, at least from tibiae to tarsi (sometimes trochanters and femora as well), pronotal lobe reddish orange (Fig. 22A–C). T3 and T4 with fasciae complete or interrupted but not reduced to separated circular patches of pale tomentum (Fig. 22B)	***E. barberiellus* Cockerell**
–	Legs brown or black, pronotal lobe black to partially or entirely reddish orange (Figs 6A–C, 10A–C). T3 and T4 with fasciae complete (Fig. 6B) or broken medially and/or laterally, and may be reduced to widely separated circular patches of pale tomentum (Fig. 10B)	**41**
41	Mesopleuron (Fig. 92K) and tegula with many punctures widely separated (i=1d). Pronotal lobe dark brown to black (Fig. 6A, C). T3 and T4 with fasciae complete (Fig. 6B) or broken medially and/or laterally (Fig. 6A, C), rarely into separated oval patches [widespread throughout North America]	***E. americanus* (Cresson)**
–	Mesopleuron (Fig. 92L) and tegula with punctures very dense (most i<1d). Pronotal lobe black to partially or entirely reddish orange (Fig. 10A, C). T3 and T4 with fasciae broken or at least greatly narrowed laterally, as well as medially into separated or narrowly connected oval patches (Fig. 10B) [California and possibly surrounding states]	***E. asperatus* Cockerell**
42	T3 and T4 with fasciae broken or at least narrowed laterally, as well as medially. Pronotal lobe reddish orange. Fig. 75A–C	***E. olympiellus* Cockerell**
–	T3 and T4 with fasciae not broken laterally, and complete or narrowly interrupted medially (Figs 20A–C, 69A–C). Pronotal lobe black (Figs 20A–C, 69C) to partially or entirely reddish orange (Fig. 69A)	**43**
43	Integument entirely dark brown or black AND mesoscutum and metasomal terga with bands of gray short appressed setae (Fig. 20) [Mid-Atlantic and Southeastern United States]	***E. banksi* (Cockerell)**
–	Integument dark brown or black to partially or entirely ferruginous on labrum, antenna, pronotal lobe, and legs, except foreleg, from trochanters to tarsi. Mesoscutum and metasomal terga with bands of off-white to pale yellow short appressed setae. Fig. 69 [widespread throughout North America]	***E. minimus* (Robertson)**

**Figure 91.**
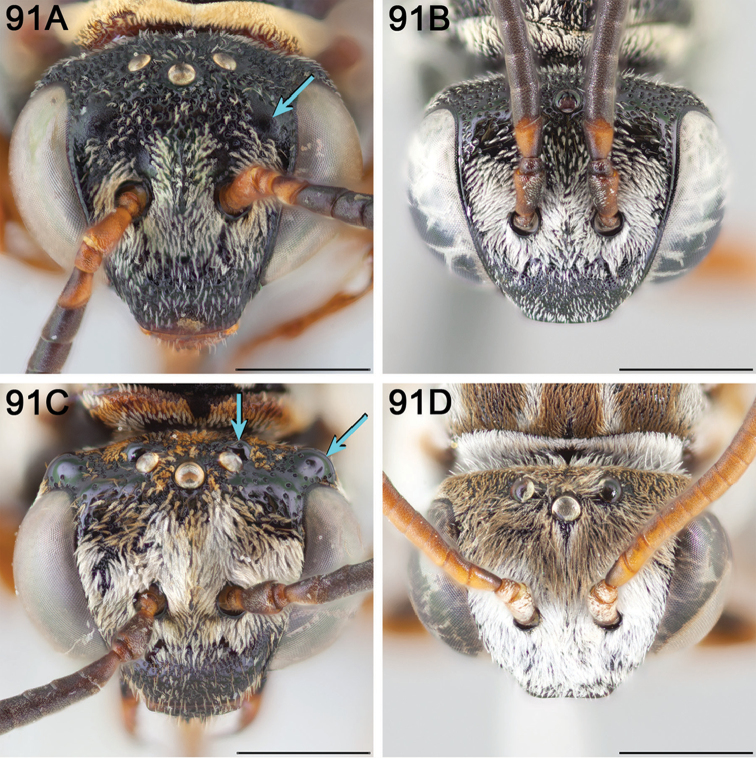
Head of female **A** *E. bifasciatus* showing frontal area with pair of granulose protrusions **B** *E. lectus* showing frontal area without protrusions **C** *E. chamaesarachae* paratype showing vertexal area with four shiny, impunctate protrusions, and **D** *E. mesillae* showing vertexal area without protrusions. Scale bars 1 mm.

**Figure 92.**
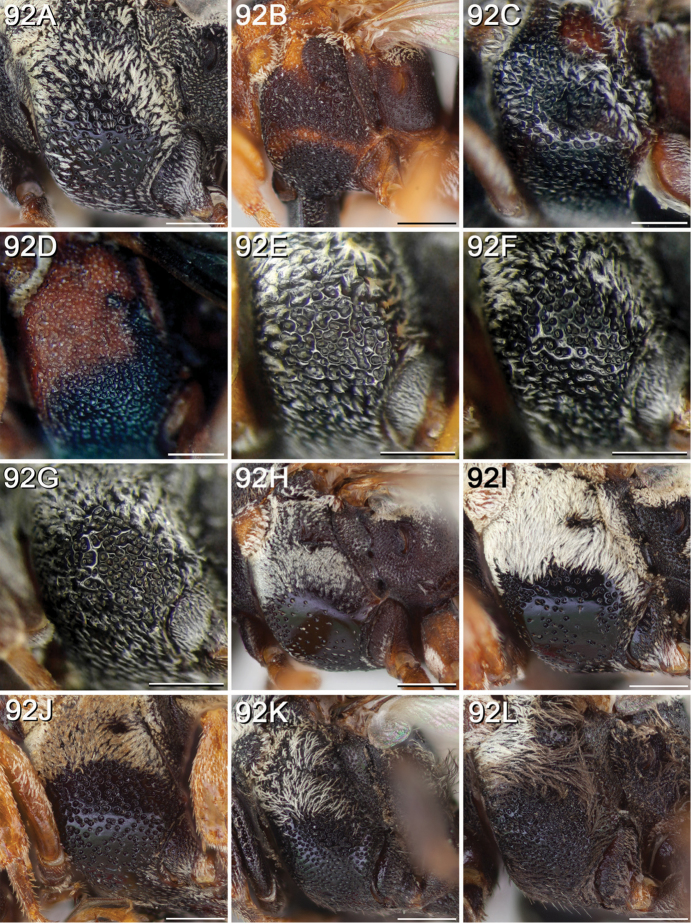
Mesopleuron (lateral view) of female **A** *E. lectus* showing sparse punctation (most i>1d) **B** *E. carolinus* showing dense punctation (most i≤1d) **C** *E. deyrupi* paratype showing moderately sparse punctation (i≤2d) **D** *E. packeri* paratype showing moderately dense punctation (most i<1d) **E** *E. erigeronis* showing very dense punctation (few if any interspaces as large as puncture diameters) **F** *E. ilicis* showing moderately dense punctation (i≤1d) **G** *E. inornatus* paratype showing moderately dense punctation (i≤1d) **H** *E. tessieris* paratype showing very sparse punctation (most i>1d) **I** *E. chamaesarachae* paratype showing very sparse punctation (most i>1d) **J** *E. diadematus* paratype showing sparse punctation, but punctures denser (many i≤1d) relative to *E. chamaesarachae* **K** *E. americanus* showing moderately dense punctation, with most punctures clearly separated (i=1d) and the interspaces shining; and **L** *E. asperatus* showing very dense punctation (most i<1d). Scale bars 0.5 mm.

**Figure 93.**
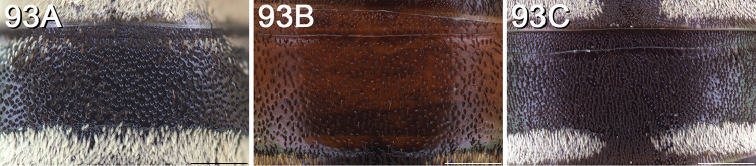
T2 (medial portion in dorsal view) of female **A** *E. lectus* with punctures coarse and deep **B** *E. glabratus* with punctures minutes and shallow, and **C** *E. inornatus* paratype with punctures minute and shallow. Scale bars 0.5 mm.

**Figure 94.**
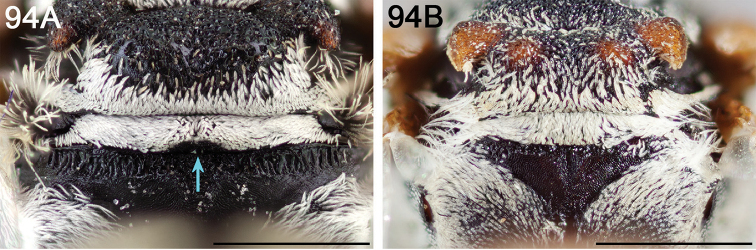
Metanotum (in posterior view) of female **A** *E. axillaris* holotype, which has a distinct posteromedial depression, and **B** *E. attenboroughi* paratype, which does not have a depression and is flat. Scale bars 1 mm.

**Figure 95.**
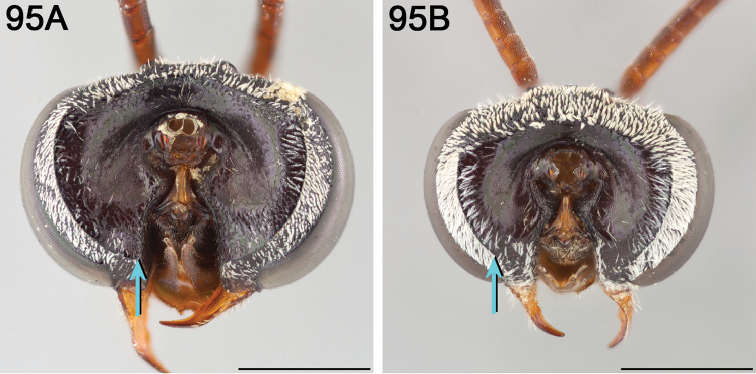
Head (in posterior view) removed from female **A** *E. ainsliei*, in which the preoccipital ridge joins the hypostomal carina, and **B** *E. attenboroughi* holotype, in which the preoccipital ridge does not join the hypostomal carina. Scale bars 1 mm. Note that these features can be seen without having to detach the head.

**Figure 96.**
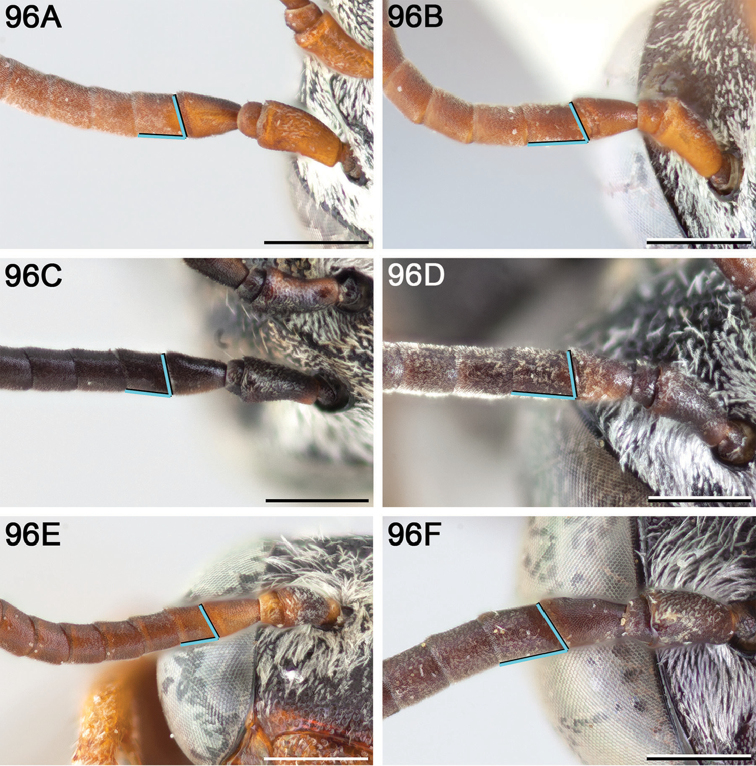
Antennae (basal portion) of female *Epeolus* spp. illustrating relative length to width of F2: **A** *E. attenboroughi* paratype, with F2 not noticeably longer than wide **B** *E. rufulus* holotype, with F2 noticeably longer than wide **C** *E. gibbsi* holotype, with F2 not noticeably longer than wide **D** *E. inornatus* paratype, with F2 noticeably longer than wide **E** *E. barberiellus*, with F2 as wide as long, or nearly so, and **F** *E. banksi*, with F2 noticeably longer than wide. Scale bars 0.5 mm.

**Figure 97.**
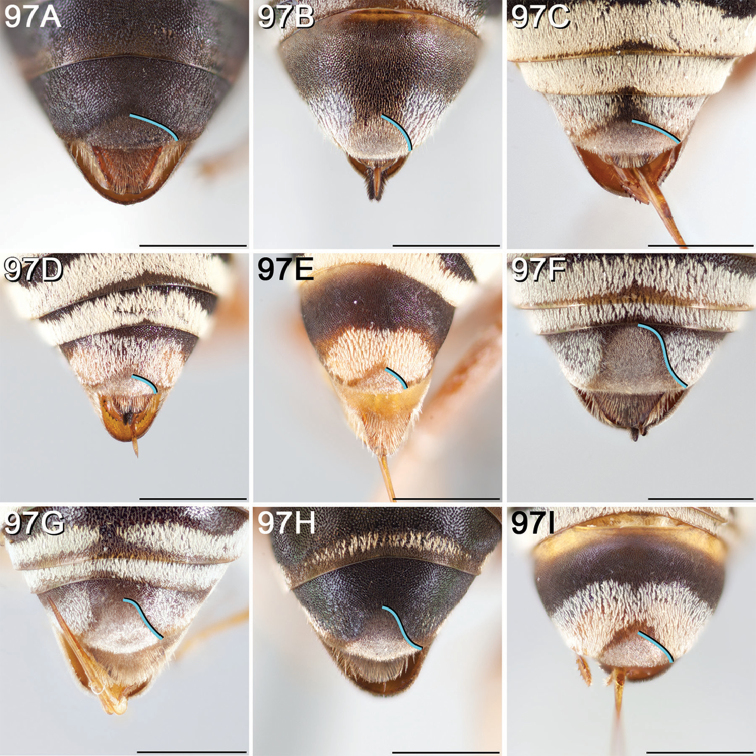
Pseudopygidial area (in dorsal view) of female **A** *E. packeri* paratype (lunate and wider than long) **B** *E. floridensis* (lunate and nearly as long as wide) **C** *E. scutellaris* (lunate and wider than long) **D** *E. basili* paratype (lunate and wider than long) **E** *E. novomexicanus* (lunate and somewhat wider than long) **F** *E. gibbsi* paratype (campanulate and nearly as long as wide) **G** *E. ilicis* (campanulate and nearly as long as wide) **H** *E. zonatus* (campanulate and nearly as long as wide), and **I** *E. australis* (lunate and wider than long). Scale bars 1 mm. The pseudopygidial area is the apical portion of T5 that changes slope from the rest of the tergum and is covered in short, silvery hairs uniform in length (posteromesad the light blue lines).

**Figure 98.**
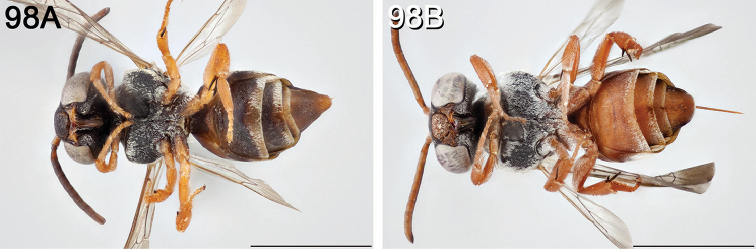
Female **A** *E. pusillus*, ventral habitus, showing color contrast between the dark brown antennae and metasomal sterna and the reddish-orange legs, and **B** *E. basili* paratype, ventral habitus, showing antennae, legs, and metasomal sterna with similar reddish-orange coloration.

**Figure 99.**
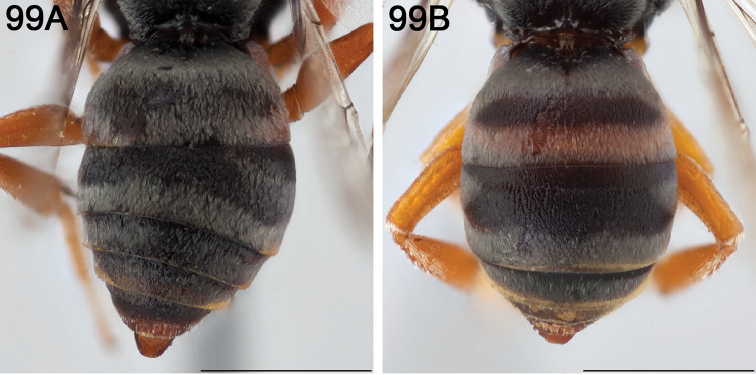
Metasoma (in dorsal view) dampened with water to show differences in integument coloration between T1 of male **A** *E. nebulosus* paratype, which is entirely black, and **B** *E. novomexicanus*, which is red beneath the apical fascia. Scale bars 2 mm. Note that lightly wetting the terga with ethanol allows for this feature to be seen without having to remove the tomentum.

**Figure 100.**
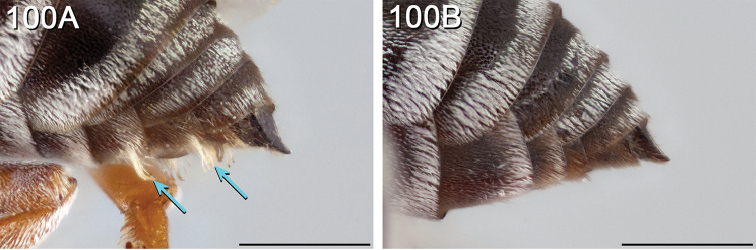
Metasoma (in lateral view) of male **A** *E. ilicis* showing long curved subapical hairs on S4 and S5 and **B** *E. inornatus* allotype showing very short straight subapical hairs on the same sterna.

**Figure 101.**
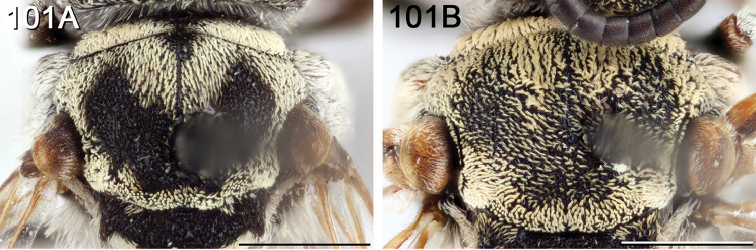
Mesoscutal pubescence (dorsal view) in males of *E. minimus* **A** as paramedian bands joined apically and **B** entirely obscuring the integument. Scale bars 1 mm.

**Figure 102.**
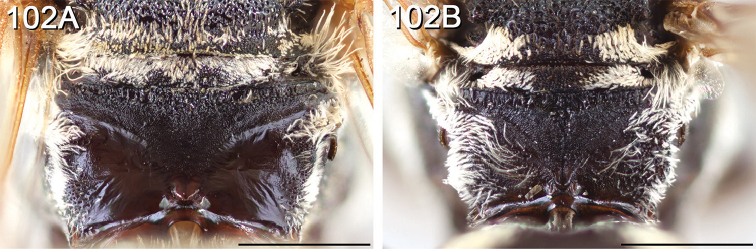
Propodeum (in posterior view) of female **A** *E. splendidus* paratype and **B** *E. canadensis*. Scale bars 1 mm.

**Figure 103.**
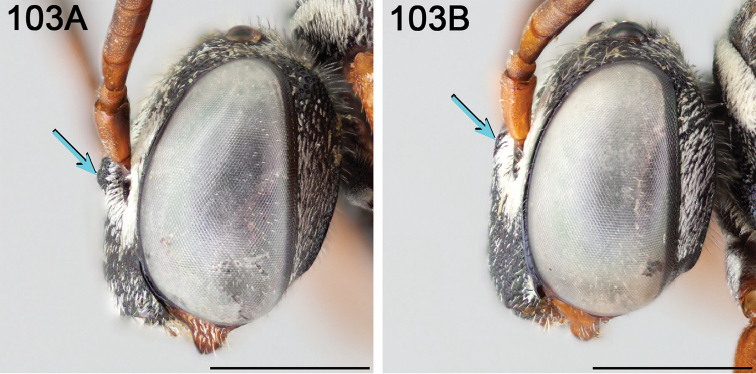
Head (in lateral view) of female **A** *E. australis*, in which the frontal keel is strongly raised, and **B** *E. brumleyi* paratype, in which the frontal keel is only weakly protuberant. Scale bars 1 mm. Note that the supraclypeal area is usually covered in dense white tomentum, which was partially removed in these specimens to show the maximum extent of the keel.

## Supplementary Material

XML Treatment for Epeolus

XML Treatment for Epeolus ainsliei

XML Treatment for Epeolus americanus

XML Treatment for Epeolus andriyi

XML Treatment for Epeolus asperatus

XML Treatment for Epeolus attenboroughi

XML Treatment for Epeolus australis

XML Treatment for Epeolus autumnalis

XML Treatment for Epeolus axillaris

XML Treatment for Epeolus banksi

XML Treatment for Epeolus barberiellus

XML Treatment for Epeolus basili

XML Treatment for Epeolus bifasciatus

XML Treatment for Epeolus brumleyi

XML Treatment for Epeolus canadensis

XML Treatment for Epeolus carolinus

XML Treatment for Epeolus chamaesarachae

XML Treatment for Epeolus compactus

XML Treatment for Epeolus deyrupi

XML Treatment for Epeolus diadematus

XML Treatment for Epeolus erigeronis

XML Treatment for Epeolus ferrarii

XML Treatment for Epeolus flavofasciatus

XML Treatment for Epeolus floridensis

XML Treatment for Epeolus gibbsi

XML Treatment for Epeolus glabratus

XML Treatment for Epeolus howardi

XML Treatment for Epeolus ilicis

XML Treatment for Epeolus inornatus

XML Treatment for Epeolus interruptus

XML Treatment for Epeolus lectoides

XML Treatment for Epeolus lectus

XML Treatment for Epeolus mesillae

XML Treatment for Epeolus minimus

XML Treatment for Epeolus nebulosus

XML Treatment for Epeolus novomexicanus

XML Treatment for Epeolus olympiellus

XML Treatment for Epeolus packeri

XML Treatment for Epeolus pusillus

XML Treatment for Epeolus rufulus

XML Treatment for Epeolus scutellaris

XML Treatment for Epeolus splendidus

XML Treatment for Epeolus tessieris

XML Treatment for Epeolus zonatus

## Supplementary materials

Supplementary material 1 Database of *Epeolus* records Data type: occurrence Explanation note: A comprehensive compilation of Nearctic *Epeolus* records used to estimate species ranges, presented for each species as a map showing the known extent of occurrence. Most records are of personally examined specimens, though some are taken from literature or online sources. Specimens used in DNA barcoding include sample processing IDs, barcode index numbers are given for sequences that are BIN-compliant, and GenBank accession numbers are presented for sequenced specimens. This is an open access article distributed under the terms of the Creative Commons Attribution License (CC BY 4.0), which permits unrestricted use, distribution, and reproduction in any medium, provided the original author and source are credited.Author: Thomas M. Onuferko

Supplementary material 2 Neighbor-joining tree of DNA barcode sequences Data type: molecular data Explanation note: A Neighbor-Joining tree of 181 Nearctic *Epeolus* COI sequences >300 bp in length (no BINs were assigned to sequences <300 bp in length) based on Kimura’s two-parameter distance model, generated in BOLDSYSTEMS (http://www.boldsystems.org) and presented in the Multipage Classic format. For each sample, the country and province or state in which the specimen that was the source of genetic material was collected is given, as is the processing ID and (if applicable) BIN. Detailed collection information for each sequenced specimen is presented in Suppl. material 1. This is an open access article distributed under the terms of the Creative Commons Attribution License (CC BY 4.0), which permits unrestricted use, distribution, and reproduction in any medium, provided the original author and source are credited.Author: Thomas M. Onuferko

Supplementary material 3 Morphological key Data type: species data Explanation note: Modifications to the key to species of *Epeolus* in Canada of Onuferko (2017) to include *E. gibbsi*, which has been discovered since from a specimen collected in Manitoba, and *E. inornatus*, which might occur in the country given its presence in New England. The modified key picks up from the second half of couplet 3 in the original key, and unless otherwise stated cites figures published in the present article. This is an open access article distributed under the terms of the Creative Commons Attribution License (CC BY 4.0), which permits unrestricted use, distribution, and reproduction in any medium, provided the original author and source are credited.Author: Thomas M. Onuferko
