# Supplementary figures and images for: A revision of the cleptoparasitic bee genus Epeolus Latreille for Nearctic species, north of Mexico (Hymenoptera, Apidae)
Source: Zookeys. 2018 May 8;(755):1–185. doi: 10.3897/zookeys.755.23939 (PMC5953965; doi:10.3897/zookeys.755.23939)

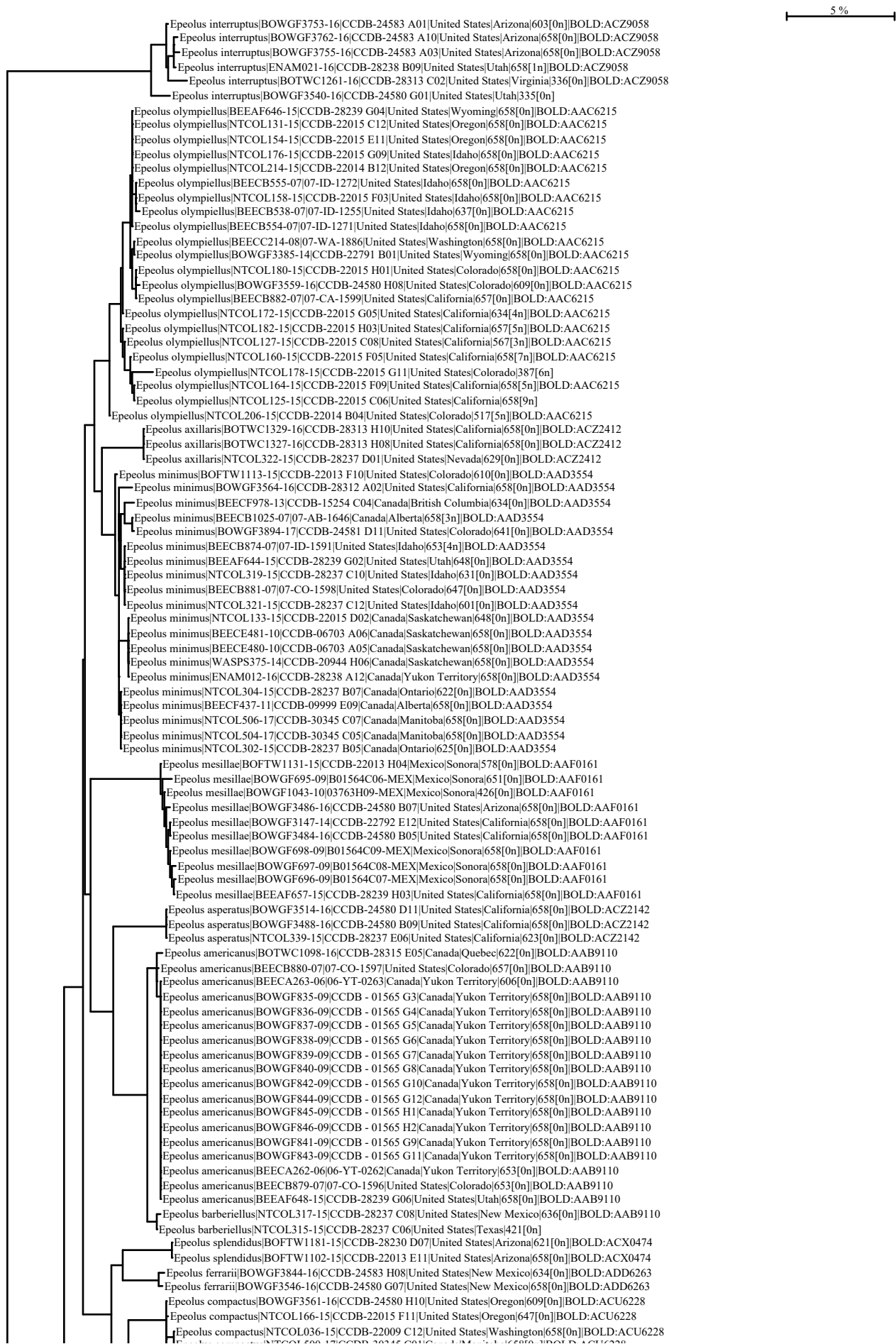

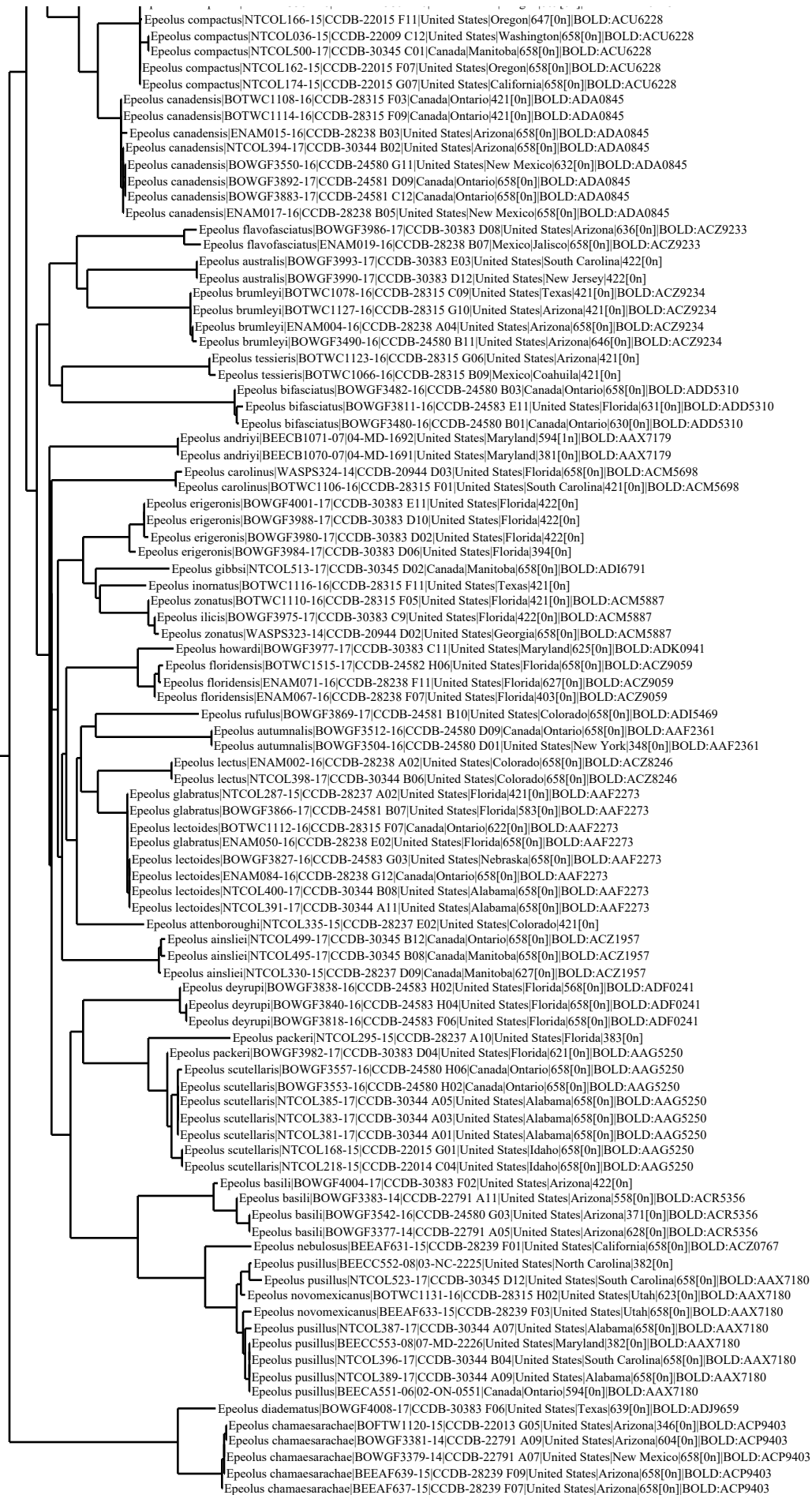

Supplement: Supplementary material 2 — Neighbor-joining tree of DNA barcode sequences [file zookeys-755-001-s002.pdf]
