# Supplementary material for: A revision of the cleptoparasitic bee genus Epeolus Latreille for Nearctic species, north of Mexico (Hymenoptera, Apidae)
Source: Zookeys. 2018 May 8;(755):1–185. doi: 10.3897/zookeys.755.23939 (PMC5953965; doi:10.3897/zookeys.755.23939)
Supplement: Supplementary material 3 — Morphological key [file zookeys-755-001-s003.docx]

1. Axilla with free portion about 2/5 its medial length or longer and distinctly hooked (i.e. not relatively straight along medial margin) (Onuferko 2017, Fig. 5D & E) 5

- Axilla with free portion less than 2/5 its entire medial length (usually ≤ 1/3) and relatively straight along medial margin (Onuferko 2017, Fig. 5F) couplet 6 of the original key

1. (Onuferko 2017)
2. Head with preoccipital ridge joining hypostomal carina (approximately at 2/5 length of proboscidial fossa) (Onuferko 2017, Fig. 11A). T5 with pseudopygidial area of female lunate, with apex at least twice as wide as medial length (Onuferko 2017, Fig. 3B) ***E. ainsliei* Crawford**

- Head with preoccipital ridge not joining hypostomal carina (Onuferko 2017, Fig. 11B). T5 with pseudopygidial area of female distinctly campanulate, with apex less than twice as wide as medial length (Onuferko 2017, Fig. 3A) 6

1. Mandible with blunt, obtuse preapical tooth (Fig. 3D). F2 of female less than 1.2 × as long as wide (Fig. 96C). Legs with brown or black more extensive than reddish orange, at least from metacoxa to metatibia (Fig. 51A & C). S4 and S5 of male with long curved coppery to silvery subapical hairs, many extending beyond apex of sternum by 1 MOD or more (Fig. 51C). T5 of female with two large patches of pale tomentum parallel to and contacting pseudopygidial area nearly throughout its length (Fig. 97F) ***E. gibbsi* sp. n.**

- Mandible simple (Fig. 3E). F2 of female more than 1.2 × as long as wide (Fig. 96D). Legs extensively reddish orange (Fig. 57A & C) or brown or black (Fig. 59A & C); IF male and legs with brown or black more extensive than reddish orange from metacoxa to metatibia, THEN S4 and S5 with short straight subapical hairs, extending little (clearly by <1 MOD) if at all beyond apex of sternum (Fig. 100B). T5 of female with two large patches of pale tomentum lateral to and separate from pseudopygidial area, or contacting pseudopygidial area at apex, diverging from it basally (Fig. 97G) 7

1. Pronotal lobe and legs more extensively reddish orange than brown or black, metatibia with anterior surface same reddish orange color as metatarsus (Fig. 57A & C). Pronotal collar, mesoscutum, and metasomal terga with bands of gray to pale yellow short appressed setae (Fig. 57). S4 and S5 of male with long curved coppery to silvery subapical hairs, many extending beyond apex of sternum by 1 MOD or more (Fig. 100A) ***E. ilicis* Mitchell**

- Pronotal lobe black to partially or entirely reddish orange. Legs usually darker (with brown or black more extensive than reddish orange), at least from metacoxa to metatibia (Fig. 59A & C). Pronotal collar, mesoscutum, and metasomal terga with bands of gray short appressed setae (Fig. 59). S4 and S5 of male with short straight subapical hairs, extending little (clearly by <1 MOD) if at all beyond apex of sternum (Fig. 100B) ***E. inornatus* sp. n.**
